# A revision of the “African Non-Spiny” Clade of *Solanum* L. (*Solanum* sections *Afrosolanum* Bitter, *Benderianum* Bitter, *Lemurisolanum* Bitter, *Lyciosolanum* Bitter, *Macronesiotes* Bitter, and *Quadrangulare* Bitter: Solanaceae)

**DOI:** 10.3897/phytokeys.66.8457

**Published:** 2016-07-13

**Authors:** Sandra Knapp, Maria S. Vorontsova

**Affiliations:** 1 Department of Life Sciences, Natural History Museum, Cromwell Road, London SW7 5BD, United Kingdom; 2 Comparative Plant and Fungal Biology, Royal Botanic Gardens, Kew, Richmond, Surrey TW7 3AB, United Kingdom

**Keywords:** Africa, classification, Madagascar, monograph, morphological variation, nomenclature, Solanum, vines, wet forests, widespread species

## Abstract

The African Non-Spiny (ANS) clade contains 14 species of mostly large canopy lianas or scandent shrubs confined to Madagascar (10) and continental Africa (4, with with one species reaching the southern Arabian peninsula). Members of the clade were previously classified in sections *Afrosolanum* Bitter, *Benderianum* Bitter, *Lemurisolanum* Bitter, *Macronesiotes* Bitter and *Quadrangulare* Bitter, and were throught to be related to a variety of New World groups. The group is an early-branching lineage of non-spiny solanums and characters shared with other vining New World solanums are homoplastic. The 14 species of the group occupy a wide range of habitats, from wet forests in western Africa to savanna and dry forests of southern Madagascar and dune habitats in South Africa. Many members of the group are highly variable morphologically, and habit can vary between shrub and canopy vine in a single locality. We here review the taxonomic history, morphology, potential relationships and ecology of these species; we provide keys for their identification, descriptions, full synonymy (including designations of lectotypes and neotypes) and nomenclatural notes. Illustrations, distribution maps and preliminary conservation assessments are provided for all species.

African Non-Spiny

## Introduction

**Figure 1.**
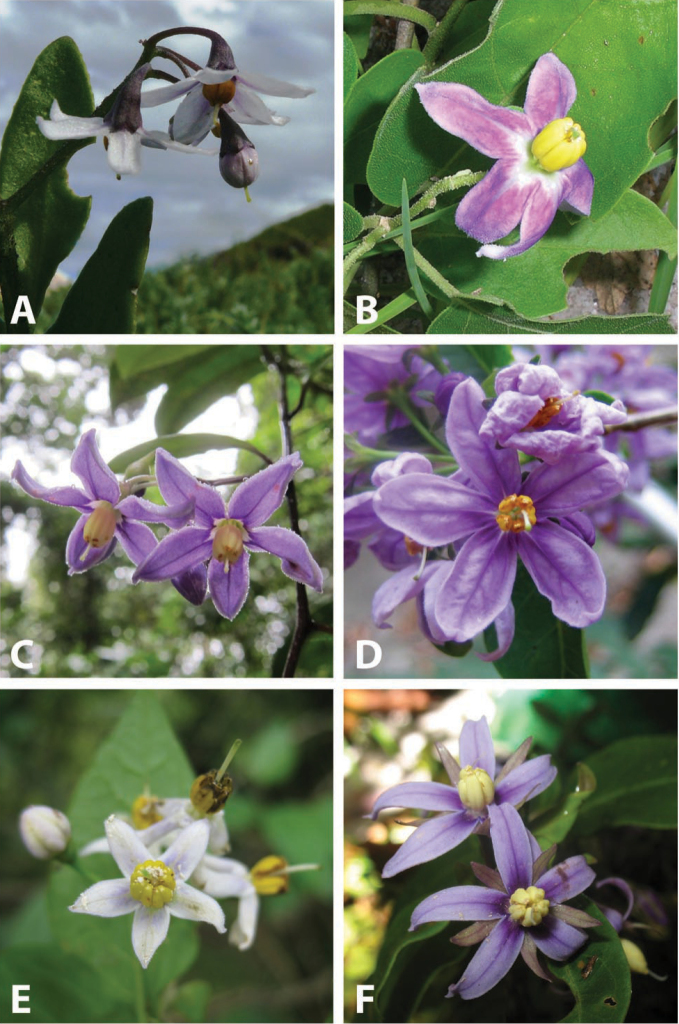
Flowers of species in the ANS clade. **A** *Solanum africanum* Mill. (South Africa, *Rebelo s.n.* – no herbarium voucher) **B** *Solanum imamense* Dunal (Madagascar, *Rakotavao 5128*) **C** *Solanum madagascariense* Dunal (Madagascar, *Vorontsova et al. 498*) **D** *Solanum sambiranense* D’Arcy & Rakot. (Madagascar, *Randrianasolo 580*) **E** *Solanum terminale* Forssk. (Kenya, *Vorontsova et al. 93*) **F** *Solanum truncicola* Bitter (Madagascar, *Antilahimena et al. 7846*). Photo credits: **A** Tony Rebelo; **B** Charles Rakotavao; **C, E** Maria Vorontsova; **D** Richard Randrianasolo; **E** Patrice Antilahimena.

*Solanum* L. is one of the ten most species-rich genera of flowering plants (Frodin 2004) and has approximately 1400 species occurring on all temperate and tropical continents. Species of *Solanum* have usually 5-merous flowers with fused sepals and petals, stellate to pentagonal corollas, stamens with short filaments, and anthers opening by terminal pores (Fig. 1). The highest diversity of both groups and species is in tropical South America, concentrated in a circle around the Amazon Basin (see Knapp 2002), but significant diversity occurs in various parts of the Old World. *Solanum* was one of Linneaus’s (1753) larger genera, with 23 species mostly described from European or African material. The last time *Solanum* was monographed in its entirety was in De Candolle’s *Prodromus* (Dunal 1852), which included 901 species (with an additional 19 recorded as incompletely known by him at the time). Until the 21^st^ century, the taxonomy of *Solanum* was largely limited to rearrangements of infrageneric taxa, species-level treatments of smaller groups within the genus, and floristic works. The large size of *Solanum* and its poorly understood infrageneric structure has meant that *Solanum* taxonomy has proceeded in a piecemeal fashion until relatively recently and the genus has acquired a reputation of being intractable. A project funded by the United States National Science Foundation’s Planetary Biodiversity Inventory (PBI) program begun in 2004 has sought to accelerate species-level taxonomic work across the genus and has resulted in a series of monographic and phylogenetic treatments from both Old and New Worlds (e.g., Tepe and Bohs 2011; Stern et al. 2013; Knapp 2013; Clark et al. 2015; Wahlert et al. 2015; Aubriot et al. 2016; Vorontsova and Knapp 2016). An electronic monographic treatment of the entire genus is begin made available online in the web resource Solanaceae Source (http://www.solanaceaesource.org). This treatment is part of that collaborative effort.

## Taxonomy of the African Non-Spiny (ANS) Clade

One of the principal divisions in *Solanum* is that between spiny (technically prickly) and non-spiny species. Early authors distinguished all spiny solanums as members of subgenus *Leptostemonum* Bitter (Dunal 1852; Bitter 1919) due to their tapered anthers, prickles, and pubescence of stellate trichomes. Dunal (1852) classified all non-spiny taxa as the group “Pachystemonum” distinguished by their lack of prickles and ellipsoid anthers. Phylogenetic studies using DNA sequences confirmed the monophyly of the spiny solanums with stellate pubescence (subgenus *Leptostemonum* or the Leptostemonum clade), but the non-spiny taxa did not form a monophyletic group (Bohs 2005; Weese and Bohs 2007; Särkinen et al. 2013). Non-spiny solanums can be divided into 11 monophyletic groups, of which the Geminata clade (Knapp 2002, 2008) is the largest. All of the non-spiny clades with the exception of the Normania clade (3 species from the Mediterranean and Macaronesia; Bohs and Olmstead 2001), the Archaeosolanum clade (13 species from Australia, New Zealand and New Guinea; Symon 1994) and the African Non-Spiny (ANS) clade (14 species treated here) are from the New World. A detailed history of *Solanum* classification can be found in Knapp (2013) and Vorontosova and Knapp (2016).

The classification of members of the ANS clade has been confused since their first description, and until the early part of the 20^th^ century these species were never recognised as being related to one another (see below). Like all biodiversity studies in Africa, early discovery and description of African non-spiny solanums were defined by the routes of early explorers, and then by delimitation of colonial territories. Linnaeus (1753) treated two of the species of the ANS clade in *Species Plantarum*. He described *Solanum guineense* L. with the locality as only “Guinea” (meaning Africa in general); he also included the species we here recognise as *Solanum africanum* as an un-named variety of the European *Solanum dulcamara* L. with a reference to Dillenius’ plate in *Hortus Elthamensis* (Dillenius 1732). Contemporary authors also described these two taxa, often based on more specifically located material. Philip Miller (1768) recognised the plant depicted in *Hortus Elthamensis* as distinct from the European *Solanum dulcamara*, at around the same time Carl Peter Thunberg, one of Linnaeus’s apostles (Blunt 1971) described *Solanum quadrangulare* Thunb. based on his own collections from the Cape region of South Africa. Another one of Linneaus’ disciples, Per Forsskål, accompanied a Danish expedition to Arabia and collected in both Egypt and the Arabian Peninsula (in what is now Yemen). He died in 1763 while in the field, but his botanical work was published posthumously (Forsskål 1775). He described *Solanum terminale* Forssk. from collections made in the mountains of Yemen.

Michel-Félix Dunal only knew of the South African species in his early works on *Solanum* taxonomy (Dunal 1813, 1816). In his synopsis of the genus (Dunal 1816) he treated *Solanum africanum* (as *Solanum quadrangulare*) as a member of his group “Maurella” together with species now recognised as members of the Morelloid clade (sensu Särkinen et al. 2015) such as *Solanum nigrum* L. *Solanum guineense* (as *Solanum aggregatum* Jacq.) was treated as a member of an un-named group that also contained members of the Geminata clade (*Solanum conocarpum* A.Rich., *Solanum havanense* Jacq.) as well as other species characterized by having single-flowered inflorescences. More species were known to Dunal by the time he produced his worldwide treatment of *Solanum* in the *Prodromus* (Dunal 1852). In that work names we now consider synonyms were scattered in several of his groups, depending on the completeness of Dunal’s knowledge of their morphology. Names we recognise as belonging to species of the ANS clade are classified in his subsection *Dulcamara* Dunal (*Solanum bifurcum* A.Rich., *Solanum quadrangulare*, *Solanum imamense* Dunal) characterised by having terminal inflorescences that later became lateral, in his subsection *Micranthes* (*Solanum aggerum* Dunal, *Solanum exasperatum* Dunal, *Solanum geniculatum* Dunal, *Solanum madagascariense* Dunal, *Solanum terminale*) characterised by a shubby habit and lateral inflorescences, and in his subsection *Pseudolycianthes* (*Solanum aggregatum* Jacq., *Solanum dasypus* Dunal, *Solanum monticolum* Dunal) characterised by single-flowered or subumbellate inflorescencces and equal anthers. Taxa that we here recognise as synonyms of *Solanum terminale* were placed in several different groups, and all of the names used by Dunal (1852) were recognised as distinct and accepted taxa (e.g., the synonyms of *Solanum guineense*). This confusion over the affinities of these species was in part due to incomplete knowledge of their growth form and their morphological similarity to the vining species now recognised as members of the Dulcamaroid clade (Knapp 2013).

The latter half of the 19^th^ century saw the expansion of European colonial occupation of Africa with concomitant exploration and species description, particularly in eastern Africa (e.g., Dammer 1906; Wright 1894, 1897). The German botanist Georg Bitter treated all African solanums in his monumental *Solana Africana* (Bitter 1913, 1917, 1921, 1922, 1923). He largely followed Dunal’s (1852) major divisions into spiny (Leptostemonum) and non-spiny (Pachystemonum) solanums, but attempted to better understand divisions within them (see Table 1). Bitter (1917) erected a new subgenus *Lyciosolanum* Bitter to accommodate the morphologically anomalous *Solanum guineense* (as *Solanum aggregatum*). He treated the rest of the African species as members of his subgenus *Eusolanum* Bitter. He was the first to bring the members of the ANS clade together as a group, and he treated them in a series of newly recognised sections (Table 1). Section *Quadrangulare* Bitter contained only *Solanum africanum* (as *Solanum quadrangulare*), section *Afrosolanum* Bitter was the largest and contained many names we here recognise as synonyms of the widespread and variable *Solanum terminale*, the Comoros endemic *Solanum macrothyrsum* Dammer and, with reservations, *Solanum runsoriense* C.H.Wright from the east African highlands of which he had seen no material. Species from Madagascar were treated in two sections *Lemurisolanum* Bitter (*Solanum humblotii* Dammer, *Solanum imamense* and *Solanum truncicola* Bitter) and *Macronesiotes* Bitter (*Solanum apocynifolium* Baker, *Solanum madagascariense*, *S nitens* Baker, here all considered synonyms of *Solanum madagascariense*), differentiated by their inflorescence size and position. Bitter (1917) erected section *Benderianum* Bitter for the heterostylous *Solanum benderianum* C.H.Wright (here recognised as a synonym of *Solanum runsoriense*), which he felt was only distantly related to the members of his section *Afrosolanum*. Bitter did not suggest relationships of any of these sections with those from the New World.

**Table 1.** Classification and recognition of currently recognised members of the African Non-Spiny (ANS) clade in previous systems as compared to the taxonomic delimitations recognised here.

Species	Section in Bitter (1917)	Section in D’Arcy and Rakatozafy (1994)
*Solanum africanum* Mill.	Quadrangulare	--
*Solanum betroka* D’Arcy & Rakot.	--	Not assigned
*Solanum guineense* L.	Lyciosolanum	--
*Solanum humblotii* Dammer	Macronesiotes	Macronesiotes
*Solanum imamense* Dunal	Macronesiotes	Macronesiotes
*Solanum ivohibe* D’Arcy & Rakot.	--	Not assigned
*Solanum macrothyrsum* Dammer	Afrosolanum	Lemurisolanum
*Solanum madagascariense* Dunal	Lemurisolanum	Lemurisolanum
*Solanum myrsinoides* D’Arcy & Rakot.	--	Lemurisolanum
*Solanum runsoriense* C.H.Wright	Benderianum (as *Solanum benderianum*); incertae sedis but as next to Afrosolanum (*Solanum runsoriense*)	--
*Solanum sambiranense* D’Arcy & Rakot.	--	Macronesiotes
*Solanum terminale* Forssk.	Afrosolanum	Not assigned to section
*Solanum trichopetiolatum* D’Arcy & Rakot.	--	Lemurisolanum
*Solanum truncicola* Bitter	Macronesiotes	Macronesiotes (as *humblotii*)

Bitter’s (1917) classification of members of the ANS has formed the basis for their treatment in subsequent generic classifications. Seithe (1962) classified *Solanum* using hair types and defined a group “chorus subgenerum *Solanum*” as those species with simple or branched trichomes; within that taxon she followed Bitter (1917) in segregating subgenus *Lyciosolanum* from subgenus *Solanum*, and in maintaining all of Bitter’s sections. She placed these in a group of vining plants with sections *Dulcamara* (Dunal) Bitter (Dulcamaroid clade, Knapp 2013), *Jasminosolanum* Seithe (Dulcamaroid clade, Knapp 2013), *Aculeigerum* Seithe (Wendlandii+Allophyllum group, see Clark et al. 2015) and *Herpystichum* Bitter (Potato clade, see Tepe and Bohs 2011). Danert (1970) also suggested a close relationship between sections *Dulcamara*, *Jasminosolanum* and *Aculeigerum* (equivalent in part to the Wendlandii/Allophyllum clade of Bohs 2005) and members of the ANS clade based on a shared vining habit. D’Arcy (1972) included all of the African vining species in subgenus *Solanum* as Bitter’s original sections (sensu Seithe 1962). Bitter’s (1917) sectional delimitations have been followed in recent floristic works (e.g., D’Arcy and Rakotozafy 1994; Edmonds 2012; see Table 1). D’Arcy (1992) suggested that section *Lemurisolanum* was relictual on Madagascar and derived from unspecified New World ancestors, and that section *Macronesiotes* was more recently derived from different South American taxa such as *Solanum uncinellum* Lindl. (as *Solanum pensile* Sendtn.; Dulcamaroid clade, see Knapp 2013).

Phylogenetic studies using DNA sequence data have shown that the African non-spiny taxa form a monophyletic group (Bohs 2005) that is one of the early branching lineages in *Solanum* (Särkinen et al. 2013); with only 3 taxa sampled, however, relationships of the group to other *Solanum* clades should be considered preliminary. Bohs (2005) was the first to show this grouping and her analysis included only species from South Africa (*Solanum africanum*, *Solanum guineense*). Särkinen et al. (2013) also included *Solanum terminale* and recovered a strongly supported clade composed of these species, plus the members of the Archaeosolanum and Normania clades (sensu Bohs 2005) that was sister to the Dulcamaroid+Morelloid clade (see fig. 1B in Särkinen et al. 2013). No Malagasy species of the group have yet been included in wider analyses, and Bohs (2005) followed D’Arcy (1992) in suggesting their affinities might lie with the New World vining species of the Dulcamaroid clade. Preliminary sequencing results using a combination of plastid and nuclear genes from three Malagasy species suggest they, in line with morphology, are members of the ANS clade (X. Aubriot, pers. comm.).

### Morphology

**Figure 2.**
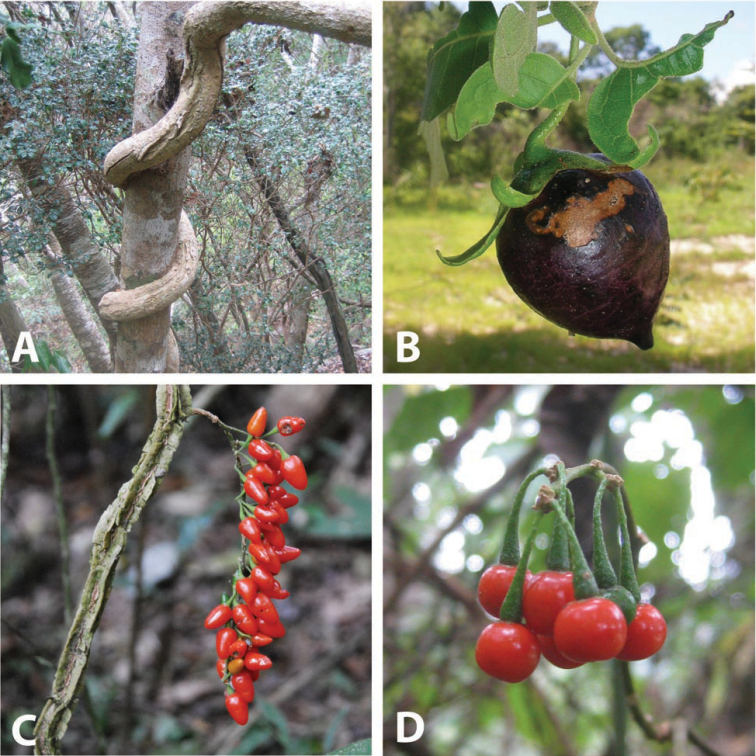
Habit and fruits of species of the ANS clade. **A** Habit of *Solanum sambiranense* D’Arcy & Rakot. (Madagascar, *Randrianasolo 580*) **B** Fruit of *Solanum imamense* Dunal (Madagascar, *Rakotavao 5128*) **C** Fruit of *Solanum terminale* Forssk., pointed form (Angola, *Goyder et al. 7749*) **D** Fruit of *Solanum terminale* Forssk., globose form (Tanzania, *Tepe et al. 2783*). Photo credits: **A** Richard Randrianasolo; **B** Charles Rakotavao; **C** David Goyder; **D** Maria Vorontsova.

***Habit and stems*.** Members of the ANS clade are all woody plants and to some extent climbers, even though some species (e.g., *Solanum guineense*, *Solanum terminale*) can be shrubby in some habitats (see discussion of *Solanum terminale*). Some species are large canopy lianas (e.g., *Solanum madagascariense*, *Solanum sambiranense* D’Arcy & Rakot., see Fig. 2A) while others are found scrambling though vegetation (e.g., *Solanum africanum*). *Solanum truncicola* and *Solanum myrsinoides* D’Arcy & Rakot. have both been recorded growing as epiphytes. Labels rarely record the height or length of stems of these plants, so the sizes are usually not known in detail, as is the case for other vines in *Solanum* (e.g., the Dulcamaroid clade, see Knapp 2013). Stems of *Solanum terminale* have recorded as large as 10 cm in diameter at the base, and *Solanum sambiranense* has been collected as a massive canopy vine (see Fig. 2A). Larger stems of *Solanum terminale* have thick, corky bark that dries a whitish grey colour in herbarium specimens. Dammer (1906), in his description of *Solanum suberosum* Dammer (a synonym of *Solanum terminale*) from Cameroon, suggested this was an adaptation to very wet environments; corky bark, however, is found on older stems of *Solanum terminale* in both wet and dry forests (e.g., *Knapp 9811* from Uganda).

Plants of species of the ANS clade can flower when quite small or before they reach the canopy. This has led to these smaller morphs being described as distinct species (e.g., *Solanum nakurense* C.H.Wright of savannah regions in east Africa). This variation in habit is also common in the vining species of the Dulcamaroid clade (e.g., *Solanum dulcamara* in Europe and North America) and has presented difficulties for taxonomists working entirely from herbarium specimens in the past.

In the ANS clade sympodial units are almost always plurifoliate with many (and a variable number) of leaves between each inflorescence in contrast to other groups such as the tomatoes (Peralta et al. 2008) or the Geminata clade (Knapp 2008) in which sympodial units are composed of 3 or fewer leaves. Plurifoliate sympodial units have the leaves arranged in a spiral fashion along the stems, and the leaves in members of the ANS clade are never geminate (paired, as seen in the Geminata clade, Knapp 2008 or in many spiny solanums, see Vorontsova and Knapp 2016). Most members of the group have monochasial branching, with a single axillary branch arising from below the inflorescence, giving the stems a zig-zag appearance (Danert 1958).

***Leaves*.** All members of the ANS clade have simple leaves when the plants are reproductive. Leaves in some species (*Solanum africanum* and *Solanum betroka* D’Arcy & Rakot.) are shallowly lobed, but the lobes are never pronounced, and the sinuses never deeper than ¼ of the leaf width. As is the case in species of the Dulcamaroid clade (Knapp 2013) lobed and unlobed leaves are commonly found on the same stem. We have seen no specimens where juvenile leaves are clearly indicated, but it is possible that like species of the Dulcamaroid clade, these taxa have lobed leaves on pre-reproductive stems. Non-flowering stems are rarely collected; field observations on these species are a priority to assess this character across the group.

Variation in leaf pubescence is common in members of the group; trichome types are treated below. Several species in the ANS clade have prominent “tufts” of tangled trichomes in the axils of the main veins on the leaf undersurfaces (e.g., *Solanum ivohibe* D’Arcy & Rakot., *Solanum macrothyrsum*, *Solanum sambiranense*). In some species such as *Solanum imamense*, the overall pubescence of the leaf undersides is denser near the vein axils, while in others such as *Solanum macrothyrsum*, trichome tufts are the only pubescence on the entire plant. These concentrations of trichomes have been shown to house small arthropods such as mites, and are often subtended by small pouches caused by invaginations of leaf tissue and referred to as domatia (O’Dowd and Willson 1991). Domatia act as refuges for beneficial mites against predators (Romero and Benson 2005) resulting in fewer pathogen attacks and reduced herbivory, but also have been shown to harbour herbivorous mites. Although tufts of trichomes have been interpreted as domatia in *Solanum* (Knapp 2002, 2008), no studies have specifically addressed their function. We have not seen mites or other small arthropods in these concentrations of trichomes in species of the ANS clade.

Unlike other some other groups of vining solanums (e.g., Dulcamaroid clade, Knapp 2013), members of the ANS clade do not have obviously twining petioles that are used for climbing. Petioles in many species appear flexuous (e.g., *Solanum myrsinoides*, *Solanum terminale*), but only in *Solanum madagascariense* (e.g., *Humbert 31597* from Marojejy) are the petioles clearly used as support. This may be because many of these species are canopy lianas and the parts collected are hanging and no longer involved in active climbing.

***Pubescence*.** Trichome types and density can be useful for species recognition in *Solanum*. Previous classifications of the genus (Seithe 1962) have been constructed using primarily trichome types, with groups defined by possession of stellate, branched or simple trichomes. Later research (e.g., Knapp 2002; Bohs 2005) has shown that trichome types do not define monophyletic groups in *Solanum*, but that they can be useful for identification at the species level. Like all other species of non-spiny solanums members of the ANS clade have simple (unbranched) or branched uniseriate trichomes or lack trichomes altogether (e.g., Edmonds 1982; Särkinen et al. 2015). Branched trichomes in these species are dendritic (e.g., *Solanum imamense*, *Solanum terminale*), arachnoid (e.g., some populations of *Solanum madagascariense*) or verging on what is usually termed echinoid (Roe 1971; Knapp 2013: 12), with many short, congested branches (e.g., *Solanum ivohibe*). Plants of most species have at least some pubescence on the new growth, but all parts of *Solanum myrsinoides* are completely glabrous. *Solanum africanum* and *Solanum guineense* have variable pubescence, with plants ranging from completely glabrous to densely and evenly pubescent, but only have simple uniseriate trichomes. Malagasy species with only simple trichomes are *Solanum trichopetiolatum* D’Arcy & Rakot. and *Solanum truncicola*. The trichomes of *Solanum trichopetiolatum* are unusual in the group in being very long and concentrated on the petioles and stems and not being markedly denser on new growth. Dendritic trichomes are found in *Solanum humblotii*, *Solanum imamense*, *Solanum ivohibe*, and *Solanum sambiranense. Solanum madagascariense*, *Solanum runsoriense* and *Solanum terminale* all are polymorphic for simple and dendritic trichomes, and in these species trichome density also varies a great deal, especially in *Solanum terminale*. Variation in trichome density and type is common in many groups of non-spiny solanums (e.g., Geminata clade, Knapp 2002; Dulcamaroid clade, Knapp 2013) and may in part be a response to water and light availability. In some species of the ANS clade, trichome variation has a geographic component (e.g., see discussion of *Solanum runsoriense*).

***Inflorescences*.** As with all species of *Solanum*, the inflorescence in members of the ANS clade is developmentally terminal, and is often later overtopped by the leading axillary shoot making it appear lateral. The basic inflorescence, as in all other species of *Solanum*, is a scorpoid cyme that is branched or unbranched. Most members of the ANS clade have branched inflorescences with a distinct peduncle (see Fig. 1 and individual species illustrations). Some species have large open inflorescences (e.g., *Solanum macrothyrsum*, *Solanum runsoriense*) with many branches, while others are merely furcate (branched once to form a ‘Y’ shape, e.g., *Solanum trichopetiolatum*). *Solanum humblotii* and *Solanum truncicola* from Madagsacar have unbranched inflorescences of very few (sometimes only one) flowers that arise directly from the stem. The inflorescences of *Solanum guineense* are few-flowered and appear as fascicles at the ends of short shoots. *Solanum terminale* has branched inflorescences, but in many populations from western Africa the inflorescence branches are extremely short so that the inflorescence appears spicate. In species with branched inflorescences the size of the inflorescence can be very variable, and may to some extent depend upon plant or inflorescence age. In solanums the inflorescence expands from the tip with each apical meristem producing multiple flowers in a proliferating manner (Lippmann et al. 2008).

Similarly to other vining solanums the pedicels in both flower and fruit are spreading to somewhat pendent, often oriented in different directions on the same inflorescence. Fruiting pedicels are more commonly deflexed from the weight of the berry, especially in species with larger fruits (e.g., *Solanum imamense*, see Fig. 2B). The abscission zone at the pedicel base in species of the ANS clade is somewhat raised, so that when pedicels fall, a small peg is left on the inflorescence rachis. This extension of rachis tissue is never a small sleeve enveloping the base of the pedicel proper like that found in members of the Dulcamaroid clade (see for example fig. 4C in Knapp 2013), but is usually distinct and can be to 0.5 mm long (e.g., *Solanum myrsinoides*) or the pedicel scar can be flush with the rhachis (*Solanum runsoriense*).

Pedicel scars indicate the spacing of flowers on inflorescences and have been useful in differentiating species in other groups of solanums (e.g., the Geminata clade, Knapp 2008). Pedicels are generally more tightly spaced near the distal tips of the inflorescence and scars along more basal portions of the inflorescence are more widely spaced. *Solanum terminale* always has flowers borne in tightly packed clusters at the end of inflorescence branches (these of varying length, see above and *Solanum terminale* description). This can be a useful feature for distinguishing specimens without flowers or fruit from the vegetatively similar *Solanum runsoriense*, whose pedicel scars not clustered.

***Calyces*.** The calyx in members of the ANS clade is synsepalous and 5-merous with the tube and lobes more or less equal in size. The shape of the calyx lobes varies considerably and is a useful character for species identification. Lobes vary from mere undulations of the calyx rim (e.g., *Solanum myrsinoides*) to deltate (many species) or lanceolate (e.g. *Solanum truncicola*). Calyx lobe apices are usually acute to acuminate, but are occasionally rounded (e.g., *Solanum africanum*) or cuspidate (e.g., *Solanum macrothyrsum*). Pubescence of the calyx tube and lobes usually parallels that of the pedicels and inflorescence rachis, but is generally sparser. Most species have green calyces, but label notes on collections of *Solanum truncicola* usually mention the purple coloration of the calyx lobes (see Fig. 1F).

***Corollas*.** In common with all species of *Solanum*, members of the ANS clade have 5-merous sympetalous corollas that are variously stellate. Colour is either white or varying shades of purple; many species have populations of both color forms. Polymorphism in corolla colour is common in many groups of *Solanum* and from our observations in the field occasionally appears to be related to light intensity. At the base of the corolla tube is often a ring or irregular area of differently colored tissue often referred to as the eye; in some groups of species (e.g., Dulcamaroid clade, see Knapp 2013; Morelloid clade, see Särkinen et al. 2015) this is consistent within a species, but in the ANS clade the eye is usually only a slightly paler coloured portion of the base of the corolla lobes (e.g., *Solanum imamense*, Fig. 1B). This colour difference is not as marked as in species such as *Solanum dulcamara* or *Solanum umbelliferum* Eschsch. (both members of the Dulcamaroid clade, see Knapp 2013).

Corollas of members of the ANS clade are stellate and vary in lobe length and width. *Solanum terminale* has deeply stellate corollas, where the lobes are more than ¾ of the total length of the corolla. Corolla diameter varies from 1 cm (*Solanum ivohibe*, some specimens of *Solanum terminale*) to 5 cm (*Solanum guineense*); most species have corollas that range from 1.5-2.5 cm in diameter. Some species (e.g., *Solanum guineense*, *Solanum terminale*) appear to have corollas that expand with age, as is seen in other Solanaceae (Dean 2001; Knapp 2013). Most populations of *Solanum terminale* have narrow, ligulate corolla lobes. *Solanum runsoriense* has less deeply stellate corollas, and the lobes are more broadly deltate; *Solanum guineense* has lobes much shorter than the tube. In many solanums, petal orientation is variable through the life of a flower; this is the case in the species of the ANS clade. *Solanum guineense* is unusual in *Solanum* in having corolla lobes that are not spreading or reflexed for at last the early part of the flowering period; this in part accounts for its early description as a species of *Atropa* L. (see discussion of *Solanum guineense*). In other species the corolla lobes are spreading to sharply reflexed, in some cases this appears to depend on flower age (e.g., *Solanum terminale*). The orientation of corolla lobes is very difficult to determine from herbarium specimens. Abaxial corolla surfaces in most solanums are papillate to pubescent, usually on corolla surfaces exposed in bud. Most species of the ANS clade are sparsely pubescent with the trichomes denser along the margins of the corolla lobes and at the tips. *Solanum terminale* is unusual in having densely papillate abaxial corolla surfaces that give the flowers a greyish white scurfy cast in herbarium specimens. *Solanum uncinellum* of the Dulcamaroid clade has similarly dense mealy-looking corolla pubescence on narrow corolla lobes; this led to the suggestion of a relationship between these New and Old World taxa (D’Arcy 1992; Knapp 2013).

***Androecium*.** The stamens of all species in the ANS clade are equal in size and length. The filament tube and filaments are glabrous and very short; in *Solanum africanum* the filament tube is almost absent. Anthers of members of the ANS clade conform to the poricidal morphology of all other species of *Solanum* (see Knapp 2001, 2002). In most non-spiny *Solanum* species the pore usually “unzips” during anther dehiscence to form a tear-drop shaped slit, from which pollen is shed during vibratile pollination (Buchmann et al. 1977; Buchmann 1986). In the ANS clade, all of the continental African members possess this typical non-spiny solanum morphology (see Fig. 1A, E), with terminal anthers elongating to form slits as flowers age. The Malagasy species of the ANS clade (including *Solanum macrothyrsum* of Mayotte), however, have pores that do not elongate at all (see Fig. 1B, C, D, F). This is unusual in non-spiny solanums, although is also found in some Antillean members of the Geminata clade such as *Solanum havanense* and *Solanum troyanum* Urb. (Knapp 2008). Anthers in the ANS group generally ellipsoid and loosely connivent, while those of some western African forest populations of *Solanum terminale* are tightly connivent, and such that the pores appear to act as a single opening to a visiting bee. It is not known how the anthers are held together but they are perhaps held together physically with a sticky “glue” as are the anthers of *Solanum dulcamara* (Glover et al. 2004) or by lateral papillae similar to those seen in the tomatoes (Peralta et al. 2008). The preponderance of this anther morphology in *Solanum terminale* populations from wet forest habitats may indicate a role for anther adhesion as a way to protect pollen in these very wet environments.

The anthers of several species of the ANS clade are occasionally papillate on the dorsal anther surface (e.g., *Solanum myrsinoides*) and appear “pubescent” in dry specimens. D’Arcy (1992) suggested these papillae either functioned to reduce pollen thieving by restricting access to anther pores or were “holdfasts” for buzzing bees, the legitimate pollinators of these species. He also noted that they were more prevalent at higher elevations and that members of “Lemurisolanum” (i.e., *Solanum madgascariense*, *Solanum ivohibe*, *Solanum myrsinoides*, *Solanum trichopetiolatum*) were polymorphic for their presence in specimens of the same species from similar sites. We have not found a relationship between elevation and papillate anthers, and the character is polymorphic in several species (e.g., *Solanum madagascariense*, *Solanum terminale*). Glover et al. (2004) induced the formation of papillae (trichomes) in *Solanum dulcamara* by overexpression of MIXTA and MIXTA-like genes; these transcription factors are responsible for the conical cells of petals that function in both pollinator attraction (Noda et al. 1994) and grip (e.g., Alcorn et al. 2012). Current work (B.J. Glover and G. Davis, pers. comm.) investigating the distribution of genetic controls for these anther papillae across *Solanum* is showing their presence is scattered across the genus, not limited to the Malagasy members of the ANS clade as D’Arcy (1992) supposed.

***Gynoecium*.** The gynoecium is typically bicarpellate; the carpels are fused in a superior ovary with axillary placentation. The ovary is usually conical to globose or slightly ellipsoid and usually glabrous, although we have seen a few specimens of *Solanum terminale* with minutely hispidulous ovaries. The flowers lack nectaries, as do all other species of *Solanum*. The style is straight or more often slightly curved, glabrous or variously pubescent, and is exserted beyond the anthers in all but *Solanum runsoriense* (see below). The stigma is capitate (e.g., *Solanum africanum*, *Solanum sambiranense*) to clavate (e.g., *Solanum truncicola*) and is sometimes distinctly bilobed (e.g., *Solanum ivohibe*). The ovules are anatropous and non-arillate.

Bitter (1917) characterised his monospecific section *Benderianum* (including only *Solanum benderianum* that we here recognise as *Solanum runsoriense*) by its large seeds and heterostylous flowers, although he stated that this latter characteristic needed further investigation since he had seen few specimens. All specimens we have seen of *Solanum runsoriense* have either had all long- or all short-styled flowers, perhaps indicating a monoecious or dioecious breeding system in this species. Pollen morphology has not yet been investigated to determine if it is inaperturate in long-styled flowers, as occurs in other dioecious solanums (e.g., Anderson and Symon 1989; Knapp et al. 1998). None of the other species of the ANS clade have heterostylous flowers, although some specimens of *Solanum terminale* have the style almost included within the anther cone.

***Fruits*.** As with all species of *Solanum*, the fruit is a bicarpellate berry. Fruits of members of the ANS clade are usually brightly colored and either juicy (e.g., *Solanum guineense*, *Solanum terminale*) or spongy to possibly somewhat woody (e.g., *Solanum imamense*, *Solanum myrsinoides*). Fruit colour varies from bright red (*Solanum terminale*) to orange (*Solanum guineense*) to dark purple (*Solanum africanum*, *Solanum imamense*) or black (*Solanum madagascariense*); immature berries are usually described on labels as green (Fig. 2). Mature fruits are not known from a number of these species (*Solanum betroka*, *Solanum humblotii*, *Solanum myrsinioides*, *Solanum sambiranense*, *Solanum truncicola*), and we have seen no fruiting collections of *Solanum ivohibe* and *Solanum macrothyrsum*.

Many species have berries that are pointed at the tip or variously elongate. *Solanum imamense* and *Solanum myrsinoides* have what appear to be quite solid berries with sharp apical points (see Fig. 2B); D’Arcy and Rakotozafy (1994) characterised those of *Solanum myrsinoides* as “woody”. A sketch on the type specimen of *Solanum imamense* at G (G00144901) shows a berry more than twice as long as wide (http://www.ville-ge.ch/musinfo/bd/cjb/chg/adetail.php?id=143074&base=img&lang=en). Other solanum berries described as woody or bony in texture (e.g., *Solanum dennekense* Dammer of eastern Africa) we have seen are not woody when fresh, the hardness is a result of drying (Vorontsova and Knapp 2016). *Solanum terminale* has a wide range of fruit shapes from globose to elongate (see Fig. 2C, D). Bitter (1922: 307) cited label data from *Mildbraed 5576* from Cameroon (identified by him as *Solanum suberosum*, a specimen presumably at B and now destroyed) as stating “Früchte leuchtend mennigrot, stark glänzend, wie kleine ‘Pfefferschotten’” (Fruits bright vermilion-red, highly lustrous, like little Scotch peppers).

In general brightly coloured berries in *Solanum* have thin pericarp; this is the case for the species of continental Africa in the ANS clade (*Solanum africanum*, *Solanum guineense*, *Solanum runsoriense* and *Solanum terminale*). In Madagascar, however, some species have the typical thin pericarp (e.g., *Solanum madagascariense*) while others have what appears to be leathery or hard pericarp (e.g., *Solanum imamense*). D’Arcy (1992) suggested the fusiform fruit shape and thick fruit walls of the Madagascar species had evolved in response to the paucity of fruit-eating birds and preponderance of fruiting-eating mammals such as lemurs on the island, but this has not been tested. We have found no field records of animals consuming the fruits of these taxa on Madagascar.

***Seeds*.** Seed morphology has been suggested to be a useful character for species-level taxonomy in *Solanum* (Souèges 1907; Lester and Durands 1984) and has been used to define morphological groups in other clades (e.g., Geminata, see Knapp 2002). Seeds of members of the ANS clade are oval, obovate or kidney-shaped in outline and usually flattened laterally, but the seeds of *Solanum terminale* are ovoid-reniform and not markedly flattened. The cells of the outer epidermal layer in some species (e.g., *Solanum imamense*, *Solanum terminale*) develop radial wall thickenings that form as “hair-like outgrowths” or “pseudohairs” in mature seeds (Souèges 1907; Lester and Durrands 1984; Lester 1991). These hair-like outgrowths often greatly enlarge the outer layer of the integument and the seed coat appears pubescent; seed measurements here include these projections. In seeds from mature fruits the pseudohairs are translucent, connate or fused laterally to each other and tightly adpressed to the epidermis giving a silky appearance to the seed surface, or if long and distinct produce a hairy or shaggy looking seed surface. The mature seeds of *Solanum terminale* have abundant pseudohairs and those of *Solanum imamense* moderately developed ones; none of the other species of the group have such markedly elongate lateral cell walls, although we have not seen mature seeds of all species. The testal cells form a reticulate or honeycomb pattern with cell outlines at the basal portions deeply sinuate and irregular (e.g., *Solanum guineense*, *Solanum trichopetiolatum*), pentagonal/rectangular (e.g., *Solanum africanum*, *Solanum runsoriense*) or somewhat intermediate (e.g., *Solanum madagascariense*).

Seed number per berry in the ANS clade is relatively small compared to many other groups of non-spiny solanums such as the Morelloid clade where fruits can have 50+ seeds (Sarkinen et al. 2015). Some species have fewer than 10 (e.g., *Solanum myrsinoides*, *Solanum trichopetiolatum*) while others (e.g., *Solanum imamense*, *Solanum madagascariense*, *Solanum runsoriense*) have up to 30 seeds per berry. Most species fall in the range of 10-20 seeds per berry. Seed size varies from 1.5 mm length and 1 mm width to 6 mm length and 4 mm width. Color varies from yellow or pale brown to dark brown. Seeds are not known from almost half of the species in the clade (*Solanum betroka*, *Solanum humblotii*, *Solanum ivohibe*, *Solanum macrothyrsum*, *Solanum sambiranense*, *Solanum truncicola*).

### Biology and natural history

***Habitats and distribution*.** The evolution of plant diversity in continental Africa has been the subject of much study, and spatial patterns of diversity have been used to define phytochoria or vegetation types (White 1983, 1993; Küper et al. 2006; Linder et al. 2005; Linder 2014). White’s (1983) original scheme of phytochoria was based on the small range sizes of most African species, and subsequent numerical analysis supported his broad regions, but with broad transitions from one vegetation type to the next (Linder et al. 2005). Some of the gaps identified by White (e.g., White 1979) are the result of collecting deficits, as is to be expected in the tropics in general. Linder (2014) explored the origins and affinities of these vegetation types and suggested that current African plant diversity was the result of sequential addition of floristic elements from different areas in both the northern and southern Hemispheres.

Vorontsova et al. (2013) showed that spiny solanums occurring in continental Africa clades were largely confined to particular vegetation types and they suggested that diversification had gone on mostly within these vegetation types, particulary in drier areas such as the “Somalia-Masai regional center of endemism” (sensu White 1983; corresponding to the northern one of Linder’s [2014] Arid floras). This concentration of both species richness and endemism in arid zones is not the case in the much older ANS clade. Of the four continental species in the ANS clade, two (*Solanum africanum* and *Solanum guineense*) are confined to the Cape region (Austro-temperate flora of Linder 2014); *Solanum runsoriense* is found in the central zone of the Tropic-montane flora (of Linder 2014; Afromontane flora of White 1981) and the widespread *Solanum terminale* occurs in all of the floristic regions identified by Linder (2014) in a wide variety of different forest types from savannah edges to lowland wet forest (see Table 2).

**Table 2.** Country distribution of members of the ANS clade.

Country	Species
Angola	*Solanum terminale*
Burundi	*Solanum terminale*
Cameroon	*Solanum terminale*
Central African Republic	*Solanum terminale*
Comoro Islands (incl. Mayotte)	*Solanum macrothrysum*, *Solanum terminale*
Côte d’Ivoire	*Solanum terminale*
Democratic Republic of the Congo	*Solanum runsoriense*, *Solanum terminale*
Equatorial Guinea	*Solanum terminale*
Eritrea	*Solanum terminale*
Ethiopia	*Solanum runsoriense*, *Solanum terminale*
Gabon	*Solanum terminale*
Ghana	*Solanum terminale*
Guinea	*Solanum terminale*
Guinea Bissau	*Solanum terminale*
Kenya	*Solanum runsoriense*, *Solanum terminale*
Liberia	*Solanum terminale*
Madagascar	*Solanum betroka*, *Solanum humblotii*, *Solanum imamense*, *Solanum ivohibe*, *Solanum madagascariense*, *Solanum myrsinoides*, *Solanum sambiranense*, *Solanum trichopetiolatum*, *Solanum truncicola*
Malawi	*Solanum terminale*
Mozambique	*Solanum terminale*
Nigeria	*Solanum terminale*
Republic of Congo	*Solanum terminale*
Rwanda	*Solanum terminale*
São Tome e Principe	*Solanum terminale*
Sierra Leone	*Solanum terminale*
South Africa	*Solanum africanum*, *Solanum guineense*, *Solanum terminale*
South Sudan	*Solanum terminale*
Tanzania	*Solanum runsoriense*, *Solanum terminale*
Togo	*Solanum terminale*
Uganda	*Solanum runsoriense*, *Solanum terminale*
Yemen	*Solanum terminale*
Zambia	*Solanum terminale*
Zimbabwe	*Solanum terminale*

The ANS clade is significantly older than the clade of spiny solanums occurring in Africa, it is one of the early-branching lineages in the genus (Särkinen et al. 2013), while the Old World clade of spiny solanums is derived within the monophyletic Leptostemonum clade. Linder (2014) suggested that the Austro-temperate flora was related to other floras of the southern hemisphere; the relationship between the ANS clade and the Archaeosolanum clade of Australia is consistent with this, but lack of sampling for the Malagasy species of the ANS clade limits our ability to further consider this hypothesis. The broad distribution in terms of both geographical extent and vegetation type of *Solanum terminale* coupled with its morphological variability (see description of *Solanum terminale*) may mean it is a taxon in the process of differentiation. The species is very plastic; in Kenya we have seen it growing in the forest as a liana and in adjacent savannah as a small erect shrub. This adaptability may be a contributory factor to its wide range.

Madagascar’s famously rich biodiversity is thought to be related to its sharply delimited contrasting ecotypes in close proximity to one another: wet east, arid south, cool High Plateau, warm and seasonal west, and vegetation similar to the Afromontane zone on its highest mountains. Distribution patterns of the majority of Madagascar’s plants are defined by these ecotypes (Callmander et al. 2011; Humbert 1955; Faramalala 1988, 1995; Moat and Smith 2007) and the ANS species are a typical example of these distributions. All of the Malagasy species are endemic and, like species of the continent, occupy very different vegetation types than those occupied by the endemic spiny solanums. Spiny solanums on Madgascar are mostly found in xeric environments (Vorontsova and Knapp 2016). Members of the ANS clade, in contrast, are found in forested regions, with the exception of *Solanum betroka* from southern Madagascar. Wet rainforests of the northeast and east are the richest in range-restricted endemics with *Solanum myrsinoides* and *Solanum trichopetiolatum* found in the north and around Marojejy, *Solanum humblotii* further south to Toamasina, *Solanum truncicola* further south still to Ivohibe, and *Solanum ivohibe* restricted to the area near Andringitra. The most common and the most broadly distributed species of the ANS clade is *Solanum madagascariense*, commonly collected across all wet forests of the island and sympatric with *Solanum myrsinoides*, *Solanum trichopetiolatum*, *Solanum humblotii*, *Solanum truncicola*, and *Solanum ivohibe*. The seasonally dry west part of the island is home to *Solanum sambiranense*, which is most similar and possibly related to to *Solanum betroka* in the arid south, and *Solanum imamense* which occurs over a broad area including the High Plateau, west, and regions bordering the arid south. *Solanum macrothysum* is endemic to Mayotte, where the only other ANS clade member recorded is *Solanum terminale* (see discussion under *Solanum terminale*).

***Pollination and breeding systems*.** Very little is known about pollination and breeding systems in the species of the ANS clade. On Madagascar flowers of native *Solanum* species are visited and buzzed by “several genera of anthophorid bees” (Apidae: Anthophorinae), species of the genus *Sphegocephala* (Apidae), species of *Thrinchostoma* de Saussure (Halictinae) and two other “genera of halictids” (Anders Nilsson pers. comm. in D’Arcy 1992). Unfortunately D’Arcy (1992) did not report the species of *Solanum* visited by these bees, but it is clear that both small and large bees buzz and presumably pollinate solanums in Madagascar.

The flowers of all species of the ANS clade except *Solanum runsoriense* are perfect; heterostyly in *Solanum runsoriense* has not been examined in the field (see above). Neither chromosome number nor compatability have been reported for any of these species; field studies of living plants in their environments are a priority for this group.

***Conservation status*.** Many species of plants endemic to Madagascar are threatened with extinction, and it is one of the global hotspots of conservation concern (e.g., Myers et al. 2000). We performed preliminary conservation assessments for all the species treated here (see Materials and Methods) and based on geographical criteria, only five of them are of conservation concern (see Table 3) using the EOO (extent of occurrence) as the principal criterion. The large size and canopy habit of many of these taxa means, however, that their population structure and local distribution is poorly known. This, coupled with the fragile nature of the wet forest habitats of Madagascar, may indicate that even those species assessed here as of least concern warrant further study. *Solanum terminale*, though the most widespread and therefore the least threatened of these species, is clearly morphologically variable and probably harbours much genetic diversity that may be vulnerable in local areas where forests are threatened.

**Table 3.** Preliminary conservation assessments of members of the African Non-Spiny Clade.

Species	Preliminary conservation assessment (IUCN 2014)
*Solanum africanum* Mill.	Least Concern (LC)
*Solanum betroka* D’Arcy & Rakot.	Least Concern (LC)
*Solanum guineense* L.	Least Concern (LC)
*Solanum humblotii* Dammer	Near Threatened (NT)
*Solanum imamense* Dunal	Least Concern (LC)
*Solanum ivohibe* D’Arcy & Rakot.	Endangered (EN, B1a, b iii)
*Solanum macrothyrsum* Dammer	Data Deficient (DD)
*Solanum madagascariense* Dunal	Least Concern (LC)
*Solanum myrsinoides* D’Arcy & Rakot.	Vulnerable (VU, B1a, B iii)
*Solanum runsoriense* C.H.Wright	Least Concern (LC)
*Solanum sambiranense* D’Arcy & Rakot.	Least Concern (LC)
*Solanum terminale* Forssk.	Least Concern (LC)
*Solanum trichopetiolatum* D’Arcy & Rakot.	Vulnerable (VU, B1a, B iii)
*Solanum truncicola* Bitter	Vulnerable (VU, B1a, B iii)

### Species concepts

Our goal for the treatment of the species of the ANS clade has been to provide circumscriptions for the members of this relatively poorly collected and morphologically variable clade, while clearly highlighting those taxa where further in-depth research would be useful. Delimitation of species here basically follows what is known as the “morphological cluster” species concept (Mallet 1995): i.e. “assemblages of individuals with morphological features in common and separate from other such assemblages by correlated morphological discontinuities in a number of features” (Davis and Heywood 1963). Biological (Mayr 1982), phylogenetic (Cracraft 1989) and the host of other finely defined species concepts (see Mallet 1995) are almost impossible to apply in practice and are therefore of little utility in a practical sense. It is important, however, to clearly state the criteria for the delimitation of species, rather than dogmatically follow particular ideological lines (see Luckow 1995; Davis 1997). Our decisions relied on clear morphological discontinuities to define the easily distinguished species. Specific characters used for recognition are detailed with each species description and in the key. Some potential reasons for variability and intergradation are recent divergence, hybridization and environmental influence on morphology. In this revision we have tried to emphasise similarities between populations instead of differences, which so often reflect incomplete collecting or local variation. We have not recognised subspecies or varieties, but have rather described and documented variation where present, rather than formalised such variability with a name which then encumbers the literature. We have been conservative in our approach, recognising as distinct entities those population systems (sets of specimens) that differ in several morphological characteristics. Minor differences in morphology, distribution, habitat, and ecology are important in some groups, where the common groundplan for the species is very similar. On the other hand, we have delimited *Solanum terminale* as an extremely widespread, polymorphic species, in marked contrast to previous treatments (e.g., Bitter 1917; Edmonds 2012). In *Solanum terminale* variation exists in certain characters, but the pattern of variation is such that no reliable units can be consistently extracted, nor is geography a completely reliable predictor of character states. Here variability within and between populations seems more important than the variations of the extremes other taxonomists have recognised as distinct. We describe this variation realising that others may wish to interpret it differently.

## Materials and methods

This monograph is based on examination of herbarium specimens supplemented with field observations in both continental Africa and Madagascar. We examined approximately 2,000 collections (just under 3,000 specimens) from the following herbaria (herbarium acronyms follow *Index Herbariorum*, found on-line at http://sciweb.nybg.org/science2/IndexHerbariorum.asp): B, BH, BM, BR, C, CAS, DS, DSM, DUKE, E, EA, ETH, F, FT, G, G-DC, GOET, JE, K, LE, LWI, M, MA, MO, NU, NY, OXF, P, PAL, S, SCA, TAN, U, US, W, WAG, WU, YA.

Measurements were made from dried herbarium material supplemented by measurements from living material. Colours of corollas, fruits, etc., are described from living material or from herbarium label data. Specimens with latitude and longitude data on the labels were mapped directly. Some species had few or no georeferenced collections; in these cases we retrospectively georeferenced the collections using available locality data. Maps were constructed with the points in the centers of degree squares in a 1° square grid. Conservation threat status was assessed following the IUCN Red List Categories and Criteria (IUCN 2014) using the GIS-based method of Moat (2007) as implemented in the online assessment tools in GeoCat (http://geocat.kew.org). The Extent of Occurrence (EOO) measures the range of the species, and the Area of Occupancy (AOO) represents the number of occupied points within that range based on the default grid size of 2 km^2^.

In order to assess the geographical components in variation in *Solanum terminale*, we measured three contiuously varying characters (bud length, bud width at the widest point and length of the lowermost inflorescence branch) and scored anther anther connation and fruit shape as binary states (free or fused; round or pointed); these are the characters used to distinguish segregate taxa by other authors (e.g., Edmonds 2012). To normalise bud shape we calculated a ratio of bud length/width. Measurements were done on 50 herbarium specimens highlighted in the dataset published on the Natural History Museum Data Portal (http://dx.doi.org/10.5519/0039445) and spanned the east-west distribution of *Solanum terminale*. Characters were measured using a stereo microscope and a captured with DinoCapture software (Dino-Lite, AnMo Electronics Ltd.).

Type specimens for *Solanum* from Africa are difficult to trace. Many taxa were described based on types housed in the Berlin herbarium (B), which was destroyed by bombing during the Second World War (see Vorontsova and Knapp 2010). In our searches of many potential repositories for original material we have been able to trace duplicates of at least some of these. In cases where we have found no duplicates for accepted names we have selected neotypes, but for synonyms we have cited the taxa in synonymy and indicated that duplicates have not been found rather than neotypifying these (often infraspecific) taxa. But in some cases where citation of specimens by the original authors is not specific enough to assign a lectotype (see McNeill 2014), we have neotypified synonyms where we could (e.g., *Solanum laurentii* Dammer). We have also made use of specimen images available via online databases: G (http://www.ville-ge.ch/cjb/bd.php), P (https://science.mnhn.fr/institution/mnhn/collection/p/item/search/form), W (http://herbarium.univie.ac.at/database/search.php), Z (http://www.herbarien.uzh.ch/index.html), the African Plants Initiative and Global Plants (http://plants.jstor.org). Georg Bitter described many taxa of *Solanum* in the course of his monumental work on African solanums and worked widely in Germany in the period between the two World Wars (Weber 1928), including, but not exclusively at Berlin. His protologues frequently include specific herbarium citations, but often do not. We have cited specimens as holotypes only when a single specimen with a single herbarium citatation is indicated in the protologue; we have not assumed his types are all in B. The German botanist Udo Dammer also described many African taxa in the early part of the 20^th^ century; none of his protologues cite specific herbaria. Where specific herbaria have not been cited in protologues we have followed McNeill (2014) and designated lectotypes rather than assuming holotypes exist. We cite page numbers for all previous lectotypifications.

Type specimens with sheet numbers are cited with the herbarium acronym followed by a dash and the sheet number (i.e., MO–1781232); barcodes are written as a continuous string (i.e., G00104280). We have cited geographically representative specimens for taxa where more than 100 collections are known. Identities of all numbered collections seen for this study are in Supporting Material (Index to Numbered Collections; Appendix 1) and full specimen details are available on the Solanaceae Source website (www.solanaceaesource.org) and in the dataset for this study deposited in the Natural History Museum Data Portal (http://dx.doi.org/10.5519/0039445).

Citation of literature follows BPH-2 (Bridson 2004) with alterations implemented in IPNI (International Plant Names Index, http://www.ipni.org) and Harvard University Index of Botanical Publications (http://kiki.huh.harvard.edu/databases/publication_index.html). Following Knapp (2013) we have used the square bracket convention for publications in which a species is described by one author in a publication edited or compiled by another. These citations are the traditional “in” attributions such as Dunal in DC. for those taxa described by Dunal in Candolle’s *Prodromus Systematis Naturalis Regni Vegetabilis*. This work is cited here as Prodr. [A.P. de Candolle] and the names are thus attributed only to Dunal. For “ex” attributions we cite only the publishing author, as suggested in the *Code* (McNeill et al. 2012). Standard forms of author names are according to IPNI (International Plant Names Index, http://www.ipni.org).

## Taxonomic treatment

**The “African Non-Spiny (ANS)” clade *sensu* Bohs, Monographs in Systematic Botany from the Missouri Botanical Garden 104: 38. 2005.**

Solanum section Afrosolanum Bitter, Bot. Jahrb. 54: 440. 1917.

Lectotype species, designated by Seithe 1962, pg. 290: *Solanum terminale* Forssk.

Solanum section Lemurisolanum Bitter, Bot. Jahrb. 43: 436. 1917.

Lectotype species, designated by Seithe 1962, pg. 291: *Solanum madagascariense* Dunal

Solanum section Macronesiotes Bitter, Bot. Jahrb. 54: 532. 1917.

Lectotype species, designated by Seithe 1962, pg. 291: *Solanum imamense* Dunal

Solanum subgenus Lyciosolanum Bitter, Bot. Jahrb. 54: 425. 1917.

Type species: *Solanum aggregatum* Jacq. (=*Solanum guineense* L.)

Solanum section Quadrangulare Bitter, Bot. Jahrb. 54: 428. 1917.

Type species: *Solanum quadrangulare* Thunb. (=*Solanum africanum* Mill.)

Solanum section Benderianum Bitter, Bot. Jahrb. 54: 487. 1917.

Type species: *Solanum benderianum* C.H.Wright (as *Solanum benderianum* Schimp. =*Solanum runsoriense* C.H.Wright)

**Description.** Woody vines, occasionally shrubs or scandent shrubs, sometimes epiphytic; stems weak and clambering or erect, sometimes winged, pubescent or glabrous, the trichomes unbranched or variously branched, never strictly stellate. Sympodial units plurifoliate, the leaves never geminate. Leaves simple or more rarely shallowly pinnatifid, green, glabrous or variously pubescent with branched or unbranched trichomes, the trichomes usually denser on the leaf undersides; petioles well developed, in some vines apparently twining to aid in climbing. Inflorescences terminal, sometimes appearing lateral due to stem growth, unbranched, furcate or more usually many times branched, with up to 100 flowers, not bracteate; peduncle present or absent, often poorly distinguished from the leafy stem; pedicels articulated near the base or somewhat above the rachis leaving a small peg of tissue when the flower falls. Flowers 5-merous, actinomorphic, usually perfect, one species heterostylous and possibly dioecious (*Solanum runsoriense*); calyx 5-parted, glabrous or pubescent; corolla 5-parted, stellate to deeply stellate, white or more often purple, sometimes with a paler or darker area at the base of the lobes; stamens 5, the filaments equal, glabrous; anthers yellow or occasionally purple, ellipsoid or rectangular, separate to tightly connivent, smooth or papillate, dehiscing by terminal pores, these either elongating to slits with age or each remaining a single round opening; ovary bicarpellate, globose to conical, usually glabrous, occasionally hispidulous; style straight or slightly curved; stigma capitate to clavate, occasionally bi-lobed. Fruit a globose, elongate or fusiform berry, red, orange or blackish purple when ripe, glabrous, the pericarp thin or thicker and apparently spongy or woody, shiny or matte; calyx lobes in fruit usually not markedly accrescent, but sometimes almost enclosing the berry. Seeds flattened or ovoid reniform, sometimes appearing hairy from outgrowths of the lateral testal cell walls.

**Distribution.** Continental Africa, the southern tip of the Arabian Peninsula, the Comoro Islands and Madagascar.

**Discussion.** The ANS clade was first defined geographically by Bitter (1917), and has been confirmed with phylogenetic analysis of DNA sequences (Bohs 2005; Särkinen et al. 2013). Like many other clades of non-spiny solanums, it has few morphological synapomorphies that define it. The vining or scandent habit is one of the few characters all of the species have in common. The species of the group from Madagascar all share anthers that open by terminal pores that do not elongate, but this is not unique to this group (e.g., *Solanum havanense* and *Solanum troyanum* of the Geminata clade have similar anther structure; Knapp 2008). Although we have identified no unique synapomorphies the following combination of characters, coupled with geography, can be found in many species of the group: 1) vining or scandent habit, 2) usually branched terminal inflorescences, 3) brightly colored fruits (usually black or red), and 4) (probably) lobed leaves on juvenile shoots.

We have cited all type specimens we have seen for these species with barcodes or accession numbers as detailed in the Materials and Methods. In many cases no type collections or herbaria were cited in original descriptions. Where we have been unable to locate type material we have indicated types with “?” to help in future tracing of these specimens. We have not neotypified all of the many synonyms for *Solanum terminale* (see discussion under that species) and hope that future digitisation of herbaria will bring duplicates of these collections to light.

Most species in the group occur either on Madagascar or in continental Africa, so geography is a very practical first distinguishing character for identification; we provide keys to the species in each region in addition to the main key. We also provide a synoptic character list to help in identification of those specimens without locality information.

### Artificial key to the species of the African Non-Spiny Clade

**Table:** 

1	Leaves glabrous on both surfaces	**2**
–	Leaves with at least some pubescence on either surface (this sometimes sparse along veins and midrib)	**10**
2	Inflorescence few-flowered (usually less than 10), unbranched (at most furcate in *Solanum betroka*)	**3**
–	Inflorescence many -flowered (more than 10 flowers), usually many times branched	**5**
3	Flowers appearing fasciculate and axillary; corolla usually somewhat campanulate; fruit orange; South Africa	***Solanum guineense***
–	Flowers not appearing fasciculate; corolla spreading or the petals reflexed; Madagascar	**4**
4	Leaves clustered on short shoots; calyx lobes deltate, not divided to base; dry forests	***Solanum betroka***
–	Leaves not clustered on short shoots; calyx lobes long triangular, divided to the base; wet forests	***Solanum truncicola***
5	Flowers or fruits (or pedicel scars) in tightly packed groups on individual branches (these sometimes very short and the inflorescence appearing spicate)	***Solanum terminale***
–	Flowers spaced on the open inflorescence, often unevenly so	**6**
6	Leaves clustered on short shoots	***Solanum betroka***
–	Leaves spaced along the stem	**7**
7	Anthers opening by pores that elongate with age; mountains of continental Africa	***Solanum runsoriense***
–	Anthers opening by delineated pores that do not elongate with age; Madagascar	**8**
8	Leaves fleshy, thick and coriaceous, the venation not visible in dry specimens; fruit with thick pericarp (woody?)	***Solanum myrsinoides***
–	Leaves membranous to coriaceous, not markedly thick and fleshy, the venation visible in dry specimens; fruit with thin pericarp, the seeds visible through the berry wall	**9**
9	Petioles with long, simple trichomes (these not extending to the lamina); seeds 4-8 per berry; inflorescence axis thin and delicate	***Solanum trichopetiolatum***
–	Petioles glabrous or with minute dendritic trichomes; seeds 20-40 per berry; inflorescence axis robust	***Solanum madagascariense***
10	Leaf trichomes simple (unbranched)	**11**
–	Leaf trichomes branched (dendritic to echinoid)	**16**
11	Inflorescence axis unbranched, the flowers closely spaced	**12**
–	Inflorescence axis branched, often many times so	**13**
12	Leaves clustered along stem; fruit orange; South Africa	***Solanum guineense***
–	Leaves more or less evenly spaced along shoots; fruit purple or black; Madagascar	***Solanum truncicola***
13	Flowers or fruits (or pedicel scars) in tightly packed groups on individual branches (these sometimes very short and the inflorescence appearing spicate)	***Solanum terminale***
–	Flowers spaced on the open inflorescence, often unevenly so	**14**
14	Stems strongly quadrangular; at least some leaves with shallow lobes; plants of seashore and dune habitats	***Solanum africanum***
–	Stems terete; leaves not lobed; plants of forests and forest edges	**15**
15	Leaf pubescence very sparse, confined to the midrib or near the petiole; flowers not heterostylous; Madagsacar	***Solanum trichopetiolatum***
–	Leaf pubescence variable, not very sparse, along veins and lamina; flowers heterostylous; mountains of continental Africa	***Solanum runsoriense***
16	Abaxial leaf surfaces with tufts of trichomes in the vein axils (domatia)	**17**
–	Abaxial leaf surfaces with trichomes on lamina and/or along veins, not with prominent tufts in the vein axils (domatia)	**19**
17	Inflorescence many times branched, open and with many flowers (>20); calyx lobes broadly deltate and extremely short; petioles to 4 cm long, thin and flexuous; Mayotte	***Solanum macrothyrsum***
–	Inflorescence furcate, more congested and with fewer flowers (<20); calyx lobes deltate, not extremely short; petioles to 2.5 cm long, thicker; Madagascar	**18**
18	Calyx lobes 0.8-2 mm long; inflorescences with 10-16 flowers	***Solanum ivohibe***
–	Calyx lobes 4-6 mm long; inflorescences with 3-10 flowers	***Solanum sambiranense***
19	Abaxial leaf surfaces evenly pubescent on veins and lamina	**20**
–	Abaxial leaf surfaces pubescent only along the veins and midrib, the trichomes not extending to the lamina	**22**
20	Anther pores lengthening to slits with age; flowers heterstylous; leaves evenly distributed along branches; mountains of continental Africa	***Solanum runsoriense***
–	Anther pores not lengthening to slit with age; flowers not heterostylous; leaves usually at least somewhat clustered on short shoots; Madagascar	**21**
21	Leaves densely pubescent with golden (when dry) loosely dendritic trichomes; flowers >2 cm in diameter; anthers 4-6 mm long; widespread in Madagascar	***Solanum imamense***
–	Leaves sparsely pubescent with white (when dry) congested dendritic trichomes; flowers 2 cm in diameter or less; anthers 3.5–4 mm long; dry forests of southern Madagascar	***Solanum betroka***
22	Inflorescence unbranched, with few (usually < 5) flowers; pedicels 1.8-4.5 cm long	***Solanum humblotii***
–	Inflorescence many times branched, with many flowers (more than 5); pedicels 0.8-1.2 cm long	***23***
23	Anther pores lengthening to slits with age; flowers heterostylous; pedicels with pubescence like the inflorescence rachis; mountains of continental Africa	***Solanum runsoriense***
–	Anther pores not lengthening to slit with age; flowers not heterostylous; pedicels always glabrous; Madagascar	***Solanum madagascariense***

### Key to species of continental Africa

**Table:** 

1	Inflorescence unbranched and the flowers appearing fasciculate on short shoots; corolla lobes campanulate to spreading	***Solanum guineense***
–	Inflorescence branched, usually many times so (sometimes appearing spicate when branches very short); corolla lobes spreading to or reflexed	**2**
2	Stems strongly quadrangular; leaves fleshy and usually somewhat lobed; plants of coastal habitats	***Solanum africanum***
–	Stems terete; leaves not fleshy, not lobed on reproductive stems; plants of forests and forest edges	**3**
3	Fruit bright red, globose to elongate; seeds with conspicuous “hairs”; corolla lobed nearly to the base; flowers not heterostylous, densely clustered on inflorescence branches	***Solanum terminale***
–	Fruit black or purplish black, globose; seeds without conspicuous “hairs”; corolla lobed only halfway to the base; flowers heterostylous, evenly and widely spaced on inflorescence branches	***Solanum runsoriense***

### Key to species of Madagascar (incl. Comoro Islands and Mayotte)

**Table:** 

1	Leaf trichomes simple (unbranched) or absent	**2**
–	Leaf trichomes branched (dendritic or echinoid)	**7**
2	New growth of shoots completely glabrous	**3**
–	New growth of shoots pubescent (sometimes finely so)	**5**
3	Inflorescence unbranched, few-flowered; calyx lobes long triangular	***Solanum truncicola***
–	Inflorescence branched (sometimes only furcate); calyx lobes broadly deltate	**4**
4	Leaves fleshy, thick and coriaceous; pedicels 1.5–2.5 cm long; anthers ca. 7 mm long; berry usually apically pointed	***Solanum myrsinoides***
–	Leaves not fleshy, membranous to coriaceous; pedicels 0.4-1.1 cm long; anthers 2.5–4 mm long; berry globose or ellipsoid	***Solanum madagascariense***
5	Pubescence of new growth consisting of sparse, elongate, simple trichomes; petioles with sparse simple trichomes to 1.5 mm long	***Solanum trichopetiolatum***
–	Pubescence of new growth denser, the trichomes usually minute; petioles glabrous or with dense pubescence, not with elongate simple trichomes to 1.5 mm long	**6**
6	Leaves clustered on short shoots; inflorescences with <5 flowers; calyx lobes triangular; dry forests	***Solanum betroka***
–	Leaves spaced along the stem; inflorescences with >20 flowers; calyx lobes deltate to broadly deltate; widely distributed in wet forests	***Solanum madagascariense***
7	Abaxial leaf surfaces with tufts of trichomes in the vein axils (domatia)	**8**
–	Abaxial leaf surfaces with the trichomes on the lamina and/or along the veins, not in tufts in the vein axils	**10**
8	Calyx lobes foliaceous, often surpassing the corolla in flower	***Solanum sambiranense***
–	Calyx lobes not foliaceous	**9**
9	Calyx lobes broadly deltate, <1 mm long; inflorescences many times branched, with more than 20 flowers; Mayotte	***Solanum macrothyrsum***
–	Calyx lobes deltate, 0.8–2 mm long; inflorescences furcate, with 10–16 flowers; Fianarantsoa	***Solanum ivohibe***
10	Leaves pubescent on lamina surface; principal veins diverging 45° from the midvein	**11**
–	Leaves pubescent only along veins and midrib; principal veins diverging 60-90° from the midvein	**12**
11	Leaves densely pubescent with golden (when dry) loosely dendritic trichomes; flowers >2 cm in diameter; anthers 4-6 mm long; widespread	***Solanum imamense***
–	Leaves sparsely pubescent with white (when dry) congested dendritic trichomes; flowers 2 cm in diameter or less; anthers 3.5-4 mm long; dry forests	***Solanum betroka***
12	Inflorescence unbranched; pedicels 1.8-4.5 cm long, sparsely pubescent	***Solanum humblotii***
–	Inflorescence many times branched; pedicels 0.4-1.1 cm long, always glabrous (even when inflorescence rachis is pubescent)	***Solanum madagascariense***

## Synoptic character list

Plants of coastal dunes and scrub: *Solanum africanum*, *Solanum guineense*

Plants of montane bamboo forests: *Solanum runsoriense*

Plants of dry forests in southern Madagascar: *Solanum betroka*

Plants epiphytic: *Solanum myrsinoides*, *Solanum truncicola*

Stems strongly quadrangular: *Solanum africanum*

Bark of older stems corky: *Solanum madagascariense*, *Solanum terminale*

At least some leaves with lobes on reproductive shoots: *Solanum africanum*, *Solanum betroka*

Leaves thick and fleshy: *Solanum africanum*, *Solanum myrsinoides*

Both leaf surfaces densely pubescent: *Solanum imamense*, *Solanum terminale*

Abaxial leaf surfaces with tufts of trichomes in vein axils (domatia): *Solanum ivohibe*, *Solanum macrothyrsum*, *Solanum sambiranense*

Leaf venation appearing strongly parallel near the midrib (veins diverging at 60–90° from midvein): *Solanum humblotii*, *Solanum madagascariense*

Inflorescences unbranched: *Solanum betroka*, *Solanum guineense*, *Solanum humblotii*, *Solanum truncicola*

Calyx lobes very broadly deltate (obscure): *Solanum macrothyrsum*, *Solanum madagascariense*

Calyx lobes long-triangular: *Solanum humblotii*, *Solanum truncicola*

Calyx lobes foliaceous: *Solanum imamense*

Anther pores elongating to slits when dry (and with age): *Solanum africanum*, *Solanum guineense*, *Solanum runsoriense*, *Solanum terminale*

Anther pores never elongating to slits: *Solanum betroka*, *Solanum humblotii*, *Solanum imamense*, *Solanum ivohibe*, *Solanum madagascariense*, *Solanum myrsinoides*, *Solanum sambiranense*, *Solanum trichopetiolatum*, *Solanum truncicola*

Anther surfaces papillate: *Solanum madagascariense*, *Solanum myrsinoides*, *Solanum terminale*

Fruit red: *Solanum terminale*

Fruit orange: *Solanum guineense*

Fruit black: *Solanum africanum*, *Solanum betroka*, *Solanum imamense*, *Solanum runsoriense*

Fruit globose (round): *Solanum africanum*, *Solanum guineense*, *Solanum imamense*, *Solanum madagascariense*, *Solanum runsoriense*, *Solanum terminale*, *Solanum trichopetiolatum*

Fruit apically pointed or fusiform: *Solanum betroka*, *Solanum imamense*, *Solanum sambiranense*, *Solanum terminale*, *Solanum truncicola*

Seeds appearing hairy from elongate lateral cell walls: *Solanum imamense*, *Solanum terminale*

### Species descriptions

#### Solanum africanum

Taxon classificationPlantaeSolanalesSolanaceae

Mill., Gard. Dict. ed. 8, no. 26. 1768.

Figures 1A , 3

Solanum quadrangulare Thunb. ex L.f., Suppl. 147. 1781. Type. South Africa. Western Cape: “CBS [Caput Bona Spei]”, *P. Thunberg [476*] (lectotype, designated here; LINN [LINN 248.26]).
Solanum crassifolium Lam., Tabl. Encycl. 2: 16. 1794. Type. Based on Dillenius, Hortus Elthamensis 365, t. 273. 1732 (lectotype, designated here: Dillenius, Hortus Elthamensis 365, t. 273. 1732)
Witheringia crassifolia (Lam.) Dunal, Hist. Nat. Solanum 108. 1813. Type. Based on Solanum crassifolium Lam.
Solanum bracteatum Thunb., Act. Gorensk. [Fl. Cap. 2: 57]. 1818. Type. South Africa. No specimens found.
Solanum aggerum Dunal, Prodr. [A. P. de Candolle] 13(1): 103. 1852. Type. South Africa. Western Cape: “Cape, in oasis Zitzikania”, *P.P.S. Krauss s.n.* (holotype: G [G00301688]; possible isotype: MO [MO-1811850]).
Solanum exasperatum Drège ex Dunal, Prodr. [A. P. de Candolle] 13(1): 104. 1852. Type. South Africa. KwaZulu-Natal: “in frutecetis haud procul a maris littore, inter Umcomas [Unkomaas] et Natal”, *Drège, J.F. s.n.* (holotype: G-DC [G00145074]; isotype: MPU [MPU011259]).
Solanum geniculatum Drège ex Dunal, Prodr. [A. P. de Candolle] 13(1): 105. 1852. Type. South Africa. KwaZulu-Natal: “in frutecetis inter Umsamculo et Omcomas [Unkomaas], haud procul a maris littore (V, C)”, *Drège, J.F. s.n.* (holotype: G-DC [G00145049]; isotype: K [K000414161]).
Solanum longipes Dunal, Prodr. [A. P. de Candolle] 13(1): 85. 1852. Type. South Africa. KwaZulu-Natal: Natal, “locis natalis incestus”, *Drège, J.F. s.n.* (holotype: G-DC [G00144869]).
Solanum quadrangulare L.f. var. integrifolium Dunal, Prodr. [A. P. de Candolle] 13(1): 77. 1852. Type. South Africa. Western Cape: Stellenbosch, *J.F. Drège s.n.* (holotype G-DC [G00144675]; isotype: K [K000414165]).
Solanum quadrangulare L.f. var. sinuato-angulatum Dunal, Prodr. [A. P. de Candolle] 13(1): 77. 1852. Type. South Africa. Western Cape: between Cape Town and Stellenbosch, *J.F. Drège s.n.* (holotype: G-DC; isotype: K [K000414162]).
Solanum quadrangulare L.f. var. glabrum Dammer, Bot. Jahrb. Syst. 38: 179. 1906. Type. South Africa. Western Cape: “sudwestliches capland: Riversdale, *Rust 430*, *484*” (type at B [?]; no duplicates found of either collection).
Solanum quadrangulare L.f. var. crassifolium (Lam.) Bitter, Bot. Jahrb. Syst. 54: 431. 1917. Type. Based on Solanum crassifolium Lam.

##### Type.

“Solanum dulcamarum Africanum, foliis crassis hirsutis”, cultivated in England, at James Sherard’s garden in Eltham (Hortus Elthamensis) in 1726, originally from South Africa, Cape of Good Hope, *Anonymous s.n.* (lectotype, designated here: Dillenius, Hortus Elthamensis 365, t. 273. 1732). “Solanum Afric., frutescens, foliis angulatis, crassis et hirsutis, fl. caerulei. H. Eltham 1726” (epitype, designated here: OXF [Dill-HE 273-352, sheet 1]).

##### Description.

Scrambling vine to shrublet, 0.5–2 m. Stems strongly 4-winged, the wings less prominent on older stems, glabrous, minutely papillate or sparsely to moderately pubescent with antrorse simple uniseriate trichomes to 0.5 mm long, these arising from a multicellular base and deciduous, leaving the base and the stems apparently toothed; new growth glabrous or minutely papillate especially on leaf margins. Bark of older stems pale yellowish brown. Sympodial units plurifoliate, the leaves not geminate, evenly distributed along branches. Leaves simple or shallowly lobed, (1)2–5.2 cm long, (0.6)0.9–2.5 cm wide, elliptic to narrowly elliptic, thick and fleshy, both surfaces glabrous; major veins 3–4 pairs, not easily visible, the finer venation not visible; base cuneate and decurrent onto the stem; margins entire or with up to 3 shallow lobes, pubescent with antrorse uniseriate trichomes from broad bases like those of the stems giving an appearance of minute teeth, revolute in herbarium specimens; apex acute to slightly rounded-acute; petiole indistinct, if discernible to 1 cm with a narrow wing of leaf tissue along entire length. Inflorescences terminal, 1–10 cm long, 2–4(6) times branched, with 10–30 flowers, glabrous or with scattered bulbous-based simple uniseriate trichomes < 0.5 mm long; peduncle 0.5–2.5 cm long, sometimes purple as are the pedicels; pedicels 0.8–1.2 cm long, ca. 0.5 mm in diameter at the base, ca. 1.5 mm in diameter at the apex, nodding or spreading at anthesis, glabrous or sparsely pubescent with simple uniseriate trichomes < 0.5 mm long, articulated at or near the base, leaving a tiny raised peg ca. 0.5 mm in the inflorescence axis; pedicel scars irregularly spaced in clumps. Buds globose becoming ellipsoid before anthesis, strongly exserted from the calyx tube long before anthesis. Flowers 5-merous, apparently all perfect. Calyx tube 1–1.5 mm long, openly cup-shaped, the lobes 1–1.5 mm long, deltate, fleshy and sometimes dark purple, the margins thickened in dry material, with a tuft of simple uniseriate trchomes at the tip, these ca. 0.25 mm long, yellowish when dry. Corolla 1.2–1.6 cm in diameter, violet-blue, stellate, lobed ¾ of the way to the base, the lobes 6–6.5 mm long, 2.5–4 mm wide, spreading at anthesis, glabrous adaxially, densely pubescent or papillate abaxially on the tips and margins, the trichomes < 0.2 mm long. Stamens equal; filament tube absent; free portion of the filaments 1–1.5 mm long, minutely papillate at the base adaxially; anthers 2.5–3 mm long, 1–1.5 mm wide, ellipsoid, loosely connivent, bright yellow, smooth abaxially, poricidal at the tips, the pores lengthening to slits with age, the tips paler and thickened in dry material. Ovary globose, glabrous; style 5.5–6.5 mm long, glabrous, apparently exserted from the bud before anthesis; stigma capitate, the surface minutely papillate. Fruit a globose to ellipsoid berry, 0.7–0.8 cm in diameter, purplish black when mature, juicy, the juice purple, the pericarp thin and shiny; fruiting pedicels 1.1–1.3 cm long, ca. 1 mm in diameter at the base, somewhat woody (?), nodding or spreading; fruiting calyx lobes slightly reflexed. Seeds 6–10 per berry, ca. 3 mm long, ca. 2.5 mm wide, flattened reniform with thickened margins, rusty brown, the surfaces minutely pitted, the testal cells pentagonal in outline.

**Figure 3.**
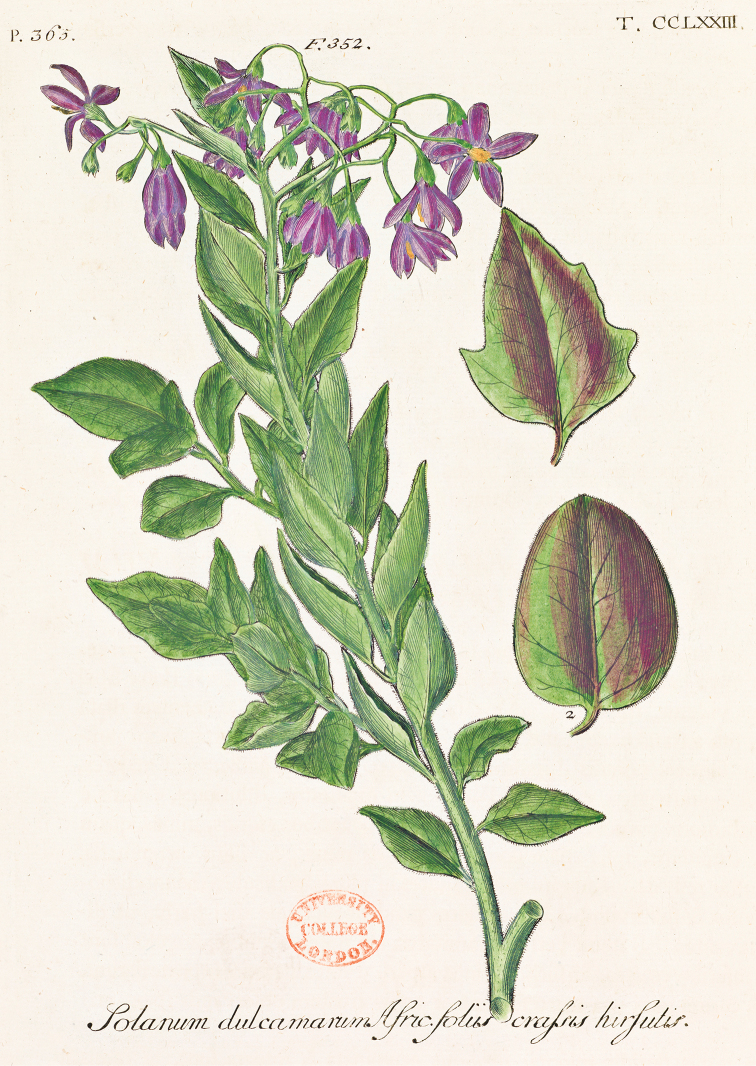
*Solanum africanum* Mill. Lectotype of *Solanum africanum* “Solanum Afric., frutescens, foliis angulatis, crassis et hirsutis, fl. caerulei H. Eltham 1726” from Dillenius, *Hortus Elthamensis* 365, t. 273. 1732. Reproduced with permission of the Natural History Museum Library.

##### Distribution

**Figure 4.**
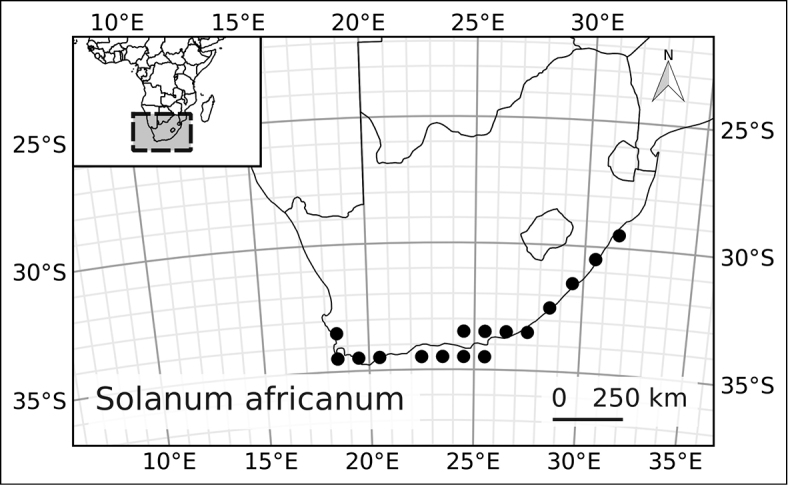
Distribution of *Solanum africanum* Mill.

(Figure 4). Endemic to the coastal region of South Africa (most commonly collected in Eastern and Western Cape provinces, a few collections from North West, Gauteng and KwaZulu-Natal provinces).

##### Ecology and habitat.

Dunes and strand habitats near the sea; 0–50 m elevation.

##### Common names and uses.

South Africa: dronkbessie (SANBI, http://www.ispotnature.org/species-dictionaries/sanbi/Solanum%20africanum).

##### Preliminary conservation status

(IUCN 2014). Least Concern (LC). EOO 62,716 km^2^ (LC), AOO 36 km^2^ (EN). *Solanum africanum* is widely distributed along the southern part of South Africa, and although the AOO calculated here indicates some conservation concern, this is in part due to the releatively low number of specimens we have examined. The species occurs in several protected areas, and is likely to be more common than our data suggest.

##### Discussion.

*Solanum africanum* was recognised by Linnaeus as part of his *Solanum dulcamara* L. It is superficially similar to that species, but differs in its ellipsoid, rather than tapering, anthers (see Knapp 2013), its purple berries and in its strongly angled stems (Fig. 1A). It is sympatric with and could be confused with *Solanum guineense*, but the branched inflorescence with smaller flowers distinguish *Solanum africanum*. The fruits of *Solanum guineense* are orange when ripe. Leaf shape and pubescence vary a great deal throughout the range of *Solanum africanum*; plants from drier habitats near the sea appear to have fleshier leaves, but pubescence does not seem to be environmentally influenced. Juvenile leaves appear to have more deeply incised margins, but some flowering specimens also have incised leaves.

Miller (1768) coined the binomial *Solanum africanum* as a replacement at the species level for Linnaeus’ variety of *Solanum dulcamara* “β Solanum dulcamarum africanum, foliis crassis hirsutis” (Linnaeus 1753), correctly recognising its distinctness. The only flowering material either author cited was the illustration (Fig. 3) from Dillenius (1732), although it is clear that Miller grew the plant at Chelsea and knew it from live material, at least vegetatively. He states clearly its differences to *Solanum dulcamara* – “some who have supported this and our common nightshade to the be same, which is certainly a great mistake, for this sort will not live abroad [outside] through the winter in England in any situation, nor does it produce flowers here with any treatment, for there are plants in the Chelsea Physic Garden of several years old, which have been differently managed, and yet have never flowered.” None of the four sheets in the Sherard herbarium at OXF match the plate from *Hortus Elthamensis* (Dillenius 1732) exactly (as is often the case, see *Solanum campechiense* L., Knapp and Jarvis 1989); the plate seems to combine some elements from several of the sheets at OXF, or may have been drawn from live plants. Two are sterile branches, and two bear one flowering branch each in addition to a sterile branch with juvenile leaf morphology. The epitype we have selected is the sheet dated “H. Eltham 1726” with one branch whose inflorescence mostly closely matches that in the plate (Dill-HE 273-352, sheet 1) and that bears the date 1726.

*Solanum quadrangulare*, the name by which this plant was long known, was the name coined for this species by Carl Peter Thunberg, Linneaus’ pupil who collected extensively in South Africa. It refers to its quadrangular stems. We have selected the sheet in the herbarium of the Linnean Society of London (LINN 248.26) as the lectotype of this name; it has an annotation in Linneaus’ handwriting and a reference to Thunberg (“T 476/CBS”).

*Solanum crassifolium* was based on the same Dillenius (1732) illustration as *Solanum africanum*, and we consider the names homotypic.

Thunberg’s species *Solanum bracteatum* first appears to have been effectively published in 1818, in his *Flora Capensis* (Thunberg 1818). In that work he refers to “Act. Gor.” that is probably an earlier place of publication (in the entry for *Solanum quadrangulare* he refers to “Acta Gor. 1812”). This is a reference to the botanic gardens of Count Alexis de Razumovsky at Gorenki, just outside Moscow, whose director was Friedrich E.L. Fischer, who later went on to be director of the botanic gardens in St. Petersburg. We have been unable to find an 1812 publication attributable to the “Société Phytographique de Gorenki”, nor is *Solanum bracteatum* listed among the plants grown at Gorenki at that time (Fischer 1812). Fischer also edited the *Mémoires de la Société Impériale des Naturalistes de Moscou*, and in the preface to volume 5 (Fischer 1817) refers to the first four volumes of the *Mémoires* being consumed by flames, presumably during the Napoleonic sack of Moscow, and the fusion of the Gorenki society with that of Moscow (“Les Mémoires de la Société Impériale des Naturalistes de Moscou consistant en quatre volumes, avoient, à la catastrophe de 1812 les sort de tant d’autres objects d’être consumes par les flammes. Il m’a paru important de faire paroître aussitôt de possible les matériaux qui se sont depuis rassemblés et qui, apres la réunion de la Société phytogeographique de Gorenki à la nôtre, sont devenus si intéressans pour la botanique – et de recommencer l’impression d’un ouvrage brûlé….”). An article by Thunberg (1817) in the same volume of the Mémoires does not include *Solanum bracteatum*, nor any references to “Act. Gor.” It is possible (and seems to us probable) that the original Thunberg publication scheduled for publication in 1812 never appeared in print, and thus this name is effectively published only in the Flora Capensis of 1818 (Thunberg 1818). The 1812 publication may, however, turn up, and that then would be the correct date of publication of *Solanum bracteatum*. We have found no specimens or other original material for *Solanum bracteatum*. Thunberg (1818) distinguished *Solanum bracteatum* from *Solanum quadrangulare* by its bracteate, less-branched inflorescences, but the branched inflorescence and black fruit clearly indicate it is a synonym of *Solanum africanum*, rather than *Solanum guineense*.

Dunal (1852) described a variety of taxa at both the species and subspecies level that we consider synonymous with *Solanum africanum*. *Solanum aggerum* was described from a specimen collected at “Zitzikania” in 1839 held in “herb. Boiss.”; a specimen at MO with the locality “Goukania, Feb” is possibly an isotype. These all represent leaf shape and pubescence variants of *Solanum africanum*. We have been unable to trace any duplicate material of the two collecctions cited by Dammer (1906) in the protologue of Solanum quadrangulare var. glabrum (*Rust 430*, *484*); these specimens were likely to have been destroyed in Berlin.

##### Selected specimens examined.

**South Africa**. **Eastern Cape**: Thornhill, 5 miles W, Dist. Port Elizabeth, 27 Apr 1947, *Acocks 13666* (K); Bushman’s River Mouth, left hand bank from sea, Oct 1973, *Arnold 594* (K); Baviaans Kloof, 3 Jun 1976, *Bayliss 7484* (G); Port Elizabeth, near the Block House, 13 Dec 1813, *Burchell 4342* (K); Port Elizabeth, at Cape Recife, 24 Dec 1813, *Burchell 4389* (K); Van Staden’s River, near the ford (Uitenhage Division), 9 Feb 1814, *Burchell 4668* (K, LE); Kowie, Dist. Bathurst [Kowie River?], Aug 1929, *Dyer 2010* (K); Slang River, Div. Humansdorp, Mar 1922, *Fourcade 2178* (K); Kahoon River, near river mouth, Div. East London, 15 Apr 1900, *Galpin 2690* (K); Alexandria forest, 28 Apr 1931, *Galpin 10687* (BM,K); Uitenhage, 33°46’ S, 25°24’ W, Jan 1899, *Haagner s.n.* (K); Boknesstrand, Richmond, Dist. Alexandria, 20 May 1954, *Johnson 930* (K); Port Elizabeth, 1894, *Laidley & Co. 274* (G); Tzitzikama Park, Storm’s River mouth near beach, Dist. Humansdorp, 30 Jan 1966, *Liebenberg 7861* (K); Sea View, 16 Mar 1931, *Long 395* (K); Quora mouth, 25 Mar 1973, *Strey 11187* (E, K); Klipdrift, Div. Humansdorp, May 1860, *Thode A-2494* (K); Bathurst, Kowie W., May 1914, *Tyson s.n.* (G); Alexandria Forest Station, Dist. Alexandria, 26 Feb 1986, *Wells 2780* (K); Uitenhage, *Zeyher 75* (K). **KwaZulu-Natal**: Umzimkulo River, *Drège s.n.* (K); Uvongo Beach, Port Shepstone region, Apr 1968, *Liebenberg 8120* (K); Illovo, lower Illovo [River], 4 May 1894, *Medley Wood 6390* (BM, E, K); Umdloti, 23 Feb 1969, *Ross 1991* (EA, K). **North West**: Zwartkopsrivier, in valley and surrounding hills from Villa Paul Mare to Uitenhaag, *Ecklon & Zeyher s.n.* (CAS). **Western Cape**: Riversdale, playas y dunas de Witsand, 20 Jan 2008, *Aedo et al. 18685* (MA); Strandfontein, 19 Jun 1956, *Baker 1001* (BM); Kynsna, 4 May 1963, *Bayliss 1402* (G); Bilou River, Knysna District, Mar 1910, *Fourcade 626* (K); Gordan’s Bay, 16 Apr 1914, *Garside 484* (K); Grootbos Forest, Stanford, E of Hermanus, 2 Feb 1982, *Hepper 7320* (K); Cape Agulhas, southernmost tip of Africa, 2 Feb 1982, *Hepper 7321* (K); Bredasdorp de Hoop, edge of lake at south end of nature reserve, 3 Feb 1982, *Hepper 7323* (K); Muizenberg, Diep River, prope Muizenberg, Mar 1899, *MacOwan 1930* (BM, G, K); Cape of Good Hope, *Nelson s.n.* (BM); Strand, Stellenbosch Division, 26 Feb 1942, *Parker 3652* (K); Mossel Bay, near Klas Meyer’s, Aug 1847, *Prior s.n.* (K); Strand, Hottentots Holland, Jan 1880, *Rogers s.n.* (K); False Bay, prope Muizenberg, 3 Apr 1892, *Schlechter 640* (G); Muizenberg, by railway, 11 Mar 1896, *Wolley-Dod 1018* (K); Walk Bay, near Capetown, Mar 1910, *Worsdell s.n.* (K).

#### Solanum betroka

Taxon classificationPlantaeSolanalesSolanaceae

D’Arcy & Rakot., Fl. Madag., Fam. 176: 77. 1994.

Figure 5

##### Type.

Madagascar. Toliara: Forêt de Zombitsy (Sakaraha), aux confins des basins du Fiherenana et de L’Onilahy, forêt tropophile sur sables siliceux de l’Isalo, 26-29 March 1955, 600-850 m, *H. Humbert, L. Bègue & R.P.R. Capuron 29654* (holotype: P [P00349299]; isotypes: K [K000414193], MO [MO-150886]).

##### Description.

Subshrub or shrub to canopy liana, of extremely variable height. Stems terete, finely dendritic pubescent at the tips, glabrescent. New growth unevenly pubescent, with densely branched dendritic to echinoid uniseriate trichomes ca. 0.2 mm long. Bark of older stems smooth, almost white to brown, glabrous. Sympodial units plurifoliate, the leaves not geminate, evenly distributed along young branches and clustered on short shoots on older plants. Leaves simple, 3-4.5 cm long, 1.2-2(3) cm wide, ovate to elliptic, rarely obovate, membranous to chartaceous, concolorous to weakly discolorous, both surfaces glabrous or pubescent with dendritic trichomes ca. 0.1 mm long; major veins 5–7 pairs, spreading at ca. 45° to the midvein and forming loops, the finer venation a prominent network of fine brown veins visible abaxially in herbarium specimens; base cuneate to truncate; margins entire or with up to 2 pairs of lobes in the lower half and with sinuses halfway to the midrib, the lobes rounded at the tips; apex acute to rounded; petiole 0.7–1.5 cm long, slender, densely dendritic-pubescent like young stems, glabrescent. Inflorescences terminal on short shoots, 2–5 cm long, unbranched or furcate, with 1–3 flowers, evenly dendritic-pubescent, the trichomes 0.1–0.2 mm long; peduncle absent or up to 2.5 cm long; pedicels ca. 1.5 cm long, apically dilated, ridged when dry, evenly dendritic-pubescent like the rachis, articulated 0–0.5 mm from base; pedicel scars irregularly spaced 1–5 mm apart, prominent. Buds ellipsoid, the corolla only just emerging from the calyx tube before anthesis, the tip of the bud shorter than the calyx lobes. Flowers 5-merous, apparently all perfect. Calyx tube 2–3 mm long, cup-shaped, the lobes 2–3 mm long, 1.5–2 mm wide at base, unevenly deltate, acute, evenly pubescent with dendritic trichomes like those of the rest of the inflorescence, with simple uniseriate trichomes up to 0.2 mm long on the margins. Corolla 1.2–2 cm in diameter, violet, stellate, lobed almost to base, the lobes 5–10 mm long, 2–3 mm wide, ovate to narrowly triangular, glabrous adaxially, sparsely pubescent abaxially, more densely pubescent at the tips with translucent, simple and/or dendritic trichomes. Stamens equal; filament tube ca. 1 mm long; free portion of the filaments 1–2 mm long, glabrous; anthers 3.5–4 mm long, ca. 1.5 mm wide, broadly oblong or ellipsoid, loosely connivent, smooth abaxially, poricidal at the tips, the pores much smaller than anther apices, ca. 0.4 mm in diameter, clearly delineated and not lengthening with age. Ovary conical, glabrous; style 7–8 mm long, protruding 2–3 mm beyond the anthers, curved, glabrous; stigma clavate, dark, the surface minutely papillose. Fruit an elongated ovoid berry, ca. 1.3 cm long, ca. 1 cm in diameter (immature?), apically pointed, the pericarp thin, glabrous; fruiting pedicels 1.5–3 cm long, ca. 0.8 mm diameter at base, pendent, ridged; fruiting calyx slightly accrescent, the lobes to ca. 5 m long, ca. 4 mm wide and forming a loose cup around the developing fruit. Seeds not known.

**Figure 5.**
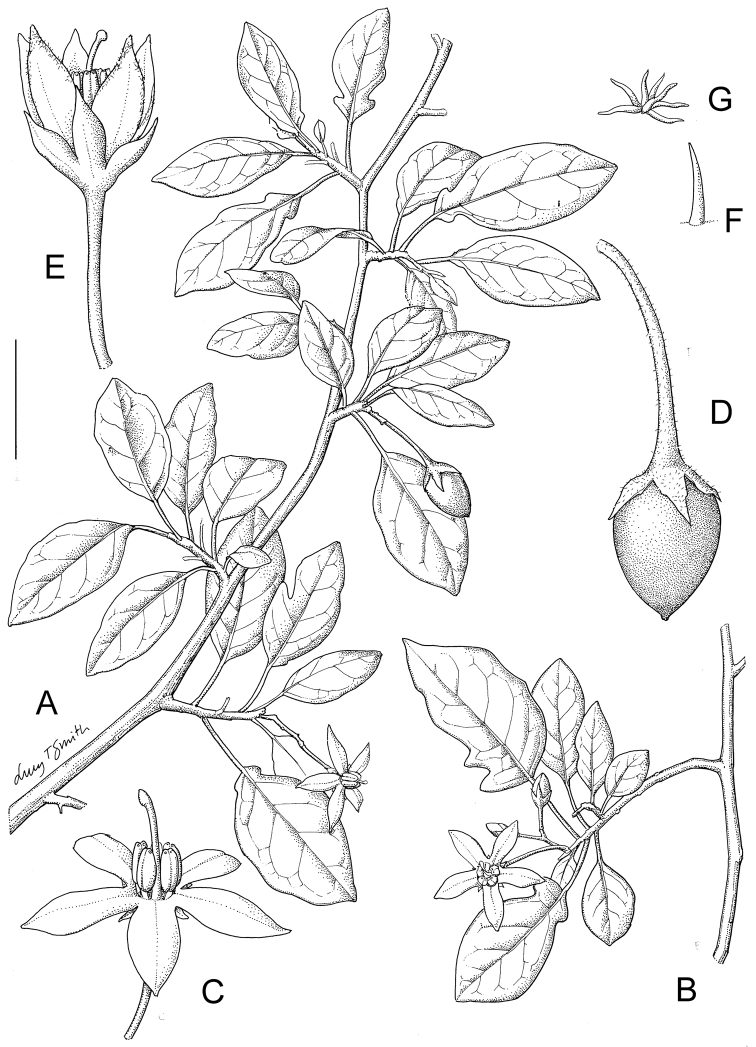
*Solanum betroka* D’Arcy & Rakot. **A** Flowering stem **B** Flowering stem with lobed leaf **C** Flower **D** Immature berry **E** Flower just before anthesis, showing elongate calyx lobes **F** Simple trichome **G** Dendritic trichome (Based on: **A–D, F, G** *Bosser 17370*; **E** *Leroy 3*). Scale bar: **A, B** = 2 cm; **C, E** = 7 mm; **D** = 1 cm; **F, G** = 0.3 mm. Drawn by Lucy T. Smith.

##### Distribution

**Figure 6.**
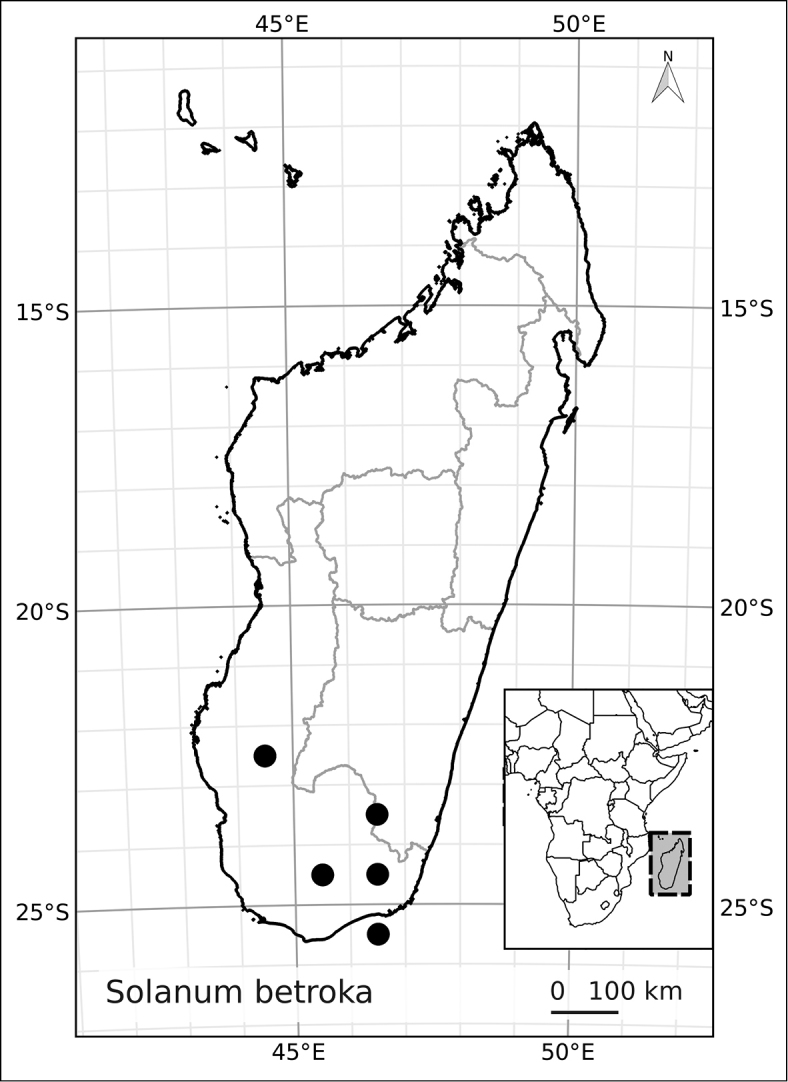
Distribution of *Solanum betroka* D’Arcy & Rakot.

(Figure 6). Endemic to Toliara province in southern Madagascar.

##### Ecology and habitat.

Open dry forest and scrub on limestone or sand; 600–1100 m elevation.

##### Common names and uses.

None recorded.

##### Preliminary conservation status

(IUCN 2014). Least Concern (LC). EOO 62,716 km^2^ (LC), AOO 36 km^2^ (EN). *Solanum betroka* is confined to the arid southern part of Madagascar, with some disjunct populations to the north, giving the EOO indicating least concern. Given the paucity of collections of all of the Malagasy members of the ANS clade, and the widespread and continuing habitat alteration in Madagascar, we feel this species would merit conservation concern, but the AOO may also be indicative of collection deficit or bias.

##### Discussion.

*Solanum betroka* is a spindly shrub or liana endemic to southern Madagascar. It has small membranous lobed leaves with prominent finer venation clustered on the tips of branches, no more than 3 flowers per inflorescence, and no tufts of trichomes in the axils of the major leaf veins on leaf undersides (domatia). It is the only species of *Solanum* endemic to Madagascar not a member of the Leptostemonum clade to commonly exhibit lobed leaves (Fig. 5) on reproductive stems; lobed leaves in *Solanum madagascariense* occasionally occur but are rare.

*Solanum betroka* can be difficult to distinguish from the very similar and possibly closely related *Solanum sambiranense* and *Solanum imamense*. It differs from *Solanum sambiranense* by its ovate to elliptic (versus elliptic to obovate) leaves under 5 cm (versus over 5 cm) long, with cuneate to truncate (versus attenuate) bases, inflorescences with 1–3 (versus 3–10) flowers, and calyx lobes 2–3 mm (versus 4–6 mm) long. *Solanum betroka* bears a strong resemblance to *Solanum sambiranense*: both have a clearly visible brown fine venation network, membranous glabrescent leaves that dry greenish brown, and a similar habit. Typical representatives of the southern *Solanum betroka* and northern *Solanum sambiranense* are clearly distinct but specimens from the centre of Madagascar are more difficult to determine. Further sampling may prove that these two taxa are in fact conspecific. *Solanum betroka* can be distinguished from *Solanum imamense* by its inflorescences with 1–3 (versus 7–13) flowers, flowers under 2 cm (versus over 2 cm) in diameter, and anthers 3.5–5 mm (versus 4–5 mm) long.

*Solanum betroka* has also been confused with unarmed specimens of the spiny solanums *Solanum batoides* D’Arcy & Rakot. and *Solanum erythracanthum* Dunal; these can be distinguished easily based on pubescence and anther morphology. *Solanum betroka* has dendritic trichomes and ellipsoid anthers while *Solanum batoides* and *Solanum erythracanthum* have the stellate trichomes and long-tapered anthers typical of members of the Leptostemonum clade (Vorontsova et al. 2013; Vorontsova and Knapp 2016).

*Solanum betroka* occurs primarily in the unique arid southern ecoregion of Madagascar, with a few records further north in the somewhat wetter western ecoregion (Humbert 1955; Faramalala 1988, 1995). It is the only member of the African non-spiny clade restricted to such severely dry climate, although a few spiny *Solanum* species also occur in the area: *Solanum batoides*, *Solanum bumeliifolium* Dunal, *Solanum croatii* D’Arcy & R.C.Keating, and *Solanum heinianum* D’Arcy & R.C.Keating; the related *Solanum imamense* is partly sympatric with *Solanum betroka* in the wetter more northern parts of its distribution range. The habitat niche of *Solanum betroka* is significantly different from that of *Solanum imamense* and *Solanum sambiranenese* both of which occur in wetter habitats further north.. The type collection has smaller leaves with more lobes and denser indumentum than the majority of specimens, and does not exhibit the characteristic pronounced brown venation on dry leaves.

##### Selected specimens examined.

**Madagascar. Toliara**: Tulear-Ihosy km 44, 12 Nov 1967, *Bernardi 11407* (G, P); environs de Betroka, Feb 1963, *Bosser 17370* (K, MO, P, TAN); Ambovombe, 2 Feb 1926, *Decary 3729[a*] (MO, NY); Taolagnaro (F-Dauphin), Angavo, massif de l’Angavo á l’Est d’Antanimora, 16 Jul 1926, *Decary 4443* (K, MO, P); Amboasary-Sud, Ampasimpolaka à l’est d’Ambovombe, 29 Jun 1931, *Decary 9046* (MO, P); Bekily, Ampandrandava, 1943, *Herb Jard Bot Tananarive 6083* (P); Zombitsy, Forêt de Zombitsy (Sakaraha) aux confins des bassins du Fiherenana et de l’Onilahy, Mar 1955, *Humbert et al. 29627* (K, MO, NY, P); 57 km de Tulear, P.K. 888, 23 Oct 1966, *Leroy 3* (P); Forêt de Zombitse [Zombitsy], 13 Apr 1961, *Peltier & Peltier 3069* (P, TAN); Bekily, environs d’Ampandrandava (entre Bekily et Tsivory), Nov 1942, *Seyrig 371* (MO, P).

#### Solanum guineense

Taxon classificationPlantaeSolanalesSolanaceae

L., Sp. Pl. 184. 1753.

Figure 7

Solanum sempervirens Mill., Gard. Dict. ed. 8, no. 25. 1768, nom. illeg. superfl. Type. Based on Solanum guineense L.
Atropa solanacea L., Mant. Pl. Alt. 205. 1771. Type. Based on Solanum guineense L.
Solanum aggregatum Jacq., Collectanea [Jacquin] 4: 124. 1791. Type. Based on Atropa solanacea L. (=Solanum guineense L.)
Solanum dasypus Drège ex Dunal, Prodr. [A. P. de Candolle] 13(1): 161. 1852. Type. South Africa. Western Cape: Riebeck’s Castle, Malmesbury Div., *J.F. Drège 1933* (holotype: G-DC ; isotypes: K, MO [MO-5471839]).
Solanum monticolum Dunal, Prodr. [A. P. de Candolle] 13(1): 161. 1852. Type. South Africa. Western Cape: Piquetberg, Jun 1837 (1838), *J.F. Drège s.n.* [Lycium 7865] (holotype: G-DC [G00145669]; isotypes: BM [BM001071606], K [K000414167, K000414168], MPU [MPU011277], S [S-11-5670]).
Solanum dasypus E.Mey., Zwei Pflanzengeogr. Docum. (Drège) 103. 1853, nom. illeg., non Solanum dasypus Dunal, 1852. Type. Based on Solanum dasypus Drège ex Dunal
Solanum dasypodum St.-Lag., Ann. Soc. Bot. Lyon 7: 135. 1880, nom. illeg. superfl. Type. Based on Solanum dasypus Dunal
Solanum bachmannii Dammer, Bot. Jahrb. Syst. 38: 188. 1906. Type. South Africa. Western Cape: Dist. Malmesbury, Darling, Aug 1883, *F. Bachmann 599* (type B?, destroyed; no duplicates found).
Solanum aggregatum Jacq. var. bachmannii (Dammer) Bitter, Bot. Jahrb. Syst. 54: 427. 1917. Type. Based on Solanum bachmannii Dammer

##### Type.

South Africa. “In Guinea”, *Anonymous s.n.* (lectotype, designated by Wijnands 1983, pg. 193: LINN 246.4)

##### Description.

Lax scrambling many-stemmed shrub to 1 m tall, rhizomatous (?) with extensive underground root system (*van Breda 4164*). Stems erect or pendent, terete, glabrous to densely and finely pubescent with minute simple uniseriate gland-tipped trichomes, occasionally also with longer simple 4–5 celled uniseriate trichomes to 0.5 mm long; new growth densely glandular pubescent. Bark of older stems pale yellow-green or greenish brown. Sympodial units plurifoliate, the leaves not geminate, clustered on short shoots or less commonly evenly distributed along branches. Leaves simple, 1.5–6 cm long, 0.7–3.3 cm wide, ovate or more rarely elliptic, highly variable in size along single branches, apparently somewhat fleshy, both surfaces glabrous to densely and finely pubescent with gland-tipped simple uniseriate trichomes < 0.5 mm long and longer, eglandular simple uniseriate trichomes ca. 0.5 mm long, these denser on the veins abaxially, if the leaves glabrous then at least some gland-tipped trichomes found on margins and abaxial veins; major veins 4–5 pairs, not clearly visible; base truncate to cuneate, only slightly decurrent onto the petiole; margins entire or occasionally with a few shallow lobes, with at least some minute gland-tipped simple trichomes; apex bluntly acute; petioles 0.5–2 cm long, glabrous to densely and finely pubescent, pubescence in parallel with the stems, denser in adaxial groove. Inflorescences arising directly from short shoots that sometimes bear condensed scale-like leaves, with 1–3(5) flowers arising all from the same point on the stem, pubescent like the stems; peduncle absent; pedicels 1.2–2.5 cm long, ca. 1 mm in diameter at the base, ca. 1.1 mm in diameter at the apex, spreading at anthesis, glabrous or finely pubescent with minute simple uniseriate trichomes < 0.3 mm long, articulated at the base; pedicel scars closely spaced in what appears to be a fascicle. Buds ovoid, deeply included in the narrowly conical calyx tube, only halfway exserted just before anthesis. Flowers 5-merous, apparently all perfect. Calyx tube 2–4 mm long, narrowly conical, the lobes 1–2.5 mm long, narrowly triangular or somewhat spathulate, the apex rounded, pubescence like that of the pedicels and stems. Corolla 2–5 cm in diameter, 1–2.7 cm long, pale violet or purple-blue to white with a darker purple centre, stellate, lobed less than halfway to the base, the lobes 10–13 mm long, 5–6 mm wide, narrow and tongue-like with rounded, hooded tips, spreading at anthesis, sparsely pubescent along the lobe midvein adaxially, densely pubescent with minute simple papillae abaxially, these denser on the margins and tips. Stamens equal; filament tube very short, almost absent; free portion of the filaments 2–3.5 mm long, glabrous or sparsely pubescent at the base; anthers 5–5.5 mm long, 1.5–2.5 mm wide, ellipsoid, connivent or slightly spreading, bright yellow, smooth abaxially, the base somewhat sagittate, poricidal at the tips, the pores lengthening to slits with age. Ovary globose, glabrous; style 1–1.2 cm long, glabrous; stigma clavate or capitate, the surface minutely papillose. Fruit a globose berry, ca. 1.5 cm in diameter, reddish orange when mature, the pericarp apparently thin and somewhat shiny; fruiting pedicels ca. 2.5 cm long, ca. 1.5 mm in diameter at the base, pendent or spreading; fruiting calyx lobes appressed and covering the lower third to half of the berry. Seeds 5–10 per berry, ca. 3 mm long, ca. 2.5 mm wide, ovoid reniform, brownish red, the surfaces shallowly pitted, the testal cells sinuate in outline.

**Figure 7.**
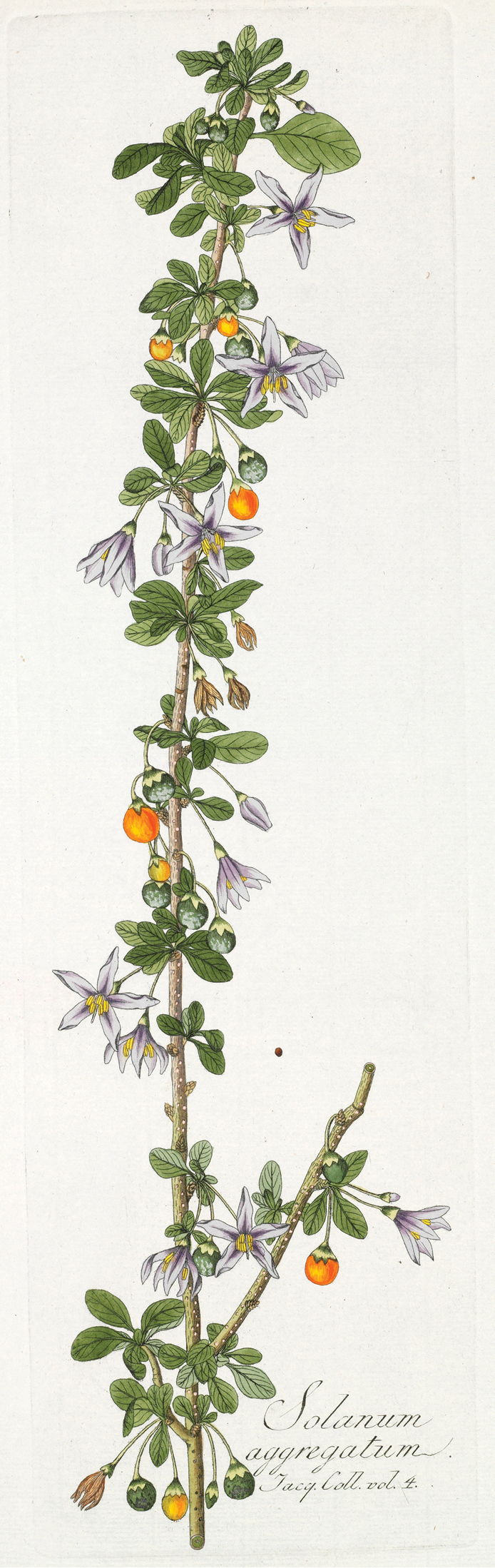
*Solanum guineense* L. Plate 323 from *Icones Plantarum Rariorum* vol. 2 (Jacquin 1792/1793, as *Solanum aggregatum* Jacq.), for dates of publication of Jacquin’s *Icones* see Schubert (1945). Reproduced with permission of the Natural History Museum Library.

##### Distribution

**Figure 8.**
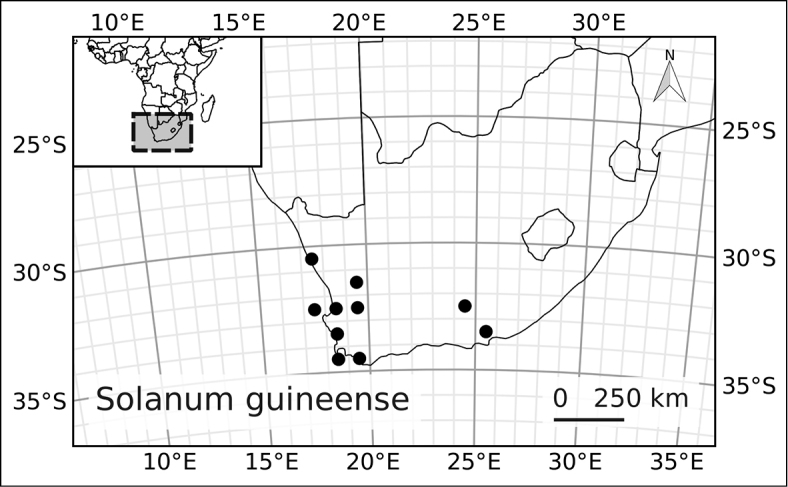
Distribution of *Solanum guineense* L.

(Figure 8). Endemic to the Cape Region of South Africa.

##### Ecology and habitat.

Dunes and strand vegetation near the sea, from sea level to 100 m elevation.

##### Common names and uses.

South Africa: coastal nightshade.

##### Preliminary conservation status

(IUCN 2014). Least Concern (LC). EOO 89,953 km^2^ (LC), AOO 24 km^2^ (EN). *Solanum guineense* has a relatively wide range in South Africa, and our calculated AOO indicating conservation concern is likely due to the number of specimems we have examined for this study. The species occurs in protected areas around the Cape, and although not weedy, appears to be common, judging from the photographic records in the online community nature recording site *iSpot* (http://www.ispotnature.org/communities/southern-africa).

##### Discussion.

*Solanum guineense* is a distinctive species, with sessile inflorescences bearing only a few flowers with campanulate to spreading corolla lobes, fleshy leaves and bright orange fleshy berries (Fig. 7). The only other species with which it could be confused is the sympatric and vegetatively somewhat similar *Solanum africanum*, with branched inflorescences with more, smaller flowers and blackish purple berries. *Solanum africanum* has a winged stem and rhomboid leaves, while *Solanum guineense* has a rounded stem and oblanceolate or obovoid leaves. *Solanum guineense* was long known by the later name *Solanum aggregatum*; it was only in the 1950s that the correct name for this South African taxon began to be used (Heine 1960). The name *Solanum guineense* (L.) Mill. is still occasionally used for the Morelloid clade species correctly called *Solanum scabrum* Mill., the Garden Huckleberry (Edmonds 2012), and is based on the basionym Solanum nigrum var. guineense L.

In describing *Solanum guineense*, Linnaeus apparently copied the locality “Guinea” from Boerhaave (1720). Heine (1960) suggested that Linnaeus used the locality pertaining to Solanum nigrum var. guineense L. rather than to Boerhaave’s “Solanum africanum lignosum, folio atroviridi angusto oblong obtuso” (Boerhaave 1720). He then later tried to correct his mistake (Linneaus 1771) by transferring this morphologically unusual species to the genus *Atropa* L. as *Atropa solanacea* L. Bitter (1917) then used the later name *Solanum aggregatum* for this taxon, and also as the basis for his subgenus *Lyciosolanum*.

The unusual few-flowered inflorescences and flowers with petals that only spread at the tips (or at least when dry looking somewhat campanulate) led Bitter (1917) to erect subgenus *Lyciosolanum*. Seithe (1962) and subsequent authors (D’Arcy 1972) followed this until molecular data showed *Solanum guineense* was nested within the paraphyletic “non-spiny” grade of the larger monophyletic *Solanum* (Bohs 2005).

In describing *Solanum sempervirens*, Philip Miller (1768) cited the Linnaean polynomial for *Solanum guineense* but with a word left out (“Solanum caule inerme fruticoso, foliis [ovatis] integerrrimis, pedunculis lateralibus filiformibus”). He obviously did not know the species from live plants; he does not describe it in English as he did other species in the work. We think he was equating his *Solanum sempervirens* with the plant named *Solanum guineense* with the same polynomial in Linnaeus (1753) and consider the names homotypic.

*Solanum aggregatum* was described from material cultivated in Vienna (Jacquin 1791). We have found no original material, but *Atropa solanacea* is cited in the protologue making this name homotypic with it (and with *Solanum guineense*).

Dunal used two specimens collected by J.F. Drège as the basis for his *Solanum dasypus* (*Drège s.n.*) and *Solanum monticolum* (*Drège “Lycium 7865*”). They represent leaf shape variants of *Solanum guineense*, and the label “Lycium 7865” indicates the difficulties collectors had identifying this as a species of *Solanum*.

No original material has been found for *Solanum bachmannii*, but we are accepting Bitter’s (1917) placement of it as a variant of what he called *Solanum aggregatum*. Dammer (1906) differentiated his new species from *Solanum aggregatum* by its thicker leaves and 4-merous flowers.

##### Specimens examined.

**South Africa. Eastern Cape**: Coernie River, near Enon, Uitenhage Div., *Baur 153* (K); near Graaf Reinet, 1866, *Bolus 51* (BM, K, S); along the Sunday River near Monkey Ford, *Burchell 2885* (G-DC, K, LE); Uitenhage, Redhouse, May 1912, *Rogers s.n.* (BM); in the forests of Adow, and on the banks of the Zwartkop River, district of Uitenhage, *Zeyher 508* (BM, K, NY). **Northern Cape**: Garies, 9 miles NNW of Garies, 24 Sep 1948, *Acocks 14946* (K, S). **Western Cape**: Stikland, between Strand and Pearl Roads, Jul 1932, *Acocks 403* (S); Kanonberg, S spur, overlooking Bottelary Road, 1 Jul 1933, *Acocks 2767* (S); above Victoria Road between Camps Bay and Clifton, 22 May 1935, *Acocks 4561* (S); Nortier Reserve, ca. 8 km N of Lambert’s Bay, 10 Jul 1970, *van Breda 4164* (K); Cape of Good Hope, Cap. Bona Spei (S sheet - ‘Tafelberg [Table Mountain]), *Drège 178* (BM, E, LE, S); Kalk Bay Mountains, at summit R, 7 Apr 1934, *Galpin 12630* (K); Van Rhyns Pass, near top just below TMS system, 15 Oct 1976, *Goldblatt 4326* (K, S); between Hout Bay and Chapman’s Peak, 27 Jun 1945, *Leighton 987* (NY); Paarlberg, 24 Apr 1962, *van den Merwe 996* (K); Atties Bed, (Percy Sladen Memorial Expedition to the Orange River 1910-1911), 1 Dec 1910, *Pillans 5436* (K); Stellenbosch, 20 Aug 1846, *Prior s.n.* (K); Camp’s Bay, 11 May 1847, *Prior s.n.* (K); Piquetberg [Terra Capensis], 24 Jun 1896, *Schlechter 7896* (G x2, K, LE); Klippies Baai, 3419 Caledon AD, Voelklip, Hermanus, 9 Mar 1980, *Williams 192* (K); Kliphuewel, Farm Spieka SE of Klipheuwel, 15 Sep 1982, *van Zyl 3136* (K).

#### Solanum humblotii

Taxon classificationPlantaeSolanalesSolanaceae

Dammer, Bot. Jahrb. Syst. 38: 184. 1906.

Figure 9

##### Type.

Madagascar. Sin. loc., *L. Humblot 509* (type material presumably destroyed at B; lectotype, designated here: P [P00349085]; isolectotypes: K [K000414188], P [P00349086, P00349087, P00349088], W [W-1889-0053791]).

##### Description.

Liana in the subcanopy or canopy or a scandent shrub, to 3 m tall or perhaps larger. Stems flexuous, ribbed, glabrous or pubescent with minute uniseriate dendritic trichomes ca. 0.1 mm long or less, glabrescent; new growth finely and minutely pubescent with dendritic trichomes < 0.1 mm long; bark of older stems longitudinally ridged, often exfoliating or flaking, pale grey to almost white. Sympodial units plurifoliate, the leaves not geminate, evenly distributed along branches. Leaves simple, 5.5–7.5 cm long, 2.5–3 cm wide, elliptic, thickly chartaceous, discolorous, glabrous and smooth on both surfaces except for minute dendritic and simple trichomes along the midrib; major veins 3–6 pairs, spreading at 60°-90° to the midvein, not prominent and the finer venation not visible; base equal or oblique, cuneate to truncate; margins entire, glabrous or with occasional minute dendritic trichomes; apex short-acuminate; petiole 0.6–1 cm long, canaliculate and minutely dendritic-pubescent adaxially. Inflorescences terminal or apparently lateral on long slender main branches, 0–2 cm long, unbranched, with 1–2(4) flowers, glabrous or with a few minute dendritic trichomes; peduncle absent or up to 8 mm long; pedicels 1.8–4.5 cm long, apically dilated, ridged when dry, articulated 0–2 mm from base, sparsely pubescent with trichomes like those on the stem with the branches to 0.15 mm long. Buds ellipsoid, the corolla soon exserted from the calyx tube. Flowers 5-merous, apparently all perfect. Calyx tube ca. 3 mm long, cup-shaped, the lobes 2–4 mm long, 1.5–2 mm wide at base, narrowly triangular to deltate, acute to long-acuminate at the tips, unevenly tearing for up to ca. 2 mm at the sinuses, often tinged purple in live plants (e.g., *Tosh et al. 100*) evenly and sparsely pubescent with minute dendritic trichomes 0.1–0.2 mm long. Corolla 2–2.5 cm in diameter, violet to deep purple with a white centre (fide *Schatz & Villiers 1820*), stellate, lobed almost to base, the 8–10 mm long, 4–5 mm wide, ovate to linear, finely dendritic-pubescent abaxially, the trichomes longer and denser at the margins and tips. Stamens equal; filament tube 0.5–1 mm long; free portion of the filaments ca. 1 mm long; anthers ca. 3.5 mm long, ca. 1.5 mm wide, apparently free but close together, ellipsoid, smooth abaxially, poricidal at the tips, the pores clearly delineated and not lengthening with age. Ovary conical, glabrous; style 9–10 mm long, protruding 3–3.5 mm above the anthers, straight, glabrous; stigma clavate or capitate, minutely papillose. Fruit a globose berry, green when immature; fruiting pedicels not markedly elongating or woody. Seeds not known from mature fruit.

**Figure 9.**
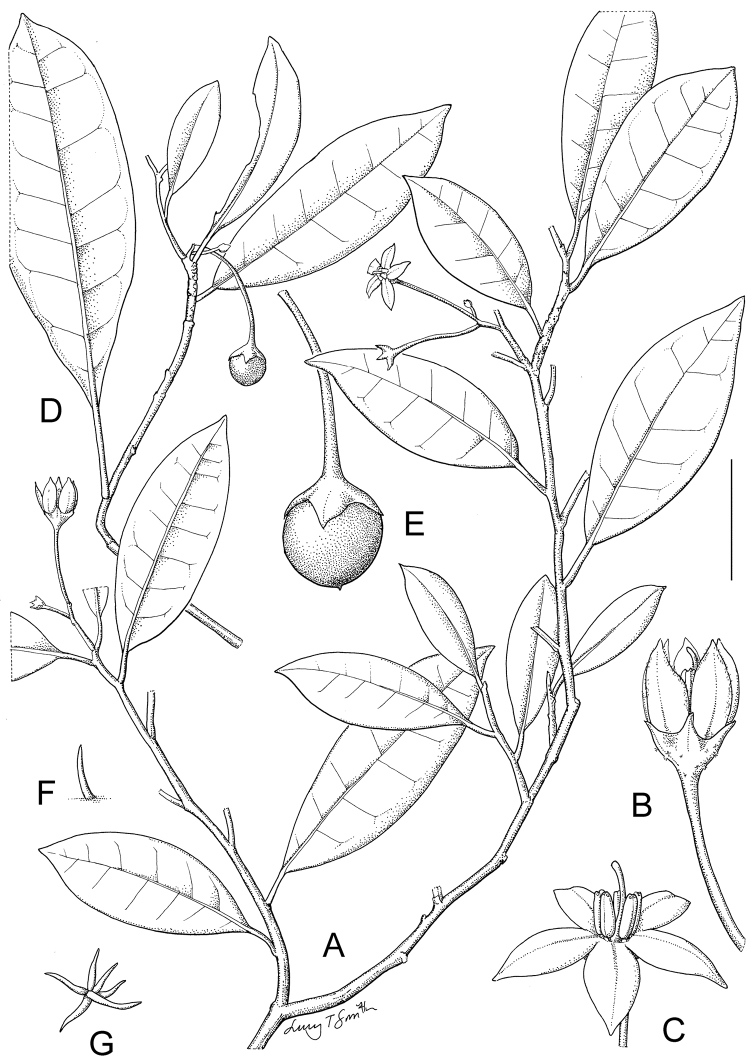
*Solanum humblotii* Dammer. **A** Flowering branch **B** Flower bud **C** Open flower **D** Fruiting branch **E** Berry **F** Simple trichome **G** Dendritic trichome. (based on: **A–C** *Ralisamana et al. 96*; **D–G** *Tosh et al. 100*). Scale bar: **A, D** = 3 cm; **B, C** = 1 cm; **E** = 1.5 cm; **F, G** = 0.2 mm. Drawn by Lucy T. Smith.

##### Distribution

**Figure 10.**
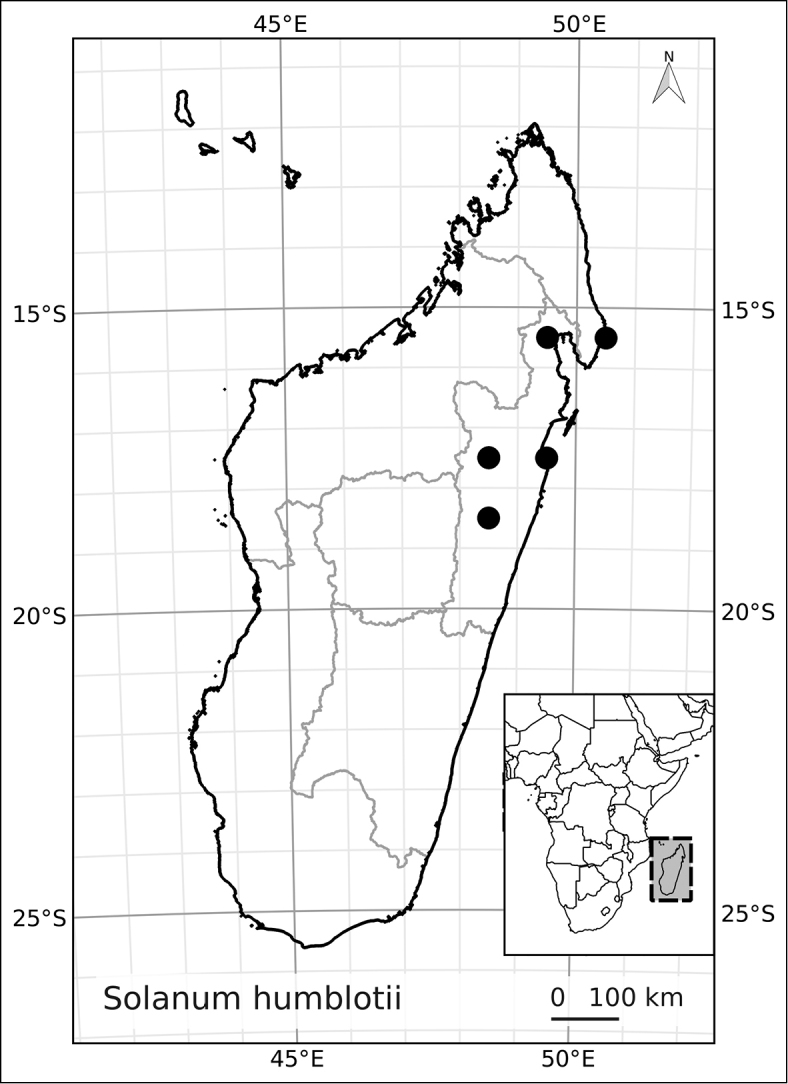
Distribution of *Solanum humblotii* Dammer.

(Figure 10). Endemic to northern Madagascar in the province of Toamasina and on the island of Nosé Mangaby.

##### Ecology and habitat.

Littoral forests on white sand and inland eastern rainforests; sea level to 700 m elevation.

##### Common names and uses.

None recorded.

##### Preliminary conservation status

(IUCN 2014). Near Threatened (NT). EOO 20,941 km^2^ (NT), AOO 40 km^2^ (EN). *Solanum humblotii* is confined to wet forests (see below), and is rarely collected. Given the decreasing extent of wet forests in Madagascar, this species is certainly of conservation concern, but it does occur in or near some forestry reserves, so may have some degree of protection.

##### Discussion.

*Solanum humblotii* is a forest liana with minute dendritic pubescence, triangular to long-triangular calyx lobes and thickly chartaceous discolorous leaves with an apiculate tip. The name *Solanum humblotii* has been mistakenly applied *Solanum truncicola* following the synonymy established by D’Arcy and Rakotozafy (1994), and to *Solanum madagascariense* though misidentification.

The vegetative morphology of *Solanum humblotii* is very similar to some populations of the widespread *Solanum madagascariense*. The two species can be distinguished easily on inflorescence size and calyx morphology. The inflorescence of *Solanum humblotii* has only 2–3 (exceptionally 4) flowers on long filiform pedicels and is never branched, while that of *Solanum madagascariense* is many times branched with many flowers except on some aberrant specimens; calyx lobes are 3–4.5 mm long in *Solanum humblotii*, and up to 1 mm long in *Solanum madagascariense*.

Specimens here recognised as *Solanum truncicola* were treated as *Solanum humblotii* by D’Arcy and Rakotozafy (1994), where the two were treated as synonyms. The two species are similar, and prior to our study *Solanum humblotii* was only known from the type collection. Further collecting in the wet forests of northern Madagascar has resulted in more specimens with which to assess the status of these two taxa (see also discussion under *Solanum truncicola*).

*Solanum humblotii* differs from *Solanum truncicola* by its petioles that are 6–10 mm (versus 1–5 mm) long, acuminate (versus acute) leaves, pubescent (versus glabrous) young stems, and inflorescences with a peduncle up to 5 mm long (versus no peduncle). The calyx of *Solanum humblotii* is fused in bud (Fig. 9); the tube is longer than the lobes and tears for up to 2 mm at anthesis with corolla expansion. The resulting calyx lobes are 3–4.5 mm long, triangular to narrowly triangular and acuminate, chartaceous, and sparsely covered in fine dendritic (almost scurfy) pubescence. This calyx morphology is similar to that of *Solanum imamense*, *Solanum betroka* and *Solanum sambiranense*. The calyx of *Solanum truncicola* is unlike that of any other Madagascar *Solanum*. It is dissected almost to the base in bud and does not tear by more than 1 mm at anthesis; the calyx lobes are linear to narrowly ovate or obovate, membranous, and usually glabrous.

*Solanum humblotii* is restricted to a small area of wet forest in the eastern ecoregion of Madagascar (Humbert 1955; Faramalala 1988, 1995), a habitat like that of the related wet forest climbing species *Solanum madagascariense* and *Solanum trichopetiolatum*. It is sympatric with the morphologically similar *Solanum madagascariense* and occurs considerably further south of *Solanum trichopetiolatum*; the distribution range of the epiphyte *Solanum truncicola* lies directly to the south of *Solanum humblotii* with some overlap in distribution ranges around the Moramanga area.The locality of the type collection within Madagascar is not known, but is likely to be from the region where all other specimens of *Solanum humblotii* have been collected. Dammer (1906) describes it as a shrub but the type specimens contain no such information; modern collections record a variety of growth forms (shrub, liana and epiphyte) in both primary and secondary habitats.

Udo Dammer worked in Berlin and it is likely he used specimens held there to describe *Solanum humblotii* but these are no longer extant. Of the four duplicates of *Humblot 509* in the herbarium in Paris, we have chosen P00349085 as the lectotype due to its numerous flowers and excellent condition.

##### Selected specimens examined.

**Madagascar. Toamasina**: Parc National de Masoala, sur la route d’Ambanizana à Analambolo (25 km N de la ville d’Ambanizana), ca. 6 km NE d’Ambanizana, Fiv. Maroantsetra, 24 Jan 1996, *Aridy et al. 77* (MO, NY); Zahamena, réserve naturelle N°3, 23 Mar 1941, *Decary 16757* (P); Moramanga, Fanovana, forêt orientale, 10 Jul 1942, *Decary 18118* (P); Tampolo Forest Station, ca. 10 km N of Fenerive, 23 Jan 1986, *Dorr et al. 4637* (K, MO, P, TAN, WAG); Tampolo STF, District Fénérive-Est, Canton Ampasina, Tampolo, station forestière de Tampolo, 2 Jul 2001, *Ludovic et al. 110* (G, MO, P); Antandrokonely, lac Alaotra, Jun 1957, *Peltier & Peltier 980* (P); forêt littorale classée de Tampolo, près du village Tanambao-Tampolo, Fenoarivo-Est, 5 Apr 1997, *Ralimanana et al. 96* (MO, TAN); Maroantsetra, Nosy Mangabé, a 520 ha island 5 km from Maroantsetra in the bay of Antongil, 2 Dec 1987, *Schatz & Villiers 1820* (K, MO, P, TAN, WAG); Tampola, 3 km N of Tampola Forestry Reserve, 14 Jan 2006, *Tosh et al. 100* (BR, TAN).

#### Solanum imamense

Taxon classificationPlantaeSolanalesSolanaceae

Dunal, Prodr. [A.P. de Candolle] 13(1): 85. 1852.

**Figure 11.**
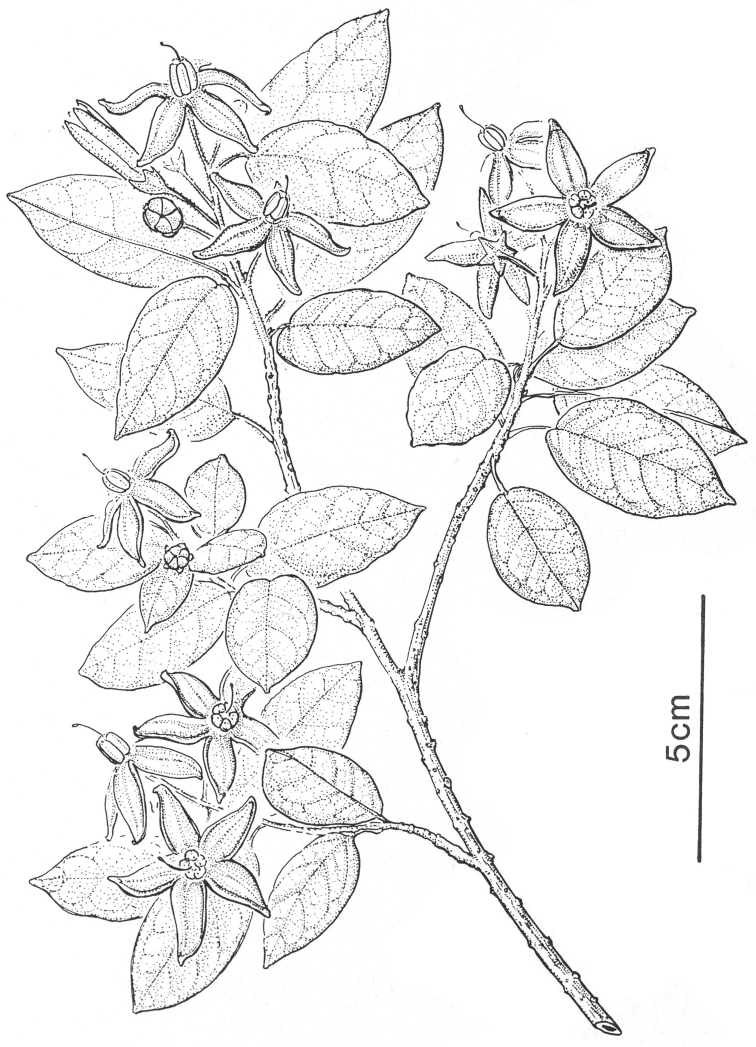
*Solanum imamense* Dunal. Flowering branch (based on *Baron 1754*). Adapted from D’Arcy and Rakotozafy (1994) with permission of Muséum National d’Histoire Naturelle.

Figures 1B , 2B , 11

Solanum imamense Dunal var. grandiflorum Dunal, in DC., Prodr. 13(1): 85. 1852. Type. Madagascar. Antananarivo: “in rupibus provinciae Emirnae [Imerina] Madagascariensis” 1833, *W. Bojer s.n.* (holotype: G-DC [G00144980]; isotypes: W [W0000604], K [K000212345]).
Solanum ankazobe D’Arcy & Rakot., Fl. Madag. Fam. 176: 67. 1994. Type. Madagascar. Antananarivo: Bois sabloneux secs, Ambongo, entre Andranomavo et Itampitso, Sep 1905, *J. Perrier de la Bâthie 9615* (holotype: P [P00349380]; isotype: MO [MO-150887]).

##### Type.

Madagascar. Antananarivo:“croit sur les rocher escarpées dans le d’Imamou”, 1839, *W. Bojer s.n.* (holotype: G-DC [G00144901]).

##### Description.

Shrub or liana, 3-4(+) m tall. Stems flexuous, flattened to terete, sparsely to densely pubescent with densely to loosely branched dendritic trichomes 0.2-0.3 (0.5) mm long, glabrescent; new growth sparsely to more commonly densely pubescent with uniseriate dendritic trichomes to 0.5 mm long, these often drying golden or golden reddish in herbarium specimens; bark of older stems longitudinally ridged, light grey. Sympodial units plurifoliate, the leaves not geminate, on short shoots or somewhat clustered towards tips of branches. Leaves simple, 2.5–5(7) cm long, 1.5–3(4) cm wide, ovate, membranous to chartaceous, discolorous, pubescent on both surfaces with uniseriate dendritic trichomes to 0.5 mm long, sometimes slightly longer and somewhat denser in the axils of the main veins with the midrib; major veins 4–7 pairs, spreading at ca. 60° to the midvein and forming loops, the finer venation often visible; base attenuate or truncate to weakly cordate; margins entire; apex acute to acuminate; petiole slender, 0.5–1.5(2) cm long, pubescent with dendritic trichomes like those of the new growth and leaves. Inflorescences terminal, at the apex of slender terminal or lateral branches, 3–7(10) cm long, furcate (sometimes unbranched), with (4)7–13 flowers, pubescent with dendritic trichomes like those of stems and leaves; peduncle 0.5–2.5 cm long; pedicels 1.1–1.7 cm long, apically dilated, pubescent with trichomes as on the young stem, articulated less than 1 mm from base and tiny pegs; pedicel scars irregularly spaced 1–4 mm apart. Buds ovoid to ellipsoid, the corolla only about halfway exserted from the corolla tube before anthesis. Flowers 5-merous, apparently all perfect. Calyx tube 2–3 mm long, conical, the lobes 3–5 (7.5) mm long, 1–1.5 mm wide at base, deltate to linear, acute at the tips, unequal in size, tearing at the sinuses by ca. 1 mm, densely pubescent with dendritic trichomes like those on the rachis. Corolla 2–3 cm in diameter, violet or purple, lobed almost to base, the lobes 10–15 mm long, 2–6 mm wide, narrowly deltate to linear, puberulous adaxially, minutely pubescent abaxially with dendritic trichomes, these larger at the tips and margins. Stamens equal; filament tube ca. 1 mm long; free portion of the filaments 1–2 mm long, glabrous; anthers 4–5(6) mm long, ca. 2 mm wide, broadly ellipsoid, not connivent, smooth abaxially with some papillae near the pores, poricidal at the tips, the pores much smaller than anther apices, ca. 0.4 mm in diameter, not lengthening with age. Ovary conical, glabrous; style 10–13 mm long, protruding 3–5(7) mm above the anthers, laterally flattened, curved, glabrous; stigma clavate to capitate, dark, the surface smooth. Fruit a globose to elongate pyriform berry, ca. 3 cm long, 1.2–1.6 cm in diameter, apically rounded or the distal portion pointed and elongate, dark purple when mature, the pericarp thin, glabrous; fruiting pedicels 1–2.5 cm long, up to 1.5 mm diameter at base, pendulous, flexible, ridged; fruiting calyx slightly accrescent, the lobes becoming wider and forming a spreading cup around the fruit. Seeds 20–30 per berry, 4–4.5 mm long, 3–3.5 mm wide, flattened reniform, yellowish brown, the surfaces minutely pitted, the testal cells pentagonal or sinuate in outline, the lateral walls with hair-like projections to 0.1 mm long.

##### Distribution

**Figure 12.**
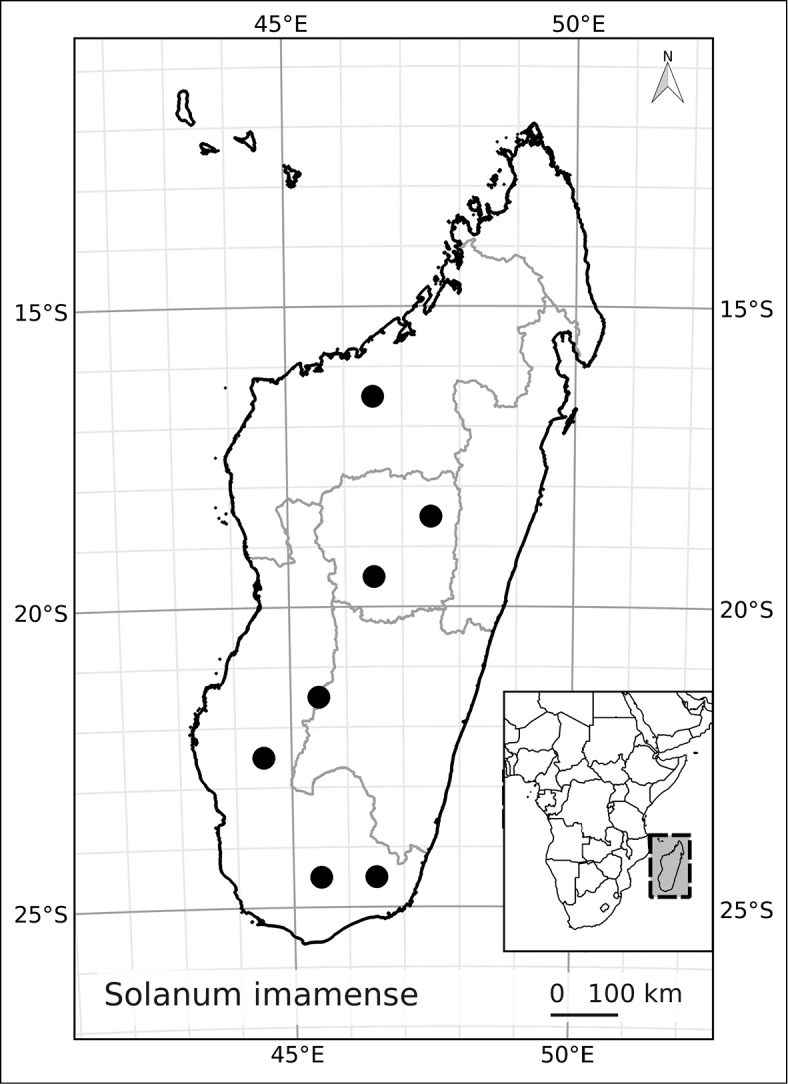
Distribution of *Solanum imamense* Dunal.

(Figure 12). Endemic to Madagascar, with a broad distribution range in the west, the south, and on the high central plateau; in the provinces of Mahajanaga, Antananarivo, and Toliara.

##### Ecology and habitat.

Open dry forest and forest edges; often growing on rocks; 900–1000 m.

##### Common names and uses.

None recorded.

##### Preliminary conservation status

(IUCN 2014). Least Concern (LC). EOO 165,095 km^2^ (LC), AOO 40 km^2^ (EN). In common with other members of the ANS clade in Madagascar, *Solanum imamense* has a relatively wide distribution, resulting in an EOO indicating lack of immediate conservation concern. The paucity of collections, indicative of local rarity, coupled with the ongoing habitat threats in Madagascar, however, do indicate monitoring and further collection to assess local and regional rarity is necessary.

##### Discussion.

*Solanum imamense* is a fairly common liana or shrub with shallowly cordate or truncate leaves clustered towards tips of branches, large (to 3 cm in diameter) violet or purple flowers, and large anthers 4–5 mm long. The pubescence of dendritic trichomes up to 0.3–0.5 mm long is visible with the naked eye on the petioles, pedicels, inflorescences and abaxial sides of the leaves (Figs 1B, 2B).

*Solanum imamense* is morphologically similar and possibly closely related to *Solanum betroka* from southern Madagascar and *Solanum sambiranense* from northern Madagascar, and perhaps also the rare and poorly known *Solanum ivohibe* from the province of Fianarantsoa. *Solanum imamense* differs from *Solanum betroka* in its inflorescences with 7–13 (versus 1–3) flowers, larger floral parts, and leaves that are always entire (versus leaves that are frequently lobed). *Solanum imamense* can be distinguished from *Solanum sambiranense* by its ovate leaves 2.5–5 cm long (versus elliptic to obovate leaves 5–10 cm long) and looser dendritic trichomes 0.2–0.5 mm (versus less than 0.1 mm) long. *Solanum imamense* differs from *Solanum ivohibe* by its ovate leaves less than 5 cm long (versus elliptic to obovate leaves 5–7 cm long), cordate to short attenuate (versus long attenuate) leaf base, and calyx lobes more than 2 mm (versus less than 2 mm) long.

*Solanum imamense* has a broad ecological profile occurring occasionally in dry to mesic parts of the island in the western, central, and southern ecoregions (Humbert 1955; Faramalala 1988, 1995). In the southern part of its distribution range it is sympatric with *Solanum betroka* and in the northern part of the range with *Solanum sambiranense*.

Solanum imamense var. grandiflorum was accepted by Bitter (1917) but synonymised with *Solanum imamense* by D’Arcy and Rakotozafy (1994). It differs from the type variety by flower size and pubescence density, but the variation in both characters is continuous. The type specimens of both were collected by W. Bojer in central Madagascar. *Solanum ankazobe* was described on the basis of three collections, and was distinguished by its small leaves, smaller calyx tube, irregular tearing of the calyx and wider ovary. The type collection of *Solanum ankazobe* (*Perrier de la Bâthie 9615*) has leaves that fall within the range of variation in *Solanum imamense*, while other specimens cited have smaller floral parts (*Perrier de la Bâthie 9577* and *9579*). We consider these specimens to fall within the range of variation represented in *Solanum imamense* and treat *Solanum ankazobe* as a synonym.

##### Selected specimens examined.

**Madagascar. Antananarivo**: Antananarivo-Nord, Tananarive, environs de Tananarive (Anositavo), 22 Apr 1928, *Decary 6276* (K, MO, P); Manerinerina, Tampoketsa d’Ankazobe, 3 Jan 1942, *Decary 17170* MO, (P); Betafo, s.d., *Perrier de la Bâthie 9574* (MO, P); Ankazobe, Manankazo, Tangeotseha entre Ihopa et Behibotra, Dec 1925, *Perrier de la Bâthie 16830* (P). **Mahajanga**: Marovoay, Anjiafitatra, près du MontTsialondraina (Boïna), Nov 1901, *Perrier de la Bâthie 1332* (MO, P); Ambongo, entre Andranomavo et Itampitso, Sep 1905, *Perrier de la Bâthie 9615* (MO, P). **Toliara**: Amboasary-Sud, Amboahangy, bassin supérieur du Mandrare (Sud-Est), Mont Amboahangy prés d’Esira, 25 Nov 1928, *Humbert 6812* (MO, P); Sakaraha, forêt d’Analafanja au N du Fiherenana, Mar 1934, *Humbert 14290* (P); Atsimo-Andrefana, Behetaheta, district et commume rural Beroroha, Fokotany, Beronono-Makay, 17 Jan 2010, *Rakotovao et al. 5128* (MO); Bekily, Ampandrandava, entre Bekily et Tsivory, 1943, *Seyrig 156* (MO, P).

#### Solanum ivohibe

Taxon classificationPlantaeSolanalesSolanaceae

D’Arcy & Rakot., Fl. Madag., Fam. 176: 97. 1994.

Figure 13

##### Type.

Madagascar. Fianarantsoa: Razafinarakoto, Antambohobe, Ivohibe, 28 Nov 1951, *Reserves Naturelles 3516* (lectotype, designated here: P [P00349043]; isolectotypes: P [P00349042], TAN).

##### Description.

Straggling shrub or occasionally a liana, ca. 2.5 m tall. Stems long and flexuous, ridged, glabrous except youngest stems unevenly pubescent; trichomes densely dendritic to echinoid, most only to 0.2 mm long, with 10–15 highly congested branches each; main stems 2–4 mm in diameter, glabrous; bark longitudinally ridged, almost white; leaf scars prominent stumps almost overlapping to 2.5 cm apart. Sympodial units plurifoliate, the leaves not geminate, the leaves clustered at tips of branches and on short shoots. Leaves simple, 5–7 cm long, 2–2.5 cm wide, elliptic to obovate, membranous, wrinkled in herbarium specimens, concolorous, glabrous on both sides except for occasional simple or dendritic trichomes ca. 3 mm long with poorly developed branches, some more densely branched dendritic trichomes on the abaxial side of the midvein; midvein raised abaxially and flat adaxially; major veins 5–6 pairs, spreading at ca. 60° to the midvein, the finer venation brown, fine and faint, with tufts of minute dendritic trichomes with few branches (domatia) in the junction between the midvein and major veins abaxially; base long-attenuate; margin entire, mostly glabrous with occasional simple or sparsely dendritic trichomes becoming more frequent towards the apex; apex acute; petiole 1–2.5 cm long, slender, flexuous, ridged, glabrous or with occasional dendritic trichomes like those on the stem;. Inflorescences terminal, at the apex of branches or short shoots, 4–5 cm long, furcate, with 10–16 flowers; peduncle 2–2.7(5) cm long; peduncle and rachis glabrous or with occasional dendritic trichomes like those on the stem; pedicels 1–2.2 cm long, apically dilated with gradual transition between the pedicel and the calyx, articulated 0–0.5 mm from base, glabrous, the pedicel scars small stumps almost overlapping to 4 mm apart. Buds ellipsoid, the corolla long-exserted from the calyx tube before anthesis. Flowers 5-merous, apparently all perfect. Calyx tube ca. 1.5 mm long, broadly cup-shaped, the lobes 0.8–2 mm long, 1–1.5 mm wide at base, deltate, acute to obtuse at the tips, glabrous with tufts of dendritic trichomes at the tips. Corolla 1–2 cm in diameter, violet, stellate, lobed almost to base, the lobes 5–10 mm long, 2–4 mm wide, narrowly ovate, glabrous in the centre of both surfaces with small simple papillate trichomes denser and longer on the margins and tip, the tips of the lobes enlarged and knob-like, perhaps fleshy in live plants. Stamens equal; filament tube ca. 1 mm; free portion of the filaments less than 1 mm long, glabrous; anthers 3.5–4 mm long, ca. 1–1.5 mm wide, broadly ellipsoid, free and clearly separated from one another, smooth abaxially, poricidal at the tips; the pores much smaller than anther apices, ca. 0.3 mm in diameter, not lengthening with age. Ovary conical, glabrous; style 0.8–1 cm long, protruding 3–4 mm above the anthers, slender, curved, glabrous; stigma capitate or distinctly bi-lobed, dark, minutely papillose. Fruit and seeds not known.

**Figure 13.**
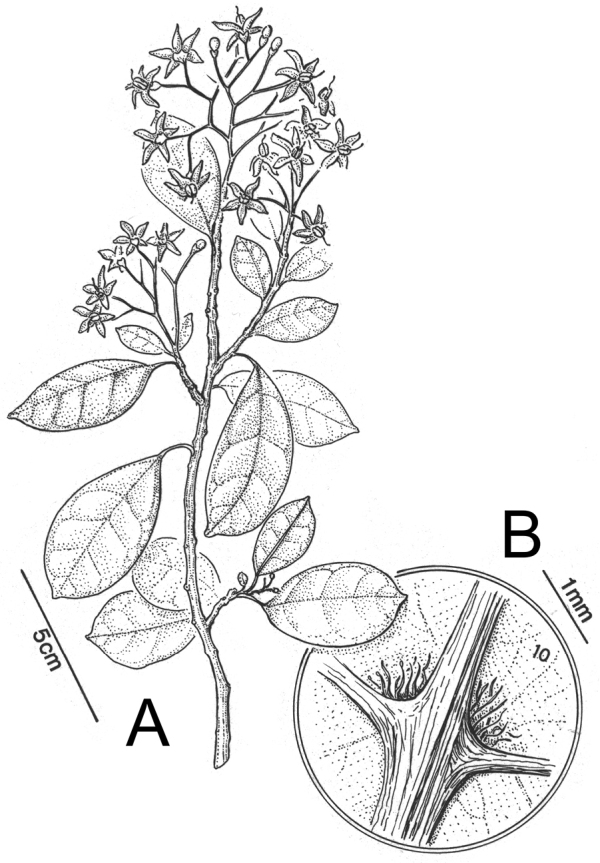
*Solanum ivohibe* D’Arcy & Rakot. **A** Flowering branch **B** Tufts of trichomes in vein axils on leaf undersides (domatia) (based on *Humbert 13429*). Adapted from D’Arcy and Rakotozafy (1994) with permission of Muséum National d’Histoire Naturelle.

##### Distribution

**Figure 14.**
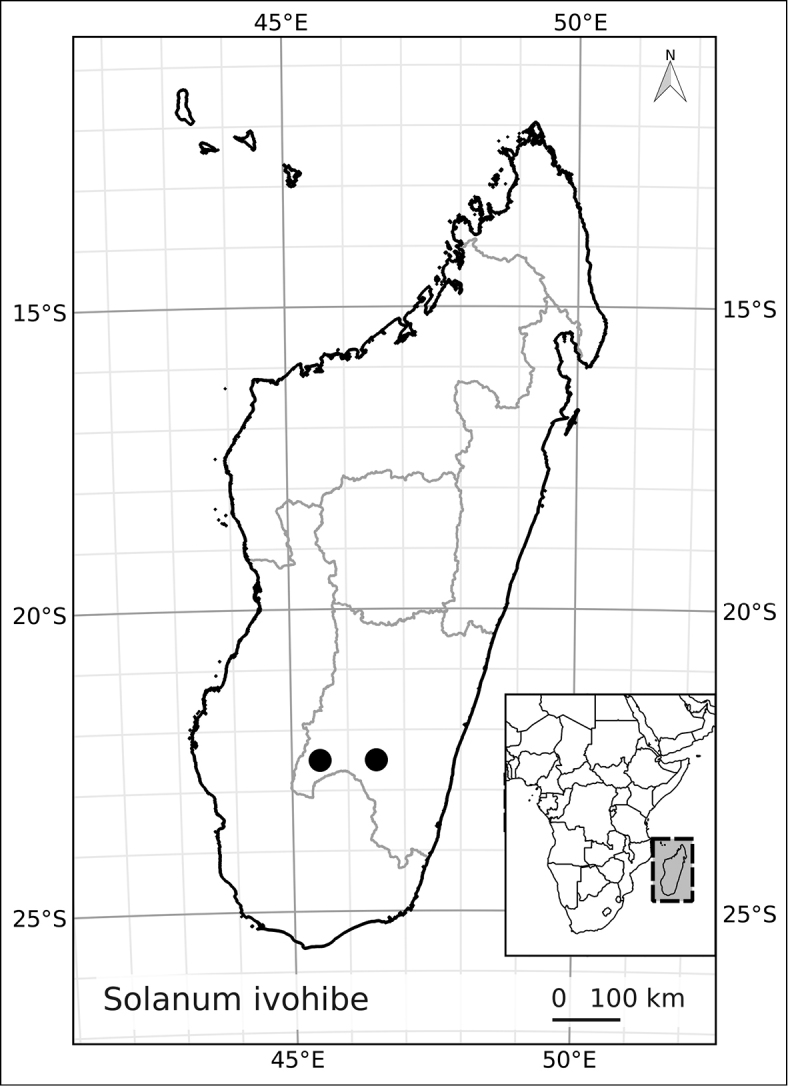
Distribution of *Solanum ivohibe* D’Arcy & Rakot.

(Figure 14). Endemic to Madgascar in the Ivohibe and Andringitra areas of Fianarantsoa province.

##### Ecology and habitat.

Riparian and secondary montane forests from approximately 800 to1200 m elevation.

##### Common names and uses.

None recorded.

##### Preliminary conservation status

(IUCN 2014). Endangered (EN B1a, b iii). EOO 950 km^2^ (EN), AOO 12 km^2^ (EN). Known collections of *Solanum ivohibe* are currently not sufficient to document its range of occurence with any degree of confidence, but its occurrence in the fragmented wet forests of eastern Madagascar and the paucity of collections leads us to give it a preliminary status that indicates conservation concern.

##### Discussion.

*Solanum ivohibe* is a slender forest shrub with almost glabrous leaves on decurrent long petioles, prominent domatia on leaf undersides (Fig. 13 inset), inflorescences with a long peduncle and 10–16 flowers. It seems likely that *Solanum ivohibe* can grow as a liana. The very few collections suggest it grows along streams in mid-elevation mesic to wet secondary forests in the underexplored and exceptionally diverse area around Andringitra National Park (Lewis et al. 1996).

Within Madagascar *Solanum ivohibe* is most similar to *Solanum sambiranense*, and to some extent also with *Solanum imamense* and *Solanum betroka*. *Solanum ivohibe* can be distinguished from *Solanum sambiranense* by its inflorescence bearing 10–16 (versus 3–10) flowers, calyx lobes 0.8–2 mm long tearing for up to 1 mm (versus calyx lobes 4–6 mm long tearing for up to 2 mm), and minute versus larger tufts of trichomes (domatia) in the axils of the veins and midrib of the lower leaf surfaces; it also occurs further south than the distribution range of *Solanum sambiranense*. *Solanum ivohibe* is sympatric with *Solanum imamense* and *Solanum betroka*; it differs from these two species by its long-decurrent (versus cuneate) leaf bases, larger leaves (> 5 cm long versus shorter than 5 cm), smaller calyx, and calyx lobes < 2 mm long (versus usually > 2 mm long). D’Arcy and Rakotozafy (1994) considered *Solanum ivohibe* to be a relative of the Mayotte endemic *Solanum macrothyrsum* but with smaller leaves and inflorescences; it shares features with both *Solanum macrothyrsum* and *Solanum sambiranense*. The anthers of *Solanum ivohibe* are longer than those *Solanum macrothyrsum* (3.5–4 mm versus 2.5–3 mm), the calyx lobes are somewhat longer (0.8–2 mm versus under 0.5 mm) and more deeply divided.

*Solanum ivohibe* appears to occupy a somewhat higher elevation, higher moisture, and more closed canopy environment than the similar species *Solanum betroka* in the arid south, *Solanum sambiranense* in fairly dry north-west, and *Solanum imamense* in mesic niches, and the distribution range of *Solanum ivohibe* does not overlap with these species. *Solanum ivohibe* apprears to be sympatric with *Solanum madagascariense* around Andringitra National Park and the surrounding forests although *Solanum madagascariense* occurs in wetter forest.

The species concept of *Solanum ivohibe* adopted here has little in common with that of D’Arcy and Rakotozafy (1994). The protologue lists five collections of *Solanum ivohibe* and places the species in section *Lemurisolanum*. In our view, two of the cited collections represent other taxa: *Humbert 13429* (P00349045, from Ankazondrana), that was used for the illustration in the original description, belongs to our circumscription of *Solanum madagascariense* and *Peltier & Peltier 980* (P00349044, from Toamasina) belongs to our circumscription of *Solanum humblotii*. The type specimen, however, represents a distinct taxon and we recognise *Solanum ivohibe* here, with a narrow circumscription encompassing the type and two other collections (one cited by D’Arcy and Rakotozafy [1994]) from very near the type locality.

The protologue of *Solanum ivohibe* cites a holotype at P. There are in fact two duplicates, one of which (P00349043) is clearly marked as such in D’Arcy’s handwriting; we select this specimen here as the lectotype of *Solanum ivohibe*.

##### Specimens examined.

**Madagascar. Fianarantsoa**: Ambalavao, Andringitra, edge of Zomandao River, 15 Nov 2010, *Rakotonasolo & Cribb RNF*-*1674* (K); District d’Ivohibe R.N.V., 28 Nov 1951, *Réserves Naturelles Madagascar 3523*-*RN* (P).

#### Solanum macrothyrsum

Taxon classificationPlantaeSolanalesSolanaceae

Dammer, Bot. Jahrb. Syst. 38: 185. 1906.

Figure 15

##### Type.

Mayotte [French Overseas Department]: Maore (Grande Terre), forêt de Combani, 6 Nov 1884, *L. Humblot 387* (second stage lectotype, designated here; first stage designated by D’Arcy & Rakotozafy 1994, pg. 100: P [P00184304]; isolectotypes: BM [BM000887183], P [P00184305], P [P00184306], P [P00184307], K [K000414185], W [W1889-0038295]).

##### Description.

Liana of forest canopy, height unknown. Stems flexuous, terete, glabrous, green; new growth glabrous or minutely papillate, green; bark of older stems smooth, light grey. Sympodial units plurifoliate, the leaves not geminate, clustered at tips of branches. Leaves simple, (5)6–8 cm long, 3–4(4.5) cm wide, oblong to obovate, membranous to chartaceous, concolorous, glabrous on both surfaces, abaxially sometimes with isolated simple to dendritic trichomes along the midvein and with tufts of tangled simple or dendritic uniseriate trichomes in the axils of the main veins, the lamina texture somewhat altered under the tufts; major veins 4–6 pairs, prominent, spreading at ca. 40° to the midvein, the finer venation faint; base attenuate and decurrent onto the petiole; margins entire; apex acute to apiculate; petiole 1.5–4 cm long, slender, canaliculate, glabrous. Inflorescences terminal, at the apex of a long slender flexuous branch, 7–10 cm long, several times branched, with 15–50 flowers, glabrous; peduncle 4.5–7 cm; pedicels apically dilated, 0.5–0.8 cm long, glabrous except for the occasional simple or sparsely dendritic trichomes just below the articulation, articulated 0–0.5 mm from base; pedicel scars 1.5–8 mm apart, becoming closer together distally, somewhat peg-like. Buds globose, the corolla soon exserted from the calyx tube before anthesis. Flowers 5-merous, apparently all perfect. Calyx tube 1.5–2 mm long, broadly cup-shaped, the lobes ca. 0.5 mm long, 1–1.5 mm wide at base, broadly deltate, rounded to cuspidate at the tips, the sinuses scarious, glabrous with tufts of translucent simple hairs at the tips and sometimes extending along the margins. Corolla 1–2 cm in diameter, mauve to lilac or white with purple towards the centre, stellate, lobed almost to base, the lobes 6–12 mm long, 1.5–4 mm wide, narrowly ovate to obovate, cucullate, glabrous adaxially, with simple to dendritic trichomes sparsely distributed abaxially, denser towards the tips, the margins densely papillate with translucent-violet trichomes ca. 0.1 mm long. Stamens equal; filament tube ca. 1 mm; free portion of the filaments 1–1.5 mm long, glabrous; anthers 2.5–3 mm long, 1.5–2 mm wide, broadly ellipsoid, spreading or very loosely connivent, smooth abaxially, poricidal at the tips, the pores much smaller than anther apices, ca. 0.4 mm in diameter, clearly delineated and not lengthening with age. Ovary conical, glabrous; style 6–9 mm long, protruding 2–3 mm beyond the anthers, slightly curved, glabrous; stigma capitate, minutely papillose. Fruits and seeds not known.

**Figure 15.**
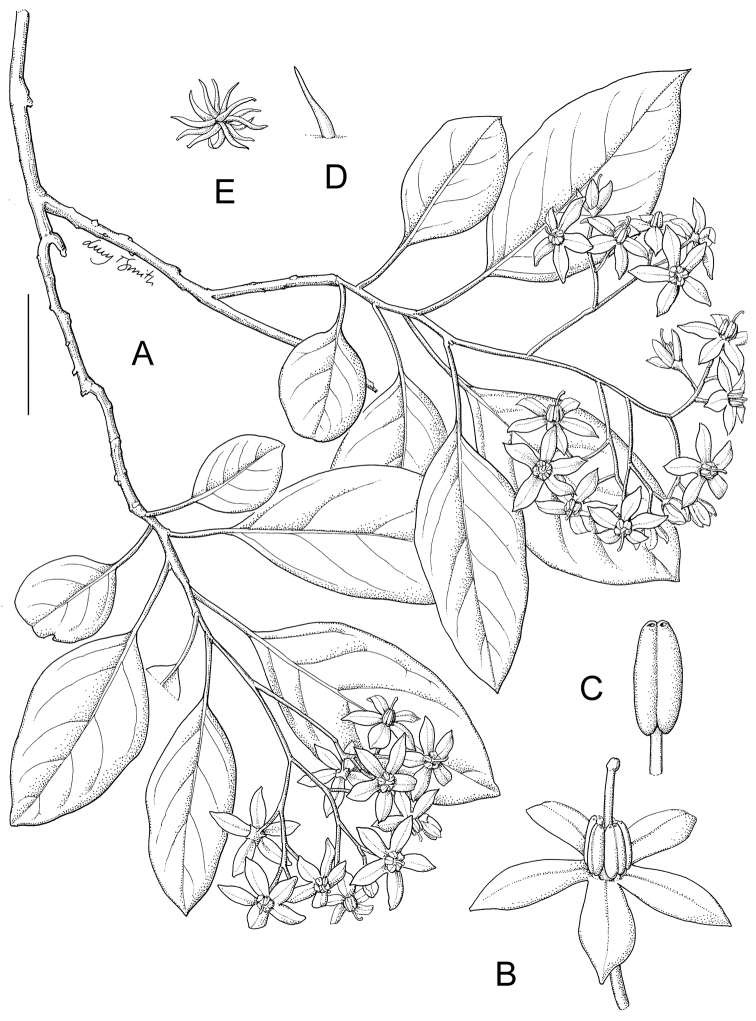
*Solanum macrothyrsum* Dammer. **A** Flowering branch **B** Open flower **C** Anther, showing round, clearly delineated pores **D** Simple trichome **E** Dendritic trichome. (based on: **A–E** *Barthelat et al. 559*). Scale bar: **A** = 2.5 cm; **B** = 7 mm; **C** = 3.3 mm; **D, E** = 0.3 mm. Drawn by Lucy T. Smith.

##### Distribution

**Figure 16.**
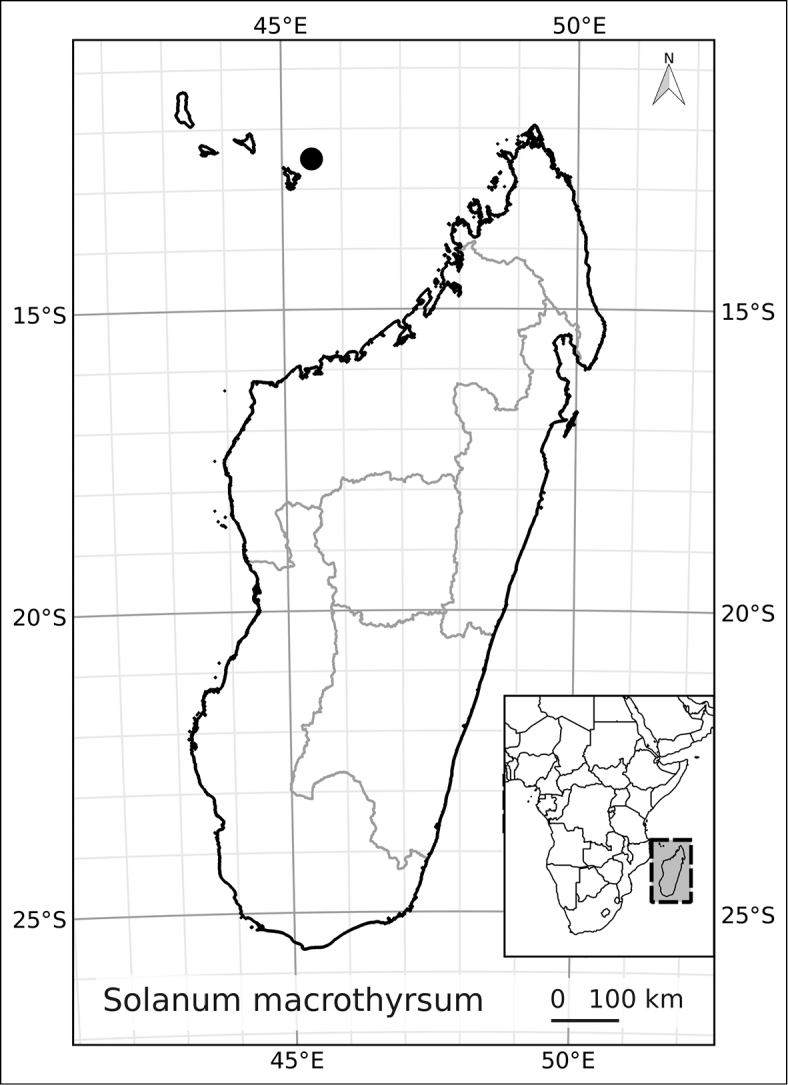
Distribution of *Solanum macrothyrsum* Dammer.

(Figure 16). Endemic to the island(s) of Mayotte (a political department of France geographically situated in the Comoro Islands); only recorded from the largest island Maore (=Mayotte or Grande-Terre).

##### Ecology and habitat.

Wet forests; 200 to 400 m elevation (calculated from locality information; elevation not recorded exactly on any specimens we have seen).

##### Common names and uses.

None recorded.

##### Preliminary conservation status

(IUCN 2014). Data Deficient (DD). Known from only two collections on the island of Mayotte collected 100 years apart (see below) with insufficient habitat information to assess its distribution. It is likely to be endangered based on its occurrence on a single small island and the paucity of collections; however, the island is not well-collected, so re-collection of this plant is a priority.

##### Discussion.

*Solanum macrothyrsum* is a rare liana endemic to Mayotte. It has large bright inflorescences of 15–50 white or violet flowers, large, membranous leaves on long petioles, a very small calyx (less than 1/8 of the corolla length at anthesis) and noticeably short, plump anthers (2.5–3 × 1.5–2 mm) on comparatively long filaments (1–1.5 mm). *Solanum macrothyrsum* is unusual in the group in having little pubescence on its vegetative parts, except some isolated trichomes on abaxial side of the midvein forming tufts in the vein axils (domatia) (see Fig. 15). The four duplicates of the type collection show considerable variation in leaf size, leaf thickness and venation; this perhaps represents leaves growing in the sun and in the shade or the type could have been gathered from several plants.

Since its original description by Dammer (1906), *Solanum macrothyrsum* been been known only from the type collection. It was rediscovered on the main island of Mayotte, Maore, in 2001 (*Barthelat & Ali Sifari 559*).

There are no species of *Solanum* on Mayotte similar to *Solanum macrothyrsum*. The species that most resembles *Solanum macrothyrsum* is *Solanum ivohibe* from eastern Madagascar: they share long petioles, decurrent leaf bases and branched inflorescences with a long peduncle. The two taxa are clearly distinct. *Solanum macrothyrsum* has anthers 2.5–3 mm (versus 3.5–4 mm) long, inflorescences branching 2–3 times (versus branching once), 15–50 (versus 10–16) flowers per inflorescence, peduncle 4.5–7 cm (versus 2–2.7 cm) long, and calyx lobes up to 0.5 mm long (versus 0.8–2 mm long). *Solanum madagascariense* is the only species with short anthers and open, lax inflorescences like *Solanum macrothyrsum*. *Solanum macrothyrsum* differs from *Solanum madagascariense* in its membranous (versus thick chartaceous to coriaceous) leaves, smaller leaf length to width ratio, petiole 1.5–4 cm (versus 0.4–2 cm) long, and smaller calyx, less than 1/8 (versus 1/6–1/3) of corolla length at anthesis.

Edmonds (2012), following the suggestion of R.N. Lester (pers. comm.) and Jaeger (1985) suggested that *Solanum macrothyrsum* was synonymous with *Solanum benderianum* (here treated as a synonym of *Solanum runsoriense*) from Ethiopia, Uganda and Kenya. Bitter (1917) also placed *Solanum macrothyrsum* as a relative of the *Solanum terminale* alliance (his section *Afrosolanum*) rather than with the Madagascar endemic species. There are numerous differences between these taxa: the calyx lobes of *Solanum macrothyrsum* are less than 0.5 mm long while the calyx lobes of *Solanum benderianum* are 2–4 mm long; the petioles of *Solanum macrothyrsum* are often longer than ½ of the leaf length while the petioles of *Solanum runsoriense* are less than 1/3 of the leaf length. In addition, the anthers of *Solanum runsoriense* have pores that elongate to lateral slits following dehiscence, while *Solanum macrothyrsum*, like the Malagasy members of this group, has anthers with distinct pores that never elongate. *Solanum terminale* also occurs in the Comoro Islands, but is known there from only a few collections (see discussion and specimens examined for *Solanum terminale*). *Solanum macrothyrsum* differs from *Solanum terminale* in its anthers that never become longitudinally dehiscent, its more openly branched inflorescences with widely spaced flowers, and its tufts of trichomes in the axils of the main leaf veins on the abaxial surfaces.

Bitter (1917) cites duplicates of type collection from B and P. Dammer almost certainly worked from the B duplicate which is now destroyed. D’Arcy and Rakotozafy (1994) stated that the lectotype is held at P but do not mention which of the four duplicates they chose. Of these one marked in pen as lectotype, two marked as isolectotypes and one is not annotated with a type label. We have selected the duplicate (P00184304) annotated as “lectotype” (although it is not in D’Arcy’s handwriting) as the second stage lectotype for *Solanum macrothyrsum*. Edmonds (2014) was apparently unaware of this lectotypification when she designated as “lectotype” a Kew duplicate of *Humblot 387*; her lectotypification is superfluous, despite D’Arcy and Rakotozafy (1994) not having stated “designated here” and identifying a particular sheet.

##### Specimens examined.

**Mayotte (French Overseas Department). Maore**: Grande Terre, Tsararano, Reserve Forestière de Tchaourembo, 20 Oct 2001, *Barthelat & Ali Sifari 559* (G, K, MO, P).

#### Solanum madagascariense

Taxon classificationPlantaeSolanalesSolanaceae

Dunal, Prodr. [A.P. de Candolle] 13(1): 99. 1852.

Figures 1C , 17

Solanum nitens Baker, J. Bot. 20: 220. 1882. Type. Madagascar. Fianarantsoa: “chiefly in Betsileo-land”, received in K July 1880, *R. Baron 145* (holotype: K [K000414183]; isotypes: E [E00193275], P [P00348975]).
Solanum apocynifolium Baker, J. Linn. Soc. 20: 213. 1883. Type. Madagascar. “Central Madagascar”, Oct 1882, *R. Baron 1767* (holotype: K [K000414194]; isotype E [E00193276]).
Solanum madagascariense Dammer, Bot. Jahrb. Syst. 38: 184. 1906. nom. illeg., non Solanum madagascariense Dunal, 1852. Type. Madagascar. Fianarantsoa: Ivohimanitra forest, Nov 1894, *C.I. Forsyth-Major 15* (lectotype, designated here: K [K000414184]; probable isolectotype: BM [BM000887181]).
Solanum clerodendroides Hutch. & Dalziel, Fl. W. Trop. Afr. 2: 206. 1931. Type. “Southern Nigeria, Eket District” [clearly incorrectly labelled], 1912-1913, *Mr. & Mrs. P.A. Talbot 3211* (holotype: K [K000414057]).
Solanum antalaha D’Arcy & Rakot., Fl. Madag., Fam. 176: 68. 1994. Type. Madagascar. Antsiranana: R.N-II, Ambohitsalonana, district Antalaha, 23 August 1950, *Reserves Naturelles Madagascar [Zaty] 2738* (lectotype, designated here: P [P00349362]; isolectotypes: P [P00346372], MO [MO-277611], MO [MO-277612]).
Solanum marojejy D’Arcy & Rakot., Fl. Madag., Fam. 176: 107. 1994. Type. Madagascar. Antsiranana: Reserve Naturelle de Marojejy; E-facing slope the Manantenina River, N of Mandena, 14°27’S 49°47’E, 230-550 m, 21 October 1989, *J.S. Miller & A. Randrianasolo 4329* (holotype: MO [MO-150888]; isotypes: AD [AD-99109143], K [K000212283], P [P00352676], PRE [n.v., fide D’Arcy & Rakotozafy 1994], TAN [TAN000700], WAG [n.v., fide D’Arcy & Rakotozafy 1994]).
Solanum madagascariense Dunal var. nitens (Baker) D’Arcy & Rakot., Fl. Madag., Fam. 176: 105. 1994. Type. Based on Solanum nitens Baker.

##### Type.

Madgascar. “In Madagascar”, *Collector Unknown* (holotype: G-DC [G00144951]).

##### Description.

Liana or small tree (*Gentry 11852*) to 8 m. Stems terete, flattened or faintly ridged, glabrous to sparsely puberulent with simple unicellular papillae ca. 0.05 mm long, or variously pubescent with a mixture of uniseriate dendritic or arachnoid trichomes to 0.5 mm long, glabrescent or the pubescence less dense with age; new growth glabrous or pubescent with dendritic or arachnoid trichomes like those of the stems; bark of older stems smooth or longitudinally ridged, reddish brown, brown, or yellowish brown, somewhat corky on stems > 1 cm in diameter. Sympodial units plurifoliate, the leaves not geminate, evenly distributed along young branches. Leaves simple, (1.5) 3–9 (12) cm long, (1.2) 2–3 (4.5) cm wide, obovate to oblong, thick-chartaceous to thin-coriaceous, often shiny, concolorous to somewhat discolorous, glabrous, sometimes with dendritic trichomes on the midvein on both sides of the lamina; major veins 5–12 pairs, spreading at 60–90° to the midvein and forming loops, the finer venation usually faint or not visible; base cuneate to truncate, sometimes attenuate; margins entire, rarely lobed with a single lobe basally on each side, the lobes up to 8 mm long, rounded, with shallow sinuses; apex acute to acuminate, rarely obtuse and apiculate; petiole 0.4–2 cm long, canaliculate, usually glabrous, less often finely dendritic-pubescent, flexuous and sometimes twining around supports (e.g., *Humbert 31597*, *Miller & Randrianasolo 4339*). Inflorescences terminal at the apex of branches, (2.5)5–20(25) cm long, furcate or several times branched, with (8)15–45(100) flowers, glabrous or with variable dendritic pubescence paralleling that of the stems; peduncle 1.5–5 cm long; pedicels 0.4–1.1 cm long, apically dilated, always glabrous, when the rachis is pubescent a clear boundary line visible between the glabrous petiole and the pubescent rachis (a few trichomes occasionally found at the pedicel base) , articulated 0–2 mm from base; pedicel scars irregularly spaced 1–4 mm apart, often appearing as apically dilated pegs. Buds globose or broadly ellipsoid, the corolla soon exserted from the calyx tube. Flowers 5-merous, apparently all perfect. Calyx tube 2–3 mm long, broadly cup-shaped or conical, the lobes up to 1 mm long, 1.5–2 mm wide at base, often irregular or almost absent, broadly deltate, rounded to cuspidate at the tips, glabrous or with sparse short simple trichomes; margin often thickened, with dense tufts of simple hairs at the tips. Corolla 1–2 cm in diameter, white to violet, stellate, lobed almost to base, the lobes 4–10 mm long 1.5–2.5 mm wide, narrowly deltate to linear, sometimes aristate with a puberulous appendage ca. 0.5 mm long arising from the adaxial surface of the lobe just below the apex, glabrous adaxially, glabrous to puberulous abaxially with dendritic trichomes that are longer at the lobe tips. Stamens equal; filament tube 0.5–1 mm; free portion of the filaments 1–2 mm; anthers 2.5–4 mm long, ca. 1.5 mm wide, broadly ellipsoid, usually somewhat connivent, smooth or papillose abaxially, poricidal at the tips, the pores about the same diameter as the anther apices, clearly delineated and not lengthening with age. Ovary conical, glabrous; style 6–12 mm long, protruding 1.5–4 mm beyond the anthers, straight or curved, glabrous; stigma clavate to capitate, the surface smooth to minutely papillose. Fruit a globose berry, sometimes ellipsoid, 0.5–1.2 cm diameter, black or purplish black at maturity, the pericarp thin, collapsing on drying to reveal the outline of the seeds, glabrous; fruiting pedicels 1–1.2 cm long, 0.5–0.7 mm diameter at base, pendent to spreading; fruiting calyx slightly accrescent, the lobes becoming woody with a light-coloured margin. Seeds 20–40(+) per berry, 1.5–4 mm long, 1–2.5 mm wide, ovoid reniform or somewhat flattened, golden-orange or reddish brown, the surface deeply pitted, the testal cells pentagonal or slightly sinuate in outline.

**Figure 17.**
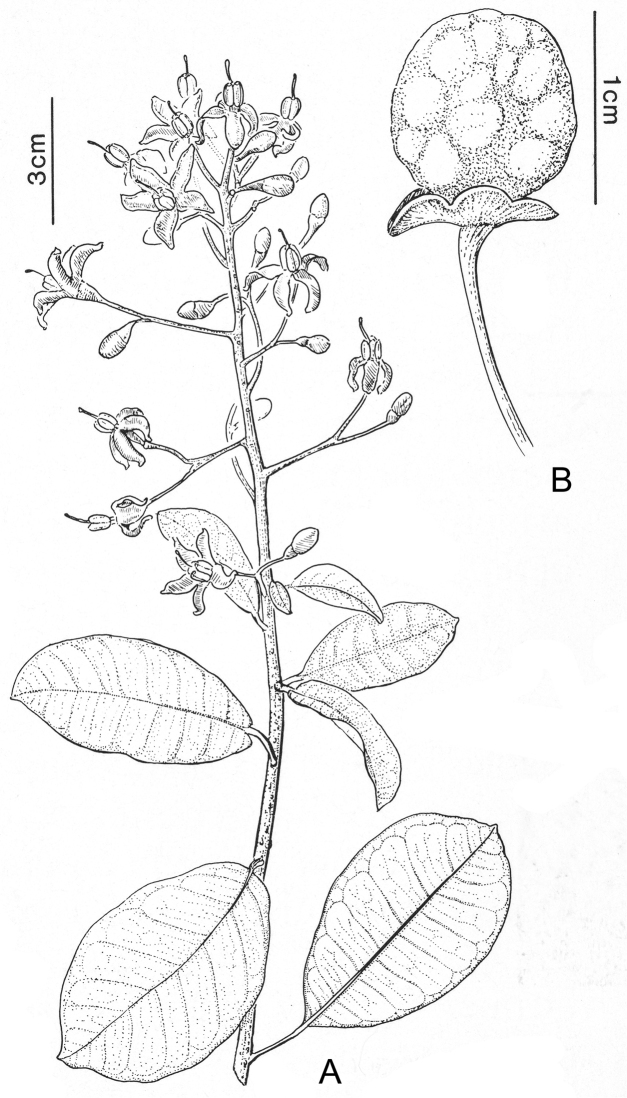
*Solanum madagascariense* Dunal. **A** Flowering branch **B** Berry showing thin pericarp through which seeds are visible (based on: A, *Bosser 16852* **B** *Homelle s.n.*). Adapted from D’Arcy and Rakotozafy (1994) with permission of Muséum National d’Histoire Naturelle.

##### Distribution

**Figure 18.**
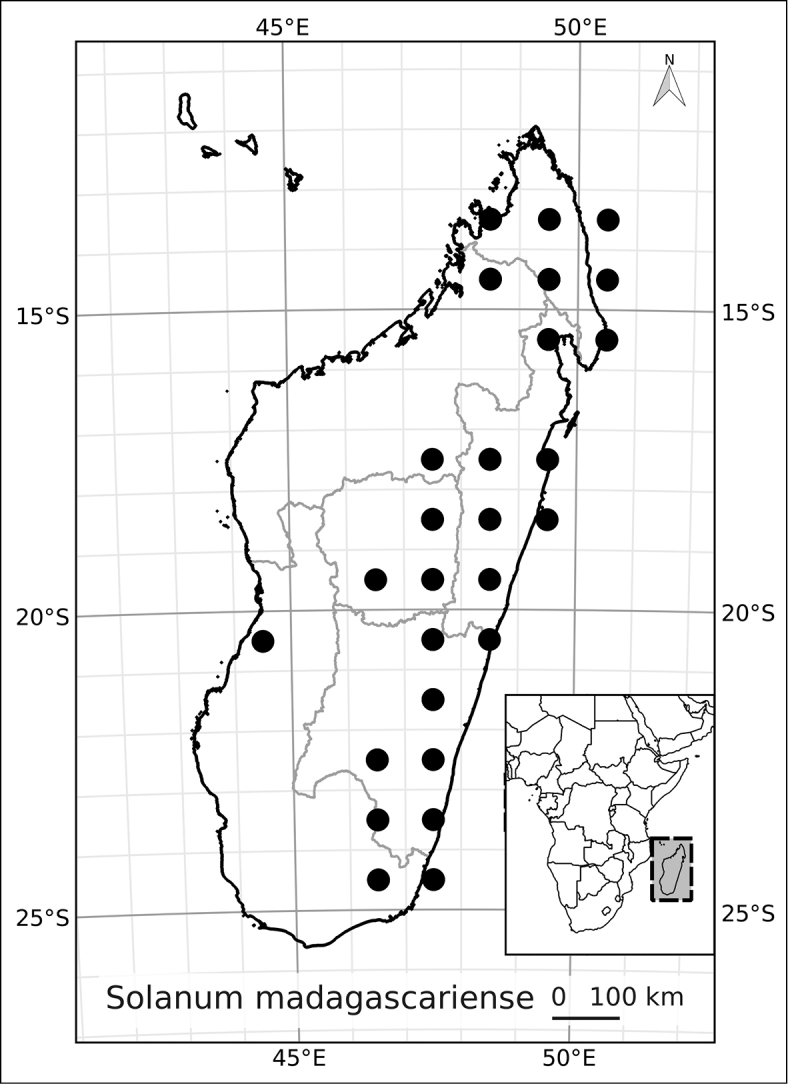
Distribution of *Solanum madagascariense* Dunal.

(Figure 18). Endemic to Madagascar, throughout central and eastern parts of the island, with a few records from humid forests on the western part of the island.

##### Ecology and habitat.

Humid and subhumid forests; sometimes found in disturbed vegetation by the roadside; 0–1500 m elevation.

##### Common names and uses.

Madagascar. Antsiranana: vahimasina (*Humbert 18109*), vahimbingy (*RN Madagascar 2738*, *Miller & Randrianasolo 4563*), vahinazo (*RN Madagascar 8388*), voajaboala (*Miller & Randrianasolo 4329*); Fianarantsoa: keranzy (*Anon. 4026*), vahimaitso (*Malcomer et al. 1581*), vahivahy (*Randriantafika 15*). No uses recorded.

##### Preliminary conservation status

(IUCN 2014). Least Concern (LC). EOO 551,384 km^2^ (LC), AOO 436 km^2^ (EN). *Solanum madagascariense* is a common liana, and occurs in several different forest types, including in some protected areas. Its local rarity and patchiness of distribution, along with its range of morphological varability (see below) suggest further studies as to local abundance are necessary.

##### Discussion.

*Solanum madagascariense* is a liana with prominent terminal inflorescences of 15–45 white to deep purple flowers (Fig. 1C), sturdy branches, and thick leaves. It occurs throughout Madagascar’s wet forests which are situated predominantly on the eastern coast, adjacent parts of the High Plateau, and northern parts of the island. It is the most common and variable species of endemic non-spiny *Solanum*.

*Solanum madagascariense* is similar and possibly closely related to the rare and local species *Solanum trichopetiolatum* and *Solanum humblotii*. It can be distinguished from *Solanum trichopetiolatum* by its glabrous petioles (versus petioles with long simple trichomes 0.5–0.15 mm long); *Solanum trichopetiolatum* also has looser inflorescences with finer branches and fewer flowers, a greater tendency towards discolorous oblong leaves and is restricted to a narrow area of Antsiranana. *Solanum madagascariense* differs from *Solanum humblotii* in its large, many-branched inflorescence with many flowers; *Solanum humblotii* has an unbranched inflorescence with few flowers and is restricted to the northern part of Madagascar in Toamasina.

*Solanum madagascariense* as delimited here encompasses great range of variation. It is possible to isolate groups of specimens with small long narrow leaves, groups of specimens with wide coriaceous leaves and fewer veins, and groups of specimens with sparse inflorescences and hariy leaves, but intermedidates between all these forms are common. The name *Solanum apocynifolium* has been used to describe individuals with tomentose stems and smaller leaves. Variation in indumentum is continuous with that observed in other populations of *Solanum madagascariense*, and leaf size seems to be largely determined by ecological factors. *Solanum apocynifolium* was accepted by Bitter (1917), tentatively accepted by D’Arcy and Rakotozafy (1994) with acknowledgement of continuous variation, and accepted at the level of variety by the late R.N. Lester (unpublished manuscript). Forms recognised as the two taxa are the extremes of a range of morphological variation and are here considered to be conspecific. *Solanum nitens* is a smaller glabrous variant of *Solanum madagascariense* with shiny subcoriaceous leaves and a shrubby, densely branched growth form that grows in drier, more central areas of Madagascar. It was accepted by Bitter (1917), reduced to a variety by D’Arcy and Rakotozafy (1994) and considered a synonym of *Solanum madagascariense* by Lester (unpublished manuscript). We agree with Richard Lester’s assessment and *Solanum nitens* is included in *Solanum madagascariense* as an arid environment variant. *Solanum antalaha* is a wet environment variant of *Solanum madagascariense* occurring further north, representing mature or shade dwelling individuals. *Solanum antalaha* has glabrous leaves, petioles, stems, and corolla, and leaves with fewer veins; it was described as having larger anthers but these are less than 3.8 mm long on the type specimen, within the variation range of *Solanum madagascariense* (2.5-4 mm long). *Solanum marojejy* was described as a Marojejy endemic with dorsally papillose anther surfaces and glabrous petioles; both of these features, however, are commonly observed in populations of *Solanum madagascariense* from other localities. *Solanum marojejy* is here considered to be conspecific with *Solanum madagascariense*. *Solanum clerodendroides* is a specimen of *Solanum madagascariense* almost certainly incorrectly labelled as collected in Nigeria (fide H. Heine 1967, in sched.)

Unusually large papillae seen on the abaxial anther surface of some collections have been postulated to restrict access to pollen or provide support or orientation cues for pollinating bees (D’Arcy 1992). Only three specimens with lobed leaves have been seen, but lobed leaves may be more common in juvenile plants as is true in the Dulcamaroid clade (Knapp 2103). An unusual specimen at P from a cultivated plant in the garden at Antananarivo (*s. coll. 2174*, P04063654) is from a plant with inflorescences on many short branches and only 2-3 flowers per inflorescence and somewhat resembles *Solanum humblotii*, but morphologically conforms to *Solanum madagascariense* in terms of floral form, calyx size and leaf morphology.

We have chosen *Forsyth-Major 15* [K000414184] as the lectotype for *Solanum madagascariense* Dammer (nom. illeg., a later homonym of *Solanum madagascariense* Dunal) as it is the only unambiguous duplicate of the type collection number we have found. It is probable that the sheet at BM labelled *Forsyth-Major 55* [BM000887181] is another duplicate (and thus an isolectotype), curatorial annotation on the BM sheet suggests 55 may be an error for 15.

The protologue of *Solanum antalaha* cites a holotype at P; of the two duplicates of the type collection P00349362 has been selected as the lectotype because it annotated as “holotype” in W.G. D’Arcy’s handwriting.

##### Selected specimens examined.

**Madagascar. Antananarivo**: Manjakandriana, Mandraka, Aug 1906, *D’Alleizette 988* (P); Tampoketsa de Ankazobe, 5-12 km E of highway 31 km N of Ankazobe, 19 May 1974, *Gentry 11852* (MO, NY); Antananarivo-Nord, la forêt à l’Est d’Ambakolaona, 11 Nov 1912, *Humbert & Viguier 1265* (P); mont Kalambatitra et ses abords, Nov 1933, *Humbert 11901* (MO, P); foret d’Ankilahila, 16.2 km SE de Tsinjoarivo, le long de la riviere d’Andrindrimbola, 21 Jan 1999, *Messmer & Andriatsiferana 744* (G, K); Andramasina, Ambohimiadana, Kelilanina, Antsararoloha, 15 Dec 2009, *Rakotonasolo et al. RNF-1535* (K); forêt d’Andranomay à 2 km à l’Est d’Andranomay et 13 km au SE d’Ankazobe, 20 Dec 1996, *Randrianaivo 47* (BR, MO); Ambohitantely RS, à 36 km au Nord Est d’Ankazobe, 1 km à l’Est des Batiments de l’Angap, 14 Jan 1997, *Randrianaivo et al. 59* (MO, NY, TAN). **Antsiranana**: Ampasindava, forêt de Betsitsika, 12 Dec 2008, *Ammann et al.193* (G); Anamalaho, massif de Makirovana, au Nord-Ouest de Sambava, 22 Aug 2007, *Andriamihajarivo et al. 1256* (TAN); Tsaratanana Massif, along path from Mangindrano to Mahatsabory Mica, 19 Oct 2001, *Birkinshaw et al. 978* (MO, NY, P, TAN); montagnes entre le haut Sambirano et le haut Maivarano (entre Mangindrano et Ampanompia), Nov 1937, *Humbert 18109* (P); environs d’Andapa, bassin de la Lokoho, 1948, *Humbert & Capuron 21939* (P); Reserve Speciale Manongarivo, E of Ankaramy, Bekolosy, 7 Dec 1992, *Malcomber et al. 1998* (BR, G, K, MO x2, NY, P); Reserve Naturelle Marojejy, along the trail to the summit of Marojejy Est, NW of Mandena between the first and second camps, 6 Oct 1988, *Miller et al. 3413* (K, MO x2, P, TAN); commune rurale de Daraina, forêt de Binara, 20 Nov 2005, *Nusbaumer & Ranirison 1637* (G,K); commune rurale de Bealampona, village de Mandritsarahely, Sud-Ouest d’Andapa, 18 Oct 1994, *Ravelonarivo et al. 418* (MO, NY, P); Ambilobe, Beramanja, fôret de Salabenono, sur la chaine Galoko, 7 km au sud-est d’Anketrabe, 25 Nov 2006, *Razafitsalama & Torze 1145* (MO, TAN); Marotolana, Ampanompy, 5 Apr 2001, *Razakamalaka et al. 104* (MO, TAN); canton de Manaka est, district d’Antalaha, Rés. nat. 3, 9 Nov 1952, *Réserves Naturelles Madagascar 4480* (P); Marozato, district d’Ambanja, Rés. 4, 18 Nov 1952, *Réserves Naturelles Madagascar 4499* (P, TAN); Sambava, Marojejy National Park, path down from camp 3 (camp Simpona) to camp 2 (camp Marojejia), 17 Oct 2011, *Vorontsova et al. 498* (K, TAN); Cap Masoala Grand Parc, haut vallée d’Anaovanandrano, 26 Sep 2003, *Wohlhauser et al. 648* (G). **Fianarantsoa**: Fianarantsoa rural, forêt d’Ambondrombe, *Anonymous 4026* (P); Betanatana Forest Reserve, Fargangana, Ankarana, Manobo, 28 Aug 2008, *Bussmann et al. 15257* (MO, TAN); forêt basse au PK 298 (Sud Ambositra), 12 Dec 1974, *Cremers 3635* (MO, P); Vondrozo, province de Farangana, bord de route en forêt, 9 Sep 1926, *Decary 5235* (P); Ambositra, Ambohimitombo, 21 Dec 1894, *Forsyth-Major 325* (K); Bassin de l’Itomampy, mont Papanga prés de Befotaka, Dec 1928, *Humbert 6912* (P); massif de l’Ivakoany, pentes orientales du massif, 1933, *Humbert 12240* (P); forêt ombrophile d’Ambatofitorahana, 2 Mar 1960, *Keraudren 229* (MO); Parc National Ranomafana, Parcelle I, south of Ambohimiera, valley of Sakavolo river, 15 Sep 1992, *Malcomber et al. 1581* (BR, G, K, MO x2, NY, P); Réserve Spéciale de Manombo, parcelle 2, 37 km au SW de Farafangana, 21 Aug 1995, *Messmer & Rakotomalaza 46* (TAN); Reserve Naturelle Integrale d’Andringitra, 50 km S of Ambalavao, near abandoned meteorological station above Ambalamarina, 12 Jan 1987, *Nicoll 236* (MO, TAN); limite nord de la Réserve Spéciale d’Ivohibe, 7.5 km ENE d’Ivohibe, 11 Oct 1997, *Rakotomalaza et al. 1398* (G, MO, NY); Ambositra, Ambalamanakana, 28 Jan 2005, *Ralimanana et al. 449* (K); forêt dense humide de l’Est d’Andranobetokana, Ampasimadinika, Marofototra, Mananjary, 16 Jan 1999, *Randriantafika et al. 15* (MO); 2 km Ouest d’Andrambovato, bord de la rivière Tatamaly, haut versant, Fivondronana Tolongoina., 19 Oct 2000, *Randriantafika 176* (MO, NY, P); Ambalavao, 23 Jan 1958, *Réserves Naturelles Madagascar 9981* (P); 7 km W of Ranomafana, S of the Namorona River at Duke University Primate Center study site and along road N of river, eastern domain, 28 Oct 1987, *Schatz et al. 1713* (BR, K, MO, P); Ambalamanakana, 18 Dec 1959, *Schlieben 8197* (BM, BR, G, TAN). **Mahajanga**: ruisseau Ambatoharanana, Andranomena, Matsoandakana, 14 Feb 2008, *Bernard et al. 813* (TAN); Tsitondroina, 16 Apr 1941, *Herb Jard Bot Tananarive 4812* (P); Tsaravilona, Amparihy, Androva, suivant une ligne de crête vers le nord est, 26 Feb 2008, *Ravelonarivo et al. 3042* (K); Bealanana, Mangindrano, Ambohimirahavavy, Antsahivo, point côté, 20 Oct 2005, *Wohlhauser et al. 786* (MO, P). **Toamasina**: Parc National de Zahamena, Fivondronana, Antanandava, Antenina, 31 Jan 2002, *Andrianjafy et al. 273* (MO, NY, P); Aloatra-Mangoro, Ambatondrazaka, Didy, Antsevabe, Sahananto, Rivière de Sahananto, 12 Dec 2005, *Andrianjafy et al. 1535* (TAN); Ambanizana, Maroantsetra, Anjahana, Ambanizana, 16 Sep 2002, *Antilahimena 1405* (MO, NY); forêt d’Analalava, sous-prefecture Tamatave II, Morarano, à 7 km du SO de Foulpointe, 10 Mar 2005, *Birkinshaw et al. 1442* (TAN); Ambatondrazaka, *Cours, G. 1058* (P); Moramanga, Forêt de Perinet à 7 km à l’Est de Perinet vers Tamatave, 28 Feb 1971, *Cremers 1481*-*3* (P); Ambodimanga à Tamatave, 11 Oct 1957, *Herbier de la Station Agricole de l’Alaotra 2807* (MO); Onibe, massif de l’Andragovalo au Sud-Est du lac Alaotra, Rés. Nat. 3 dite de Zekamena, bassin de l’Onibe, Oct 1937, *Humbert 17746* (MO, P); Maroantsetra, massif de l’Anjanaharibe (pentes et sommet Nord) à l’Ouest d’Andapa, 1951, *Humbert & Cours 24533* (P); Betampona Rèserve Naturelle Intègrale, 40 km NW of Toamasina, 27 Sep 1993, *Lewis & Razafimandimbison 639* (MO); Forêt de Mantady, road to N (Tamatave), 29 Oct 1993, *Nek et al. 2021* (BR, TAN); Ikongo, Ranomafana, 50 km E of Fianarantsoa on Mananjary road, across R. Namorona from Ambatolahy, 4 Nov 1986, *Nicoll 139* (K, MO x3, P, TAN); bois des environs de la baie d’Antongil, 1912, *Perrier de la Bâthie 8686* (P); Vatomandry, Ambalabe, Ambinanindrano II, hameau villageois Tobin’i Foara, le long du sentier Nord, 4 Oct 2005, *Ranaivojaona et al. 1185* (MO,P); canton de Varaina Manatsambaliny est, district d’Ambatondrazaka, 13 Nov 1948, *Réserves Naturelles Madagascar 1598* (P); Parc National de Masoala, excursion d’Andranobe à Bedinta, entre Ambatoavo et Bedinda, Nov 2001, *Sauquet et al. 86* (P); Ankirindro Massif, slopes above the village Ambodivato, ca. 5 km NW of Ambinanitelo along the Vohimaro River, 20 Nov 2002, *Schatz & Antilahimena 4018* (MO,NY); Vohibinany, Anivoranokely, Ambidimanga, 21 Sep 1954, *Vigreux 15491* (P). **Toliara**: Betroka, Ivahona, Réserve Spécial de Kalambatritra. Forêt d’Analamaro, 5 Nov 2005, *Andrianjafy et al. 497* (MO); Morondava, Bara, 1880, *Cowan s.n.* (BR,P); col de Tsitongabarika, 16 Nov 1932, *Decary 11017* (P); bassin de la Manampanihy, Col de Fitana, 15 Oct 1928, *Humbert 6050* (P); massif du Beampingaratra, vallée de la Maloto, 1928, *Humbert 6302* (NY, P); N of Taolagnaro town; along Andranoroa River, border of Marosoy Forest and Andohahela Reserve, 2 Dec 1989, *McPherson 14591* (MO).

#### Solanum myrsinoides

Taxon classificationPlantaeSolanalesSolanaceae

D’Arcy & Rakot., Fl. Madag., Fam. 176: 115. 1994.

Figure 19

##### Type.

Madagascar. Antsiranana: partie occidentale du Massif de Marojejy (Nord-Est) de la vallée de l’Ambatoharanana au bassin supérieur de l’Antsahaberoka, 1200-1400 m, 9 Nov – 2 Dec 1959, *H. Humbert & P. Saboureau 31469* (lectotype, designated here: P [P00352407]; isolectotypes: P [P00352405], P [P00352406], MO [MO-277613]).

##### Description.

Liana or epiphyte growing up to 4 m above ground. Stems somewhat ribbed or winged, glabrous, the wings to 2 mm wide on herbarium specimens; new growth completely glabrous; bark of older stems longitudinally ridged (when dry), brown. Sympodial units plurifoliate, the leaves not geminate, evenly distributed along branches. Leaves simple, 10–20 (31) cm long, 2.5–4.5 (6) cm wide, linear to elliptic or obovate, thick coriaceous to fleshy and probably somewhat succulent, concolorous, glabrous on both surfaces; major veins 4–5 pairs, not easily visible, the finer venation not visible; base cuneate to rounded; margins entire, sometimes revolute in dry material; apex acute to acuminate; petiole 1–2.5 cm long, thick and fleshy, often somewhat flattened and drying with prominent wings and ridges perpendicular to the axis, glabrous, possibly twining. Inflorescences terminal, at the apex of terminal branches or slender lateral branches, 7–11 cm long, furcate or occasionally unbranched, with 2–6 flowers; peduncle 4–9 cm long, less than 1 mm diameter at the base, much thinner than the stem, glabrous; pedicels 1.5–2.5 cm long, apically dilated, glabrous, drying ridged, articulated in the lower ⅓, 0.1–0.8 cm from base; pedicel scars prominent peg-like stumps, darker distally, irregularly spaced 3–10 mm apart. Buds oblong, the corolla strongly and long-exserted from the calyx tube. Flowers 5-merous, apparently all perfect. Calyx 1–2 mm long, ca. 6 times shorter than the corolla at anthesis, broadly cup-shaped, the lobes less than 1 mm long, 2–3 mm wide at base, broadly deltate, obtuse at the tips, glabrous with tufts of papillae at the lobe apices and some papillae along the margins. Corolla ca. 2 cm in diameter, white to violet-purple, stellate, lobed almost to base, the lobes 10–15 mm long, 2.5–3 mm wide, narrowly deltate to long-triangular, both surfaces glabrous, the margins densely papillate, the tips cucullate. Stamens equal; filament tube ca. 0.5 mm long; free portion of the filaments ca. 1.5 mm long, glabrous; anthers ca. 7 mm long, ca. 1.5 mm wide, ellipsoid, tightly connate, papillate abaxially, poricidal at the tips, the pores slightly smaller than anther apices, clearly delineated and not lengthening with age. Ovary conical, glabrous; style 8–9 mm long, protruding 1–2 mm beyond the anthers, filiform, straight, glabrous; stigma clavate, the surface smooth. Fruit a long-ellipsoid, fusiform or pyriform berry, (1.5)3–4 cm long, 1–2 cm diameter at maturity, with an apical beak 2–5 mm long, basally rounded or the base narrowing and the berry fusiform, ripe fruit colour not known, mature pericarp glabrous, the flesh thick and the fruit apparently spongy or woody, no seeds borne in the elongate apex; fruiting pedicels ca. 1.6 cm long, 1–2 mm diameter at the base, spreading; fruiting calyx lobes minute, becoming reflexed as the fruit enlarges. Seeds 5–8 per berry, 5–6 mm long, 3–4 mm wide, flattened reniform and imbedded in thick pulp, dull golden-yellow, the surfaces minutely pitted, the testal cells rectangular (? – only seen in immature seeds) in outline.

**Figure 19.**
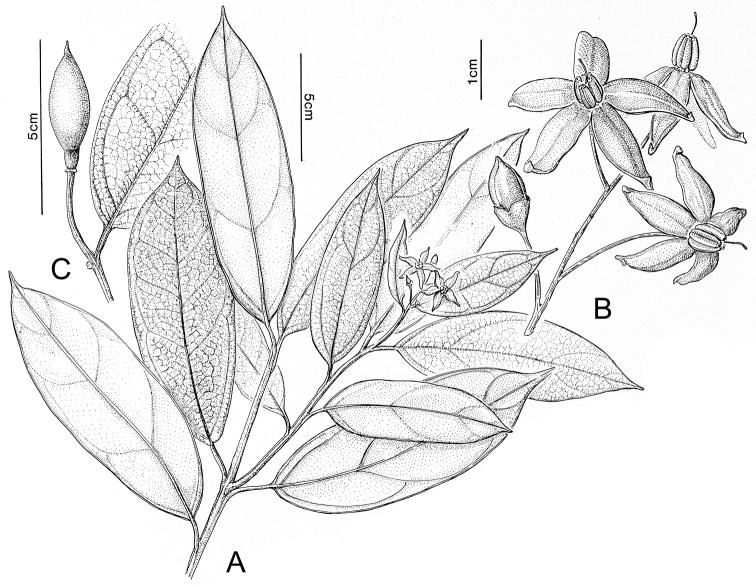
*Solanum myrsinoides* D’Arcy & Rakot. **A** Flowering branch **B** Open flowers **C** Berry showing tapering at both ends (based on: **A** *Humbert & Saboureau* 31429 **B** *Humbert 22253* **C** *Cours 3326*). Adapted from D’Arcy and Rakotozafy (1994) with permission of Muséum National d’Histoire Naturelle.

##### Distribution

**Figure 20.**
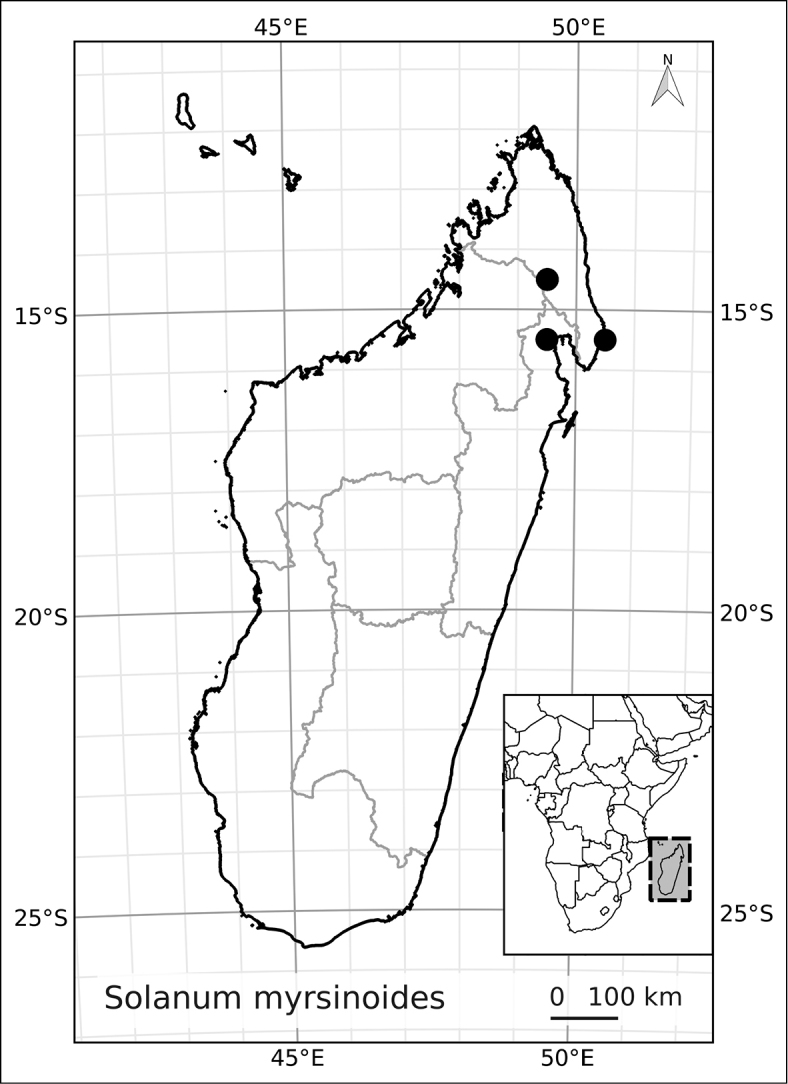
Distribution of *Solanum myrsinoides* D’Arcy & Rakot.

(Figure 20). Endemic to northeastern Madagascar, in Toamasina and Antsiranana provinces.

##### Ecology and habitat.

Wet montane forests; 500–1500 m elevation.

##### Common names and uses.

None recorded.

##### Preliminary conservation status

(IUCN 2014). Vulnerable (VU B1a,b iii); EOO 7,482 km^2^ (VU), AOO 52 km^2^ (EN). *Solanum myrsinoides* is known from fewer than 10 locations, all in a restricted area around Antogil Bay in northeastern Madagascar. Its population density may be underestimated as it is a rarely collected canopy liana or epiphyte. It occurs in several protected areas in the region and so is not assessed here as Endangered as the small AOO might warrant.

##### Discussion.

*Solanum myrsinoides* is a distinctive wet forest endemic. Its large coriaceous or fleshy leaves, often epiphytic habit and apically elongated fruit are unusual in *Solanum*. *Solanum myrsinoides* seems to grow as a liana but can lose connection with the ground and survive as an epiphyte as high as 4 m in the canopy. The stems of *Solanum myrsinoides* are often winged. The thick petioles are flexuous and have a wrinkled woody texture when dry; it seems likely that these are used for climbing. Size and shape of the leaves is highly variable between collections and probably therefore plants. The peduncle and rachis are long and slender, in contrast to the thick stems (Fig. 19). It is the only *Solanum* species in Madagascar that is completely glabrous on all vegetative parts, with nothing but small papillae on the apices of the calyx and corolla lobes. D’Arcy and Rakotozafy (1994) report that these trichomes are sometimes branched, but we have not seen this in any of the specimens we have examined.

*Solanum myrsinoides* is unlikely to be confused with another species of *Solanum*. Some large leaved individuals of *Solanum truncicola* are highly reminiscent of *Solanum myrsinoides* in their leaves and habit, perhaps reflecting common adaptation to montane wet forest understorey. *Solanum myrsinoides* can be distinguished by its calyx lobes that are less than 1 mm long (versus 4–11 mm long), inflorescences with 2–6 flowers (versus 1–2 flowers), and distinct slender peduncle 4–9 cm long (versus peduncle absent). The distribution of the two species does not overlap; *Solanum myrsinoides* occurs at lower elevations further north.

The developing fruit of *Solanum myrsinoides* has a globose basal part and a clearly distinct elongated apical beak that gradually tapers to a point (Fig. 19). At maturity the basal globose part narrows to produce a woody (in dry specimens) ovoid shape; sometimes the basal part of the fruit also narrows so the berry is fusiform (spindle-shaped) and tapers at both ends. Among the non-spiny *Solanum* species endemic to Madagascar, similar apically pointed fruits occur in *Solanum betroka*, *Solanum imamense*, *Solanum sambiranense*, and *Solanum truncicola*; globose berries have been observed in *Solanum imamense*, *Solanum madagascariense*, and *Solanum trichopetiolatum*. The thick, possibly spongy flesh seen in *Solanum myrsinoides* is not apparent in other Madagascar solanums, but these species are rarely collected in fruit; further field observations are necessary.

A holotype and an isotype are cited from P. There are three duplicates of the type collection at P and a single sheet was not selected at the time of description. P00352407 is marked “type” with a label attached over (and apparently thus after the determination) D’Arcy’s determination slip, indicating that perhaps it is the one D’Arcy and Rakotozafy intended as the holotype. We here select this sheet as the lectotype to avoid ambiguity because there is no evidence of the author’s selection of a particular sheet as holotype (see McNeill 2014).

##### Selected specimens examined.

**Madagascar. Antsiranana**: Sambava, pentes orientales du Massif de Marojejy, (N-E), a l’ouest de la riviere Manantenina, affluent de la Lokoho, 15 Dec 1948, *Humbert 22504* (K, Px2); Ambatosoratra, Vallée de la Lokoho (Nord-Est), mont Ambatosoratra au Nord d’Ambalavoniho et de Belaoka, Jan 1949, *Humbert & Cours 22863* (MO, P); contreforts occidentaux du massif de Marojejy (Nord-Est) près du col de Doanyanala (limite des bassins de la Lokoho et de l’Andraronga, 1949, *Humbert 23142* (P); Reserve Naturelle de Marojejy, along the trail to the summit of Marojejy Est, N of Mandena, 3 Dec 1989, *Miller & Randrianasolo 4675* (MO, TAN); Préfecture d’Antalaha, Sous-Préfecture d’Andapa, commune rurale de Bealampona, sud-ouest d’Andapa, Réserve Spéciale d’Anjanaharibe-Sud, village d’Andranotsarabe, suivant la route Nationale d’Andapa-Bealanana de la piste vers à l’ouest, Ambatomainty, Camp No. 2, 3 Nov 1994, *Ravelonarivo & Rabesonina 475* (MO); limite entre Anjialavabe et Doany, Andapa, deuxième montagne d’Ankarongameloka, *Ravelonarivo 1889* (P). **Toamasina**: Marovovonana, Befotsila Camp, 1 Sep 2004, *Antilahimena 2694* (MO, P); Masoala National Park, N ridge of Ambohitsitondroinan’Mahalevona, just below summit, ESE of village of Mahalevona, 23 Feb 2003, *Lowry et al. 6120* (MO, TAN); Antongil, environs de la Cave, baie d’Antongil, Aug 1912, *Perrier de la Bâthie 8718* (P); Masoala peninsula, Ambanizana, ‘S Trail’ (S of Androka river) climbing into hills SE of Ambanizana, 1 Nov 1992, *Schatz et al. 3391* (MO, P).

#### Solanum runsoriense

Taxon classificationPlantaeSolanalesSolanaceae

C.H.Wright, Uganda Prot. 1: 362. 1902.

Figure 21

Solanum benderianum C.H.Wright, Fl. Trop. Afr. [Oliver et al.] 4(2): 212. 1906, as “*bendirianum*”. Type. Ethiopia. Oromia: Harwash and Maki Rivers [Shewa Region], Jan 1899, *M.S. Wellby s.n.* (lecctotype, designated here: K [K000788686]).
Solanum benderianum Dammer, Bot. Jahrb. Syst. 38: 184. 1906 [“1907”], nom. illeg., isonym, not Solanum benderianum C.H.Wright, 1906. Type. Ethiopia. Amhara: “Kirchengehölzes Herroe Gottes Georgis bei Gaffat” [South Gonder, near Debre Tabor], 8400 ft., 1 Oct 1863, *G.H.W. Schimper 1227* (lectotype, designated here: BM [BM000847516]; isolectotypes: BM [E [E00193238], K [K000414030, K000414031], US [US0027474=US-806522], W [W18990146870], WU [WU0033424, WU0033425]).
Solanum benderianum C.H.Wright var. lanceolatum Bitter, Bot. Jahrb. Syst. 54: 488. 1917. Type. Ethiopia. Oromia: “Gallahochland, Kiritscha, Utadara”, Dec 1900, *O. Neumann 62* (B, destroyed, no duplicates found); Sidamo, Njam-Njam [Jem-Jem], Dec 1900, *H. Ellenbeck 1761* (no herbarium cited).
Solanum benderianum C.H.Wright var. ruwenzoriense Bitter, Bot. Jahrb. Syst. 54: 489. 1917. Type. Uganda. Western: Ruwenzori, Kivatu, 2500-2800 m, 1893-1894, *G.F. Scott-Elliot 7733* (lectotype, designated here: BM [BM000847515]; isolectotypes: K [K000414032]).
Solanum keniense Standl., Smithson. Misc. Coll. 68, no. 5: 16. 1917, nom. illeg., non Solanum keniense Turrill, 1915. Type. Kenya. Central: western slopes of Mount Kenya, along the trail from West Kenya Forest station to the summit, ca. 3630 m, 21-27 Sep 1917, *E.A. Mearns 1416* (holotype: US [US00027640]).
Solanum benderianum A.Schimp. ex Engl., Abh. Naturwiss. Vereine Bremen 25: 246. 1922. Type. Probably not intended as a new name; orthographic correction to Wright (1906).
Solanum longipedicellatum De Wild., Pl. Bequaert. 1: 428. 1922, nom. illeg., non Solanum longipedicellatum Bittter, 1912. Type. Democratic Republic of the Congo. Nord-Kivu: Ruwenzori, Lanuri [Valley], ca. 3000 m, 3 Jun 1914, *J. Bequaert 4676* (lectotype, designated here: BR [BR0000008993151]; isolectotypes: BR [BR0000008993120], LWI [LWI486608136]).
Solanum dewildemanianum Robyns, Fl. Spermatophyt. Parc. Nat. Albert 2: 209. 1947. Type. Based on Solanum longipedicellatum De Wild.
Solanum runsoriense C.H.Wright subsp. benderianum (C.H.Wright) Edmonds, Fl. Trop. East Africa, Solan. 120. 2012, as “*bendirianum*”. Type. Based on Solanum benderianum C.H.Wright

##### Type.

Uganda. Western: Ruwenzori, [rec. at Kew 13 Mar 1901], *W.G. Doggett s.n.* (holotype: K [K000413968])

##### Description.

Vine or woody liana, sometimes semi-herbaceous, to 10 m. Stems flexuous, terete, glabrous to densely pubescent with mixed simple 4–6-celled and short-branched dendritic uniseriate trichomes 0.5–1(1.5) mm long, glabrescent when older; new growth almost glabrous to densely pubescent with dendritic and simple uniseriate trichomes, when these dense the new growth appearing golden in dry material. Bark of older stems reddish brown, smooth. Sympodial units plurifoliate, the leaves not geminate, evenly distributed along branches. Leaves simple, 4–12 cm long, 2–5.6 cm wide, elliptic or less commonly ovate, membraneous, the adaxial surfaces glabrous to moderately pubescent with mostly simple uniseriate trichomes 0.5–1 mm long, in more pubescent individuals some trichomes dendritic, the abaxial surfaces glabrous or with a few simple uniseriate trichomes along the veins and margins to densely pubescent with short-branched dendritic trichomes 0.5–1.5 mm long, these often drying golden; major veins 5–10 pairs, usually yellowish beneath, especially on glabrous individuals; base acute; margins entire, if the leaves otherwise glabrous the margins have a few simple trichomes to 1 mm long near the base; apex acute to acuminate; petioles 0.7–3.2 cm long, very variable in length along the stem, nearly glabrous to densely dendritic-pubescent, sometimes twining. Inflorescences terminal, 5–15(+) cm long, lax and open, many times branched, with 20–100 flowers many open at the same time, pubescence in parallel to that of stems and leaf undersides; peduncle ).5-)2–4.5 cm long, sometimes the inflorescence branches starting very near the last stem leaves; pedicels 0.8–1.2 cm long, ca. 0.5 mm in diameter at the base, ca. 1 mm in diameter at the apex, spreading at anthesis, pubescent like the rest of the inflorescence axis, but slightly less densely, articulated at the base leaving a very minute raised portion of the axis; pedicel scars irregularly spaced 1–6 mm apart. Buds ellipsoid, the corolla completely enclosed in the calyx tube until shortly before anthesis, when young the elongate sepals spreading at the bud tips. Flowers 5-merous, heterostylous, short-styled and long-styled flowers apparently borne on different plants and the plants possibly dioecious. Calyx tube 1–1.5 mm long, open-conical, the lobes 1–2 mm long, narrowly triangular with a long-acuminate tip, usually thickened and keeled abaxially (this easier to see on glabrous specimens), glabrous to densely pubescent with mixed dendritic and simple uniseriate trichomes, even if glabrous the tips with a tuft of simple uniseriate trichomes. Corolla (1.5)2–3.5 cm in diameter, pale violet or white with a darker purple centre, stellate, lobed ca. halfway to the base, the lobes 7–9 mm long, 6–8 mm wide, spreading at anthesis, variably pubescent, from almost glabrous with dense papillae on the tips and margins to densely pubescent with minute dendritic trichomes all usually < 0.5 mm long (to 1 mm long in *Taylor 1408*). Stamens equal; filament tube absent; free portion of the filaments 1.5–2.5 mm long, glabrous or sparsely pubsvent with dendritic trichomes (on pubescent plants); anthers 2.5–3 mm long, 1.1.5 mm wide, ellipsoid, bright yellow, connivent to somewhat spreading, smooth abaxially, somewhat sagittate at the base, poricidal at the tips, the pores lengthening to slits with age, in dry material the pores thickened and paler at the distal tips. Ovary globose, glabrous; style of different lengths on individual plants, the short-styled plants with styles 2.5–3 mm long, held within the anther cone or exceptionally to 5 mm long and at the level of the anther tips, the long-styled plants with styles 7–9 mm long, exserted 4–6 mm beyond the anther cone, glabrous (on *Wollaston s.n.* [SS plant from Ruwenzori] densely dendritic-pubescent in the distal half); stigma of short styles minutely capitate, of long styles strongly bilobed or clavate, the surfaces minutely papillate. Fruit a globose berry, 0.8–0.9 cm in diameter, purple when ripe, the pericarp thin and brittle in dry material, probably shiny in fresh material; fruiting pedicels 2–2.5 cm long, tapering markedly from a base ca. 1 mm in diameter to the apex 4–5 mm in diameter, the distal half usually darker in dry material, spreading, possibly somewhat woody; fruiting calyx lobes to 0.5 cm long, spreading. Seeds 10–12(16) per berry, 2.5–3.5 mm long, 2–2.5 mm wide, ovoid reniform, reddish brown, the surfaces shallowly pitted, the testal cells very small, pentagonal in outline.

**Figure 21.**
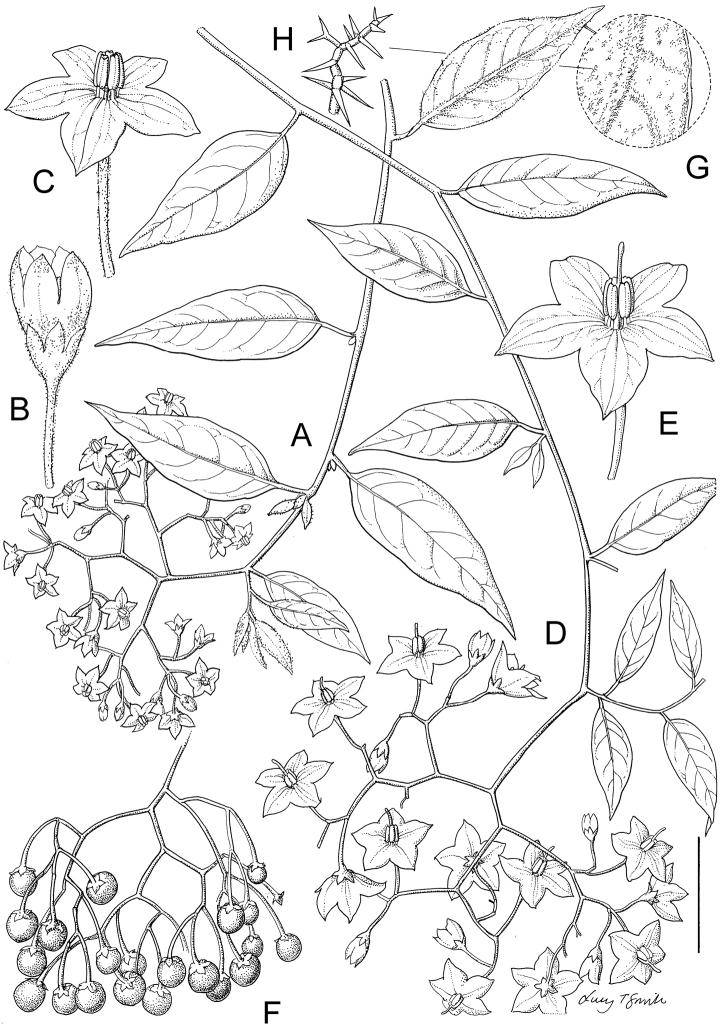
*Solanum runsoriense* C.H.Wright. **A** Flowering branch from short-styled plant **B** Bud from short-styled flower **C** Open short-styled flower **D** Flowering branch of long-styled plant **E** Open long-styled flower with long-exserted style **F** Infructescence **G** Detail of dense dendritic pubescence of leaf undersides **H** Elongate dendritic trichome. (Based on: **A–C** *Napier 5130*; **D, E, G, H** *Friis et al. 3610*; **F** *Mooney 7016*). Scale bar: **A, D, F** = 4 cm; **B, C** = 7 mm; **E** = 1.5 cm; **G** = 5 mm; **H** = 0.4 mm. Drawn by Lucy T. Smith.

##### Distribution

**Figure 22.**
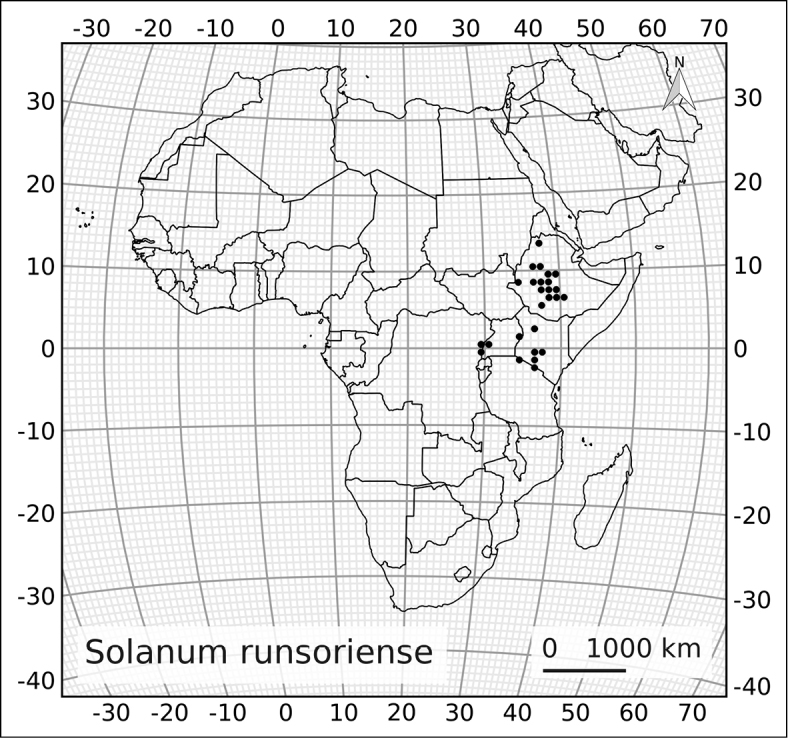
Distribution of *Solanum runsoriense* C.H.Wright.

(Figure 22). In the mountains of eastern Africa in Ethiopia, Kenya, Uganda, and the Democratic Republic of the Congo.

##### Common names and uses.

None recorded.

##### Ecology and habitat.

Montane forests, bamboo forests; often in open areas and forest edges; 1500–3700 m elevation.

##### Preliminary conservation status

(IUCN 2014). Least Concern (LC). EOO 1,346,193 km^2^ (LC), AOO 200 km^2^ (EN). Although *Solanum runsoriense* as delimited here occupies a large geographical range, it is found at high elevations on isolated mountain systems. Genetic differentiation at the population level across its range could therefore be important for conservation and should be assessed.

##### Discussion.

*Solanum runsoriense* is a montane forest edge species with large, openly branching inflorescences of purple (“blue”) flowers. It is not likely to be confused with any other species on the African continent; it the only species of the ANS clade with short-styled and long-styled flowers (see below, Fig. 21). *Solanum runsoriense* is partly sympatric with *Solanum terminale* but occurs at higher elevations and apparently in wetter forests. *Solanum terminale* has more deeply stellate corollas, flowers tightly clustered in groups rather than evenly and widely spaced on the inflorescence branches, and bright red rather than blackish purple berries. Pubescence is extremely variable in *Solanum runsoriense*; populations from Ethiopia (described as *Solanum benderianum*) are almost completely glabrous on stems and leaves, while populations from the Ruwenzoris in Democratic Republic of the Congo and from the Aberdare Mountains in Kenya are densely pubescent with golden dendritic trichomes. Individual populations are isolated in what has been recognised as the Afromontane (Wright 1978, 1981) or Tropic-montane Flora (Linder 2014) and limited gene exchange may allow these variants to become fixed. Flower, fruit and seed morphology are constant throughout the species range.

Edmonds (2012) placed *Solanum macrothyrsum* in synonymy with *Solanum runsoriense*; she cited Dammer (1906) and Jaeger (1985) both of whom suggested there were affinities between the two taxa. The two taxa share openly branched inflorescences and *Solanum macrothyrsum* is superfically similar vegetatively to more glabrous forms of *Solanum runsoriense*, but *Solanum macrothyrsum* has deeply stellate corollas and anthers that open with two terminal pores that never extend to slits as the flower ages, while *Solanum runsoriense* has shallowly stellate corollas and anther pores that extend to longitudinal slits with age.

*Solanum runsoriense* has the only member of the ANS clade that is heterostylous; in all but one of the specimens we have examined flowers on a given stem are either short-styled or long-styled. The Ethiopian collection *Gilbert 138* (K000788688) is the only one we have seen with fruit and short-styled flowers on same plant. No other inflorescences with short-styled flowers had fruits, and when specimens were fruiting, all flowers in the inflorescence had set fruit. Collections are often of some sheets with flowers (short-styled) and duplicates with fruit (e.g., *Schimper 1227*); it is not clear if these represent branches from a single plant or different individuals. *Solanum runsoriense* could be either monoecious or dioecious, but most monoecious solanums have a mixture of short- and long-styled flowers in the same inflorescence (Diggle and Miller 2004; Miller and Diggle 2003; Whalen and Costich 1986) so we suspect *Solanum runsoriense* is another instance of dioecy in the genus (see Symon 1970; Symon 1979; Anderson and Symon 1989; Knapp et al. 1998; Martine et al. 2009). Field observations on the breeding system of *Solanum runsoriense* are a priority, and microscopic examination of pollen morphology to see if long-styled flowers have inaperurate pollen like other dioecious solanums (e.g., Knapp et al. 1998) might help to determine the breeding system of this species.

Engler (1892) was the first to publish the name *Solanum benderianum*, but without description; he attributed the name to Schimper and cited Schimper’s 1863 manuscript list of plants collected in Ethiopia. Wright (1906) and Dammer (1906) both used Engler’s epithet and provided descriptions, but Wright’s treatment was published in February 1906, a few months before Dammer’s that appeared in August 1906 making *Solanum benderianum* Dammer an isonym and illegitimate. Both botanists attributed the name to Schimper, cited Engler’s (1892) publication and based their descriptions on two collections (presumably different duplicates), *Schimper 1227* and *Scott-Elliot 7733*. Wright (1906) also cited *Wellby s.n.* from Ethiopia. Bitter (1917) later used *Scott-Elliot 7733* as the type of his var. *ruwenzoriense*. We have lectotypified these three names with the three different specimens used in various combinations in their protologues in order to avoid making them homotypic. Edmonds (2012) incorrectly used Wright’s (1906) mis-spelling of the name “*bendirianum*”; we consider this a correctable spelling mistake because Wright cited Engler’s publication in which the name was spelled in the way Schimper intended, to honour his son-in-law “Herr Bender”.

De Wildeman (1922) specified no herbarium or sheet in his description of *Solanum longipedicellatum* (a later homonym of *Solanum longipedicellatum* Bitter, a synonym of the potato species *Solanum stoloniferum* Schltdl., Spooner et al. 2004). Of the two sheets of *Bequaert 4676* at BR, we have chosen that (BR0000008993151) annotated incorrectly as “holotype” by the late R.N. Lester as the lectotype because it has a label with the number and collecting locality. The isolectotype at BR (BR0000008993120) has no original label, but has a scrawled sheet in De Wildeman’s handwriting stating the differences between his species and *Solanum runsoriense*.

##### Selected specimens examined.

**Democratic Republic of the Congo**. **Nord-Kivu**: Upper Ruamoli valley, 3 Aug 1952, *Ross 800* (BM); Katwah Kitumo, pistes des Beulele et a Mahonge (P.N.A. [Parc Nacional Albert = Virunga]), 12 May 1948, *de Wilde 55* (BR).

**Ethiopia**. **Addis Ababa**: Addis Ababa, 1956, *Mooney 6712* (K). **Amhara**: Semien, Uulkefit, Lumalumo, 10 Jul 1909, *Chiovenda 877* (FT); West Shewa region W of-Washa Forest, 3 Dec 1984, *Edwards & Tewolde 3572* (ETH); Choke Mountains, Gojjam, vicinity of the upper Ghiedeb valley, 5 Aug 1957, *Evans 413* (BM, ETH, FT, K); Debre Workto Mota, 30 Oct 1981, *Mesfin Tadese* & *Kagnew 1684* (ETH, K); Mussolini Pass, between Debre Berhan and Dobre Sina, about 200 km NNE of Addis Ababa, 23 Jul 1965, *de Wilde & de Wilde-Duyfjes 7369* (BR). **Oromia**: Menegasha State Forest, Mount Wuchacha, 48 kms due W of Addis Ababa on Ambo road, turn off just W of Menengasha village, 21 Oct 1971, *Ash 1299* (EA, K); Ghidami, Mar 1939, *Benedetto 390* (FT); Bale Zone, Dolo Mena (Masslo) to Goba, 29 Oct 1984, *Friis et al. 3610* (ETH, K); Mount Chilalo, a few kilometres S of mountain, track leading from road South of Asella to Ticho, 25 Nov 1968, *Gilbert & Gilbert 1098* (EA, K); Ticcio track; Arsi region, 28 Nov 1966, *Gilbert 138* (ETH, K); Mt. Zuquala, Nov 1994, *Hylander, K. 139* (ETH); Mount Maigudo, 24 Oct 1954, *Mooney 6156* (EA, FT, K, S); Debre Berhan, Wofasha, 23 Mar 1955, *Mooney 6475* (FT, K); ca. 10 km N of Koffale, Gobe Livestock Farm, 13 Oct 1971, *Thulin 1502* (ETH, K); Mount Wuchacha, about 15 km W of Addis Ababa, 27 Oct 1965, *de Wilde & de Wilde-Duyfjes 8480* (BR, ETH, K); Arsi, Mount Borulucciu, Asella to Ticcio, E slope of Mount Borulucciu, 6 Dec 1965, *de Wilde et al. 9213* (BR, K). **Southern Nations, Nationalities and Peoples Region**: Amaro Mountains, Mount Delo, E slope, 29 Jan 1953, *Gillett 15034* (K); road from Wondo to Agere Selam [Hagere Selam], 13 km from Wondo, 24 Jan 1968, *Westphal & Westphal 3157* (BR); Agere Selam [Hagere Selam], about 30 km S of Wondo, 21 Oct 1965, *de Wilde & de Wilde-Duyfjes 8360* (BR, ETH).

**Kenya**. **Central**: Aberdare National Park, Aberdare Mtns., ca. 1 km W of Jerusalem Gate (West), Dist. Nyandarua, 12 Jan 1975, *Croat 28375* (K, MO); Katamayu, Uplands District, Aug 1933, *Napier 5130* (K, MA); Murang’a, Tuso Fishing Camp, Oct 1932, *Jex-Blake 3304* (EA); Aberdare National Park, North Kinangop-Nyeri road, 30 Jul 1960, *Polhill 250* (BR, EA, K). **Rift Valley**: Samburu, Mount Nyiru, 30 Dec 1955, *Adamson 543* (EA, K); Samburu, Mount Nyiru, Mbarta forest zone, 29 Mar 1995, *Bytebier et al 28* (BR, EA, K); Laikipia, district around Nyasi, Lakipia Plateau and Aberdare range, 1908, *Routledge s.n.* (K); Naivasha, Naivasha-Nyeri track, 28 Oct 1934, *Taylor 1408* (BM).

**Uganda. Eastern**: Mount Elgon, Apr 1930, *Liebenberg 1637* (K); Mount Elgon, western section, 300 m E of Nabulalo, 1 km SE of Maika, 29 Mar 1993, *Sheil & Musingizi 1813* (K); Mount Elgon, Bulambuli, 6 Sep 1932, *Thanes 650* (K). **Western**: Ruwenzori Mountains, Kaleveru slopes, 6 kms west of Kilembe, 3 Jun 1970, *Katende 345 A* (EA); Mahoma, Ruwenzori Mountains 19 Jul 1960, *Kendall & Richardson 25* (EA, K); Mount Ruwenzori, below Kanyasabo rock shelter, 3 Jan 1969, *Lye, K. 1340* (K); Nyamitaba, R. Mobuku valley, 11 Jul 1952, *Osmaston 1549* (BM); Lake Mahoma, Jul 1960, *Richardson & Livingstone s.n.* (DUKE); Mobuka valley, above Kichundu, 15 Jul 1952, *Ross 578* (BM); Ruwenzori, Nyamgasani valley, Jan 1935, *Synge 1444* (BM); Toro, Ruwenzori, Namwamba valley, 6 Jan 1935, *Taylor 2913* (BM). Bwamba, Ibonde Pass, 1 Oct 1932, *Thomas 767* (EA); Nyabitaba Hut, Bujuku Valley below hut, slope down to bridge, 16 Jan 1967, *Wood 816* (EA); Ruwenzori E, Mt. Ruwenzori, 21 Feb 1906, *Wollaston s.n.* (BM).

#### Solanum sambiranense

Taxon classificationPlantaeSolanalesSolanaceae

D’Arcy & Rakot., Fl. Madag., Fam. 176: 123. 1994.

**Figure 23.**
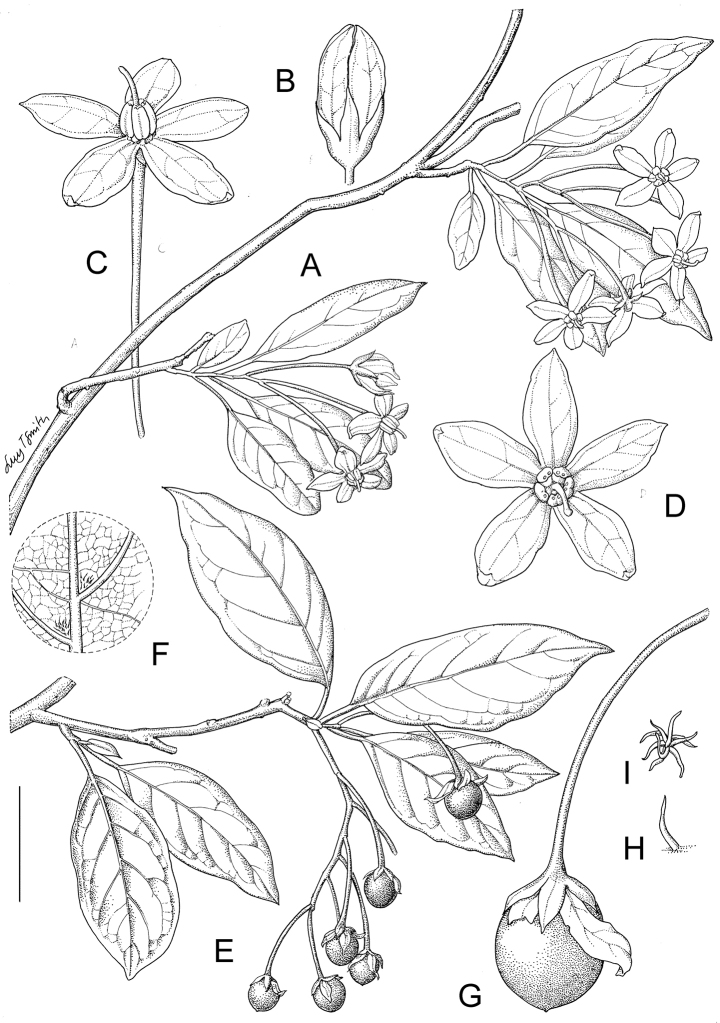
*Solanum sambiranense* D’Arcy & Rakot. **A** Flowering branch **B** Flower bud **C** Open flower from side showing spreading corolla lobes **D** Open flower from above, showing terminal anther pores **E** Fruiting branch **F** Domatia on leaf abaxial surface at junction of midvein and lateral veins **G** Immature berry **H** Dendritic trichome **I** Simple trichome. (Based on: **A** *Ranirison & Nusbaumer 1032*; **B, C** *Nusbaumer 860*; **D, E** *Randrianasolo 580*, drawn from photograph; **F–I** *Perrier 2580*). Scale bar: **A, E** = 3 cm; **B–D** = 1.5 cm; **F, G** = 1 cm; **H, I** = 0.3 mm. Drawn by Lucy T. Smith.

Figures 1D , 2A , 23

##### Type.

Madagascar. Antsiranana: vallée du Sambirano, Sep 1909, *J. Perrier de la Bâthie 2324* (lectotype, designated here: P [P00352331]; isolectotypes: P [P00352332], MO [MO-150889]).

##### Description.

Liana to 20 m high in canopy. Stems flattened to terete, glabrous or evenly pubescent with dendritic uniseriate trichomes to 0.1 mm long, glabrescent; new growth densely pubescent with dendritic trichomes. Bark of older stems longitudinally ridged, brown to almost white. Sympodial units plurifoliate, the leaves not geminate, somewhat clustered towards tips of branches or on short shoots. Leaves simple, 5–8 (10) cm long, 2.5–4 (5) cm wide, elliptic to obovate, membranous to chartaceous (occasionally thick-chartaceous on older branches), concolorous to weakly discolorous, glabrous with occasional short-branched dendritic trichomes along the midvein or sparsely pubescent on both surfaces with uniseriate dendritic to somewhat echinoid trichomes 0.2–0.5 mm mm long on the veins and lamina, these much denser at the axil of midrib and major veins forming tangled tufts (domatia); major veins 5–7 pairs, spreading at ca. 45° to the midvein and forming loops, the finer venation a prominent network of fine brown veins usually visible on both surfaces in dry material; base attenuate; margins entire; apex acute to acuminate; petiole 1–2(2.5) cm long, glabrous or densely dendritic pubescent with trichomes like those on the stem, pubescence of petiole denser than that of stem. Inflorescences terminal at the apex of short slender lateral branches, 3–6.5 cm long, unbranched or furcate, with 3–10 flowers, usually glabrous, sometimes finely pubescent with dendritic trichomes like those of the stem; peduncle 1–3 cm long; pedicels 1–2.5 cm long, apically dilated, drying ridged, usually glabrous, sometimes evenly pubescent like the rachis, articulated 0–0.5 mm from base; pedicel scars irregularly spaced 1.5–4 mm apart. Buds ellipsoid, the corolla exserted ca. halfway from calyx tube but not exserted beyond the tips of the calyx lobes before anthesis. Flowers 5-merous, apparently all perfect. Calyx tube ca. 2 mm long, an open cup, the lobes 4–10 mm long, 4–8 mm wide at base, uneven in size, deltate, acute at the tips, usually glabrous, sometimes evenly dendritic-pubescent like the rachis. Corolla 2–3.2 cm in diameter, violet or dark purple with a darker midvein, stellate, lobed almost to base, the lobes 10–13 mm long, 3–5 mm wide, ovate to linear, glabrous adaxially, usually glabrous abaxially or sometimes dendritic-pubescent like the rachis, densely papillate on the tips and margins. Stamens equal; filament tube ca. 1 mm; free portion of the filaments ca. 1.5 mm long, glabrous; anthers ca. 3.5 mm long, 1–1.5 mm wide, ellipsoid, not tightly connivent, smooth abaxially, poricidal at the tips, the pores much smaller than anther apices, ca. 0.4 mm in diameter, not lengthening with age. Ovary conical, glabrous; style ca. 9 mm long, protruding ca. 2 mm beyond the anthers, curved, glabrous; stigma capitate, dark, the surface smooth. Fruit an ovoid berry, ca. 0.9 cm long, 0.8 cm in diameter (immature), apically pointed, the pericarp thin, glabrous, drying black, but mature colour not known; fruiting pedicels 2.3–3 cm long, ca. 1 mm diameter at base, spreading, ridged; fruiting calyx lobes strongly accrescent, increasing to 1 cm long, sometimes extending further than the developing fruit, spreading (immature fruit only). Seeds not known from mature fruit.

##### Distribution

**Figure 24.**
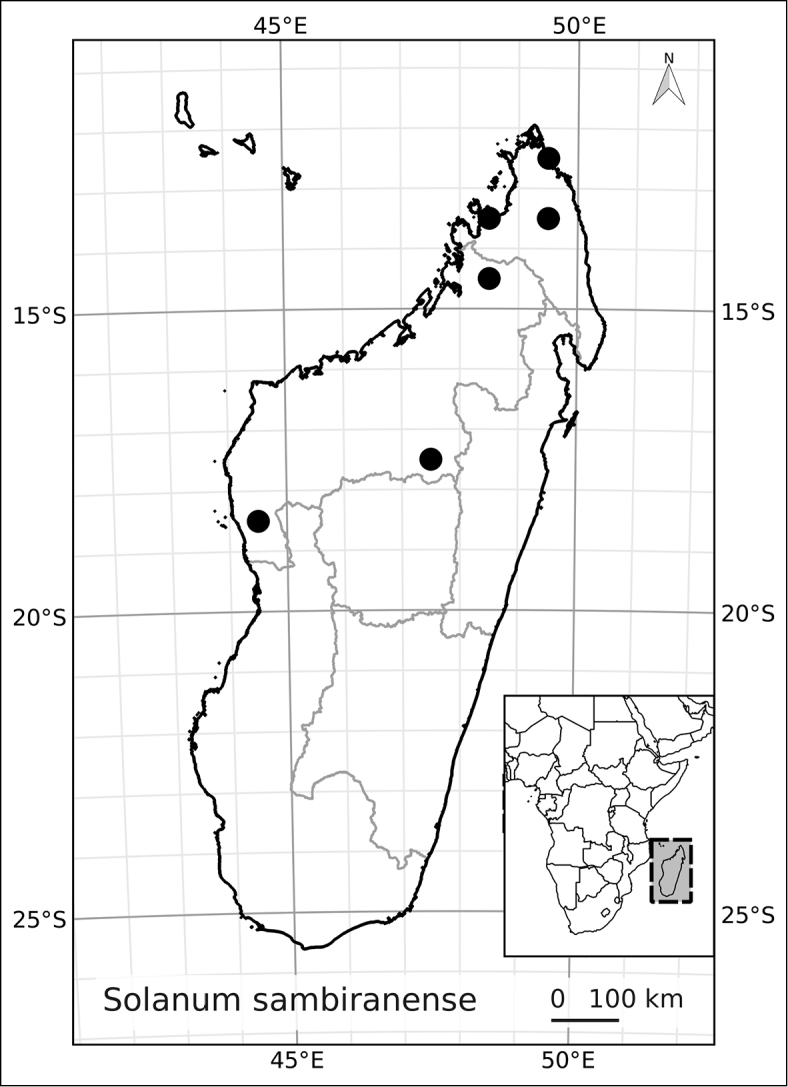
Distribution of *Solanum sambiranense* D’Arcy & Rakot.

(Figure 24). Endemic to northwestern and north-central Madagascar in the provinces of Mahajanga and Antsiranana.

##### Ecology and habitat.

Dry to subhumid woodland on limestone; 500–1500 m elevation.

##### Common names and uses.

None recorded.

##### Preliminary conservation status

(IUCN 2014). Least Concern (LC). EOO 109,250 km^2^ (LC), AOO 40 km^2^ (EN). In common with other members of the ANS clade in Madagascar, *Solanum sambiranense* has a relatively wide distribution, resulting in an EOO indicating lack of immediate conservation concern. The paucity of collections, indicative of local rarity, however, coupled with the ongoing habitat threats in Madagascar, does indicate monitoring and further collection to assess local and regional rarity is necessary.

##### Discussion.

*Solanum sambiranense* is a large-leaved liana (Figure 2A) with very large showy flowers (Figure 1D) endemic to the northern half of Madagascar. It has long petioles, long attenuate leaf bases, prominent tufts of tangled trichomes in the axils of the midrib and main leaf veins beneath (domatia, see Fig. 23), inflorescences with 3–10 flowers and foliaceous calyx lobes up to 6 mm in flower, often expanding past the developing fruit. Prior to the description of *Solanum sambiranense* in 1994 collections of this species were thought to belong to the similarly dendritic-pubescent *Solanum imamense*.

*Solanum sambiranense* is similar to *Solanum imamense* and *Solanum betroka* and could potentially also be confused with *Solanum ivohibe*. *Solanum sambiranense* can be distinguished from *Solanum imamense* by its elliptic to obovate (versus ovate) leaves 5–10 cm (versus 2.5–5 cm) long, and glabrescent leaf surface with dendritic trichomes below 0.1 mm long (versus densely pubescent leaves with indumentum reaching 0.2–0.5 mm in length). The distribution of *Solanum sambiranense* and *Solanum imamense* overlaps in north-central Madagascar even though *Solanum imamense* occupies drier areas further south. *Solanum sambiranense* differs from *Solanum betroka* by its inflorescences with 3–10 (versus 1–3) flowers, calyx lobes 4–6 mm (versus 2–3 mm) long, and leaves 5–10 cm (versus under 5 cm) long with attenuate (versus cuneate to truncate) leaf bases. *Solanum sambiranense* is morphologically similar to and potentially confusable with *Solanum betroka*: both have a clearly visible brown fine venation network, green brown leaves on herbarium specimens, membranous glabrescent leaves without thick indumentum, and a similar habit. Typical representatives of the southern *Solanum betroka* and northern *Solanum sambiranense* are clearly distinct but specimens from the centre of Madagascar are more difficult to determine; the two species occupy distinct ecological niches with *Solanum betroka* restricted to the more arid south. *Solanum sambiranense* can be distinguished from *Solanum ivohibe* by its inflorescences with 3–10 (versus 10–16) flowers, and calyx lobes 4–6 mm long tearing for up to 2 mm (versus calyx lobes 0.8–2 mm long tearing for up to 1mm); *Solanum sambiranense* also occurs further north than the only known locality of *Solanum ivohibe* in Fianarantsoa.

The distribution of *Solanum sambiranense* spans northern and northwestern Madagascar, from locations with more dry and seasonal climatic conditions than the wet eastern rainforests, warmer climate than the High Plateau, and more moisture than the south. Larger-leaved forms predominate towards the north and east as habitats get more humid.

D’Arcy and Rakotozafy (1994) state that *Solanum sambiranense* differs from other species thought to be related to it by its prominent domatia. These are dense tangled tufts of dendritic trichomes in *Solanum sambiranense*; no domatia are seen in *Solanum betroka*, but some increased trichome density in the leaf axils has been observed in *Solanum imamense* (*Baron 1754*). All specimens of *Solanum sambiranense* have membranous leaves except *Perrier de la Bâthie 13022* with membranous leaves on young shoots that become thick chartaceous on main branches. *Grevé 241* differs from the typical *Solanum sambiranense* by its densely pubescent leaves, but with foliaceous calyx lobes clearly belongs to it.

The protologue cites a holotype from P; of the two duplicates of the type collection held in Paris we here select one of these (P00352331) as the lectotype of *Solanum sambiranense* as it bears a note “type collection” in D’Arcy’s handwriting, while the other duplicate of *Perrier de la Bâthie 2324* (P00352332) has no additional annotation.

##### Selected specimens examined.

**Madagascar. Antsiranana**: Ambanja, Sambirano, bassin supérieur du Sambirano, berges du Sambiran, 1937, *Humbert 18586* (P); Ambanja, Sambirano, bassin supérieur du Sambirano: forêt de Besanatribe, 1937, *Humbert 18709* (P); Antsiranana Rural, Diego-Suarez, a la limite N des collines et plateaux calcaires de l’Ankarana, s.d., *Humbert 19072* (MO, P); Ambanja, Tsaratanana, Mont Tsaratanana, 14°01’ S, 48°58’ E Alt: 1000m, 1000 m, Dec 1912, *Perrier de la Bâthie, J.M.H.A. 2580* (P); Vohemar, Dairaina, forêt de Binara, a 1370 m du point côté 779, 14 Dec 2005, *Ranirison & Nusbaumer 1032* (G, K); canton de Marovato, district d’Ambanja, Réserve naturelle 4 [Tsaratanana], 12 Dec 1953, *Réserves Naturelles Madagascar 5766* (MO, NY, P, TAN). **Mahajanga**: Beanka, partie sud, Kinahango, 23 Nov 2011, *Gautier et al. 5707* (G); Tsiampihy, district d’Antsalova, 15 Oct 1932, *Léandri 297* (NY, P); Maevatanana, Mahatsinjo, au sud d’Andriba, Feb 1920, *Perrier de la Bâthie 13022* (P); Maevatanana, Mahatsinjo, au Nord du Tampoketsa d’Ankazobe, 1000 m, Sep 1926, *Perrier de la Bâthie 17783* (P).

#### Solanum terminale

Taxon classificationPlantaeSolanalesSolanaceae

Forssk., Fl. Aegypt.-Arab. 45. 1775.

**Figure 25.**
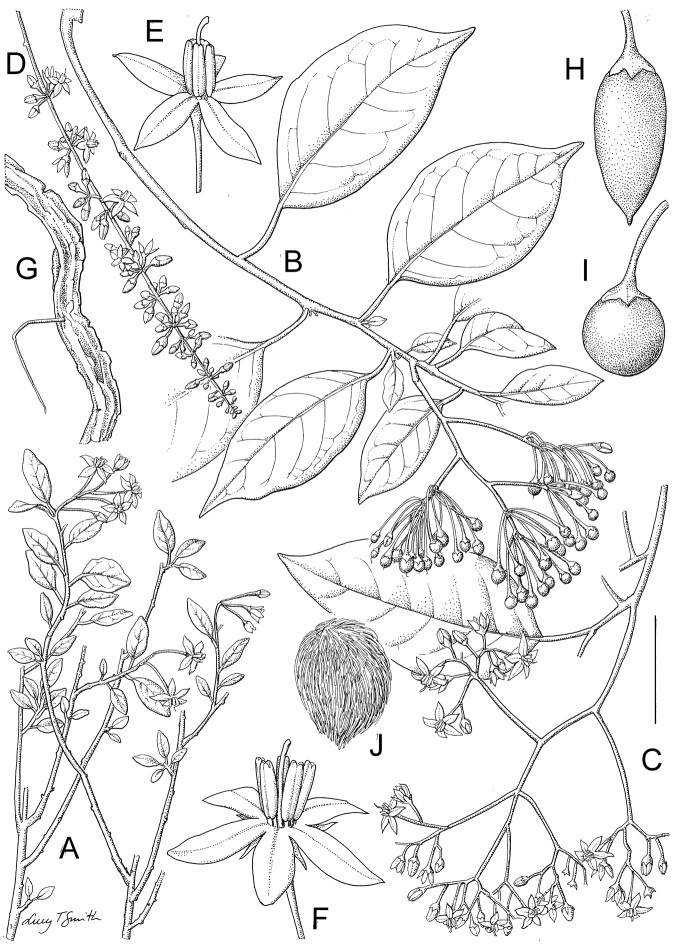
*Solanum terminale* Forssk. **A** Flowering branch of savanna form (nakurense-type) **B** Fruiting branch of climbing plant with open infructescence with globose berries (terminale-type) **C** Flowering branch of climbing plant with open inflorescence **D** Flowering branch of climbing plant with narrow, spicate inflorescence (welwitschii-type) **E** Flower with fused anther cone (welwitschii-type) **F** Flower with non-fused anther cone (terminale-type) **G** Corky bark of older stems **H** Fusiform mature berry **I** Globose mature berry **J** “Hairy” seed from mature fruit showing elongate lateral cell walls. (Based on: **A** *Ash 2932*; **B** *Drummond 3155*; **C** *Gilbert & Friis 8417*; **D, E** *Etuge & Thomas 31*; **F** *Greenway & Kanuri 13883*; **G** *Synnot 617*; **H, J** *Mbani 142*; **I** *Mbatchou 21*). Scale bar: **A, C, D, G** = 3 cm; **B** = 4 cm; **E, F, H, I** = 7 mm; **J** = 2 mm. Drawn by Lucy T. Smith.

Figures 1E , 2C & D , 25

Solanum bifurcatum Hochst. ex A.Rich., Tent. Fl. Abyss. 2: 98. 1851. Type. Ethiopia. Tigray: Adoa [Adwa], 14 Jun 1837, *G.H.W. Schimper 201* (lectotype, designated by Lester 1997, pg. 281; P [P00344566]; isolectotypes: BM [BM000847558], BR [BR0000006289041, BR0000008422248], E [E00193277], G [G00301682, G00343569], G-DC [G00144683], GH [GH00139638], GOET [GOET003592], HBG [HBG511409], HOH [HOH009830], LE [3 sheets], K [K000232603], M [M0105581, M0105582], MPU [MPU011243, MPU011244], NY [NY00169749], OXF, P [P00344587, P00341599, P00344570, P00344567], PAL, REG [REG000401], S [S-09-33851, S-09-33853, S-115601], STU [STU000029, STU000028], U [U0113932], US [US-945142, barcode US00730876], W [W0000632, W1889-0283815]).
Solanum bifurcum Hochst. ex Dunal, Prodr. [A. P. de Candolle] 13(1): 77. 1852, nom. illeg. superfl., orthographic variant. Type. Based on Solanum bifurcatum A.Rich.
Solanum inconstans C.H.Wright, Bull. Misc. Inform. Kew 1894: 127. 1894. Type. Equatorial Guinea. Bioco: “Fernando Po”, Dec 1859, *G. Mann 62* (lectotype, designated here: K [K000414056]; isolectotype: P [P00341583]).
Solanum phytolaccoides C.H.Wright, Bull. Misc. Inform. Kew 1894: 126. 1894. Type. Ethiopia. [Amhara/Tigray]: “ex Tigrè v. Begemder”, 24 Sep 1862/13 Sep 1863, *G.H.W. Schimper 310* (lectotype, designated here: K [K000232602]; isolectotypes: BM [BM000838174, BM000838175], E [E00526857], US [US-806525]).
Solanum welwitschii C.H.Wright, Bull. Misc. Inform. Kew 1894: 126. 1894. Type. Angola. Cuanza Norte: Golungo Alto, *F.M.J. Welwitsch 6098* (lectotype, designated here: BM [BM000838177]; isolectotype: K [K000414110], LISU [LISU220797]).
Solanum welwitschii C.H.Wright var. oblongum C.H.Wright, Bull. Misc. Inform. Kew 1894: 127. 1894. Type. Cameroon. Sud-Ouest: Ambas Bay, *G. Mann 10* [s.n., No. X] (lectotype, designated by Edmonds 2012, pg. 113: K [K000414053]; isolectotype: P [P00331492]).
Solanum welwitschii C.H.Wright var. strictum C.H.Wright, Bull. Misc. Inform. Kew 1894: 127. 1894. Type. Equatorial Guinea. Bioco: “Fernando Po”, 1860, *G. Mann 274* (lecctotype, designated here: K [K000414054]; isolectotype: P [P00331491].
Solanum campanuliflorum C.H.Wright, Bull. Misc. Inform. Kew 1894: 127. 1894. Type. Angola. Cunene: Cunene [River], Sep 1883, *H.H. Johnston s.n.* (lectotype, designated here: K [K00414073]).
Solanum nakurense C.H.Wright, Bull. Misc. Inform. Kew 1897: 275. 1897. Type. Kenya. Rift Valley: Masai [Nakuru ex protologue], 1893, *G.F. Scott-Elliot 6800* (holotype: K [K000096900]; isotype: BM [BM000847730]).
Solanum symphyostemon De Wild. & T.Durand, Ann. Mus. Congo, Sér. 2, Bot., ser. 2, 1: 44. 1899. Type. Democratic Republic of the Congo. Équateur: Bolombo, près de Ngala (Ngali), 21 Sep 1986, *F. Thonner 96* (lectotype, designated here: BR [BR0000008993137]; isolectotypes: BR [BR0000008994462], K [K000414043]).
Solanum lujaei De Wild. & T.Durand, Bull. Soc. Roy. Bot. Belgique 38: 209. 1899. Type. Democratic Republic of the Congo. Bas-Congo: “Sona Gunga, cataractes”, 21 Nov 1898, *É. Luja 112* (lectotype, designated here: BR [BR0000014800313]).
Solanum togoense Dammer, Bot. Jahrb. Syst. 38: 59. 1906 [“1907”]. Type. Togo. Maritime: Badja, Mar 1900, *F.R.R. Schlechter 12974* (lectotype, designated by Edmonds 2012, pg. 110: K [K000414055]; isolectotypes: BM [BM000838103], BR [BR0000006288921], P [P00341582]).
Solanum buchwaldii Dammer, Bot. Jahrb. Syst. 38: 180. 1906 [“1907”]. Type. Tanzania. Tanga: Usambara [Mtns., Lushoto District], Muafa, Apr [1900], *J. Buchwald 542* (lectotype, designated by Bitter 1917, pg. 450: B, destroyed, no duplicates found).
Solanum bilabiatum Dammer, Bot. Jahrb. Syst. 38: 181. 1906 [“1907”]. Type. São Tomé and Principe. São Tomé: Sin. loc., 2-7 m, [1885], *A.F. Moller 146* (type in B [?], destroyed; no duplicates found).
Solanum comorense Dammer, Bot. Jahrb. Syst. 38: 181. 1906 [“1907”]. Type. Mayotte (French Overseas Department). Maore: forêt du Combani, 6 Oct 1884, *L. Humblot [1]284* (neotype, designated by D’Arcy & Rakatozafy 1994, pg. 126: P [P00184319]; isoneotypes: BM [BM000887171], K [K000788514]).
Solanum laurentii Dammer, Bot. Jahrb. Syst. 38: 182. 1906 [“1907”], nom. illeg., non Solanum laurentii De Wild., 1905. Type. Democratic Republic of the Congo. Sin. loc., “*Laurent s.n.*” (type at B [?], destroyed; no duplicates found). Democratic Republic of the Congo. Équateur: environs d’Eala, 11 May 1905, *M. Laurent 701* (neotype, designated here: BR [BR0000014801273]).
Solanum plousianthemum Dammer, Bot. Jahrb. Syst. 38: 180. 1906 [“1907”] Type. Tanzania. “Usambara, im Gebüsch niederer Hügel”, Jul 1892, *C. Holst 3731* (lectotype, designated by Bitter 1917, pg. 457): B, destroyed; no duplicates found).
Solanum suberosum Dammer, Bot. Jahrb. Syst. 38: 182. 1906 [“1907”]. Type. Cameroon. Sud-Ouest: Barombi Station between Station and Ninga town, Mar 1889, *G.R Preuss 18* (type, B [?], destroyed; no duplicates found); Barombi-Bache, SW of Station, 1400 m, Apr 1889, *G.R. Preuss 171* (type, B [?], destroyed; no duplicates found); Buea, 1400 m, May 1891, *G.R Preuss 885* (type, B [?], destroyed; no duplicates found); Buea Station, 1000 m, Apr 1898, *Lehmbach 211* (type, B [?], destroyed; no duplicates found); Yaounde, May 1894, *G.A. Zenker & A. Staudt 328* (type, B [?], destroyed; no duplicates found); Yaounde, Apr 1890, *G.A. Zenker 268* (type, B [?], destroyed; no duplicates found);northern Cameroon, Bangwe, Jun, *G. Conrau 200* (type, B [?], destroyed; no duplicates found).
Solanum preussii Dammer, Bot. Jahrb. Syst. 38: 183. 1906 [“1907”]. Type. Cameroon. Sud-Ouest: “Barombi Station”, Aug 1890, *P.R. Preuss 397* (lectotype, designated here: P [P00331480]; isolectotype: E [E00193273]).
Solanum lykipiense C.H.Wright, Fl. Trop. Afr. [Oliver et al.] 4, 2: 220. 1906. Type. Kenya. Rift Valley: Lykypia, 6000-8000 ft., *J. Thomson s.n.* (lectotype, designated here: K [K000096902]).
Solanum mangaschae Pax, Bot. Jahrb. Syst. 39: 648. 1907. Type. Ethiopia. Southern Nations, Nationalities and Peoples: “Amaniel am Gazerit”, 2300, 2 Apr 1905, *F. Rosen s.n.* (holotype: WRSL, not seen).
Solanum subcoriaceum T.Durand & H.Durand, Bull. Jard. Bot. État Bruxelles 2: 394. 1909. Type. Based on and nom. nov. for Solanum laurentii Dammer, not Solanum laurentii De Wild.
Solanum leucanthum Dammer, Wiss. Ergebn. Deut. Zentr.-Afr. Exped. (1907–1908), Bot., 2: 284. 1911 [“1914”]. Type. Rwanda. Western: Rukarara, Rugege Forest, 2900 m, Aug 1907, *G. Mildbraed 894* (B [?], destroyed; no duplicates found); “Kissenye, Bugoyer, Hügelland, Hecken”, 2100 m, 27 Oct 1907, *G. Mildbraed 1431a* (B [?], destroyed; no duplicates found).
Solanum aculeolatum Dammer, Bot. Jahrb. Syst. 48: 237. 1912, nom. illeg., non Solanum aculeolatum M.Martens & Galeotti, 1845. Type. Kenya. “Escarpment, in Lichtungen”, 2500 m, Feb 1903, *F. Thomas s.n.* (type in B [?], destroyed; lectotype, designated here: E [E00193280]).
Solanum penduliflorum Dammer, Bot. Jahrb. Syst. 48: 255. 1912. Type. Kenya. Rift Valley: “Mau Plateau, common railway cuttings and open places, Molo, Ravine, Londiani and Njoro”, 2300-3000 m, *G.S. Baker 133* (type in B [?], destroyed; no duplicates found).
Solanum bansoense Dammer, Bot. Jahrb. Syst. 48: 237. 1912. Type. Cameroon. Nord-Ouest: “Bansso-Gebirge”, 1700 m, Oct 1909, *C.L. Ledermann 5778* (type in B [?], destroyed; no duplicates found).
Solanum massaiense Bitter, Repert. Spec. Nov. Regni Veg. 11: 18. 1912. Type. Based on and nom. nov. for Solanum aculeolatum Dammer, not Solanum aculeolatum M.Martens & Galeotti.
Solanum holtzii Dammer, Bot. Jahrb. Syst. 53: 329. 1915. Type. Tanzania. Morogoro: “Uluguru, Bez. Morogoro, Waldreservat Banduki II”, Mar 1913, *W. Holtz 3148* (type in B [?], destroyed; no duplicates found).
Solanum rhodesianum Dammer, Bot. Jahrb. Syst. 53: 326. 1915. Type. Zimbabwe. Manicaland: Melener, Chirinda Forest, 9 Oct 1905, *C.F.M. Swynnerton 86* (lectotype, designated here: K [K000414111]; isolectotypes: BM [BM000838178], K [K000414112]).
Solanum stolzii Dammer, Bot. Jahrb. Syst. 53: 327. 1915. Type. Tanzania. Mbeya: Rungwe Crater, Kyimbila Station, 2000 m, 19 Dec 1911, *A. Stolz 1035* (lectotype, designated here: M [M0105595]; isolectotypes: B [B 10 0165205, received 1990], C [C10003110], G [G00343580], K [K000096901], S [S09-36468], W [W19150004892].
Solanum meyeri-johannis Dammer, Bot. Jahrb. Syst. 53: 328. 1915. Type. Tanzania. “Ussagara, Bez. Kilossa, Bugaberge” Nov-Dec 1911, *Dr. Houy 1242* (type in B [?], destroyed; no duplicates found).
Solanum lateritium Dammer, Bot. Jahrb. Syst. 53: 325. 1915. Type. Tanzania. Mbeya: Kyimbila Station, 600-800 m, Aug 1912, *A. Stolz 1514* (lectotype, designated here: JE [JE00004286]; isolectotypes: G [G00442975], K [K000413992], S [S-G-5699], U [U0113933], WAG [WAG0003361], W [W19150008146]).
Solanum leucanthum Bitter & Dammer, Bot. Jahrb. Syst. 54: 456. 1917, nom. illeg., non Solanum leucanthum Dammer, 1911. Type. Rwanda. Western: Rukarara, Rugege Forest, 2900 m, Aug 1907, *G. Mildbraed 894* (no herbarium cited, B [?], destroyed; no duplicates found).
Solanum hemisymphyes Bitter, Bot. Jahrb. Syst. 54: 477. 1917. Type. Democratic Republic of the Congo. Nord-Kivu: Kwa Muera, Fort Beni, Jun 1908, *J. Mildbraed 2238* (type in B [?], destroyed; no duplicates found).
Solanum nakurense C.H.Wright var. lykipiense (C.H.Wright) Bitter, Bot. Jahrb. Syst. 54: 449. 1917. Type. Based on Solanum lykipiense C.H.Wright
Solanum plousianthemum Dammer subsp. holtzii (Dammer) Bitter, Bot. Jahrb. Syst. 54: 467. 1917. Type. Based on Solanum holtzii Dammer
Solanum plousianthemum Dammer subsp. kasima Bitter, Bot. Jahrb. Syst. 54: 468. 1917. Type. Tanzania. Mbeya: Neu-Langenburg, Kyimbila, 1350 m, Oct 1912, *A. Stolz 355* (holotype: B, destroyed; lectotype, designated here: G [G00070223]; isolectotype: K [K000413993]).
Solanum plousianthemum Dammer var. angustifrons Bitter, Bot. Jahrb. Syst. 54: 462. 1917. Type. Tanzania. Kilimanjaro: Mount Kilimanjaro, Marangu, 1500 m, Apr 1894, *G. Volkens 2109* (type in B [?], destroyed; no duplicates found).
Solanum plousianthemum Dammer var. buchwaldii (Dammer) Bitter, Bot. Jahrb. Syst. 54: 459. 1917. Type. Based on Solanum buchwaldii Dammer
Solanum plousianthemum Dammer var. commixtum Bitter, Bot. Jahrb. Syst. 54: 464. 1917. Type. Tanzania. Kilimanjaro: Mount Kilimanjaro, ca. 1600 m, May 1894, *H.H.Johnston s.n.* (lectotype, designated here: K [K000413995]; isolectotype: BM [BM000838176]).
Solanum plousianthemum Dammer var. conglutinans Bitter, Bot. Jahrb. Syst. 54: 461. 1917. Type. Tanzania. Tanga: Usambaras, Kwa Mshuza, “Handei”, 1570 m, Aug 1893, *C. Holst 8927* (lectotype, designated here: P [P00341605]; isolectotypes: COI [COI00077085], K [K000413989], LE, W [W18940006582]).
Solanum plousianthemum Dammer var. devians Bitter, Bot. Jahrb. Syst. 54: 466. 1917. Type. Rwanda. “Mpororo (Rufua), Issenge-Posten, an nordlichen Zuflussen Kagera”, Jul 1907, *J. Mildbraed 336* (type in B [?], destroyed; no duplicates found).
Solanum plousianthemum Dammer var. endosiphonotrichum Bitter, Bot. Jahrb. Syst. 54: 465. 1917. Type. Rwanda. Southern: Mount Niansa [Nyanza], 1700 m, *Dr. Kandt 147* (holotype: B, destroyed; no duplicates found).
Solanum plousianthemum Dammer var. microstelidium Bitter, Bot. Jahrb. Syst. 54: 461. 1917. Type. Rwanda. Western: Lake Kivu, Lubengera, Mugarura Island, *H. Meyer 909* (type in B [?], destroyed; no duplicates found).
Solanum plousianthemum Dammer var. epapillosum Bitter, Bot. Jahrb. Syst. 54: 464. 1917. Type. Tanzania. Kilimanjaro: Mount Kilimanjaro, Useri, 2200 m, Mar 1894, *G. Volkens 1991* (type in B [?], destroyed; no duplicates found).
Solanum plousianthemum Dammer var. gracilifilum Bitter, Bot. Jahrb. Syst. 54: 463. 1917. Type. Tanzania. Kilimanjaro: Mount Kilimanjaro, Marangu, 2000 m, May 1894, *G. Volkens 2265* (lectotype, designated here: BR [BR0000006495794]).
Solanum plousianthemum Dammer var. rhodesianum (Dammer) Bitter, Bot. Jahrb. Syst. 54: 461. 1917. Type. Based on Solanum rhodesianum Dammer
Solanum plousianthemum Dammer var. subtusbarbellatum Bitter, Bot. Jahrb. Syst. 54: 467. 1917. Type. Kenya. Eastern: [Kitui County], Galunka [Galinka], 25 May 1902, *L.C.T. Kässner 804* (holotype: B, destroyed; lectotype, designated here: BM [BM000847733]; isolectotype: K [K000788864]).
Solanum plousianthemum Dammer var. ugandae Bitter, Bot. Jahrb. Syst. 54: 460. 1917. Type. Uganda. Sin. loc., 1 Jan 1891, *F. Stuhlmann 1329* (lectotype, designated here: K [K000413994])..
Solanum plousianthemum Dammer var. kundelunguense Bitter, Bot. Jahrb. Syst. 54: 467. 1917. Type. Democratic Republic of the Congo. Katanga: Kundelungu, 15 May 1908, *L.C.T. Kässner 2760* (holotype: B, destroyed; lectotype, designated here: BM [BM000847803]; isolectotypes: K [K000414041], P [P00341576]).
Solanum ruandae Bitter, Bot. Jahrb. Syst. 54: 471. 1917. Type. Rwanda/Democratic Republic of the Congo: Kissenye, “Bugoyer Hügelland, Hecken”, 2100 m, 27 Oct 1907, *J. Mildbraed 1431a* (holotype: B, destroyed; no duplicates found).
Solanum suberosum Dammer var. calvum Bitter, Bot. Jahrb. Syst. 54: 486. 1917. Type. Cameroon. Sud-Ouest [?]: “Nordwestkamerum, Spitze des Kamerumberges” [Mount Cameroon], *A. Weberbauer 38, 60* (syntypes: B, destroyed; no duplicates found).
Solanum suberosum Dammer var. ramosivelutinum Bitter, Bot. Jahrb. Syst. 54: 486. 1917. Type. Democratic Republic of the Congo. Orientale: Ituri, Irumu-Mawambi, Apr, *J. Mildbraed 3038* (type in B [?], destroyed; no duplicates found).
Solanum sychnoteranthum Bitter, Bot. Jahrb. Syst. 54: 472. 1917. Type. Democratic Republic of the Congo[?]. “lava plain”, 18 Aug 1908, *L.C.T. Kässner 3250* (holotype: B, destroyed; lectotype, designated here: E [E00193274]; isolectotypes: BM [BM000847732], P [P00331494]).
Solanum plousianthemum Dammer var. angustatum Bitter, Repert. Spec. Nov. Regni Veg. 18: 302. 1922. Type. Tanzania. Morogoro: “Ulugurus”, 19 Oct 1013, *W. von Brehmer 891* (no herbarium cited; no duplicates found).
Solanum plousianthemum Dammer var. kyimbilense Bitter, Repert. Spec. Nov. Regni Veg. 18: 302. 1922. Type. Tanzania. Mbeya: Bex, Neu-Langenburg, Kyimbila, near Madehani, 2000 m, 3 Dec 1913, *A. Stolz 2315* (lectotype, designated here: K [K000413990]; isolectotypes: A [A00139637, A00219304], BM [BM000838180], BR [BR0000006495107], CAS [CAS- 130438], K [K000414991], P [P00341613]).
Solanum bansoense Dammer var. episporadotrichum Bitter, Repert. Spec. Nov. Regni Veg. 18: 303. 1922. Type. Cameroon. Est: at junction of Sanaga [River?] with the Djerem [River], (“hylaea um Deng-Deng”), 250 km NE of Yaounde, between Mbo’s and Sardi, southwest of Deng-Deng, 8 Mar 1914, *G. Mildbraed 8555* (holotype: B, destroyed; no duplicates found).
Solanum bansoense Dammer subsp. sanaganum Bitter, Repert. Spec. Nov. Regni Veg. 18: 304. 1922. Type. Cameroon. Est: at junction of Sanaga [River?] with the Djerem [River], (“hylaea um Deng-Deng”), 250 km NE of Yaounde, Mar 1914, *G. Mildbraed 8619* (holotype: B, destroyed; lectotype, designated here: K [K000414042]).
Solanum welwitschii C.H.Wright var. laxepaniculatum Bitter, Repert. Spec. Nov. Regni Veg. 18: 306. 1922. Type. Cameroon. Est: [Upper Nyong District], Lomie District, Dscha region, in Tugumedjo’s village, 13°30-12°25 W latitude, 18 May 1911, *G. Mildbraed 5265* (holotype: B, destroyed; lectotype, designated here: HBG [HBG511510]).
Solanum lianiforme De Wild., Pl. Bequaert. 1: 427. 1922. Type. Democratic Republic of the Congo. Nord-Kivu: Walikale, 7 Jan 1915, *J. Bequaert 6531* (lectotype, designated here: BR [BR0000008993489]; isolectotypes: BR [BR0000008994448, 2 sheets]).
Solanum butaguense De Wild., Pl. Bequaert. 1: 424. 1922. Type. Democratic Republic of the Congo. Nord-Kivu: Butagu, Ruwenzori, ca. 2200 m, 13 Apr 1914, *J. Bequaert 3616* (lectotype, designated here: BR [BR0000008994431]; isolectotypes: BR [BR0000008993502, BR0000008993472]).
Solanum balboanum Chiov., Racc. Bot. Miss. Consol. Kenya 87. 1935. Type. Kenya. Central: Tusu, 1 Feb 1911, *P.G. Balbo 579* (lectotype, designated here: FT [FT003031]).
Solanum terminale Forssk. subsp. inconstans (C.H.Wright) Heine, Kew Bull. 14: 247. 1960. Type. Based on Solanum inconstans C.H.Wright Solanum terminale Forssk. subsp. sanaganum (Bitter) Heine, Kew Bull. 14: 248. 1960. Type. Based on Solanum bansoense Dammer subsp. sanaganum Bitter
Solanum terminale Forssk. subsp. welwitschii (C.H.Wright) Heine, Kew Bull. 14: 248. 1960. Type. Based on Solanum welwitschii C.H.Wright

##### Type.

yemen. “Mokhaja” [Mukhajah =Jebel Barad], *P. Forsskål s.n.* (lectotype, designated here: C [C10003105] Herb. Forsskal 419; isolectotype: C [C10003106] Herb. Forsskal 406).

##### Description.

Shrub to high-climbing woody liana, from ca. 1 m tall to canopy height. Stems terete, to 10 cm in diameter in liana forms, glabrous with a few simple uniseriate or dendritic trichomes to densely pubescent with simple and/or dendritic uniseriate trichomes, these soon deciduous; new growth sparsely to densely pubescent with simple and/or dendritic 4–6-celled uniseriate trichomes 0.5–1 mm long. Bark of older stems pale yellowish white, corky on larger woody stems > 0.5 cm in diameter. Sympodial units plurifoliate, the leaves not geminate, evenly distributed along branches. Leaves simple, (1.5)4–16 cm long, (1)2–8 cm wide, very variably sized on individual stems, elliptic, the adaxial surfaces glabrous to sparsely pubescent with simple or less commonly dendritic uniseriate trichomes 0.5–1 mm long, these denser on the veins, the abaxial surfaces glabrous to densely and evenly pubescent with dendritic and/or simple uniseriate 4–8-celled trichomes 1–1.5 mm long; major veins 5–7 pairs, usually somewhat yellowish in dry material; base acute to truncate, very occasionally somewhat cordate, not decurrent on the petiole; margins entire; apex acute to acuminate; petiole (0.5)2–7(11) cm long, highly variable from leaf to leaf even in leaves of the same size, sparsely to densely pubescent with simple and/or dendritic trichomes like those of the leaves. Inflorescences terminal, 1–20(+) cm long, many times branched, in some forms the branches very short and the inflorescence appearing spicate, with (5)10–100 flowers in clumps at the ends of branches, glabrous or sparsely pubescent with simple and/or dendritic uniseriate trichomes ca. 0.5 mm long, these denser at the base of flower clumps; peduncle to 10 cm long; pedicels 0.5–1 cm long, ca. 0.5 mm in diameter at the base and apex, glabrous to sparsely pubscent in parallel with the rest of the inflorescence, spreading at anthesis, articulated at the base leaving a tiny raised lump on the rachis when flower falls; pedicel scars in tightly packed clusters at the ends of long branches or along the main axis. Buds ovoid to narrowly elliptical (see commentary), the corolla strongly exserted from the calyx tube long before anthesis. Flowers 5-merous, apparently all perfect. Calyx tube 1–1.5 mm long, open-conical, the lobes 1–2 mm long, deltate with acuminate tips (exceptionally to 4 mm long in *Strid 3113*), glabrous or pubescent with simple or dendritic trichomes like those of the inflorescence axis. Corolla 1–2.5 cm in diameter, lilac to white with varying degrees of lilac towards the centre, deeply stellate, divided 3/4 to nearly to the base, the lobes 5–9 mm long, 2–2.5 mm wide, narrowly ligulate, spreading to reflexed at anthesis, the adaxial surface glabrous or densely pubescent/papillate with 2–3-celled simple trichomes, the abaxial surface densely pubescent with 2–3-celled weak-walled simple trichomes, these denser along the margins and at the tips, sometimes purple-tinged, the corolla on herbarium specimens with a mealy appearance. Stamens equal; filament tube <0.1 mm long to absent; free portion of the filaments 0.5–1 mm long, glabrous; anthers (3-)3.5–5 mm long, 1–2 mm wide, ellipsoid to narrowly ellipsoid, loosely connivent to tightly fused due to internal papillae, smooth or papillate abaxially, somewhat sagittate at the base, poricidal at the tips, the pores lengthening to slits with age. Ovary globose, glabrous or occasionally minutely hispidulous with short erect uniseriate trichomes (*Gossweiler s.n.*, coll. 1910); style 8–10 mm long, exserted beyond the anthers approximately equal to the anther length, glabrous; stigma capitate, the surfaces minutely papillate. Fruit a globose to elongate and fusiform berry, 5–7 mm in diameter, bright red when ripe, the pericarp very thin and shiny; fruiting pedicels 0.5–1.5 cm long, ca. 1 mm in diameter, spreading at anthesis; fruiting calyx lobes not markedly enlarging. Seeds 12–20 per berry, 1.5–2.5 mm long, 1–2 mm wide, ovoid reniform, reddish brown, the surfaces deeply pitted, the testal cells pentagonal to rectangular, the lateral cell walls composed of elongate filamentous projections to 0.5 mm long, giving the seeds a hairy appearance.

##### Distribution

**Figure 26.**
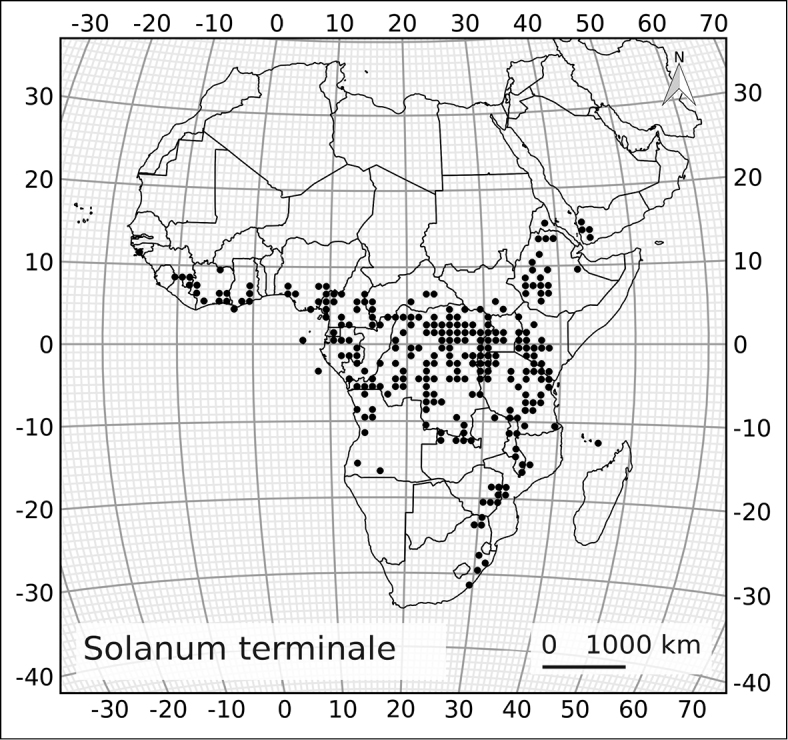
Distribution of *Solanum terminale* Forssk.

(Figure 26). Widely distributed in continental sub-Saharan Africa (see also Table 2); also found in the Comoro Islands and on the Arabian peninsula in Yemen. We have seen specimens from Angola, Burundi, Cameroon, Central African Republic, Comoros, Côte d’Ivoire, Democratic Republic of the Congo, Equatorial Guinea, Eritrea, Ethiopia, Gabon, Ghana, Guinea, Guinea Bissau, Kenya, Liberia, Malawi, Mozambique, Nigeria, Republic of Congo, Rwanda, São Tome e Principe, Sierra Leone, South Africa, South Sudan, Tanzania, Togo, Uganda, Yemen, Zambia, and Zimbabwe.

##### Ecology and habitat.

A wide variety of forest habitats; also from savannah; near sea level to 3300 m elevation. Swamp forests, humid forest, sclerophyllous woodland, grassland savannah. Plants growing in the open in savanna habitats area shorter and more shrub-like than those of forest edges or interiors.

##### Common names and uses.

Many common names are recorded on herbarium specimens of *Solanum terminale*, some with the language, and some without. We have listed here those that occur multiple times, with a single voucher for each. All common names associated with individual specimens can be found in the data on the Natural History Museum Data Portal (http://dx.doi.org/10.5519/0039445) and on the Solanaceae Source website (http://www.solanaceaesource.org). Burundi. agatoretore (Kirundi language, *Michel 987*), umuhanuraguba (*Becquet 861*); Democratic Republic of the Congo. belele (Lombo language, *Louis 981*), belemba (Lombo language, *Louis 6362*), itotofo (Lombo language, *Jeanty 48*), itotofo i boliki (Lombo language, *Louis 14146*), kampako (Bofunda language, *Corbisier-Baland 1034*), kedekede (*Giorgi 745*), kongalo n pako (Ngala language, *Claessens 453*), mampoko (Mbole language, *Leonard 802*), muhwehwe (Tembo language, *Troupin 10162*), qwadja (*Reygaert 226*), umuranuhankuba (Kinyarwanda language, *Spitaels 119*); Kenya. mutandambuga (Meru language, *Graham 1752*); Rwanda. umuranuhankuba (Kinyarwanda language, *Troupin 6631*); Tanzania. kadaru (Bantu language, *Koritschoner 866*), luvuvu (Kirangi language, *Burtt 1141*); Uganda. omuhanurankuba (Nkore-Kiga language, *Purseglove P-1654*). The fruit said to be poisonous and the leaves eaten in the Central African Republic (Burkhill 2000).

##### Preliminary conservation status

(IUCN 2014). Least Concern (LC). EOO 21,154,570 km^2^ (LC), AOO 2,316 km^2^ (NT). *Solanum terminale* is widespread across continental Africa in a wide variety of habitats, and occurs in several protected areas. The near threatened status suggested by the AOO calculation is likely due to collection bias; *Solanum terminale* is a very common plant. Genetic differentiation across this wide range, however, may be important for population level conservation or national conservation priorities.

##### Discussion.

*Solanum terminale* is the most widespread and common species of the ANS clade on the African mainland, and is often recognised as three distinct entities; *Solanum nakurense*, *Solanum terminale* s.s. and *Solanum welwitschii* (e.g., Edmonds 2012). Bitter (1917, 1922) recognised 20 species and 23 infraspecific taxa, while Heine (1960) amalgamated all these into a single species with three infraspecific taxa in western Africa, and suggested that a similar course of action needed to be taken throughout the continent. We are recognising these populations as a single species because the characters used to distinguish them are continuously variable and field observations indicate that habitat plays a significant role in overall morphology (see below).

*Solanum terminale* is not broadly sympatric with any other species of the ANS clade, although in eastern South Africa *Solanum africanum* also occurs, but not strictly sympatrically; *Solanum runsoriense* also occurs in the same regions as *Solanum terminale* but usually at higher elevations. *Solanum terminale* is easily distinguished from others in the clade by its flowers with densely papillate abaxial corolla lobes, flowers clustered at the ends of inflorescence branches (of varying length) and bright red berries. Populations from western Africa tend to have shorter inflorescence branches (see Fig. 25D), tightly connate anthers and elongate berries and have been recognised as *Solanum welwitschii*, while those from eastern Africa have more open inflorescences (see Fig. 25B, C), more loosely connate anthers and globose berries and have been called *Solanum terminale*. Small plants with few-flowered inflorescences primarily from eastern Africa have been called *Solanum nakurense* (Fig. 25A). The leaves of *Solanum terminale* are usually pubescent to some degree, but are occasionally completely glabrous (e.g., the type of *Solanum welwitschii*). Dense tufts of trichomes in the vein axils (domatia) do not occur in *Solanum terminale*, but the pubescence is often denser near the midvein.

**Figure 27.**
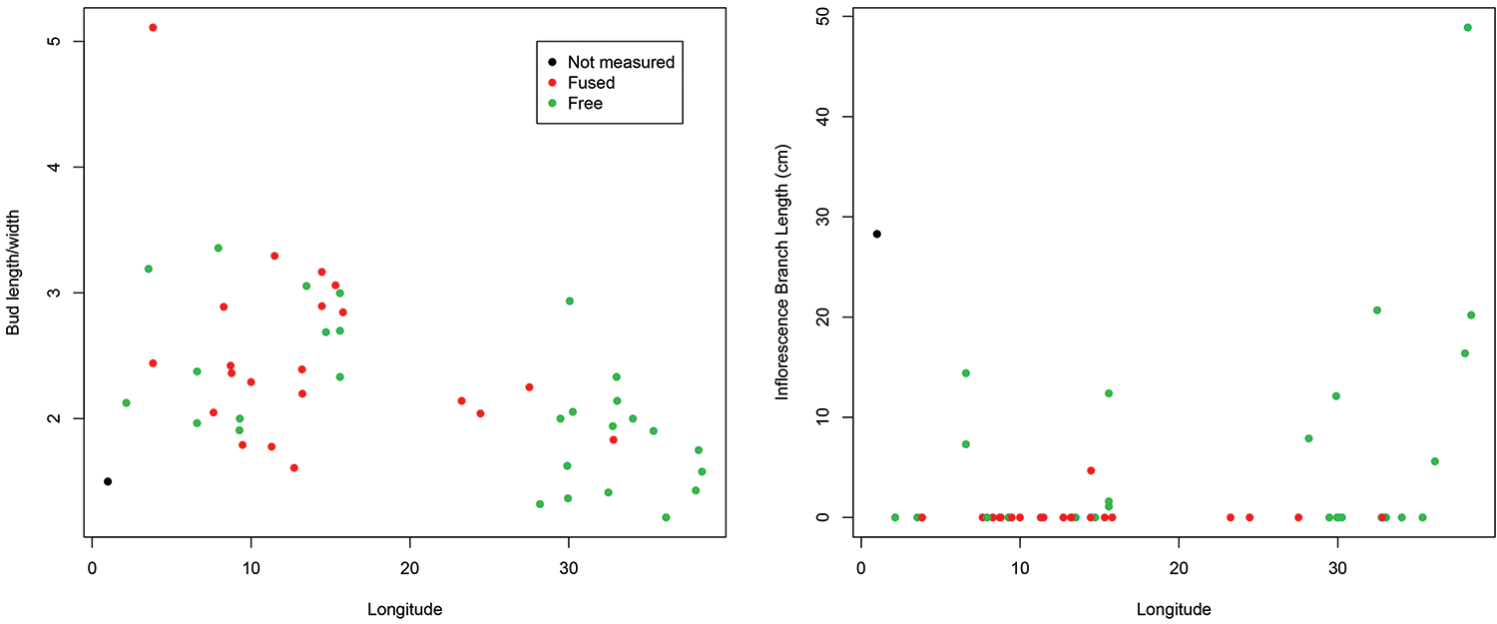
Morphology of *Solanum terminale* Forssk. across continental Africa. All coordinates are decimal degrees. **A** Bud length/width ratio plotted against longitude **B** Length of lowermost inflorescence branch against longitude. Red points represent plants with fused (connate anthers), green points plants with free anthers; black points represent plants with no open flowers whose bud and anther characters were not measured. For details of character choice see Materials and Methods and discussion of *Solanum terminale*.

In order to determine if there was any geographical component to the morphogical variation seen in *Solanum terminale*, we measured the key characters used to distinguish *Solanum terminale* from *Solanum welwitschii* in a range of specimens from across continental Africa. Long thin buds that open to flowers with fused anthers, and spicate inflorescences with short to non-existent branches are thought to be typical of *Solanum welwitschii* (e.g., Edmonds 2012). Neither character segregated clearly into two classes in our simple analysis (Figure 27). Bud shape is similarly distributed across longitude, but the longest, thinnest buds occur in western Africa (see point at top left-hand corner of Fig. 27A). Spicate inflorescences are somewhat more common in the wet forests of western Africa (Fig. 27B). The type of Solanum welwitschii var. laxepaniculatum (*Mildbraed 5265* - http://plants.jstor.org/stable/viewer/10.5555/al.ap.specimen.hbg511510), however, is a good example of a “terminale” type inflorescence in western Africa. Anthers on specimens from wet forests of western Africa (red dots in Fig. 27) were more often fused than those from drier forests of eastern Africa. Anther connation may be a way of protecting pollen in damp, humid environments, but this remains to be tested. The characters used to distinguish the taxa we here recognise as the single species *Solanum terminale* appear to be the extremes of continuous variation, and perhaps represent fixation of particular morphologies in certain populations. Field study of these plants coupled with molecular analysis at the population level will be essential to untangle the complex morphological variation with *Solanum terminale*.

In their catalogue of Forsskål’s plant collections Hepper and Friis (1994) cited both specimens in C as “type” of *Solanum terminale*; of these two sheets *Herb. Forsskål 419* (C10003105) has the larger inflorescence with more flowers and we select it here as the lectotype. They considered *Herb. Forsskål 406* (C10003106) a duplicate (Hepper and Friis 1994); we concur and consider it an isolectotype.

Both Udo Dammer and Georg Bitter described many infraspecific taxa in what we now recognise as the widely distributed and polymorphic *Solanum terminale*. Both botanists worked in Berlin before the Second World War and it is probable that much of the material upon which they based their descisions is no longer extanct (Vorontsova and Knapp 2010). Bitter (1917) acknowledged his lack of ability to travel to other herbaria due to military service and war as a short-coming of his treatment of this polymorphic complex. We have searched extensively for duplicates of this material throughout the course of the PBI Solanum project, and in some cases (e.g., see Solanum plousianthemum var. subtusbarbellatum above) have encountered duplicates, while in many, if not most, others we have not been successful. Rather than neotypifying these many infraspecific names we cite type collections in the hope that duplicates will eventually be encountered in the collections we have not yet been able to visit. Dammer (1906, 1912, 1915) never cited particular herbaria in association with specimens he used in describing new taxa (see Vorontsova and Knapp 2016), Bitter (1917, 1922) occasionally did so. Where he cited herbaria in association with type specimens we have indicated this, otherwise no herbarium was cited and we chose lectotypes from duplicates we have seen. Our choices of lectotypes are based on the quality of the particular sheet selected. When syntypes were cited, our choice has been made based on the number of herbaria in which particular syntype collections now are found; where more duplicates of a collection are extant, we selected that as the lectotype.

In his treatment of the species of the ANS clade in *Solana Africana* Bitter (1917) re-circumscribed many of Dammer’s (1906, 1915) species and excluded some of the specimens cited by Dammer as types of new infraspecific taxa. He explicitly cited types for Dammer’s names, something that later authors did not realise, resulting in some superfluous later lectotypifications (e.g., Edmonds 2012). *Solanum leucanthum* was first described by Dammer (1906) then explicitly redescribed as “*Solanum leucanthum* Bitter & Dammer n.sp.” by Bitter (1917). Dammer (1906) originally cited two syntypes from Cameroon, *Mildbraed 894* and *Mildbraed 1431a*, while Bitter restricted his concept to only *Mildbraed 894*. Bitter (1917) recognised *Mildbraed 1431a* as a distinct species *Solanum ruandae*. We have found no duplicates of either of these collections that were probably in Berlin and destroyed.

We have not selected a neotype for Dammer’s (1906) *Solanum suberosum*; of the seven syntype collections cited we are certain that duplicates will be found eventually. If not, the description and comments on the corky stem morphology will be easy to match, and a recent and widely distributed collection from Cameroon should be selected as a neotype.

Dammer’s (1906) illegitimate name *Solanum laurentii* (not *Solanum laurentii* De Wild., 1905) was described using a single specimen collected by “Laurent” from an unspecified locality in “Congo”. This sheet was in all likelihood at B and is no longer extant; the lack of specifics makes duplicates very difficult to trace. Durand and Durand (1909: 394) coined the replacement name *Solanum subcoriaceum*, but again did not specify a herbarium, sheet or locality; nor did they specify which Laurent, Émile or Marcel, was the collector of the type. We did not find a sheet annotated with this name in BR. We have selected a neotype in the Brussels herbarium collected by Marcel Laurent in 1905 (BR0000014801273) for *Solanum laurentii* Dammer (=*Solanum subcoriaceum* De Wild. & Durand) that matches the description in Dammer’s (1906) protologue.

Dammer (1906) described *Solanum plousianthemum* citing three collections made by C. Holst in Tanzania (*Holst 232*, *3731*, *8927*). Bitter (1917) later cited *Holst 232* and *Holst 3731* as elements in his redefinition of *Solanum plousianthemum*, and effectively lectotypified the name by stating “letztere Nr. der Typus zu Dammer’s originaldiagnose” – the latter number [*Holst 3731*] the type of Dammer’s original diagnosis). He cited *Holst 8927* as the type for his variety *conglutinans* without citing a herbarium. Edmonds (2012) superfluously lectotypified *Solanum plousianthemum* with the Kew duplicate of *Holst 8927*, none of the other collections cited in Dammer’s protologue have been found. We select the Paris duplicate (P00341605, with flower and fruit) of *Holst 8927* as the lectotype of Bitter’s var. *conglutinans*.

Similarly C.H. Wright (1894, 1906) rarely cited herbaria in new taxon descriptions, but because he worked at the herbarium of Kew, we have designated lectotypes at that institution unless a much higher quality specimen was available elsewhere (e.g., *Solanum welwitschii* where of the two syntypes *Welwitsch 6081, 6098* the BM sheet of the better documented syntype *Welwitsch 6098* has both flowers and fruits). In some cases (e.g., Wright 1897) the herbarium at Kew was mentioned in the introductory material in the publication, thus *Scott-Elliot 6800* at Kew (K000096900) is the holotype of *Solanum nakurense*. However, in the case of *Solanum lykipiense* where Wright (1894) states “ the specimen from which this description is drawn is about 6 in. long, but sufficient to show that the species is a very distinct one”, we have lectotyified the name with the K sheet which answers to this description rather than taking it as the holotype because there is no evidence that there were not several sheets with small fragments. Two collections, *Schweinfurth 3498* and *Mann 274* were cited in the protologue of Solanum welwitschii var. strictum (Wright 1894: 127); we select the Kew duplicate (K000414054) of *Mann 274* as the lectotype because another sheet is in Paris and we have only seen one duplicate of *Schweinfurth 3498*. For *Solanum phytolaccoides*, we have selected *Schimper 310* as the lectotype, rather than the other collection (*Johnston s.n.*) cited, so that can be used as the lectotype of Solanum plousianthemum var. connmixtum. In herbaria where we have examined duplicates of *Schimper 310* two different collection dates have been written on sheets, but the sheet in US (labelled as “ex Berol.”) has both dates; all duplicates of *Schimper 310* are here cited as isolectotypes, regardless of collection date.

De Wildeman and Durand (1899), Durand and Durand (1909) and De Wildeman (1922) worked at Brussels, but again did not cite herbaria in their species descriptions. For these names we have selected lectotypes at BR with the best quality duplicate available; for example, the lectotype for *Solanum lianiforme* is not the one without the original label, the sheet we selected (BR0000008993489) has label in De Wildeman’s hand with the species name and is a flowering specimen. The late Richard Lester affixed holotype labels to specimens at BR (e.g., *Thonner 96* the type collection of *Solanum symphyostemon*), and his “designations” were followed by Edmonds (2012). We have, in general, used these sheets as the lectotypes, unless duplicates in BR are of better quality.

Two collections were cited in the description of *Solanum balboanum* (Chiovenda 1935); *Balbo 425* and *Balbo 579*. We have selected *Balbo 579* (FT003031) because it has both flowers and fruits.

##### Selected specimens examined.

**ANGOLA. Cuanza Norte**: Munza, 8 Apr 1870, *Schweinfurth 3498* (K). **Cuanza Sul**: Kumbira Forest, 12 Jun 2014, *Goyder & Maiato 7749* (K). **Huíla**: Bumbo, Serra da Chela, Oct 1859, *Welwitsch 6032* (BM). **Luanda**: Cazengo, 1903, *Gossweiler s.n.* (BM). **Malanje**: Pungo Andongo, *Welwitsch 6107* [a] (BM, K, P); Mata de Cabondo, Feb 1857, *Welwitsch 6107* [b] (BM, G). **Zaire**: Ganji, 1910, *Gossweiler 5092* (BM).

**BURUNDI**. **Bujumbura**: Karonge, 17 Jun 1966, *Lewalle 981* (BR, EA). **Bururi**: Kwitaba, 13 Dec 1977, *Reekmans 6747* (BR, EA, K, P). **Cibitoke**: Mayabi, territoire Bubanza; Mabaye, riv. Lua, frontière du Rwanda, 2 Mar 1969, *Lewalle 3249* (BR); **Gitega**: pâturages naturelles de Kitego [=Gitega], 8 Dec 1922, *Elskens 239* (BR); territoire Bubanza; riv. Nyamagana, 21 Mar 1971, *Lewalle 5343* (BR); Nyabiraba, 4 Jun 1971, *Reekmans 698* (BR). **Karuzi**: Karuzi, 19 Dec 1957, *Lebrun 11269* (BR). **Makamba**: Kininya, Kininga [=Kininya], Mosso, 1 Jul 1952, *Michel 3159* (BR). **Muramwya**: Teza, 23 Jan 1980, *Bouharmont 14177* (BR); territoire Muramvya; Bugarama, 2 Dec 1967, *Lewalle 2446* (BR); Matara, 9 Sep 1973, *Reekmans 2939* (EA); Kiganda, 4 Jan 1978, *Reekmans 6848* (BR, EA, K). **Ngozi**: Gundava, Mar 1935, *Becquet 861* (BR). **Rutana**: Kiofi, 15 Mar 1952, *Michel & Reed 1572* (BR); Kininya, Mosso, 18 Jun 1952, *Michel 2915* (BR). **Ruyigi**: Nyabitare, 1 Jun 1952, *Michel 2604* (BR); Kigamba, 12 Oct 1978, *Reekmans 7184* (BR, EA, K).

**CAMEROON**. **Centre**: 2 km NE of Nguélémendouka, near Bamo River, 9 Apr 1962, *Breteler 2744* (K, P); Mefou Proposed National Park, Ndanan 1, 100m after bridge, 10 Mar 2004, *Cheek & Nana 11597* (BR, K); Ndokononoro, a 15 km au SW de Ndikiméki, 20 Dec 1971, *Letouzey 10894* (P); Nkol Emana, pres Mebo-Mezoa, 12 km SE Ngomedzad, soit a 45 km au SSW de Mbalmayo, 27 Jun 1972, *Letouzey 11385* (P); Colline Nkolandjom, près Ngoakélé, à 25 km à l’W de Ngoulémakong (route Mbalmayo-Tbolowa), 12 Jul 1972, *Letouzey 11471* (P); Mefou Proposed National Park, along the dry Mbeme river bed to Ndaligan 1, 25 Mar 2004, *Tadjouteu et al. 575* (BR, K). **Est**: Somalomo, 69 km SE Akonolinga, 17 Jun 1981, *Asonganyi 306* (P); 15 km N of Bertoua along road to Deng Deng, 15 Mar 1961, *Breteler 1205* (K, P); Gounté, ca. 27 km from Bertoua, along road to Betaré-Oya, 11 Dec 1961, *Breteler 2200* (BR, K, P); Djaposten, 38 km N of Lomié, right bank of Dja River, near bridge in road to Abong Mbang, 17 Sep 1965, *Leeuwenberg 6779* (BR, P); Yokadouma, 6 km along road to Batouri, 21 Jun 1961, *Breteler 1533* (BR, K, P); a 4-8 km de ‘Ene de Djolempoum, 26 Apr 1961, *Letouzey 3933* (P); Laye, 30 km W de Batouri, 5 Apr 1962, *Letouzey 4661* (P); Mopwo, 16 km a l’WNW de Mopwo, village située au km 22 de la route Yokadouma-Batouri, 4 Jun 1963, *Letouzey 5221* (P); entre Ngatto & Zoubalot, a 95 km au SW de Yokadouma, 31 Mar 1973, *Letouzey 12219* (BR, K x2, P); Boubara, 40 km NE de Batouri, 11 Apr 1962, *Letouzey 4712* (P); Deng Deng, Apr 1914, *Mildbraed 8838* (K); entre riv. Bango et Djombi, 27 km E de Batcha Ilo, village située a 25 km N de Moloundou, 9 Apr 1971, *Villiers 636* (NY, P); 5 km W Bateka Mahambe village situé à 26 km NE de Moloundou, 16 Apr 1971, *Villiers 654* (BR, K, P). **Littoral**: Mt Nlonako, flanc NW, 25 Dec 1967, *Bamps 1564* (BR, K, P). **Ouest**: Ndé, Bamena, about 15 km W of Banganté, 29 Apr 1964, *de Wilde & de Wilde-Duyfjes 2327* (BR). **Nord-Ouest**: Bambili, 3 Mar 1970, *Bauer 44* (K); Boyo, Tum, from Aboh, path towards Tum, 22 Nov 1996, *Etuge & Pollard 3576* (K); Bamenda, Fongom, May 1931, *Maitland 1690* (K, P); Mezam, Bali Ngemba F.R [Forest Reserve], 10 Apr 2002, *Onana 1982* (K); Mezam, Mantum, Bali Ngemba Forest Reserve, 12 Apr 2002, *Pollard et al. 953* (K); Bafut-Ngemba F.R. on the secondary bush by the side of Forest Nursery no. 1, near the boundary, 7 Apr 1951, *Ujor FHI*-*30026* (K). **Sud**: Lolodorf, route a Ebolowa, 17 Jun 1918, *Annet 401* (P); Colline Ongongondje, près Akonekye, 15 km NW de Amban, 23 Mar 1970, *Letouzey 10216* (P); Okpweng, 40 km route Djoum-Oveng, 17 Nov 1966, *Mesili 56* (BR); N’Koemvone, 14 km on the road from Ebolowa to Ambam, along the Seng river, 11 Jan 1975, *de Wilde 7893* (BR, P); Ebolowa, ca. 15 km S of Ebolowa, 1 Mar 1964, *de Wilde & de Wilde-Duyfjes 2023* (P); Bipinde [Bipindi], 1913, *Zenker, G. 4675* (BM, G, GOET, LE, P, S). **Sud-Ouest**: Mount Cameroon, Bimbia, 4 km SE Limbe (TB), 3 Aug 1993, *Baker 231* (K); Mount Kupe, Kupe Village, path between Kupe Rock and main trail to summit, 16 Jul 1996, *Cable et al. 3860* (K); Mount Cameroon, Bakingili, above Bakingili on hunters path leaving village behind school, through extensive old German bamboo plantation to ‘red hill’ a steeply ascending ridge of red soil, 21 Dec 1993, *Cheek et al. 5843* (K); Ngombombeng, near Ngombombeng village, north of Nyassosso, Apr 1986, *Etuge & Thomas 31* (BR, K, P); Mount Cameroon, Mount Etinde, Fako Division, Buea District, near Ekonjo, 1 Sep 1993, *Hunt 64* (K); Mount Cameroon, Bimbia, Mabeta-Moliwe, 2 Apr 1992, *Jaff 39* (K); Mount Cameroon, Likombe, 15 May 1992, *Mbani 119* (K); Fako, Buea, Upper Boando, between U. Boando and Etome, 25 May 1992, *Mbani 142* (K); Mount Cameroon, Boa, Operation Raleigh plots, 5 May 1994, *Ndam 1347* (K, SCA); Konye Esamanga Road, western Bakossi, 17 Feb 1988, *Nemba & Mambo 786* (US); Mount Cameroon, Etome, along an old timber exploitation road SW`of the Camp, 31 Jan 1997, *Nning et al. 200* (K, SCA, YA); Mount Cameroon, Mabeta, Mabeta-Moliwe Reserve area 1, 12 Jan 1992, *Sunderland 1055* (K, SCA, YA); W Bamboutos Mts, Fosimondi, Fossimondi village, alongthe road leading to government school, 28 Apr 2005, *Tchiengue et al. 2232* (K); Mount Cameroon, Mount Etinde, Batoke Path, 28 Oct 1992, *Tchouto 478* (K,SCA,YA); Barombi Camp, 5 km S of Kumba on Buea/Douala Road, 21 Jun 1983, *Thomas 2176* (NY); Fako, Bakingili, trail up Mt Cameroon, 9 Jul 1984, *Thompson et al. 1555* (BH); Nyasoso, Mount Kupe, 4 Jun 1996, *Zapfack et al. 682* (K).

**CENTRAL AFRICAN REPUBLIC**. **Haute-Kotto**: 60 km W de Yalinga, *Le Testu 3910* (BM, BR, P); 39 km S de Yalinga route de Bangassay, 30 Jun 1922, *Le Testu 3971* (BM, BR, P); **Lobaye**: Boukoko, Riviere Dungwa, 20 Mar 1923, *Tisserant 1251-1040* (P); Mbaiki, Boukoko, Nzua-Motoli (Lissango), 4 Aug 1950, *Tisserant 1861* (BM, G, P x2); Mbaiki, Boukoko, Station Centrale de Bounoko, Nzua-Motoli (Lissango), 16 Apr 1951, *Tisserant 2078* (BM, G, P x2). **Mambéré-Kadéï**: Berbérati, forêt de la mission Catholique, 1 May 1937, *Tisserant 3490* (BM, P). **Nana-Mambéré**: region de Bouar, Sep 1960, *Koechlin 6257* (P). **Ouaka**: Riv. Ngumanga, Bambari, region L’Oubangui, Jun 1921, *Tisserant 286* (P); Moronbas, 20 Mar 1923, *Tisserant 1040* (BM, P).

**CÔTE D’IVOIRE**. **Lagunes**: Yapo-Nord, ad orientem-oppidi, 15 Mar 1962, *Bernardi 8681* (G, P, US); Orumboboka, 40 km S of Toumodi, 13 Jun 1968, *Bokdam 2789* (BR); forêt du Yapo, 11 May 1967, *Cremers 643* (BR, P); km 9 of Yakassé Mé-Kodiosou road, 31 Jul 1970, *Leeuwenberg 8068* (BR, K, P). **Bas-Sassandra**: Soubré, 70 km S of Soubré, 3 Apr 1968, *Geerling & Bokdam 2489* (BR); 61 km N. of Sassandra, W of Niapidou, 520 Jan 1959, *Leeuwenberg 2518* (K). **Comoé**: Mbasso, Indénié, Region de Moyen-Comoe, 25 Dec 1909, *Chevalier 22652* (P). **Montagnes**: Mont Tonkoui, 6 Mar 1968, *Aké Assi 9938* (G); Mont Nimba, sur la crête, entre le point E et le point 1380, 10 Feb 1989, *Gautier-Beguin & Gautier 1133* (G); Mont Nimba, près de la base IFAN du Mt. Nimba (Guinee francaise), 23 Dec 1946, *Roberty 6680* (G); Mont Tonkoui, Jan 1950, *Schnell 4079* (P); Monts Nimba [border with Guinea], Apr 1950, *Schnell 5341* (P); ); piste Guessabo, Mont Do, 31 Jan 1969, *Bamps 1995* (BR).

**DEMOCRATIC REPUBLIC OF THE CONGO**. **Bandundu**: Kaniama, Jan 1939, *Brédo 2721* (BR); Kibulu, [=Kibubu], 7 Feb 1950, *Callens 2432* (BR); Bolobo, *Demeuse 451* (BR); Lupatapata [Luhatahata], NE Dibaya, Bakura Sumpi, crête partage Lubi-Lubilash (plateaux), 16 Feb 1937, *Gillardin 202* (BR, K); Bokoro, 12 Nov 1947, *Jans 621* (BR); Loozi, [Lozi], 19 Feb 1904, *Luja 151* (BR); Kikwit, 28 Oct 1993, *Masens 1341* (BR); Bas-Katanga, Kaniama-Haut Lomami, 24 Feb 1947, *Mullenders 1520* (BR); Popokabaka, 29 Nov 1958, *Pauwels 778* (BR); Lukombe, Oct 1910, *Sapin s.n.* (BR); Kikwit, Jan 1914, *Vanderyst 2881 bis* (BR); region Babunda, Mar 1921, *Vanderyst 8980* (BR); Ipami [Ipamu] a Kikval, Jul 1921, *Vanderyst 9814* (BR); Lemfu, Kistanu-Kwango [=Inkisi], *Vanderyst 14229* (BR); Kwango, Goa, 1926, *Vanderyst 19168 bis* (BR). **Bas-Congo**: Thysville, [Mbanza-Ngungu], 3 Jun 1915, *Bequaert 7747* (BR); Luki [Luki Biosphere reserve, Mayombe Forest], *Brixhe 19* (BR); Kingedi, Terr. Lukula, 19 Aug 1959, *Compère 128* (BR); Mission de Lemfu, 30 Mar 1944, *Germain 2007* (BR); Kinanga, 1874, *Gillet s.n.* (BR); Twataba, 1909, *Gillet s.n.* (BR); Kisantu, [=Inkisi], *Gillet 717* (BR); Sanda, Apr 1903, *Gillet 3016* (BR); Gimbi, 2 May 1958, *Laurent 562* (BR); Sona Gangu, cataractes [Sona Gungu], 21 Feb 1898, *Luja 112* (BR); Ndembo, 14 Apr 1911, *Vanderyst s.n.* (BR); Kinkosi, 26 Feb 1907, *Vanderyst s.n.* (BR); Lazaret du Sacré Coeur [Kisantu, =Inkisi], Mar 1911, *Vanderyst s.n.* (BR); Monte Ngala, Kisantu [=Inkisi] -Kwango, Jun 1925, *Vanderyst 14444* (BR); Temvo, 17 Feb 1919, *Vermoesen 964* (BR); Boma, INEAC-LUKI, 16 Feb 1959, *Wagemans 2172* (BR); Takalu, 29 Aug 1921, *Wellens 286* (BR). **Équateur**: Bongabo, terr. Gemena, 8 Jun 1971, *Breyne 1703* (BR); Lokolenge, *Bruneel 46* (BR); Eala-Bamania, 18 Aug 1912, *Chevalier 28197* (P); Mbandaka, Eala, 19 Apr 1931, *Corbisier-Baland 1034* (BR, K, P); Station Inéac Boketa, 14 Sep 1955, *Evrard 1788* (BR); Monkoto, route Wafania-Biyanga, 13 Nov 1957, *Evrard 2975* (BR, K); Likimi, Mobeka, Likimi, Jan 1913, *de Giorgi 69* (BR); Yambata, *de Giorgi 1344* (BR); Yambata, *de Giorgi 1824* (BR); Bikoro, environs, Lac Tumba, Dec 1920, *Goossens 1522* (BR); Pasha, environs, Lac Tumba, Dec 1920, *Goossens 2384* (BR); Monsole, Jul 1923, *Goossens 4209* (BR); Sud Ubangi, Kalo, Dist. de Mbangi, May 1924, *Goossens 4318* (BR); Bokuma, 028 Feb 1941, *Hulstraert 567* (BR); Wafanya, 20 Dec 1941, *Hulstraert 592* (BR); Porte de Moma, à un kilometre au porte sur la route vers la rive de la Tshuapa, Nov 1910, *Jespersen s.n.* (BR); Tshuapa, Belo, (Ikela) porte de Belo, 1Oct 1910, *Jespersen s.n.* (BR, K); Bakasu, 17 Sep 1905, *Laurent s.n.* (BR); Wendji, environs de Coquilhatville [Mbandaka], May 1930, *Lebrun 307* (BR); Bolombo, Eala, May 1930, *Lebrun 438* (BR); entre Bokuma et Bokatola, Jul 1930, *Lebrun 1354* (BR, EA, P); Businga (Ubangi), Jan 1931, *Lebrun 1972* (BR); Buta (Uele-Gamberi), Mar 1931, *Lebrun 2483* (BR); Bili (Uele-Gambiri), May 1931, *Lebrun 2828* (BR); Mobwasa, 15 May 1913, *Lemaire 202* (BR); Mbandaka, Eala, route de Coq, 7 Oct 1946, *Leonard, A. 802* (BR, EA, P); Mongo, 25 Sep 1935, *Louis 146* (BR, EA, FT); Likimi, environs a Likimi, 9 Sep 1910, *Malchair 300* (BR); Mbandaka, Coquilhatville [Mbandaka], 019 Jul 1906, *Pynaert 274* (BR); Dundusana, *Reygaert 226* (BR); Parc National Monkoto, Parc National Monkoto, au sud de Booke (terr. Kole) [=PN de la Salonga], May 1958, *Robin 67* (BR); Eala, route de Bolombo, Territ. Coquilhatv[ille], Sep 1925, *Robyns 684* (K); Equateur, Mabali, Lac Tumba, I.R.S.A.C., Terr. Bikoro, 14 Feb 1957, *Thonet 154* (BR); Bokondji, 7 Sep 1959, *de Wankel 148* (BR). **Kasaï-Occidental**: Kasai, Luiza, Nov 1921, *Achien 639* (BR); Hempt St Ben, 1 Jan 1917, *Callewaert s.n.* (BR); Kakenge, terre du Bakuba, Dec 1937, *Gillardin 317* (BR, K); Concession Elkasai a Tumba (Terr. Luisa), 26 Feb 1957, *Liben 2571* (BR); Kasongo, Bateteur [Kasongo Batetela], 1906, *Sapin s.n.* (BR); Tshibangu, Feb 1910, *Sapin s.n.* (BR); Tshibangu, Jan 1910, *Sapin s.n.* (BR). **Kasaï-Oriental**: Sankuru, Kole, Oct 1904, *Claessens 299* (BR); Katako-Kombe, (Kindu), Jun 1860, *Claessens 453* (BR); Sankuru, Bena Dibele, Oct 1904, *Claessens 949* (BR); Mukumari, Lomela, May 1939, *Gillardin 586* (BR); Sankuru, entre Lusambo et la Lomami, Dec 1898, *Laurent s.n.* (BR); Bombaye [Bombaie], 27 Nov 1903, *Laurent & Laurent s.n.* (BR); Gandajika, [Gandajika], 10 Dec 1956, *Liben 2065* (BR); Mwenetontole [Mwene Tonto], 1 Feb 1936, *Luxen 384* (BR). **Katanga**: a Kaziba, col. Kachika (district Mitwaba), 2 Sep 1958, *Laurent 542* (BR); Rivière Mwera, 10 km S de Kiembe (Haut Shaba), 5 Feb 1982, *Malaisse & Robbrecht 2378* (BR); Kapanga, Nov 1933, *Overlaet 1086* (BR); Kankima River, galerie de Kankima, Jul 1939, *Quarré 39* (BR); Gorge de Kalaba, Jul 1939, *Quarré 698* (BR); Muhila, [Bugana-Muhila] Ferme Bertrand, Oct 1945, *Quarré 7051* (BR); Elisabethville, [Lubumbashi], Jun 1920, *Rogers 26252* (G); Lualaba, Dilolo, 1935, *Vin 45* (BR). **Kinshasa**: Tshangu, Maluku, route Mabana km 3, 27 Nov 1970, *Breyne 981* (BR); Leopoldville, Territ. Lukula, route Kimbuya-Kingedi, 24 Aug 1959, *Compère 160* (BR, K); Leopoldville, [Kinshasa], 30 Jan 1907, *Vanderyst s.n.* (BR). **Maniema**: Tschopo, Namoya, Mwendamboko Hill, 12 Apr 2008, *Bytebier & Luke 2784* (BR); Kindu, chemin forestier de Bweni, 15 Apr 1959, *Gaillez 281* (BR); Usega, Waniema [Maniema], Jul 1932, *Lebrun 5705* (BR); Kamusuku, territoire de Shabunda, 18 Aug 1959, *Leonard 5974* (BR, K); Muhulu, de Kyampsumba, a 42 km au NNW de Kolweli, 6 Dec 1983, *Schaijes 2088* (BR); Pope, Kasonga Lunda, 1925, *Vanderyst 17358* (BR). **Nord-Kivu**: route Goma-Costermanville, environs de Goma, 24 Jun 1953, *van der Ben 548* (BR, K); Beni, 2 Apr 1914, *Bequaert3344* (BR, K); Irangi, 70 km W of Lake Kivu, 26 Aug 1959, *Cambridge Congo Expedition 1959 428* (BR, US); Kahele, Irangi, pres Camp ICRSAC a Irangi, km 110 route Kavamu-Walikale, 13 Aug 1956, *Christiaensen 1835* (BR, CAS); Bwito, Station Kikondo, Colline Shinga, 23 Mar 1954, *Deru 102* (BR); Colline Kashaka, route Goma-Sake, 11 Jan 1945, *Germain 3323* (BR); Kokola, km 100 Irumu-Beni, Aug 1939, *Gille 267* (BR); Kililema, Terr. Masisi [=Kilima], 29 Aug 1957, *Gutzwiller 1541* (BR); Lugandamo, Biega [Mont Biega], May 1947, *Hendrichx 4602* (BR); plaines de lavas entre les lacs Kivu et Edouard, Apr 1929, *Humbert 7915* (BR,P); chaine des Virunga (Kivu), Volcan Karisambi, *Lebrun 4933* (BR, G, K); Kasindi-Lubango (Kibali-Ituri), chaine W de Lac Edouard, 1932, *Lebrun 4788* (BR, K); Nyiragongo, entre Kibali et Nyiragongo, Nov 1937, *Lebrun 8692* (BR); Lac Muguga [Mugunga], Nov 1937, *Lebrun 8856* (BR); Rutshuru, Kivu, Dec 1937, *Lebrun 8979* (BR, K); Kirambo, Terr. Masisi [=Kilumbo], 15 Jan 1959, *Leonard 2562* (BR); Ruzizi, Kahuzi, [Kahuzi], 28 May 1960, *Meurillon 931* (BR); Musisi, route Bukavu-Bitale, 13 Mar 1959, *Pierlot 2771* (BR, K); km 193 route Butavu-Goma, abords de cette ville, 3 Jun 1959, *Pierlot 2947* (BR); Kisharo, km 30 route Rutshuru-Katwe (près de la frontière de l’Uganda), territoire de Rutshuru, 8 Jun 1959, *Pierlot 3090* (BR); Irangi, Territ. Kalehe, route Kavumu-Walikale, vers km 110, Irangi, Reserve IRSAC, 10 Jul 1957, *Troupin 3795* (BR, K); Mutsora, Parque Nacional Albert, Talya, 17 Apr 1953, *de Witte 8843* (BR, K); Mont Muhete, près Mutsora, 24 Feb 1954, *de Witte 9866* (BR); Lusilube, affl. droit Semliki (P.N.A.=Virungas National Park), 13 Sep 1954, *de Witte 11084* (BR); N’Jomaluini, marais près N’Zinga, P.N.A. [Virungas National Park), 12 Mar 1955, *de Witte 11977* (BR); Tsliaberimu Mt. [Tschiaberimu], 15 Apr 1955, *de Witte 12153* (BR); Saya-Sago, rig. Mt. Huyo [Mount Hoyo], 114 Jul 1955, *de Witte 12531* (BR); Kideduya, près riv. Lusilube, 13 Sep 1955, *de Witte 12689* (BR); Riv. Talya affl. Lumi, près Mutsora, 29 Mar 1956, *de Witte 13040* (BR); Riv. Kenyamoshii, affl. droit Murve (Kashubu), 7 Aug 1956, *de Witte 13224* (BR). **Orientale**: Ituri, Foret de Djugu, Riv. Ruida, 17 Apr 1958, *Bamps 160* (BR); Yangambi, île Esali, 4 Mar 1959, *Bamps 392* (BR); Barumbu, 30 Oct 1913, *Bequaert 1036* (BR); Yambuya, 21 Nov 1913, *Bequaert 1242* (BR); Avakubi, 13 Jan 1914, *Bequaert 1915* (BR); Ituri, Irumu, 9 Mar 1914, *Bequaert 2859* (BR); Yafake Commuinity, Lilanda, 7 May 2010, *Boyekoli Ebale Congo Expedition 2010 271* (BR); Bomane, Aruwimi River, 21 May 2010, *Boyekoli Ebale Congo Expedition 2010 658* (BR); Bambesa, et environs, 1933, *Brédo s.n.* (BR); Haut-Uele, Dingila, 4 Jul 1933, *Brédo 135* (BR); Isangi, Yangambi (réserve forestière), 12 Feb 1979, *Breyne 3604* (BR); Barumbu, Dec 1920, *Claessens 27* (BR); Bafwaboli, *Claessens 308* (BR); Nebasa, Apr 1921, *Claessens 531* (BR); Dinda, May 1921, *Claessens 634* (BR); Ituri, Lekwa, Djugu, 6 May 1959, *Deville 341* (BR, CAS); Basankusu, terr. de Mbale, District Tshuapa, Jun 1934, *Dubois 432* (BR); Stanleyville [=Kisangani], 16 Dec 1898, *Duchesne 2* (BR); Rucu (Mahagi), 27 May 1959, *Froment 451* (BR); Ituri, Berunda, (Mahagi), 12 Jun 1959, *Froment 539* (BR); km 24 route Kole-Kanwa, Terr. de Banalia, 13 Jun 1932, *Galdermans 11* (BR); Tukpwo, 1 Apr 1954, *Gerard 1286* (BR); Bambesa, 12 Nov 1956, *Gerard 2519* (BR); Nadabu, (Zobia), 15 Apr 1957, *Gerard 2812* (BR); Kurukwata, (Aba), 10 Aug 1957, *Gerard 3562* (BR); Kurukwata, (Aba), 2 Sep 1957, *Gerard 3681* (BR); Rubi, (Buta), 28 May 1958, *Gerard 3918* (BR); Ituri, zone de Mambasa, along trail to Aketu Hill from abandoned coffee plantation in Nzaro (about 90 km N of Mambasa on road to Isiro, 8 Mar 1994, *Gereau et al. 5457* (EA, MO). Bambesa, 3 Jul 1942, *Gilbert 465* (BR); Haut-Uele, Riv. Guruba (Doruma), May 1936, *de Graer 550* (BR); Mulimba, Mutongo, territoire Walikale, 1 May 1958, *Gutzwiller 2641* (BR); Kaseke, Mutongo, territoire Walikale, 2 May 1958, *Gutzwiller 2703* (BR); Ibucha, Mutongo, Terr. Walikale, 12 May 1958, *Gutzwiller 2883* (BR); Mumu, Mutongo, Terr. Walikale19 Jun 1958, *Gutzwiller 3174* (BR); Epulu, Zone de Mambasa, Ituri, 114 Jul 1988, *Hart 863* (BR); Bamanya [Bamanga], Bosako, 31 Mar 1945, *Hulstraert 1392* (BR); Ituri, Djugu (Tibali - Ituri), Jul 1931, *Lebrun 3941* (BR, P); 500 m au SE de Kabondo, Kisangani, 30 Apr 1977, *Lejoly 1444* (BR); Ile Kongolo, a la confluence avec le fleuve Zaire [Congo], 10 Feb 1979, *Lejoly 4774* (BR); Kitshanga, Terr. Walikale, 17 Feb 1959, *Leonard 2826* (BR); Haut-Zaïre, route Kisangani-Opala, 2 Jun 1973, *Lisowski 18587* (BR); Tschopo, Yangambi, réserve-flore-Isalowe, 13 Jan 1938, *Louis 7498* (BR, NY); Lilanda, route d’Isangi, près de la basse Lilanda, 9 May 1938, *Louis 9280* (BR, FT); Ile Idjwi, Lake Kivu, 1 Mar 1939, *Loveridge 639* (K); Mobwasa, *Reygaert 1360* (BR); Haut-Uele, Garamba National Park, piste Wilibadé, source Nambia-Boisquet, 18 Jul 1948, *Robyns 3125* (BR); bords Kidi - Gombari, 26 Jan 1906, *Seret 576* (BR); Ituri, Reserve de Djugu, 29 Oct 1951, *Smeyers 72* (BR); Tshopo, Panga, Popoy, km 139 de la route Banalia-Panga, près dernier pont avant Panga, 20 May 1987, *Szafranski 1277* (BR); Mbolohu, Chefferie Wanundi, piste Eringete, 17 Apr 1944, *de Wilde 367* (BR, CAS); Mont Mangalaba, près Talia Nord, rég. Tshiaberimu, 17 Mar 1954, *de Witte 9919* (BR); Bikito, près Malibotu affl. Ilimbo, 2 Jul 1954, *de Witte 10827* (BR). **Sud-Kivu**: Lukando, 15 Feb 1936, *Babault 845* (P); Idjwi, Lac Kivu, Réserve C N Ki, Kalambo, 229 Sep 1953, *van der Ben 909* (BR); Tahibati, Kabare Territ., 14 Aug 1953, *Christiaensen 5* (BR, NY); Kabare, Lwiro, Centre ICRSAC, 4 Jul 1958, *Christiaensen 2444* (BR, CAS); Lubarika, Plaine de la Ruzizi, 30 Jan 1950, *Germain 5920* (BR,K); Kalongé, Ruwenzori, Aug 1932, *Hauman 199* (BR); Tshizimwho [Mont Kahuzi], 1 Oct 1940, *Hendrickx 1288* (BR, K); Ndjondohe, Tshirumbi, Jan 1946, *Hendrickx 3844* (BR); Kavumu, Luhasagula, May 1946, *Hendrickx 4060* (BR); entre Kahuzi et Biega, Nov 1946, *Hendrickx 4246* (BR); Ile Idjwi, Lac Kivu, May 1929, *Humbert 8333* (P); Parc National Virunga, massif de Ruwenzori, entre Mutwanga et la gite Kalonge, 28 Dec 1977, *Lejoly 2339* (BR); Mikonzi, vallée de la Tshinginda, km 42 route Kavamu-Walikale, territoire de Kahele, 12 Feb 1957, *Pierlot 1461* (BR); forêt de la Musisi, (Kahusi), territoire de Kabare, 7 May 1958, *Pierlot 2006* (BR); Kahuzi, an der Strasse Bukavu-Walikale (Kahuzi Sattel), 14 Jan 1955, *Stauffer 1043* (BR, K, P); Centre Iwiro, IRSAC, riv. Iwiro [Lwiro], Kabare Territ., 21 Jun 1958, *Troupin 7517* (BR, NY); le long de la Butahu, pres de Kalongé, Parc National Albert, 19 Aug 1952, *de Witte 8006* (BR, CAS); Kalongé, versant droit de la vallée de la Butahu, en fase de Kalongé, 13 May 1953, *de Witte 9015* (BR).

**EQUATORIAL GUINEA**. **Bioko Norte**: Fernando Po, El Pico, 10 Dec 1951, *Boughey 190* (K); carretera del pico Basilé, km 6-7, 12 Dec 1989, *Carvalho 4232* (K, MA). **Bioko Sur**: Gran Caldera de Luba, descenso en al dirrecion a Moraca 28 Feb 1990, *Carvalho 4274* (K, MA); Musola, Nov 1942, *Guinea 1094* (MA); Fernando Po, Missola [Musola], trocha de servicio Agronómico, 10 Jan 1947, *Guinea 1144* (K); Finca Puente, km 17, carretera de Musola a Moka, 5 Mar 1950, *Guinea 1666* (MA); Homiga Camp, 23 Jan 2009, *Luke 13206* (EA, K, MA). **Centro Sur**: Parque Nacional de Monte Alén, Ocuam, 26 Nov 1998, *Pérez Viso 623* (BM, K, MA). **Litoral**: Bata-Niefang, estrada Km 35, en zonas de Adjape e de Comayá, entre os Rios Sama e Comayá, 18 Feb 1995, *Carvalho 5814* (BM, K, MA).

**ERITREA**. **Maekel**: Hamasen, Asmara, 15 Oct 1909, *Baldrati 407* (FT); Hamasen, Asmara, 15 Oct 1909, *Baldrati 3532* (FT).

**ETHIOPIA**. **Addis Ababa**: Addis Ababa, 23 Jan 1963, *Tekle 220* (K). **Amhara**[?]: Lötho, 11 Nov 1857, *Schimper 480* (E, FT, G x2, P). **Amhara**: Agew Awi, Injiabare [Injiabara] to Chilga, 31 Oct 1995, *Friis et al. 7011* (K); Dessie, Wollo province, 23 Aug 1946, *Hall 98* (BM); 34 km N of Bahar Dar, along the road to Gondar, counted form the bridge crossing the Blue Nile River, 17 Sep 1970, *de Wilde 7173* (BR). **Gambella**: [Gojam floristic region] 30 km W of Tepi, 21 Jul 1993, *Renya 77* (ETH); Illubabor region, Bishan Waqa Lake [Bishan Wak’a Hayk’], 24 Sep 1996, *Tesfaye 408* (ETH). **Oromia**: Bale, Robie [Bale Robe], 11 May 1975, *Ash 2932* (BR, K, US); West Shewa, Ambo [Hagre Hiwof], Sep 1938, *Benedetto 26* (FT); Illubabor, Gore, towards Tepi, 4 Nov 1986, *Gilbert & Friis 8417* (ETH, K); Harerge, 22 Feb 1932, *Gillett 5149* (K); Belleta Forest, 718 Apr 1997, *Pavlov 97113* (ETH); East Welega, 5 km E of Nekemt[e], 16 Apr 1965, *de Wilde & de Wilde-Duyfjes 6315* (ETH). **Southern Nations, Nationalities and Peoples Region**: Keffa, 20 km SW of Mezan Tefari (Aman), 29 Nov 1984, *Friis et al. 3842* (ETH, K); Kibre Mengist to Wondo, 12 Apr 1975, *Gilbert & Jones 187* (K); Gidole, [Dirashe], 22 Jan 1985, *Haugen 440* (ETH); Leliso River, 18 Nov 1979, *Mesfin Tadese 759* (ETH); Jimma, 5 Dec 1955, *Stewart B*-*19* (BM, EA, K); Keffa prov., about 8 km along the new road for Bonga to Mizan Tefari (counted from turnoff to Bonga), 2 Aug 1969, *de Wilde 5310* (BR); [Nolaita] Soddo, 5 Mar 1938, *Vatova 1896* (FT). **Tigray**: Temben [Tembien], *Solazzo 284* (FT).

**GABON**. **Estuaire**: Mont de Cristal, km 20 road Kongouleu-Assak, 8 Oct 1986, *Breteler & Lemmens 8395* (BR, P); Monts de Cristal, Mont Mela, 9 Feb 1968, *Hallé & Villiers 5118* (P). **Haut-Ogooué**: km 20 Moanda-Mbinda, 18 Sep 1970, *Breteler 6502* (BR); SSW of Moanda, 113 Oct 1970, *Breteler 6869* (BR, P); between Mouila and Yeno, about 40 km. on road from Mouila, 23 Sep 1986, *Breteler et al. 8127* (BR); region de Lastourville, Miçoungangui, 6 May 1930, *Le Testu 8069* (BM, BR, MA, P); ca. 17 km SE of Franceville, along the road to Ndoumou, 4 km before the village of Ndjoky, 6 Dec 1989, *de Wilde et al. 9942* (BR). **Ngouie**: Mimongo, route de Libamba a Etéké, 14 May 1963, *Hallé & Cours 5868* (K, P). **Ogooué-Lolo**: Chantier Bambidie (CEB), ca. 55 km E of Lastourville, 40 km on CEB road to Okondja, 1 Nov 2005, *Sosef et al. 2176* (BR). **Ogooué-Ivindo**: Station d’Ipassa, 10 km S de Makokou, 11 Mar 1977, *Florence 37* (P); route de Belinga km 60, 21 Mar 1978, *Florence 669* (P); Nké, campement, 4 Oct 1983, *Floret & Louis 1600* (MA, P); M’Passa Field Station, near Makokou on Rivière l’Ivondo, 28 Jun 1981, *Gentry 33014* (MO, P); Mekambo, 20–50 km W of Mekambo, 14 Oct 1964, *Hallé 2612* (P); Belinga, 11 Nov 1964, *Hallé 3125* (P); Makokou, 10 km SSW of Makokou, near Ivindo R., right bank, 1 Nov 1977, *Leeuwenberg 11403* (BR, P). **Wolem-Ntem**: 7 km E of Mitzic, 9 Jul 1957, *Jeffrey 36* (K); Medouneu, Mitzic, 2 Aug 1957, *Jeffrey 199* (K, P).

**GHANA**. **Ashanti**: Akin, Begoro district, Jan 1930, *Irvine 1172* (K); Akin Ranch, Begoro district, Mar 1932, *Irvine 1812* (E, K); Asin Cocoa Station, Juaso, Jul 1957, *West-Skinn 19* (K). **Eastern**: Atewa Range Forest Reserve, beside path in east central part near Kibi, 5 Nov 1982, *Hepper 7406* (K); Aburi, 23 Nov 1898, *Johnson 158* (K); Atewa Range, starting in Awhiniasi on the Kibi-Apapam road, walking W to meet the road between Apapam and the other side of the range, 30 Jun 1994, *Jongkind & Abbiw 1638* (BR); Accra-Kumasi Highway, 4 km along the forest service road that intersects the highway at the village of Sagyimase, Atewa Range Forest Reserve, 1 Jul 1995, *Merello et al. 1147* (K, MO); Kade, 4 Feb 1953, *Morton GC*-*8387* (K); Bosuso, road between Bosuso and Begaro, 9 May 1947, *Robertson K*-*9* (K). **Volta**: Togo Plateau Forest Reserve, 19 Jun 1972, *Abbiw GC*-*43712* (BH, EA). **Western**: Ateiku, May 1930, *Anonymous 1952* (K); Nzema East, Axim, Jul 1905, *Chevalier 13825* (P).

**GUINEA**. **Nzérékoré**: Yah River, 14 Sep 1964, *Adames 536* (K); Loffa, Macenta, 14 Feb 1949, *Adam 3734* (P); Zoubouromai, 20 Mar 1949, *Adam 4008* (P); Sérédan, May 1936, *Jacques-Felix 913* (P); Boola, Mount Vtongoy, pays des Guerzés, Montagne de Boola, 16 Mar 1909, *Chevalier 20935* (P); entre Lola et Nzo, Pays des Guerzés, 24 Mar 1909, *Chevalier 20992* (P); Mt. Yonon, forêt classée, 19 May 1971, *Jongkind 10971* (BR); Simandou range, east slope of Pic de Fon, near the water intake of Canga East mining camp, 20 Mar 2008, *Burgt et al. 1139* (K); Macenta, Kouankan, 2 km près du village, 12 Jun 1970, *Camara 691* (G); forest around Whiskey 2, 25 Mar 2008, *Tchiengue & Haba 3136* (K).

**GUINEA BISSAU**. **Tombali**: Bedanda-Cadique, floresta de Cantanez, 5 Jan 1962, *Alves Pereira 2640* (K, MA); Cantanhez-Bedanda, 1 Sep 1962, *Alves Pereira 3148* (MA); Camocote, 7 Nov 1995, *Malaisse & Claes 16813* (BR).

**KENYA**. **Central**: Nyeri, 20 Jul 1929, *Balbo 425* (FT); Nyandarua, Kinangop, *Belcher H*-*138*-*53* (EA); Kirarkop, Aberdare Mountains, Apr 1938, *Chandler 2216* (BRK); Thika, Blue Post Hotel, above Thika falls, behind the Hotel., 5 Jan 1967, *Faden 67/1* (EA); Aberdare National Park, 2 Feb 1932, *Fries 1565* (EA); Kiambu, Muguga, 1 Aug 1960, *Greenway 9700* (EA, FT, K); Kikuyu Escarpment Forest, Lari Forest Reserve and adjacent areas, Kiambu District, 26 Nov 1972, *Hansen 790* (BR, EA, FT, K); Kiambu, Banana Hills, 14 Sep 1969, *Hindorf 805* (BR); Muranga, Kimakia Forest Station, Essex, Aberdares, 29 Jul 1958, *Kerfoot 667* (EA); Kieni Forest, 27 Oct 1974, *Mungai 77* (EA); Kiambu, Kikuyu, Mar 1944, *Nattrass 345* (EA); Kikuyu Escarpment Forest, along Naivasha road, opposite Ngubi Forest Reserve Guard Post, 15 Dec 1966, *Perdue & Kibuwa 8252* (EA, K); Kiambu, Limuru, 10 Jul 1918, *Snowden 584* (BM, K); Muguga, 13 miles on Nairobi-Naivasha road, 6 Jun 1952, *Verdcourt 653* (EA, K); Nyeri, Mt. Kenya, 800 m outside the Naro Moru Gate to Mt. Kenya National Park, 14 May 2009, *Vorontsova et al. 56* (BM, EA). **Coast**: Taita Taveta, Ngangao Forest, Taita Hills, 15 May 1985, *Faden et al. 483* (EA, K, US); Taita Taveta, Chawia Forest, Taita Hills., 22 May 1985, *Faden et al. 857* (EA); Taita Taveta, Mbololo Forest, Oct 1938, *Joana 8996* (EA); Taita Taveta, Taita Hills, Mgambonyi, Uminga, Mgambonyi market, 25 Jan 2006, *Kirika et al. 01/2009 29* (EA, K); Ngangao Forest, northern end, 26 Aug 1999, *Mbuthia & Mwangangi 185* (NY); Taita Taveta, Bura, Taita Hills, top of ridge about 5 km S of Bura Mission west side of Bura Valley, 8 Apr 1998, *Mwachala et al EW-1251* (EA, K, NY); Taita Taveta, Chawia Forest, Taita Hills, 18 May 1931, *Napier 1132* (EA, K); Wundanyi, Mar 1939, *Opiko 8758* (EA). **Eastern**: Makueni, Chyulu, 14 Jun 1938, *Bally 7786* (K); Meru, Mount Kenya forest, *Battiscombe K*-*854* (EA, K); Meru, Mount Kenya forest, about 2 km from Meru, Mt. Kenya Lodge in WNW direction, 4 Jan 1986, *Beentje 2691* (EA); Meru, Chuka, 13 Jun 1932, *Graham 1752* (EA, K); Chyulu Hills, main forest North of Camp 3, 18 Feb 2001, *Luke & Luke 7355* (EA); Mount Kenya, from camp on Mount Kenia (2550 meters) westward to the Kasorongai River (1950 meters), 17 Oct 1909, *Mearns 1821* (US); Meru, Nyambeni Hills, base of Kirima Mountain near the Nyambeni Hills Tea Estate, 11 Oct 1960, *Polhill & Verdcourt 297* (BR, EA, K); Marsabit, Mount Kulal, S end, Mar 1972, *Tweedie 4280* (K). **Nyanza**: Kisii, Sep 1933, *Napier 2926* (EA); Kisii, River Riaguma, 11 May 1978, *Plaizier 1321* (EA); Kisii, 30 Oct 1974, *Vuyk & Breteler 51* (EA). **Rift Valley**: Nandi, Kapsabet 6 miles to the W, 31 Mar 1961, *Archer 249* (EA); Kericho, Londiani, Mt. Blackett, 11 Mar 1952, *Argyle 25* (EA); Kajiado, Ol Doinyo Orok, 6 Dec 1944, *Bally 4146* (EA, K); Kericho, Sotik, Cains Farm, 1981 m, 19 Jul 1945, *Bally 4577* (EA, K); Kajiado, Ngong Hills, East valley, 31 Dec 1984, *Beentje 1798* (EA); Uasin Gishu, Soy, Oct 1931, *Brodhurst-Hill 211* (EA, K); Trans Nzoia, Saiwa Swamp National Park, 22 Mar 1992, *Cheseny 488* (EA); Narok, Aitong, Oledebesi-Lemoko hillside behind, Kapkimolwa, about 45 miles North of Aitong, 28 Apr 1961, *Glover et al. 765* (BR, EA, K); Elburgon, 20 miles from Olokurto on road to Elburgon, Nakuru District, 14 May 1961, *Glover et al. 1092* (BR, EA, K); Uasin Gishu, Eldoret-Elgeyo escarpment, *Harger s.n.* (BM); Elgeyo Marakwet, Kapsowar, Kipkunurr Forest Reserve, E end of Cherangani Hills, 14 Apr 1975, *Hepper & Jaeger 4976* (EA, K, P); Trans Nzoia, Mount Elgon, Dec 1931, *Jack s.n.* (EA); Samburu, Leroghi Plateau, Karissia Forest, near Maralal, 30 Jul 1960, *Kerfoot 2116* (EA); Kericho, Tinderet Forest Reserve, Camp 4, Nyanza Province, Londiani District, 29 Jun 1949, *Maas Geesternanus 5246* (BR, K); Nakuru, Eastern Mau Forest Reserve, Camp 9, Rift Valley Province, Nakuru District, 25 Aug 1949, *Maas Geesternanus 5894* (BR, G, K); Mau Forest Reserve, Nakuru District 24 Nov 1971, *Magogo 1517* (EA, K); Mau, summit (Mau Summit), 1928, *Mettam 178* (DS, K); West Pokot, Kapenguria, West Suvi, 6 May 1953, *Padwa 65* (EA, K); Kericho, 6.5 miles to the East in forest along the south perimeter of Sambret Tea Estate, 6 Dec 1967, *Perdue & Kibuwa 9288* (BR, EA, K); Trans Nzoia, ‘Kavirondo’ [Winam Gulf of Lake Victoria, along Nzoia River and W slopes of Mt. Elgon], 1907, *Skene 121* (G); Kajiado, Ngong Hills, Sep 1939, *van Someren 9835* (EA); Trans Nozia, Mount Elgon, foothills, Kabyoyon Valley; Nov 1971, *Tweedie 4135* (FT, K); Kajiado North, Ngong, top of Ngong Hills, 200 m from radar installations, 19 May 2009, *Vorontsova et al. 93* (BM, EA, NY); Nandi, 2nd day’s march from Eldama Ravine, 1898, *Whyte s.n.* (K). **Western**: Kakamega, Yala River, Opposite Nature Reserve, near Quarry Hill, 26 Jan 1982, *Gilbert 6880* (EA, K); Kakamega, Kakamega Forest, NW part of forest, 6 May 1971, *Mabberley 1093* (K); Vihiga, Malaba Forest, Mar 1963, *Tweedie s.n.* (K).

**LIBERIA**. **Nimba**: Clairiére, 5 Jan 1965, *Adam 20447* (K); Mt. Alpha Mine, upper Yiti River, 16 May 1973, *Adam 27565* (P); Mt. Alpha Mine, upper Yiti River, 16 May 1973, *Adam 27588* (P); Yiti Valley, crête orientale, 6 Aug 1974, *Adam 28907* (P); Trig Point 36, 22 Nov 1964, *Adames 779* (K); Mount Nimba, upper edges of the forest on Mount Nimba, 14 Dec 1966, *Bos 2393* (BR, K, P).

**MALAWI**. **Central**: Dedza Mountain Forest, 19 Jan 1987, *Balaka 1859*-*B* (K, NY); Ntchisi Forest, 2 Feb 1983, *Dowsett-Lemaire 618* (BR); Nchisi Mountain, 19 Feb 1959, *Robson 1662* (BM, K). **Northern**: Misuku Hills, Mugesse Forest, 1 Mar 1983, *Dowsett-Lemaire 667* (BR); Chitipa, Muyumba, above the Waterfield’s house, Misuku District, 1926, *Maitland s.n.* (K); Mzuzu, Vipya Plateau, E of Champira, Lwanjati peak, 11 Jan 1975, *Pawek 8927* (EA, K); Mpalampala, highest elevation on Vipya, top of Mpalampala, Nkhata Bay District, Vipya Plateau, 30 miles SW of Mzuzu, 13 Feb 1977, *Pawek 12349* (K); Nkhata Bay District, 5 miles E of Mzuzu, 1 May 1977, *Phillips 2164* (BH); Chikangawa, Nkhata Bay District, 3.5 miles SE of Chikangawa, 10 Sep 1978, *Phillips 3899* (K); Chitipa, Misuku Hills, 10 Jan 1969, *Richards 10596* (K); Mzimba, Mtangatanga Forest Reserve, Mzimba District, 5 Nov 1968, *Salubeni 1195* (K). **Southern**: Zomba District, Zomba Plateau, 27 Jan 1979, *Blackmore 208* (BM, K); Cholo Mountain, Cholo District, 16 Feb 1970, *Brummitt & Banda 8591* (K); Zomba Plateau, by Mlunguzi River near Trout Ponds, Zomba District, 18 Apr 1970, *Brummitt 9975* (K); Naming’omba Tea Estate, at the source of Maponga Stream, Thyolo District, 25 Aug 1982, *Chapman & Tawakali 6377* (BR, K); foot of Mt Mulanje, along Nakolu Stream about Lauderdale estate, 30 Oct 1985, *Chapman & Chapman 6740* (E); Phalombi, Mount Mchese, 5 Jun 2008, *Harris et al. 532* (K); on top of Majemba Hill where the flag is, under the stone, Mulanje, 9 Feb 1980, *Patel & Morris, B. 479* (K).

**MAYOTTE (French Overseas Department)**. Mayotte [Maore], Bandrélé, 14 Apr 1999, *Pignal 1143* (MO).

**MOZAMBIQUE**. **Gaza**: Chirinda zone, picada para o riacho Machecane no lado do picada, 18 Sep 1980, *Nuvungaet al. 318* (BM, K). **Manica**: Mossurize, (Espungabera), Serra de Espungabera (Manica e Sofala), 14 Feb 1966, *Pereira et al. 1358* (BR); Mossurize, (Espungabera), Nova Sintra (Gogoi), Monte Maruma (proximo a fronteira entre os rios Macuha e Nhamoa) (Manica e Sofala), 1 Mar 1966, *Pereira et al. 1585* (BR); Chimoio, prox. da Misao de Amatongas (Manica e Sofala), 10 Dec 1943, *Torre 6283* (BR, EA); Muza, Vallée du Muza, *Vasse s.n.* (P). **Zambezia**: Milange, Serra do Chiparana, subida pelo chefo Marraga, vertiente voltada a NE, 28 Jan 1972, *Correia & Marques 2365* (BR); Gúruè, serra do Gúruè, ao km 3 a seguir a cascata picada para o pico Namúli (Gf), 23 Feb 1966, *Torre & Correia 14805* (K, P).

**NIGERIA**. **Akwa Ibom**: Eket, 1912, *Talbot s.n.* (BM); Bakosi Proposed Reserve. Kumba district, 19 May 1951, *Olorunfemi 30589* (K). **Cross River**: Sankwala area, 30 Dec 1948, *Savory & Keay FHI*-*25255* (K); Oban, 1911, *Talbot s.n.* (BM); Obudu Plateau, 1 Nov 1964, *Tuley 1058* (K). **Edo**: Irhue, Ehor Forest Reserve, Ehor District, 2 Jun 1974, *Eimunjeze Oguntayo FHI-72752* (K). **Ondo**: Akuru Forest Reserve, 15 Aug 1946, *Jones s.n.* (K). **Osun**: Omo Forest Reserve, near PSP.208A’s boundary, 25 May 1960, *Emwiogbon FHI-44266* (K); Shasha Forest Reserve, 20 Apr 1935, *Richards 3373* (BM, BR). **Taraba**: Mambilla Plateau, between Kara and Titong, Sardauna District, 9 Aug 1973, *Chapman 88* (K); Mambilla Plateau, 28 Apr 1972, *Chapman 2777* (K).

**REPUBLIC OF CONGO**. **Brazzaville**: Brazzaville, 22 km N de Brazzaville, Plateaux Batiké, 3 Feb 1970, *Makany 1528* (P). **Lekoumou**: Komono, piste SW, 17 Jan 1965, *Bouquet 957* (P); Meyah, 19 Dec 1969, *Hallé 1715* (P); Fôret de Bangou, près de la grotte Yengo, 6 Feb 1970, *Hallé 1795* (P). **Niari**: Niari region, Col de Bamba, 8 Dec 1990, *La Croix 5027* (BR). **Pool**: Brazzaville, 32 km N de Brazzaville (route Nord), 20 Jan 1971, *Makany 1673* (P). **Sangha**: Nouablé-Ndoki National Park, Goualougo Study Site, 37.92 km E de Bomassa., 17 Apr 2007, *Ndolo E-52* (E); Nouablé-Ndoki National Park, Goualougo Study Site, 36.85 km E-NE de Bomassa, 18 Dec 2008, *Ndolo E-410* (E).

**RWANDA**. **Eastern**: Birenga, pref. Kibungo, route de la frontière du Burundi au S de Bare (sector Igahara), 22 Jun 1978, *Raynal 20648* (P); Forêt de Mukura au N et près de la roche de Ndaba, Isabinyama, Kivumu, Pref. Kibuye, 18 Mar 1980, *Runyinya 958* (BR); Mutara, environ de Mimuli, reserve IRSAC, Terr. Biumba, 23 May 1957, *Troupin 3196* (BR, EA, K); Mutara, colline Karukwanzi, Terr. Biumba, 9 Mar 1958, *Troupin 6631* (BR,K); Mutara, colline Gikandura, Terr. Biumba, 14 Nov 1958, *Troupin 8773* (BR, NY); Mutara, colline Nteko, 17 Jan 1959, *Troupin 9614* (BR); Mutara, Mimuli, Reserve IRSAC, Colline Ntako, rivière Kakitumba, 10 Jun 1959, *Troupin 11702* (US). **Kigali**: Tarabana, Kigali, May 1933, *Becquet 674* (BR). **Northern**: Gishwati, Forêt de Gishwati, route de Gisenyi à Kibuye, a 32 km de Gisenyi, 6 Feb 1972, *Auquier 2434* (BR); Lac Bulera, riv. Kabaga, Pref. Ruhengeri, 15 Feb 1972, *Bamps 3143* (BR); Rukoro, Kidalho, Ruhengeri (pref) Graniko, 23 Mar 1962, *Nshorere 121* (BR). **Southern**: Rubona INEAC, Colline Shyunga, 21 Oct 1958, *Michel 5794* (BR); Rubona INEAC, Colline Gashikeri, 2 Dec 1958, *Michel 5879* (BR); Uwinka, route Bukavu-Astrida, environ d’Uwinka, colline Bunyanguru, Terr. Sangugu, 12 Mar 1959, *Troupin 9826* (BR, EA). **Western**: forêt de Rugege (Cyangugu), au km 91 de la route Butare-Cyangugu, 24 Jul 1974, *Auquier 3368* (BR); Nyungwe, Forêt de Nyungwe (Cyangugu), a km 1.3 de la route de Pindura a la frontière du Burundi, 26 Jul 1974, *Auquier 3438 bis* (BR); Cyangugu, route Butare-Cyangugu vers km 100, 10 Jan 1980, *Bridson 187* (BR, EA, K); Gisovu, Wisumo, 17 Feb 1980, *Bridson 415* (BR, EA, K); Uwinka, piste Uwinka- Rangiro, forêt de Nyungwe, pref. Cyangugu, 3 Mar 1980, *Bridson 488* (K); entre Sisovu et Wisumo, pref. Kibuye, 12 Mar 1973, *Troupin 14630* (BR); Karisimbi, lava field, 25 Feb 1972, *van der Veken 9577* (BR).

**SÃO TOME E PRINCIPE**. **São Tomé**: Lagoa Amelia, pic de M. Café, Sep 1905, *Chevalier 14284 is*, (P); S. Nicolau, 1885, *Moller 95* (BM); Bom Successo, Jul 1885, *Moller 404* (BM); Vermelho, Roça Sandade, 1885, *Moller 516* (BM); Monte Café, Lagina S. Pedro, Oct 1885, *Quintas 920* (BM); Bom Sucesso, chemin Lago Amelia, station Jachere, 31 Jul 1997, *Stevart & de Oliveira 8* (BR); Angra, Angolares, 1886, *Juintas 1018* (BM).

**SIERRA LEONE**. Troma, 16 Dec 1865, *Jaeger 8531* (K). **Eastern**: Tingi Mountains, 12 Dec 1965, *Morton & Gledhill SL*-*2959* (K).

**SOUTH AFRICA**. **Eastern Cape**: 7 miles NW of Port St. Johns, 1949, *Skinner & McGough s.n.* (US); Eggosa Forest, 22 Aug 1969, *Strey 8863* (K). **KwaZulu-Natal**: Ndandhla Forest, 10 miles SE of Nkandhla, 12 Jun 1946, *Codd 1393* (K); Inanda, [Hillcrest], Apr 1879, *Medley Wood 559* (K); Zwaartkop, 16 Feb 1907, *Medley Wood 10241* (P); Otto’s Bluff, Jul 1931, *Rump s.n.* (NU); Eshowe, *Saunders 9* (K). **Limpopo**: Hanertsberg, 21 Oct 1938, *Acocks 1374* (S); Mahonde Mission Station, 15 miles NE of Sibosa, Dist. Zoutpansberg, 19 Feb 1952, *Codd 6844* (K); km 5 from Haenertsburg to Pietersburg, 5 Dec 1975, *Crawford 354* (K); Zoutpansberg, Entabeni Forest, environs de Louis Trichardt (Transvaal) Zoutpansberg Mountains, 7 Sep 1933, *Humbert 10617* (P); Pietersburg, Mountain Home Farm, Woodbush area, 40 miles E, 2 Jan 1945, *Mogg 17435* (BM, BR, K, P); Modjadjes, Div. Pietersburg [Polokwane], Dec 1915, *Rogers 18111* (K); Zoutpansberg, Louis Trichardt, Jun 1918, *Rogers 21326* (BM); Rosendal, near bridge across ‘Mati stream, between the waterfalls, Dist. Duiwelskloof [Modjadjiskloof], 24 Jun 1958, *Scheepers 396* (K); Zoutpansberg, ca. 9 miles W of Louis Trichar[t] farm, Rustfontein, 9 Nov 1955, *Schlieben 7555* (BR, K, NY, US).

**SOUTH SUDAN**. **Equatoria**: Loubouti, Lafuka, Imatong Mtns., Mt. Baghanj, 14 Jun 1939, *Andrews 1430* (K); Talanga, Imatong Mountains, 4 Dec 1980, *Friis & Vollesen 683* (K); Mondalia, Mongalla, Kagelu, *Hamdi 38* (K); Lotti Forest, 24 Jun 1953, *Jackson 3045* (K).

**TANZANIA**. **Arusha**: Monduli, Ketumbeine Forest Reserve, ca. 3 km S of Ketumbeine Peak on foot trail ca. 4 km N of Elang’atadapashi, 1 Apr 2000, *Gobbo et al. 674* (MO, NY); Ngorogoro, E edge of Malanja, 8 Mar 1966, *Henlocker 342* (K); Monduli, Ketumbeine Forest Reserve, 5 Apr 2000, *Massawe et al. 708* (MO, NY); Ngorongoro, near Safari Lodge, 2 Feb 1961, *Newbould 5699* (EA, K, US); Arumeru, Mount Meru, Arusha National Park Near Miriakamba Hut, 9 Oct 1997, *Phillipson et al. 4933* (MO); Ngurdoto Crater National Park, Arusha region, 5 May 1965, *Richards 20353* (EA, K); Mount Meru, Kilinga Forest, 6 Apr 1971, *Richards 26922* (EA, K); Mount Meru, Jekuhama, Arusha National Park, Arusha District, 6 Feb 1970, *Richards* *25346* (K); Ngorongoro Crater, E side of crater, 8 Sep 1968, *Robinette & Gilbert 3125* (EA); Monduli, Monduli Forest Reserve, Mwandeti Forest near Kriganyisho pond, 22 Jan 2001, *Simon et al. 671* (MO, NY); Arusha National Park, Nasula camp, 18 Aug 1969, *Vesey-Fitzgerald 6393* (EA). **Dodoma**: Kolo Hills, C. Katikati, Kolo na Beroko, Katissa mhina, 17 Jan 1938, *Burtt 1141* (K). **Iringa**: Mufindi, forest road from Ngwazi to Lugoda, 20 Mar 1986, *Bidgood & Keeley 368* (EA, K); Ilongelo, near Kiboma, Mar 1954, *Carmichael 419* (EA); Luhega Forest Reserve, Iringa District, 20 Feb 1996, *Frimodt-Moller et al. NG*-*041* (K); Ludewa, Livingstone Mountains, on saddle N of summit of Msalaba Mountain, near foot trail from mission at Luana, 11 Jan 1991, *Gereau & Kayombo 3522* (BR, G, MO); Mufindi, Lake Ngwazi, Dam end, 9 Apr 1986, *Lovett et al. 632* (K, MO); Iringa Rural, New Dabaga/Ulangambi, 15 Jul 1999, *Massawe DA*-*75* (K, MO); Mufindi, Lulanda, village, SW of Fufu forest, 19 Oct 1998, *Mwangoka et al. 66* (MA, MO, P); Uzungwe Mountains National Park, Kilolo District, W of Kidatu dam, 5 Nov 2005, *Mwangoka et al. 4530* (EA); Dabaga Highlands, Ihangana Forest Reserve, near Kibengu, 18 miles South of Dabaga, 14 Feb 1962, *Polhill & Paulo 1479* (BR, EA, K); Ibumu Village, about 30 miles E of Iringa and S of the Gt. N Road, 14 Dec 1961, *Richards 15628* (K). **Kagera**: Bukoba Rural, Minziro Forest Reserve, Nyanja Etagera Forest, 8 Aug 2000, *Festo 702* (MO, NY); Bukoba Dist., Minziro Forest, Nov 1958, *Procter 1055* (EA,K); Ngara Dist., Mumwendo, Mumwendo Ferry, 11 Feb 1960, *Tanner 4703* (BH, BR, EA, K); Buseke, Keza, Bushubi, Ngara District, 20 May 1960, *Tanner 4930* (BH, DUKE). **Kigoma**: Gombe Stream Reserve, Sleeping Buffalo ridge, 14 Feb 1970, *Clutton-Brock 387* (EA); Kigoma, Kakombe Valley, Gombe National Park, at base of lowest waterfall, 6 Mar 1996, *Gereau et al. 5930* (MO); Kabogo Mts, 17 Feb 1963, *Kyoto University Expedition 325* (EA); Gombe Stream Reserve, Middle Kakombe, 5 May 1992, *Mbago & Lyanga 1109* (K); Mkenke, Gombe River, Feb 1972, *Parnell 2284* (EA); Mahali Mountains, Mt. Mhensabantu, 9 Jan 1974, *Uehara 1-80* (EA). **Kilimanjaro**: Rau, Moshi District, Feb 1928, *Haarer 995* (EA, K); Masai Dist., Ngorongoro, Conservation Area, Olmasiko on Endulen road, 15 Dec 1965, *Herlocker 246* (EA); Same, Kanza Village, Gonja Ward, along Yongoma River just below bridge, 3 Dec 2000, *Kindeketa 526* (MO, NY); Masai Dist., Makuyuni, W. Usambaras, Jun 1935, *Koritschoner 609* (EA, K); Marangu, Kilimanjaro, 22 May 1961, *Machangu 67* (EA, K); Same, Mponde Village, South Pare Mountains. Mbaga Ward, Manka/Mponde Mwanyesi Sacred Forest, 13 Nov 2000, *Mlangwa & Kaniki 1094* (MO, NY); Same, Shengena Forest Reserve, Gonja Bombo Beat, W side of Mvaa Subvillage near forest boundary, 8 Aug 2001, *Mlangwa & Msangi 1596* (MO, NY); Pare Dist., Muheza, Amani Bustani, 28 Oct 1969, *Ngoundai 404* (EA, K); Kilimanjaro, track to Shira Plateau, Moshi District, 12 Feb 1969, *Richards 24031* (BR, K); Lemosho, Kilimanjaro, 1 Jan 1967, *Robertson 445* (EA, K); Lyamungu, C.R.S., Moshi District, 21 Dec 1943, *Wallace 1164* (EA, K); Marangu, Kibo Hotel, 7 May 1962, *Wright 6* (K). **Lindi**: Rondo Forest Reserve, Rondo Plateau, Lindi District, 20 Feb 1991, *Bidgood et al. 1674* (K); Rondo Forest Reserve, Lindi District, 22 Nov 1966, *Gillett 17990* (EA, K). **Manyara**: Mbulu Dist., Oldeani, 6 Sep 1968, *Gilbert 3518* (EA); Ngorogoro, Oldean Mountain, 1 Jan 1989, *Pócs & Chuwa 89-002/C* (K); Pienaars Heights, Great North Road, 12 miles S of Arusha, Mbulu District, 5 May 1962, *Polhill & Paulo 2341* (BR, EA, K); Nou Forest Reserve, Tomati, 5 Jan 1968, *Trut 576* (EA). **Mbeya**: Mount Rungwe, W slopes, Lukuyu District, 11 Mar 1932, *St. Clair-Thompson 977* (K); World’s End View, Ipinda, Mbeya Range, 6 Feb 1976, *Cribb et al. 10579* (K); Poroto Mountains, Chumvi Forest, above Irambo, 8 Feb 1979, *Cribb et al. 11340* (EA, K); Mbeya Peak, Myembe, Mbeya Peak Forest Reserve, 28 Oct 1958, *Gaetan 93* (EA); Mbeya Range, 25 Nov 1961, *Kerfoot 3251* (EA); Rungwe Dist., Rungwe Mt., top of mountain, 8 Mar 1950, *Milton 56* (EA); Rungwe Mountain, N slope, Tukuyu District, 8 Feb 1961, *Richards 14331* (EA, K); Elton Plateau, 30 Nov 1963, *Richards 18480* (EA, K); Mount Mbeya, Chunya Escarpment, Mbeya District, 10 Dec 1963, *Richards 18618* (K); north of Lake Nyasa, Nbaka Oberlauf, Station Kyimbila [Rungwe District], 1 Mar 1912, *Stolz 1159* (BM, K). **Morogoro**: Nguru Mountains, 11 Dec 1966, *Beesley 285* (BR, K); Chenzema, 1 km before town on roadside, Uluguru Mountains, 26 Jan 1976, *Cribb & Grey-Wilson 10441* (K); Mount Mnyera, Ukaguru Mountains, Kilosa District, 31 Jan 1976, *Cribb & Grey-Wilson 10489* (K); Ulanga Dist., Mahenge, Nawenge Forest Reserve, 2.5 km SW of Mahenge, 20 Feb 2006, *Gereau et al. 6891* (EA, NY); Mwere River, Uluguru Mts., 26 Sep 1970, *Harris et al. 5155* (EA); Mamiwa Forest Reserve, Ukaguru Mountains, ridge to the N of Mandege Forest Station, Kilosa District, 2 Aug 1972, *Mabberley 1346* (K); Maskati Mission, Nguru Mountains, 29 Jan 1991, *Manktelow et al. 91009* (K); Morogoro Rural, Uluguru Mountains, Bunduki Forest Reserve, 29 Aug 2000, *Mhoro UMBCP*-*474* (K, MO); Morogoro Rural, Nguru South Forest Reserve, Kwechazungu, Dikulula River, 21 Aug 2004, *Mwangoka et al. 3305* (MO); Ilole Forest, Mikumi Divison, Kiluma District, 9 Feb 2007, *Mwangoka & Kidanka 5121* (BR); Ukaguru Mountains, along track between Mandege and Vingwete, Kilosa District, 3 Jun 1978, *Thulin & Mhoro 2916* (K). **Njombe**: Njombe, Matamba, Kitulo Plateau above Matamba, below waterfall on headwaters of Ndumbi River, 22 Nov 1986, *Brummitt & Congdon 8112* (K); Njombe, Aug 1931, *Hornby & Hornby 36* (EA, K);Kipengere Mountains, Nyarere River, Njombe District, 9 Jan 1957, *Richards 7639* (K); Elton Plateau, foothills to Kepengere Mtns. [Kitulo Plateau], 26 Jan 1961, *Richards 14194* (K). **Ruvuma**: Songea, Mitanga area, Magengu, 21 Oct 1956, *Mgaza 120* (EA, K); Songea, Lupembe Hill, 29 Feb 1956, *Milne-Redhead & Taylor 8909* (BR, EA, K). **Shinyanga**: Shinyanga, 11 Mar 1937, *Burtt 5568* (BM, EA). **Singida**: Singida Rural, Mangonyi, Ikungi Div., about 25 km on rd E & S from Ikungi, then 1.2 km SW along old track17 Mar 2002, *Kuchar 25227* (MO, NY); Itigi, Road Itigi-Singida, Itigi thicket, 26 Mar 1965, *Richards 19926* (K). **Tabora**: Igunga road, Tabora region, 28 Nov 1978, *Lawton 2155* (K). **Tanga**: Vugiri, Lushoto District, 12 Jun 1963, *Archbold 216* (K); Kwakomo Forest Reserve, East Usambaras, SE of Kwakoro Tea Estate, Muheza District, 28 Oct 1986, *Borhidi 86260* (K); Kitivo-Lushoto, 7 Mar 1974, *Damaris 2* (K); Bumbuli, West Usambaras, 1.5 miles NE of Sakarani, 6 May 1953, *Drummond & Hemsley 2410* (BR, EA, FT, K); Soni, W Usambaras, Lushoto District, 24 Jun 1971, *Faulkner 4603* (BR, K); Korogwe, Ambangulu Forest Reserve under TFCG control, SE of Makweli village near tea estate plot #18, 1 Feb 1999, *Mwangoka* *289* (MO, P); Korogwe, Tamota village in cultivated area along road to village, 13 Apr 1999, *Mwangoka 418* (MO, P); Korogwe, Kieti Village, forest patch near Mbaha River and tea estate forest, 24 May 1999, *Mwangoka 578* (MO); Kwetango, village, NE part of forest, Muheza district, 23 Feb 2008, *Mwangoka et al. 5638* (BR); Mkusa Forest Reserve, Lushoto District, 17 Jan 1967, *Richards 22005* (K); Lushoto, Camphor near Staff-quarters, 1 Mar 1969, *Shabani 318* (BR, EA, K); Magamba Forest Reserve, near the Magamba Forestry Office, 23 Mar 2010, *Tepe et al. 2783* (BM, DSM); Korogwe, Kunga Tea Estates, West Usambaras; Bungu, Masange Forest, 22 Mar 2010, *Vorontsova et al. 163* (BM, DSM); Bomole, 10 Aug 1916, *Zimmermann 8065* (EA).

**UGANDA**. **Central**: Entebbe, Victoria Nyanza, 21 Apr 1905, *Bagshawe 679* (BM); Buikwe, Mabira Forest, Kyagwe, Sep 1933, *Brasnett 1388* (K); Mengo, Kajansi, Entebbe, Jun 1935, *Chandler 1247* (BR, EA, K); Mabira forest, W. Umbango, Jan 1916, *Dümmer 1387* (BM, P, US); Mulange, Maburu, Nov 1922, *Dümmer 5608* (K); Mabira Forest, 2 km N of Namanyama-Mabira, 1 Jun 1971, *Katende 964* (EA, K); Kisimbiri, on the trail from Entebbe, Victoria Nyanza, to Butiaba, Albert Nyanza, 23 Dec 1909, *Mearns 2457* (BM, NY, US); Entebbe, mile 13 Kampala-Entebbe Road, Feb 1931, *Snowden 1919* (K). **Eastern**: Teso, Serere, Sep 1932, *Chandler 959* (EA, K, S); Mabura, Sep 1920, *Dümmer 4492* (K); Busoga, Lolui [Dolwe Islsand], Jun 1960, *Goodall 19* (EA); Mount Elgon, Dec 1931, *Mrs Jack s.n.* (K); Mbale, Namasindwa, Mt. Elgon, 25 May 1924, *Snowden 889a* (EA, K, P); Elgon, Namasindo, *Snowden 889* (BM, BR); Bugishu, Mount Elgon, Namasindo, *Snowden 890* (BM, K); Bunya, Busoga, Nov 1937, *Webb 62* (K). **Northern**: Karamoja, Morongole [Mount Morungole], Jun 1946, *Eggeling 5655* (EA). **Western**: Budongo Forest Reserve, Masindi District, Nyakafunjo Block, ca. 2.5 km N of Nyabyeya Forestry College, 18 Jun 1998, *ATBP 554* (MO); Kireba Gap, Kigezi District, area between hills and camp, Dec 1938, *Chandler 2644* (BR, K); Bundibungyo, Semliki Forest, 1905, *Dawe 672* (K); Kinkisi, Siba Forest, Kinkizi County, Bunyoro District (NE Albert); about 4 miles S of Susingoro hill, 25 miles W of Masindi, near boundary of coupes 4 and 5 of Siba concession, 21 May 1951, *Dawkins D*-*751* (K); Kabula, on Masaka-Ankole border, Oct 1932, *Eggeling 695* (K); Mbarara, Mar 1968, *Harrington 302* (EA); Ntungamo, Ankole, Ruizi River, 21 Nov 1950, *Jarrett253* (EA); Echuya Forest Reserve, 25 Apr 1970, *Katende K*-*221* (EA, K); Bushenyi District, Central Kashoha forest, 21 Sep 1987, *Katende 3197* (MO); Kibale National Forest, edge of Cyperus swamp near Rweimba bridge, N of MUBFS, 13 Aug 2003, *Knapp & Mallet 9811* (BM, NY); Bwindi Impenetrable Forest, Kayonza Sector, Ishasha Gorge, 10 Feb 1998, *Eilu 247* (K); Bujuko, Mwakoto County, 2 km E of Bujuko, on Mubende road, 26 Feb 1969, *Lye 1950* (K); Bunyaruguru, Aug 1938, *Purseglove P*-*358* (K); Kigezi District, path from Chehafi to Behungi, 2 miles from and behind lake (N.q.), 13 Jan 1933, *Rogers & Gardner 358* (BR, K); Buhweju, Kamukaki-Rugongo, 31 Jul 1992, *Rwaburindore 3430* (MO); Budongo Forest, Bujenje, 16 Jul 1971, *Synnott 617* (EA, K); Kigezi District, Virunga Mountains, pass betwen Mgahinga and Sabinio, 21 Nov 1934, *Taylor 1875* (BM); Bukenyi, Ankole, 2 Sep 1938, *Thomas 2520* (BR, K).

**YEMEN**. **Ibb**: Wadi Bana, c. 15 km south of Yarim, c. 15 km east of main Sana’a to Taiz Road, near As Saddah, 13 Mar 1981, *Miller 3025* (E); Jebel Badaan [Jabal Ba’dan], 26 Aug 1977, *Wood 1864* (BM). **Sana’a**: Bei Hajjera, Menacha, 13 Mar 1983, *Müller-Hohenstein & Deil 1353* (E).

**ZAMBIA**. **Copperbelt**: Chingola, 25 Aug 1954, *Fanshawe 1480* (K); Ndola, 5 Mar 1956, *Fanshawe 2817* (BR, K). **North-Western**: Mwinilunga, Mwinilunga, West Lunga River at Mwinilunga, 23 Jan 1975, *Brummitt et al. 14028* (K); Solwezi, 6 km W of Solwezi, 18 Mar 1961, *Drummond & Rutherford-Smith 6998* (K); by R. Matonchi-Luao junction, Mwinilunga District, 18 Dec 1937, *Milne-Redhead 3723* (BR,K); Mwinilunga, 56 miles south of Mwinilunga on road to Kabompo, 3 Oct 1952, *White 3442* (BM, BR, K). **Northern**: Mbala, Abercorn District, Tasker’s deviation Chilongowelo, 23 Jan 1955, *Richards 4231* (BR, K).

**ZIMBABWE**. **Manicaland**: Melsetter, Bridal Veil Falls, 11 Jan 1974, *Bamps et al. 763* (BR); Inyanga, Nyangami, 24 Nov 1949, *Chase 1839* (BM); Umtali, Norris farm, 11 Jan 1951, *Chase 3660* (BM, K); Melsetter, Sona farm, 15 Mar 1953, *Chase 4872* (BM, BR); Cross Hill, Commonage, Umtali District, 4 Feb 1950, *Corner s.n.* (E); Umtali, Bant i Forest, 4 Feb 1955, *Exell et al. 191* (BM); Makone, Rusape, Dec 1919, *Eyles 5202* (K); Inyanga, 30 Oct 1930, *Fries et al. 2434* (BM, BR, NY); Stapleford Forest Reserve, Central Patrol, 13 Jun 1934, *Gilliland B*-*314* (BM); Chimanimani, Gungunyana Forest Reserve, Melsetter, top of Chiredza Gorge, Nov 1961, *Goldsmith 99/61*, (K); Nyanga, Rhodes Inyanga Orchards, 23 Mar 1966, *Simon 769* (K). **Mashonaland East**: Wedza, Wedza Mountain, 27 Feb 1964, *Wild 6343* (BR, K).

#### Solanum trichopetiolatum

Taxon classificationPlantaeSolanalesSolanaceae

D’Arcy & Rakot., Fl. Madag., Fam. 176: 130. 1994.

Figure 28

##### Type.

Madagascar. Antsiranana: pentes orientales du massif de Marojejy (Nord-Est) à l’Ouest de la rivière Manantenina, affluent de la Lokoho, 1500 m, 24 Mar 1949, *H. Humbert 23653* (holotype: P [P00352290]; isotype: MO [MO-3707383], K [K000414176]).

##### Description.

Shrub (*Ravelonarivo & Rabesonina 636*) or slender liana to 8 m in forest canopy. Stems flexuous, ribbed, variably pubescent with simple uniseriate trichomes 0.7–2 mm long, these appressed or spreading, often concentrated around the leaf bases, glabrescent; new growth glabrous or pubescent with simple uniseriate trichomes to 1 mm long. Bark of older stems longitudinally ridged when dry, dark orange to dark red, glabrescent. Sympodial units plurifoliate, the leaves not geminate, evenly distributed along young branches. Leaves simple, 5–10 cm long 1.8–2.5 cm wide, oblong to obovate, thick-chartaceous, sometimes shiny, weakly to strongly discolorous, glabrous on both surfaces or sparsely pubescent in the basal part with simple uniseriate trichomes along the margins; major veins 4–12 pairs, spreading at 45–60° to the midvein and forming loops, the finer venation not visible, or if visible seen as a delicate network; base equal or slightly oblique, short-attenuate; margins entire; apex acuminate to caudate; petiole 0.6–1.2 cm long, canaliculate, pubescent with simple uniseriate 4–8-celled trichomes 0.5–1.5 mm long, these denser along the margins, not apparently twining. Inflorescences terminal at the apex of long slender main branches, 5–12 cm long, furcate or branched, with 3–7 flowers; peduncle 1–7 cm long glabrous, or basal part of peduncle rarely pubescent with simple uniseriate trichomes like those on the stem; pedicels 1.2–2 cm long, apically dilated, glabrous, articulated 0–0.5 mm from base, often leaving a prominent small peg on the rachis; pedicel scars irregularly spaced 0.2–2 cm apart. Buds ellipsoid, the corolla soon exserted from the calyx tube before anthesis. Flowers 5-merous, apparently all perfect. Calyx 1–2 mm long, an open cup, the lobes ca. 1 mm long, ca. 1.5 mm wide at base, broadly deltate to almost absent, unusually to 2 mm long and linear (*Birkinshaw 2181*), usually rounded to cuspidate at the tips, glabrous with a few simple papillae at the tips, the margins thickened and white in dry material. Corolla 1.2–2.2 cm in diameter, violet, stellate, lobed almost to base, the lobes ovate, 7–12 mm long, 4–5 mm wide, largely glabrous on both surfaces, with minute simple trichomes along and around the margins adaxially. Stamens equal; filament tube 0.5–1 mm; free portion of the filaments 1.5–2 mm long, glabrous; anthers 3–4 mm long, ca. 1.5 mm wide, ellipsoid, not connivent, smooth abaxially, poricidal at the tips, the pores much smaller than anther apices, ca. 0.3 mm in diameter, clearly delineated and not lengthening with age. Ovary conical, glabrous; style 6–9 mm long, protruding 2–4 mm beyond the anthers, straight or curved, glabrous; stigma capitate, the surface finely papillose. Fruit a globose berry, ca. 9 mm in diameter (immature?), green, mature colour not known, the pericarp thin, collapsing on drying to reveal the outline of the seeds, glabrous; fruiting pedicels ca. 2 cm long, ca. 0.6 mm diameter at base, pendulous; fruiting calyx slightly accrescent, the lobes spreading and somewhat reflexed. Seeds 4–8(-10) per berry, 3–4 mm long, ca. 2.5 mm wide, flattened reniform, dull orange-yellow; the surface deeply pitted, the testal cells sinuate in outline.

**Figure 28.**
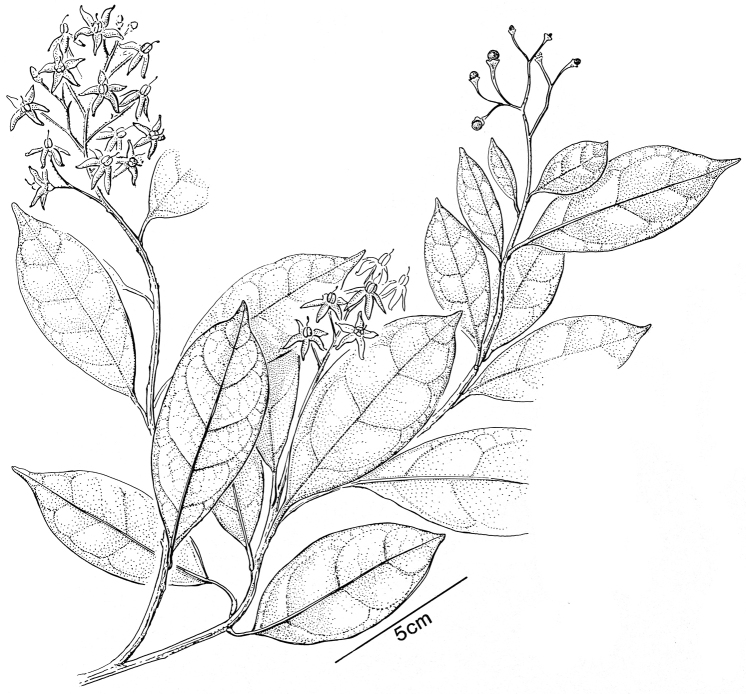
*Solanum trichopetiolatum* D’Arcy & Rakot. Flowering branch (based on *Humbert 23653*). Adapted from D’Arcy and Rakotozafy (1994) with permission of Muséum National d’Histoire Naturelle (see text for explanation of changes from original).

##### Distribution

**Figure 29.**
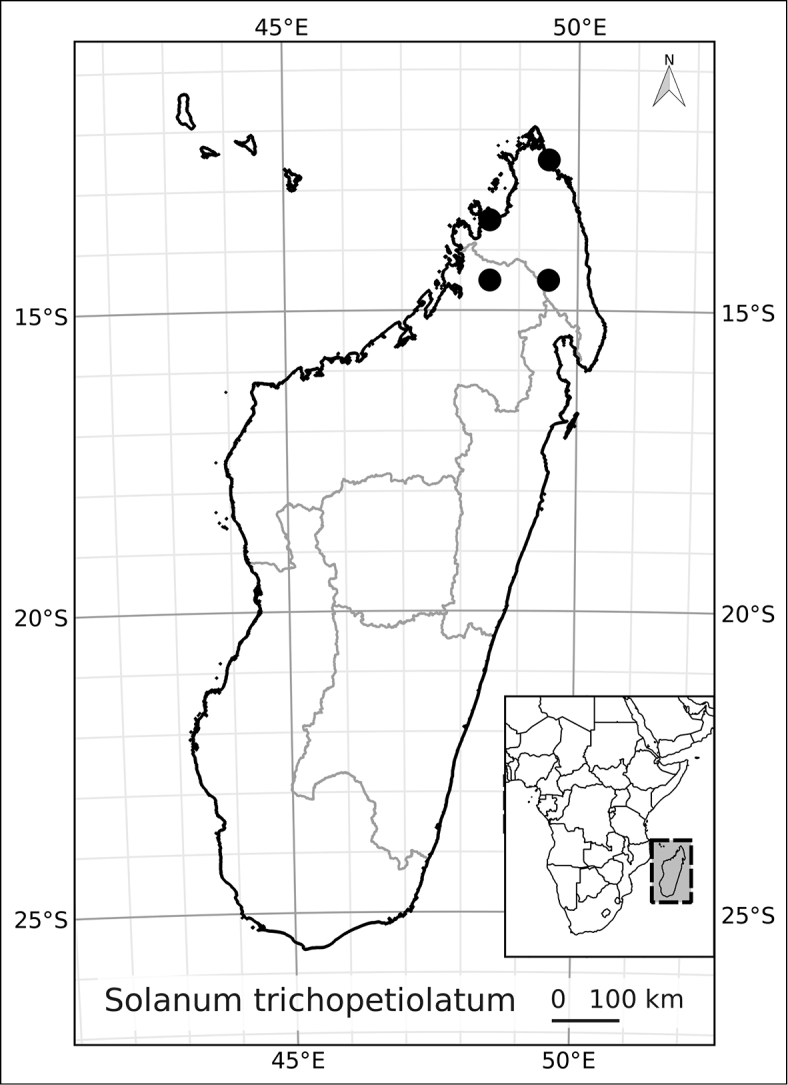
Distribution of *Solanum trichopetiolatum* D’Arcy & Rakot.

(Figure 29). Endemic to northeastern Madagascar in the area around the mountain of Marojejy, Antsiranana and Mahajanga provinces.

##### Ecology and habitat.

Wet montane forests, often growing as a epiphyte in cloud forests; 500 to 1500 m elevation.

##### Common names and uses.

Madagascar. Antsiranana: liaña vato (*Miller & Lowry 4072*).

##### Preliminary conservation status

(IUCN 2014). Vulnerable (VU B1a, biii). EOO 12,121 km^2^ (VU), AOO 28 km^2^ (EN). *Solanum trichopetiolatum* is a species of wet forests, and these are shrinking in extent due to habitat alteration. It does, however, occur in protected areas (e.g., the Marojejy Reserve). In common with other members of the ANS clade in Madagascar, the paucity of collections, indicative of local rarity, coupled with the ongoing habitat threats in Madagascar, indicate monitoring and further collection to assess local rarity are priorities.

##### Discussion.

*Solanum trichopetiolatum* is a slender liana endemic to northern Madagascar, with obovate leaves and pale violet flowers. The species is characterised primarily by its unusual pubescence of straight or curved simple 4-8-celled appressed or spreading trichomes 0.5-1.5 mm long on the petioles and sometimes on the young stems. *Solanum trichopetiolatum* is the only one of the several species morphologically similar to *Solanum madagascariense* described by D’Arcy and Rakotozafy (1994) that we consider distinct enough to merit species status. In addition to the indumentum described above, *Solanum trichopetiolatum* can be distinguished from the widespread *Solanum madagascariense* by its slender, loose inflorescences (Fig. 28) with usually fewer than 10 flowers (versus denser more robust inflorescences with more than 10 flowers), glabrous (versus glabrous to densely pubescent) corolla, and leaves that are more obovate and discolorous on herbarium specimens than is typical of *Solanum madagascariense*. *Solanum trichopetiolatum* differs from *Solanum humblotii* in its possession of a branched inflorescence with usually more than 10 flowers, rather than a few-flowered, unbranched inflorescence. It can also usually be distinguished by its calyx that is not lobed or has minute lobes to only 1 mm long (versus calyx lobes 3-4.5 mm long), but *Birkinshaw 1281* from 2500 m elevation in Mahajanga has the distinctive trichomes of *Solanum trichopetiolatum* but has unusual filiform calyx lobes ca. 2 mm long. Although the plate of *Solanum trichopetiolatum* in D’Arcy and Rakotozafy (1994) illustrates what appears to be a papillate anther, the protologue describes the anthers as smooth; all specimens we have seen have smooth, rather than papillate anthers. We have removed this part of the original illustration of D’Arcy and Rakotozafy (1994) in our Fig. 28.

The part of Antsiranana surrounding the mountain of Marojejy has been identified by D’Arcy (1992) as an area of high diversity for wet forest *Solanum*. The overall aspect of *Solanum trichopetiolatum* on herbarium sheets is highly reminiscent of those specimens considered by D’Arcy and Rakotozafy (1994) to belong to *Solanum marojejy* (here recognised as belonging to *Solanum madagascariense*), and leaves of the two species dry a similar dark golden-brown colour on herbarium sheets; the long trichomes and slender inflorescences of *Solanum trichopetiolatum* allow easy identification. Field and population studies are necessary to further understand the relationship between *Solanum trichopetiolatum* and *Solanum madagascariense*.

##### Specimens examined.

**Madagascar. Antsiranana**: Ambilobe, Marojenty, contreforts occidentaux du massif de Marojejy (Nord-Est) près du col de Doanyanala (limite des bassins de la Lokoho et de l’Andraronga), 1949, *Humbert 23075* (P); Reserve Naturelle de Marojejy, along the trail to the summit of Marojejy Est, NW of Mandena., 14 Feb 1989, *Miller & Lowry 4072* (MO, TAN); Antsiranana Rural, Antsiranana Reserve nationale n. 12-Marojejy; sentier qui mène au 3r camp, au dessus du village de Manentenina, 27 Mar 1990, *Randrianasolo 107* (MO, P, TAN); Réserve naturelle intégrale 12, Marojejy, au nord d’Andapa, aux environs du sommet de l’Est, 21 Jan 1994, *Rasoavimbahoaka et al. 8* (K, MO, P, TAN); Préfecture d’Antalaha, Sous-Préfecture d’Andapa, commune rurale de Bealampona, Quartier de Befingotra. Sud-Ouest d’Andapa, Réserve Spéciale Anjanaharibe-Sud, village de Mandritsarahely, suivant la rivière de Andranomenabe de la route nationale approchant la chaîne d’Anjanaharibe-Sud, 14 Feb 1995, *Ravelonarivo & Rabesonina 636* (MO, TAN); Sous-Préfecture d’Andapa, Commune rurale d’Ambodimanga I, Quartier d’Andilandrano, environ de Hiakan’ny Zamandrabosy, 6 km à l’ouest d’Andilandrano, dans la réserve d’Anjanaharibe-Sud, 4 May 1995, *Ravelonarivo & Rabesonina 792* (MO, P, TAN). **Mahajanga**: ruisseau d’Andasinananatsomanga Amparihy, commune rurale de Matsoandakana, 24 Feb 2008, *Bernard et al. 891* (MO, TAN); Tsaratanana Massif, ridge 2 km N of Mahatsabory-Mica, 23 Feb 2003, *Birkinshaw 1281* (G, MO).

#### Solanum truncicola

Taxon classificationPlantaeSolanalesSolanaceae

Bitter, Bot. Jahrb. Syst. 54: 435. 1917, as “truncicolum”

Figures 1F , 30

##### Type.

Madagascar. Fianarantsoa: “Süd-Betsiléo, Wald von Ankafina”, Mar 1881, *J.M. Hildebrandt 3954* (lectotype designated here: JE [JE00004288]; isolectotypes: B [destroyed], BM [BM000797934], G [G00442590], K [K000414186], M [M0105578], W [W18890032421]).

##### Description.

Epiphytic shrub or liana, 1–1.5 m tall. Stems terete, ribbed or slightly winged, glabrous, the short shoots on larger plants occasionally minutely pubescent with simple uniseriate trichomes ca. 0.1 mm long, glabrescent; new growth glabrous or minutely puberulent. Bark of older stems smooth or longitudinally ridged, light grey to brown, glabrous. Sympodial units plurifoliate, the leaves not geminate, evenly distributed along young branches or clustered on short shoots. Leaves simple, 1.5–8(13) cm long, 0.5–3 cm wide, lanceolate to elliptic or oblong, coriaceous to thick chartaceous, drying concolorous, dull green to reddish brown, glabrous on both surfaces or the abaxial midvein and margins sometimes sparsely pubescent with simple uniseriate trichomes ca. 0.1 mm long; major veins 4–5 faint pairs at ca. 60° to the midvein, the finer venation not visible; base cuneate to attenuate, occasionally long attenuate with lamina extending to the base of the petiole; margins entire, slightly revolute in herbarium specimens, occasionally minutely pubescent with dendritic or simple trichomes like those on the midvein; apex acute; petiole 0.1–0.5 cm long, thick, drying ridged, glabrous or with a few simple uniseriate trichomes like those of the midvein abaxially. Inflorescences terminal at the apex of short slender lateral branches, appearing axillary when terminal shoot is condensed, 2–4 cm long, unbranched, with 1–2 flowers, glabrous; peduncle absent; pedicels 1.3–2 cm long, apically dilated, ridged in dry material, usually glabrous, sometimes with sparse erect simple multicellular hairs up to 0.15 mm long, articulated 0–1 mm from base; pedicel scars indistinct. Buds oblong, the corolla immediately exserted from the calyx tube in bud long before anthesis, exceeding the calyx lobes. Flowers 5-merous, apparently all perfect. Calyx tube ca. 1 mm long, openly cup-shaped, the lobes 0.4–1.1 cm long, 1–1.5 mm wide at base, linear to ovate, obtuse to acute at the tips, foliaceous, glabrous or with occasional simple trichomes on the margins. Corolla 1.7–3 cm in diameter, white to pale or dark purple, stellate, lobed almost to base, the lobes 8–15 mm long, 2–4 mm wide, narrowly elliptic, occasionally expanded near the base with what look like wings, often aristate with a subulate appendage ca. 1 mm long arising from the adaxial surface of the lobe just below the tip, glabrous adaxially, with enlarged papillose cells and unicellular to simple uniseriate trichomes on the tips and margins. Stamens equal; filament tube 0.5–1 mm; free portion of the filaments ca. 1.5 mm long, glabrous; anthers 5–6 mm long, ca. 1.5 mm wide, ellipsoid, loosely or tightly connivent, poricidal at the tips, the pores slightly smaller than anther apices, not lengthening with age, the surface dark and papillose adaxially. Ovary conical, glabrous; style 6–9 mm long, protruding 1–2.5 mm beyond the anthers, curved, glabrous; stigma clavate, the surface smooth. Fruit an ovoid berry, ca. 1.1 cm long, ca. 0.6 cm in diameter (immature), apically pointed, the pericarp thin, glabrous; fruiting pedicels ca. 3 cm long, ca. 0.8 mm in diameter at base, pendent or spreading, ridged; fruiting calyx lobes up to 8 mm long, accrescent and as long as or longer than developing fruit. Seeds not known.

**Figure 30.**
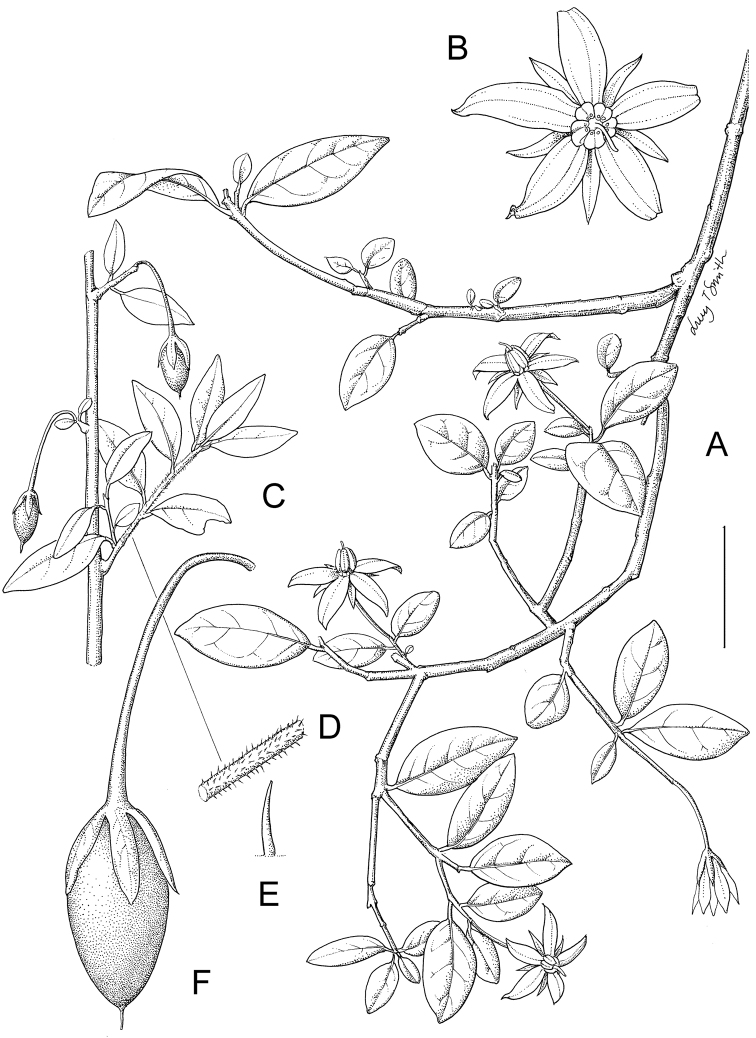
*Solanum truncicola* Bitter. **A** Flowering branch **B** Open flower **C** Fruiting branch **D** Stem showing minute simple pubescence **E** Simple trichome **F** Mature (?) berry. (Based on: **A** *Malcomber et al. 1353*; **B** *Philipson et al. 5875* drawn from photographs; **C–F** *Randrianasolo et al. 1163*). Scale bar: **A, C** = 3 cm; **B** = 1.5 cm; **D, F** = 7 mm; **E** = 0.3 mm. Drawn by Lucy T. Smith.

##### Distribution

**Figure 31.**
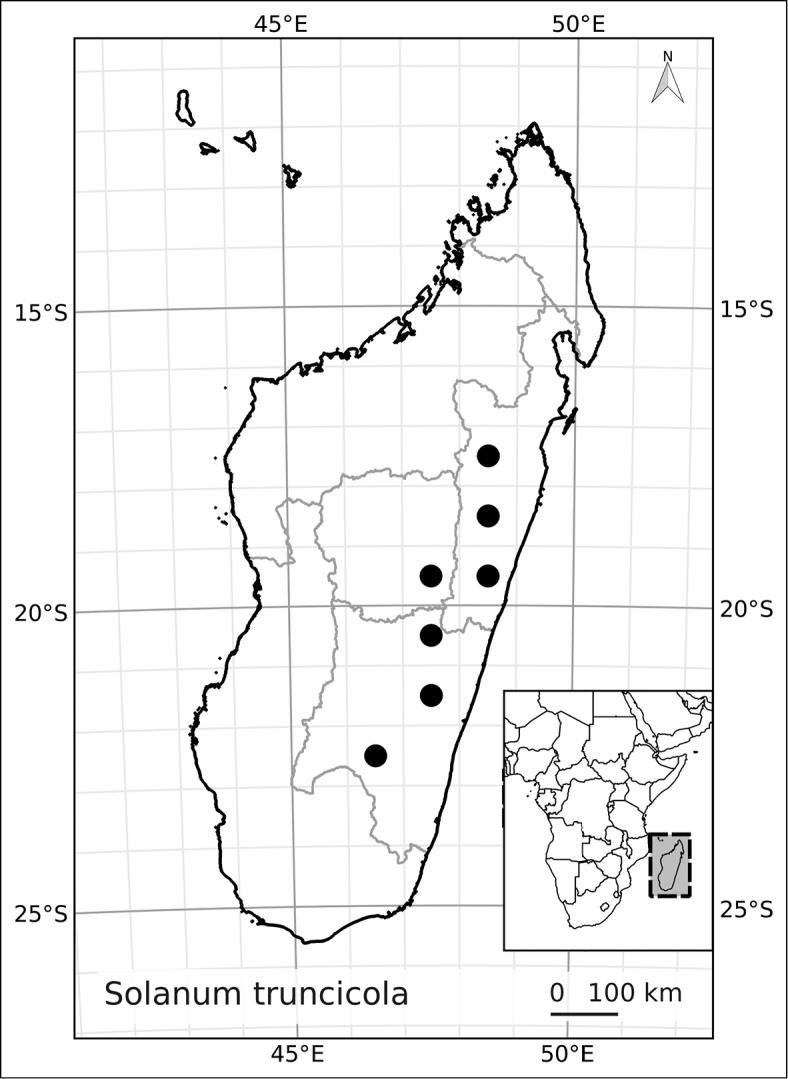
Distribution of *Solanum truncicola* Bitter.

(Figure 31). Endemic to the east-central region of Madagascar in Antananarivo, Fianarantsoa, and Toamasina provinces.

##### Ecology and habitat.

Humid and subhumid montane forest; (600-)1000–2000 m elevation.

##### Common names and uses.

Madagascar. hendramena, farafandoha (D’Arcy and Rakotozafy 1994, as *Solanum humblotii*).

##### Preliminary conservation status

(IUCN 2014). Vulnerable (VU B1a, biii). EOO 19,845 km^2^ (VU), AOO 56 km^2^ (EN). Although *Solanum truncicola* has been been consistently but sparsely recorded over a coherent stretch of the eastern Malagasy rainforest from Toamasina in the northernmost part of its distribution range to the Ambalavao area in the southernmost part, the highly fragmented nature of these eastern forest habitats gives cause for conservation concern.

##### Discussion.

*Solanum truncicola* is a rare and unusual montane forest shrub or epiphyte. It is easily identifiable by its coriaceous leaves and long, almost linear calyx lobes which are divided to the base before the corolla begins to expand (Fig. 30A). The length of the calyx lobes (0.4–1.1 cm) in combination with the deeply stellate corollas with narrow lobes makes its flowers appear to be two overlapping stars (see Fig. 1F). Petioles of *Solanum truncicola* are the shortest of all Madagascar *Solanum* species (1–5 mm), usually less than 1/8 of the total leaf length. The flowers can reach 3 cm in diameter, and anthers to 6 mm in length.

Misidentification of *Solanum truncicola* is not likely, even though its height, internode length, and leaf size vary spectacularly across its distribution range: leaves of the type specimen are ca. 1.5 cm long, while leaves on other collections reach 13 cm. Some large leaved forms become reminiscent of *Solanum myrsinoides* in habit and can be distinguished from *Solanum myrsinoides* by their inflorescences of 1–2 (versus 2–6) flowers and calyx lobes 0.4–1.1 cm (versus less than 0.1 cm) long.

Bitter (1917) correctly described *Solanum truncicola* as distinct from *Solanum humblotii*. *Solanum truncicola* can be distinguished from *Solanum humblotii* by its petioles 0.1–0.5 cm (versus 0.6–1 cm) long, acute (versus acuminate) leaves, glabrous (versus pubescent) young stems, narrowly ovate to obovate (versus deltate to acuminate) calyx lobes, and a different overall appearance. D’Arcy and Rakotozafy (1994) included *Solanum truncicola* as a synonym of *Solanum humblotii*, perhaps because Bitter’s key only highlighted the more superficial indumentum and size differences between the two species, and the type collections were not carefully consulted. During the years that followed D’Arcy and Rakotozafy’s treatment, many specimens of *Solanum truncicola* were determined as *Solanum humblotii*; this has caused considerable confusion in herbaria. The two species occur in similar wet forest habitats but are allopatric with *Solanum truncicola* having a more southerly distribution and at higher elevations than *Solanum humblotii*.

Although the original spelling of the epithet is “*truncicolum*” (Bitter 1917) the word element -*cola* should be treated as a noun in apposition and this is a correctable error to *truncicola* (Article 23.5, McNeill et al. 2012).

Label details on *Malcomber et al. 1353* (MO) record the pedicel and calyx as dark purple. The anthers on at least one flower of *Malcomber et al. 1353* are clearly connate but distinctly free on other flowers of the same specimen and on other collections.

Bitter (1917) cited three herbaria in the protologue – G-Boiss., B and “Haussknecht” (now held in JE). We have selected the duplicate at JE as the lectotype because it bears an annotation label in Bitter’s handwriting and is a larger specimen with more flowers than that at G.

##### Selected specimens examined.

**Madagascar. Fianarantsoa**: Ivohibe, Andringitra, Andringitra, camp IV, ca. 38 km S of Ambalavao, Andringitra reserve on ridge above headwaters of Sahavatoy river, Dec 1993, *Lewis et al. 1083* (P); Ifanadiana, Parc National de Ranomafana, S of Namorona river, parcelle III, trail to Maharira, C. 5 km N of Maharira, 1 Feb 1992, *Malcomber & Rakoto 1353* (MO, P, TAN); Ambositra, between Ambositra and Fianarantsoa, 16 Mar 2005, *Phillipson et al. 5785* (MO); Ambalavao, Andrisoa, district Ambalavao, 16 Feb 1954, *Rakoto & de la Croix 6481* (MO); Andringitra RNI, District d’Ambalavao, Canton de Sendrisoa, 13 Jan 1958, *Réserves Naturelles Madagascar 9918*-*RN* (MO, P); Ranomafana National Park, parcelle 3, S of National Road 25 at 7 km W of Ranomafana, Talatakely trail system, trail AA 0 m [beginning of trail], 18 Mar 1993, *Turk et al. 341* (NY, TAN). **Toamasina**: Atsinanana, District Brickaville, Commune Maroseranana, Fokotany Ambodilendemy, Ankerana, 26 Mar 2011, *Antilahimena et al. 7846* (MO); Rombevavy, Rahobevava, 11 Mar 1951, *Cours 4275* (MO, P, TAN); Aloatra-Mangoro, forêt de Perinet, au Nord à 7 km de Perinet vers Tamatave, 28 Feb 1971, *Cremers 1481-2* (TAN); Moramanga, Andasibe, Andasibe (Perinet), N of road from Antanarivo to Tametave, NW of Izouard graphite mine on road to col de la Mantady, 1987, *Lowry & Schatz 4286* (MO, P, TAN); Chutes de la Mort, route Anosibe-Moramanga, 11 Oct 1960, *Peltier & Peltier 2676* (P); Aloatra-Mangoro, Corridor Forestier Analamay Mantadia, 24 Apr 2012, *Rasoazanany & Ratolojanahary Tafita 93* (MO, P, TAN); Alaotra Mangoro, Lakato, forest E of Manasamena village, along the Ankandrabe trail, 2 Jun 2007, *Randrianasolo et al. 1163* (NY).

### Names not validly published

*Solanum rubrum* Drège ex Dunal, Prodr. [A. P. de Candolle] 13(1): 77. 1852. Pro. syn. Solanum quadrangulare var. sinuatoangulatum Dunal = *Solanum africanum* Mill.

*Solanum rupicolum* Bojer ex Dunal, Prodr. [A.P. de Candolle] 13(1): 85. 1852. Pro. syn. *Solanum imamense* Dunal = *Solanum imamense* Dunal (herbarium name on holotype specimen of *Solanum imamense*, *Bojer s.n.* at G)

*Solanum pentapetaloides* Bojer ex Dunal, Prodr. [A.P. de Candolle] 13(1): 85. 1852. Pro. syn. Solanum imamense var. grandiflorum Dunal = *Solanum imamense* Dunal (herbarium name on holotype specimen of Solanum imamense var. grandiflorum, *Bojer s.n.* at G)

*Solanum togoense* Dammer, in Schlechter, Westafr. Kautschuk-Exped. 312. 1900. Nom. nud. = *Solanum terminale* Forssk.

## Supplementary Material

XML Treatment for Solanum africanum

XML Treatment for Solanum betroka

XML Treatment for Solanum guineense

XML Treatment for Solanum humblotii

XML Treatment for Solanum imamense

XML Treatment for Solanum ivohibe

XML Treatment for Solanum macrothyrsum

XML Treatment for Solanum madagascariense

XML Treatment for Solanum myrsinoides

XML Treatment for Solanum runsoriense

XML Treatment for Solanum sambiranense

XML Treatment for Solanum terminale

XML Treatment for Solanum trichopetiolatum

XML Treatment for Solanum truncicola

## Appendix

### Index to numbered collections

Only first collectors in collections made by two or more collectors are listed here; pairs of collectors are listed in the Specimens examined section, while groups of three or more are listed as *et al*. Collections by anonymous collectors without date or other identifying features are not listed here. Full collector strings can be found on Solanaceae Source (http://www.solanaceaesource.org) or on the Natural History Museum Data Portal (http://dx.doi.org/10.5519/0039445).

Abbiw, D.K. GC-43712 (*terminale*).

Abdallah, R. 96/257 (*terminale*).

Achien, L. 639 (*terminale*).

Acocks, J.P.H. 403 (*guineense*); 1374 (*terminale*); 2767, 4254, 4561 (*guineense*); 13666 (*africanum*); 14946, 15362 (*guineense*); 21433 (*africanum*).

Adam, J.G. 3734, 4008, 20447, 27565, 27588, 28907 (*terminale*).

Adames, P. 536, 779 (*terminale*).

Adamson, J. 543 (*runsoriense*).

Aedo, C. 18685 (*africanum*).

Afzelius, A. 167 (*madagascariense*).

Agnew, A.D.Q. 7171 (*runsoriense*).

Aké Assi, L. 9938 (*terminale*).

Alleizette, C. d’ 675-m (*madagascariense*).

Alves Pereira, J. 2640, 3148 (*terminale*).

Ammann, M.Y. 193 (*madagascariense*).

André, E. 8 (*madagascariense*).

Andrews, F.W. A-1430 (*terminale*).

Andriamihajarivo, T. 1256 (*madagascariense*).

Andrianarisata, M. 229 (*madagascariense*).

Andrianjafy, N.M. 273, 497, 1152, 1535 (*madagascariense*).

Annet, E. 401 (*terminale*).

Antilahimena, P. 1405, 1777, 2639 (*madagascariense*); 2694 (*myrsinoides*); 7846 (*truncicola*).

Archbold, M.E. 216, 2525 (*terminale*).

Archer, P.G. 249 (*terminale*).

Argyle, M.C. 25 (*terminale*).

Aridy, J. 77 (*humblotii*).

Armand, M.W. 14 (*madagascariense*).

Arnold, T.H. 594 (*africanum*).

Ash, J.W. 1299 (*runsoriense*); 2932 (*terminale*).

Asonganyi, J.N. 306 (*terminale*).

ATBP 554 (*terminale*).

Atherstone, W.G. 14 (*africanum*).

Auquier, P. 2051, 2434, 3368, 3438 bis, 4473 (*terminale*).

Babault, G. 14, 187, 637, 845, 851 (*terminale*).

Bagshawe, A.G. 432, 573, 679, 1135 (*terminale*).

Baker, H.A. 1001 (*africanum*).

Baker, W.J. 231 (*terminale*).

Balaka, J.L. 1859 B (*terminale*).

Balbo, P.G. 425, 579 (*terminale*).

Baldrati, I. 407, 3532 (*terminale*).

Bally, P.R.O. 1027, 1121, 2756, 4146, 4577, 7786 (*terminale*).

Bamps, P. 160, 392, 763, 1564, 1995, 3143 (*terminale*).

Banda, E.A.K. 466 (*terminale*).

Baron, R. 145, 1150, 1466 (*madagascariense*); 1754 (*imamense*); 1767 (*madagascariense*); 2295 (*imamense*); 2781, 2796 (*madagascariense*); 3090 (*imamense*); 3626, 4210, 4243, 4466, 5148, 7032 (*madagascariense*).

Barthelat, F. 559 (*macrothyrsum*).

Bates, G.L. 1187 (*terminale*).

Battiscombe, E. K-854 (*terminale*).

Batty, M. 983 (*terminale*).

Bauer, P.J. 44 (*terminale*).

Baur, R. 153 (*guineense*).

Bayliss, R.D.A. 1402, 6984, 7484 (*africanum*).

Becquet, A. 674, 861 (*terminale*).

Beentje, H.J. 1798, 2691 (*terminale*).

Beesley, J.S.S. 285 (*terminale*).

Belcher, C. H 138-53 (*terminale*).

Ben, D. van der 548, 909, 1853 (*terminale*).

Benedetto, P. 26 (*terminale*); 390 (*runsoriense*).

Benoît, M.P.O.M. 533 (*terminale*).

Bequaert, J.[C.C.] 1036, 1242, 1715, 1915, 2859, 3344, 3616, 3744 (*terminale*); 4676 (*runsoriense*); 6424, 6531, 7747 (*terminale*).

Bernard, R. 813 (*madagascariense*); 891, 925 (*trichopetiolatum*).

Bernardi, L. 8681 (*terminale*); 11407 (*betroka*).

Bidgood, S. 368, 1674 (*terminale*).

Birkinshaw, C. 978 (*madagascariense*); 1281 (*trichopetiolatum*); 1442 (*madagascariense*).

Bjornstad, A. AB-1888 (*terminale*).

Blackmore, S. 208 (*terminale*).

Blommaert, U. 242 (*terminale*).

Boiteau, P.L. 5240 (*madagascariense*).

Bokdam, J. 2789 (*terminale*).

Bokwala, C. 35 (*terminale*).

Bolus, H. 51 (*guineense*).

Boone, A. 93 (*terminale*).

Borhidi, A. 86260 (*terminale*).

Bos, J.J. 2393 (*terminale*).

Bosser, J.M. 16535, 16852 (*madagascariense*); 17370 (*betroka*).

Boughey, A.S. 190 (*terminale*).

Bouharmont, J. 14177 (*terminale*).

Bouquet, A. 957 (*terminale*).

Bouxin, G. 179, 225, 1272 (*terminale*).

Boyekoli Ebale Congo Expedition 2010 271, 658 (*terminale*).

Brande, J.F. van den 375 (*terminale*).

Brasnett, N.V. 1388 (*terminale*).

Braun, K. [C.] 675, 1955 (*terminale*).

Brédo, H.J. 135, 328, 581, 585, 1038, 1097, 1108, 1157, 1191, 2721 (*terminale*).

Breteler, F.J. 786, 1016, 1205, 1533, 2200, 2440, 2744, 2888, 6502, 6869, 8127, 8395 (*terminale*).

Breyne, H. 981, 1703, 3604 (*terminale*).

Bridson, D.M. 187, 415, 488 (*terminale*).

Brixhe, A. 19 (*terminale*).

Brodhurst-Hill, E. 211 (*terminale*).

Brown, E. 14, 474 (*terminale*).

Bruce, E.M. 463 (*terminale*).

Brummitt, R.K. 8112, 8591, 9975, 14028 (*terminale*).

Bruneel, A. 46 (*terminale*).

Burchell, W.J. 2885 (*guineense*); 4342, 4375, 4389, 4534, 4668 (*africanum*).

Burgt, X.M. van der 1139 (*terminale*).

Burtt, B.D. 1141, 5568 (*terminale*).

Bussmann, R.W. 15257 (*madagascariense*).

Bytebier, B. 28 (*runsoriense*); 2784 (*terminale*).

Cable, S. 179, 3289, 3586, 3780, 3860 (*terminale*).

Callens, H. 691, 2432, 3485 (*terminale*).

Camara, F. 691 (*terminale*).

Cambridge Congo Expedition 1959 171, 428 (*terminale*).

Carmichael, W. 419 (*terminale*).

Carvalho, M.F. do 4232, 4274, 5814, 5814 (*terminale*).

Catat, L.D.M. 1774 (*madagascariense*).

Chandler, P. 959, 1247, 2216, 2644 (*terminale*).

Chapman, H.M. 88 (*terminale*).

Chapman, J.D. 2777, 6377, 6740 (*terminale*).

Chase, N.C. 141, 1839, 3660, 3661, 4872 (*terminale*).

Cheek, M. 5400, 5843, 7039, 7885, 11597 (*terminale*).

Cheseny, C.M.C. 488 (*terminale*).

Chevalier, A. 13825, 14284 bis, 20935, 20992, 22652, 28197 (*terminale*).

Chiovenda, E. 877, 3081 (*runsoriense*).

Christiaensen, A.R. 5, 929, 1212, 1835, 2444 (*terminale*).

Claessens, J. 27, 299, 308, 453, 531, 550, 634, 949 (*terminale*).

Clutton-Brock, T.H. 387, 429 (*terminale*).

Codd, L.E. 1393, 6844 (*terminale*).

Compère, P. 128, 160 (*terminale*).

Cooper, T. 2813 (*terminale*).

Corbisier-Baland, A. [A.P.J.G.] 1034, 1298, 1516 (*terminale*).

Correia, M.F. 2365 (*terminale*).

Cours, G. 851, 1058, 1954, 2807 (*madagascariense*); 3326 (*myrsinoides*); 4275 (*truncicola*); 4323, 4738 (*madagascariense*).

Crawford, R. 354 (*terminale*).

Cremers, G. 643 (*terminale*); 1481 3 (*madagascariense*); 1481-1, 1481-2 (*truncicola*); 3635 (*madagascariense*).

Cribb, P.J. 10080, 10441, 10489, 10579 (*terminale*); 11340 (*terminale*).

Croat, T.B. 28375 (*runsoriense*).

Cummins, H.A. 45 (*terminale*).

Curle, C. 182 (*runsoriense*).

D’Alleizette, C. 988 M (*madagascariense*).

Damaris 2 (*terminale*).

Davies, R.M. 854 (*terminale*).

Dawe, M.T. 672 (*terminale*).

Dawkins, H.C. 751 (*terminale*).

De Wanckel, P. 49, 148 (*terminale*).

Decary, R. 2693, 3729 [a], 4443 (*betroka*); 5068, 5100 [a], 5100 [b], 5235, 5745, 5843 (*madagascariense*); 6276 (*imamense*); 7478, 7479 (*madagascariense*); 9046 (*betroka*); 11017, 16473 (*madagascariense*); 16756, 16757, 16808 (*humblotii*); 17170 (*imamense*); 18118 (*humblotii*).

Demel, T. 1316 (*runsoriense*).

Demeuse, F. 451 (*terminale*).

Dequaire, J. 28012 (*madagascariense*).

Derleth, P. 137 (*madagascariense*).

Deroin, T. 4101 (*terminale*).

Deru 102 (*terminale*).

Descoings, B. 1054 (*madagascariense*); 2666 (*imamense*).

Deville, A. 204, 341 (*terminale*).

Dewèvre, A. 563, 1057, 1057 a (*terminale*).

Dewulf, A. 484, 712, 818 (*terminale*).

Donis, C. 4012 (*terminale*).

Dorr, L.J. 4637 (*humblotii*).

Dowsett-Lemaire, F. 618, 667 (*terminale*).

Dowson, W.J. 17, 608 (*terminale*).

Drège, J.F. 91, 178 (*guineense*); Lycium 7865 (*guineense*).

Drummond, R.B. 2410, 3155, 6998 (*terminale*).

Dubois, L. 156, 432 (*terminale*).

Duchesne, E. 2 (*terminale*).

Dümmer, R.A. 358, 401, 1384, 1387, 4492, 5127, 5608 (*terminale*).

Dyer, R.A. 2010 (*africanum*).

Edwards, S. 3572 (*runsoriense*).

Eggeling, W.J. 695, 1396, 1449, 2127 (*terminale*); 3792 (*runsoriense*); 5655 (*terminale*).

Eilu, G. 247, 293 (*terminale*).

Eimunjeze FHI-72752 (*terminale*).

Elskens, O.A.J. 239 (*terminale*).

Emwiogbon, J.A. FHI-44266 (*terminale*).

Ensermu, K. 1999, 4226 (*runsoriense*).

Eriksson, T. T 930 (*madagascariense*).

Etuge, M. 31, 1324, 1811, 2150, 2754, 3576, 4101 (*terminale*).

Evans, I.M. 413 (*runsoriense*).

Evrard, C. 1788, 1918, 2975 (*terminale*).

Ewango 592 (*terminale*).

Exell, A.W. 191, 219 (*terminale*).

Eyles, F. 5202, 6786 (*terminale*).

Faden, R.B. 67/1, 67/707, 63/722, 266, 483, 857 (*terminale*).

Fanshawe, D.B. 1480, 2817 (*terminale*).

Faucher, P. 32 (*terminale*).

Faulkner, H.G. 4603 (*terminale*).

Festo, L. 702 (*terminale*).

Fishlock, W.C. 43 (*runsoriense*).

Florence, J. 37, 669 (*terminale*).

Floret, J.J. 1600 (*terminale*).

Forsyth-Major, C.I. 15, 55, 115, 325 (*madagascariense*).

Fourcade, G.H. 626, 2178, 2760 (*africanum*).

Fries, T.C.E. 1565, 2434 (*terminale*).

Friis, I. 683 (*terminale*); 3610 (*runsoriense*); 3842 (*terminale*); 5546 (*runsoriense*); 7011 (*terminale*).

Frimodt-Moller, C. NG-041 (*terminale*).

Froment, D. 98, 451, 539 (*terminale*).

Fyffe, R. 20 (*runsoriense*).

Gaetan, M.F.R. 93 (*terminale*).

Gaillez, L. 281 (*terminale*).

Galdermans, M. 11 (*terminale*).

Galpin, E.E. 2690, 2854, 10687 (*africanum*); 12630 (*guineense*).

Garnier, A. 134 (*madagascariense*).

Garside, S. 484 (*africanum*).

Gautier, L. 5707 (*sambiranense*).

Gautier-Beguin, D. 1133 (*terminale*).

Geerling, C. 2489 (*terminale*).

Gentry, A.H. 11846, 11852 (*madagascariense*); 33002, 33014 (*terminale*).

Gerard, P. 1286, 1813, 2519, 2812, 3562, 3681, 3918, 4670, 5473 (*terminale*).

Gereau, R.E. 3522, 5457, 5930, 6891 (*terminale*).

Germain, R.G.A. 2007, 3323, 5920 (*terminale*).

Gerrard, W.T. 502, 539, 1413 (*terminale*).

Ghesquiere, J. 3486 (*terminale*).

Gilbert, E.F. 129, 138 (*runsoriense*).

Gilbert, G. 465, 1608, 2308 (*terminale*).

Gilbert, M.G. 187 (*terminale*); 1098, 1698 (*runsoriense*); 6880, 8417 (*terminale*).

Gilbert, V.C. 3518 (*terminale*).

Gillardin, J. 202, 317, 586, 1094 (*terminale*).

Gille, P. [J.M.C.] 50, 267 (*terminale*).

Gillet, J. 717, 3016, 3607, 3779 (*terminale*).

Gillett, J.B. 5149 (*terminale*); 15034 (*runsoriense*); 16771, 17990, 19032 (*terminale*).

Gilliland, H.B. 314, B314 (*terminale*).

Giorgi, S. de 69, 661, 664, 745, 996, 1344, 1433, 1579, 1824 (*terminale*).

Glover, E.C. 1358 (*terminale*).

Glover, P.E. 765, 1092, 1358 (*terminale*).

Gobbo, G. 671, 674 (*terminale*).

Godman, E.M. 231 (*terminale*); 309 (*runsoriense*).

Goldblatt, P. 4326 (*guineense*).

Goldsmith, B. 99/61 (*terminale*).

Goodall, L.M. 19 (*terminale*).

Goossens, V. 1522, 2384, 2647, 2716, 4151, 4209, 4312, 4318, 4377, 6152 (*terminale*).

Gossweiler, J. 5092 (*terminale*).

Goyder, D.J. 7749 (*terminale*).

Graer, P.A.M. de 550 (*terminale*).

Graham, M.D. 1752 (*terminale*).

Greenway, P.J. 9016, 9700, 13883 (*terminale*).

Grevé, H. 241 (*sambiranense*).

Grimshaw, J.M. 93- 79 (*terminale*).

Grote, M. 8066 (*terminale*).

Guinea, E. 1094, 1144, 1146, 1644, 1666, 1766 (*terminale*).

Gutzwiller, R. 1149, 1372, 1512, 1541, 2140, 2641, 2703, 2883, 3174, 3406, 3705 (*terminale*).

Haarer, A.E. 995 (*terminale*).

Haba, P.M. 217 (*terminale*).

Hall, G.P. 98 (*terminale*).

Hallé, F. 1715, 1795 (*terminale*).

Hallé, N. 2612, 3125, 5118, 5868 (*terminale*).

Hamdi, O. 38 (*terminale*).

Hanekom, W.J. 1152 (*guineense*).

Hansen, O.J. 790 (*terminale*).

Harden-Smith, M.C. 12 (*terminale*).

Harger, R.L. s2676 (*terminale*).

Harrington, G.N. 302 (*terminale*).

Harris, B.J. BJH-5155 (*terminale*).

Harris, T. 532 (*terminale*).

Hart, T. 863 (*terminale*).

Harvey, W.H. 75, 494 (*africanum*).

Harvey, Y.B. 272 (*terminale*).

Haugen, T. 440 (*terminale*); 1291, 1507 (*runsoriense*).

Hauman, L. 199 (*terminale*).

Hedberg, O. 1143 (*terminale*).

Hédin, L. 363, 1220 (*terminale*).

Hendrickx, F.L. 603 bis, 1288, 3615, 3844, 4060, 4246, 4602 (*terminale*).

Henlocker, D. 246, 342 (*terminale*).

Hepper, F.N. 4976 (*terminale*); 7320, 7321, 7323 (*africanum*); 7406 (*terminale*).

Herbier de la Station Agricole de l’Alaotra 2807 (*madagascariense*); 4275 (*humblotii*).

Herlocker, D. 246, 342 (*terminale*).

Hildebrandt, J.M. 3666 (*madagascariense*); 3954 (*truncicola*).

Hindorf, ? 805 (*terminale*).

Hislop, A. 343 (*terminale*).

Hoffman, P. 226 (*madagascariense*).

Holst, C. 8927 (*terminale*).

Homolle, A.M. 1122 (*truncicola*).

Hornby, A.J. 36 (*terminale*).

Hulstraert, R.P. 246, 567, 592, 882, 887, 891, 1392 (*terminale*).

Humbert, H. 1265, 3049, 3172, 3940, 6050, 6302 (*madagascariense*); 6812 (*imamense*); 6912 (*madagascariense*); 7915, 8333, 8848, 10617 (*terminale*); 11901, 12240, 13429, 14133 (*madagascariense*); 14290 (*imamense*); 14298 [b] (*betroka*); 17746, 18109, 18163 (*madagascariense*); 18586, 18709, 19072 (*sambiranense*); 21939 (*madagascariense*); 22253, 22504, 22863 (*myrsinoides*); 23075 (*trichopetiolatum*); 23142 (*myrsinoides*); 23653 (*trichopetiolatum*); 24533 (*madagascariense*); 25714 (*trichopetiolatum*); 28423 (*madagascariense*); 29627, 29654 (*betroka*); 31365, 31469, 31596 (*myrsinoides*); 31597, 31609 (*madagascariense*).

Humblot, L. 284 (*terminale*); 387 (*macrothyrsum*); 481, 482 (*madagascariense*); 509 (*humblotii*).

Humphreys, G.N. 1204 (*runsoriense*).

Hunt, L.V. 64 (*terminale*).

Hylander, K. 139 (*runsoriense*).

Hørlyck, V. TZ-454 (*terminale*).

Irvine, F.R. 1172, 1812 (*terminale*).

Irwin, P.H. 139 (*terminale*).

Jackson, J.K. 3045 (*terminale*).

Jacquemin, H. H-595-J, H-675-J (*madagascariense*).

Jacques-Felix, H. 913, 2385, 4709, 5109 (*terminale*).

Jaeger, P. 8531 (*terminale*).

Jaff, B. 39 (*terminale*).

Jans, H. 621, 621 bis (*terminale*).

Jard. Bot. Tananarive 4628 (*truncicola*); 4812 (*madagascariense*); 6083 (*betroka*).

Jarrett, T. 253 (*terminale*).

Jeanty, C.E. 21, 28, 47 b, 48 (*terminale*).

Jeffrey, C. 36, 199 (*terminale*).

Jesperson, K. 68, 134 (*terminale*).

Jex-Blake, A.J. 28 (*runsoriense*); 332 (*terminale*); 3304 (*runsoriense*).

Joana, B. 8996 (*terminale*).

Johnson, S.M. 930 (*africanum*).

Johnson, W.H. 158 (*terminale*).

Jongkind, C. 1638, 10971 (*terminale*).

Juintas 1018 (*terminale*).

Junod, H.A. 1499 (*terminale*).

Jurion, F. 107 (*terminale*).

Kamau, W. 743 (*terminale*).

Kandt, R. 147 (*terminale*).

Kassner [Kässner], T. [L.C.T.] 804, 2760, 3250 (*terminale*).

Katende, A.B. 221 (*terminale*); 345-A (*runsoriense*); 571, 964, 1526, 3197 (*terminale*).

Kayombo, C.J. 1414 (*terminale*).

Kendall, R. 25 (*runsoriense*).

Keraudren, M. 224, 229 (*madagascariense*).

Kerfoot, O. 667, 741, 1639 (*terminale*); 2000 (*runsoriense*); 2116, 3191, 3234, 3251, 3730, 3865, 4340, 4723 (*terminale*).

Kindeketa, W. 295, 526, 553, 818 (*terminale*).

Kirika, P. 5, 29 (*terminale*).

Kisena, C.M. 137, 842 (*terminale*).

Kluguess, L.M. 67 (*terminale*).

Knapp, S. 9811 (*terminale*).

Koechlin, J. 6257 (*terminale*).

Koning, J. de 241 (*terminale*).

Koritschoner, H. 609, 866, 1270, 1351 (*terminale*).

Kotozafy, A. 298, 319 (*madagascariense*).

Koufani, A. 85, 215 (*terminale*).

Kuchar, P. 7830, 25227 (*terminale*).

Kyoto University Expedition 325, 327 (*terminale*).

La Croix, I.F. 5027 (*terminale*).

Lacomblez, M. 25 (*terminale*).

Laidley & Co. 274 (*africanum*).

Lane, P. 115 (*terminale*).

Laurent, J.F. 358, 542, 562, 602, 1286, 1579 (*terminale*).

Laurent, M. 701 (*terminale*).

Lawton, R.M. 2155 (*terminale*).

Le Testu, G. 3910, 3971, 5298, 8069 (*terminale*).

Leakey, C.L.A. 124 (*runsoriense*).

Léandri, J. 297 (*sambiranense*).

Lebrun, J.P. 266, 271, 307, 438, 1073, 1354, 1972, 2483, 2828, 3096, 3941, 4788, 4933, 5312, 5705, 8408, 8692, 8856, 8979, 11269 (*terminale*).

Leemans, J. 78 (*terminale*).

Leeuwenberg, A.J.M. 2440, 2518, 6779, 8068, 8299, 11403 (*terminale*).

Leighton, F.M. 987 (*guineense*).

Lejoly, J. 1444, 2339, 3863, 4774 (*terminale*).

Lemaire, H. 202, 316 (*terminale*).

Leonard, A. 802, 932, 972, 2562, 2826, 2899, 3243, 5974, 5976 (*terminale*).

Leroy, J.F. 3 (*betroka*).

Letouzey, R. 1590, 1686, 3592, 3933, 4661, 4712, 5221, 8534, 10216, 10894, 11385, 11471, 12219, 15019 (*terminale*).

Lewalle, J. 981, 2446, 3249, 5343, 6352 (*terminale*).

Lewis, B. 639 (*madagascariense*); 1083 (*truncicola*).

Leyser, E.A. de 162 (*terminale*).

Liben, L. 2065, 2571 (*terminale*).

Liebenberg, L.C.C. 1637 (*runsoriense*); 7861, 8120 (*africanum*).

Lind, E.M. 2103, 2974 (*terminale*).

Linder, D.H. 1785 a, 2432 (*terminale*).

Lisowski, S. 15028, 15335, 16938, 18587 (*terminale*).

Long, F.R. 395 (*africanum*).

Louis, J.L.P. 146, 245, 368, 981, 2394, 2397, 3795, 4328, 6362, 7375, 7498, 9280, 9726, 12735, 14146, 15092, 15835, 15855 (*terminale*).

Loveridge, M.V. 75, 108, 639 (*terminale*).

Lovett, J.C. 632 (*terminale*).

Lowry, P.P. 4286 (*truncicola*); 6120 (*myrsinoides*).

Ludovic, R. 110 (*humblotii*).

Luja, E. 112, 151 (*terminale*).

Luke, [W.R.] Q. 7355, 7822, 13206 (*terminale*).

Luxen, F. 384 (*terminale*).

Lye, K.A. 1340 (*runsoriense*); 1950, 4017 (*terminale*).

Lynes, H. Fr-57 (*terminale*).

Maas Geesternanus, R.A. 4905, 5246, 5378, 5525, 5740, 5894 (*terminale*).

Mabberley, D.J. 199, 1093, 1346, 3962-A (*terminale*).

Machangu 67 (*terminale*).

MacOwan, P. 1930 (*africanum*); 2002 (*guineense*); 3369 (*africanum*).

Magogo, F.C. 1517 (*terminale*).

Maitland, T.D. 1690, 1960 (*terminale*).

Makany, L. 1528, 1592, 1673 (*terminale*).

Malaisse, F. 2378, 16813 (*terminale*).

Malchair, L. 249, 300, 339, 390 (*terminale*).

Malcomber, S.T. 1353 (*truncicola*); 1581, 1998 (*madagascariense*); 2440 (*truncicola*); 2697 (*myrsinoides*).

Manktelow, M. 91009 (*terminale*).

Mann, G. 10, 62, 274 (*terminale*).

Masens, B. 1341 (*terminale*).

Massawe, G. DA 75, 478, 708, 711 (*terminale*).

Mbago, F.M. 1109, 1729 (*terminale*).

Mbailwa 106 (*terminale*).

Mbani, J.M. 119, 142 (*terminale*).

Mbatchou, G.T. 21 (*terminale*).

Mbuthia, K.W. 30, 185 (*terminale*).

McPherson, G. 14591, 16513 (*madagascariense*).

Mearns, E.A. 1416 (*runsoriense*); 1821, 2457 (*terminale*).

Medley Wood, J. 559, 559 (*terminale*); 6390 (*africanum*); 10241 (*terminale*).

Meeuse, A.D.J. 9661 (*africanum*).

Menavanza, F. 62 (*terminale*).

Merello, M. 1147 (*terminale*).

Merwe, P. van den 996 (*guineense*).

Mesfin Tadese 759 (*terminale*); 1684, 2691, 4823, 7212, 9098 (*runsoriense*).

Mesili, F. 56 (*terminale*).

Messmer, N. 46, 696, 744, 857 (*madagascariense*).

Mettam, R.V. 178 (*terminale*).

Meurillon, A. 931 (*terminale*).

Meyer, F.G. 7537 (*runsoriense*).

Mezili, P. 56 (*terminale*).

Mgaza, C.D. 120 (*terminale*).

Mhoro, B. UMBCP-474 (*terminale*).

Michel, E. 987 bis, 1571 bis, 1572, 2604, 2915, 3159, 3324 (*terminale*).

Michel, G. 5794, 5879 (*terminale*).

Mildbraed, G.W.J. 8619, 8838, 9057, 9264, 9294 (*terminale*).

Miller, A.G. 3025 (*terminale*).

Miller, A.K. 448 (*terminale*).

Miller, J.S. 3413 (*madagascariense*); 4072 (*trichopetiolatum*); 4183, 4329, 4360, 4563 (*madagascariense*); 4675 (*myrsinoides*).

Millman, C. 16 (*runsoriense*).

Milne-Redhead, E. 3723, 8909 (*terminale*).

Milton, O. 56 (*terminale*).

Mlangwa, J.A. 1094, 1371, 1596 (*terminale*).

Mogg, A.O.D. 17435 (*terminale*).

Moller, A.F. 95, 163, 404, 516 (*terminale*).

Monod, T. 11727 (*terminale*).

Mooney, H.F. 6156, 6380, 6475, 6712, 7016 (*runsoriense*).

Morat, P. 2265 (*madagascariense*); 4946 (*myrsinoides*).

Mortehan, M.G. 125, 364, 395, 940, 959, 1064 (*terminale*).

Mortimer, M.J. T-114 (*terminale*).

Morton, J.K. SL 2959, GC 8387 (*terminale*).

Mosango 541 (*terminale*).

Moureau, J. 32 (*terminale*).

Mullenders, W. 1520, 1632, 1701 (*terminale*).

Müller-Hohenstein, K. 1353 (*terminale*).

Mungai, G.M. 77 (*terminale*).

Mwachala, G. 315, EW-1251, EW-3543 (*terminale*).

Mwangoka, M.A. 66, 289, 418, 488, 545, 578, 1840, 2693, 3305, 4530, 5121, 5638 (*terminale*).

Napier, E.R. 322, 1132 (*terminale*); 2709 (*runsoriense*); 2926 (*terminale*); 5130 (*runsoriense*); 5337 (*terminale*).

Nathass, R.M. 619, 675 (*terminale*).

Nattrass, M.S. 1457 (*terminale*).

Nattrass, R.M. 320, 345, 1400 (*terminale*).

Ndam, N. 1347 (*terminale*).

Ndolo E, S.T. 52, 196, 410 (*terminale*).

Negri, G. 120 (*terminale*).

Nek, F.I. van 2021 (*madagascariense*).

Nemba, J. 786 (*terminale*).

Newbould, J.B. 5699, 5897 (*terminale*).

Ngoundai, S. 222, 404 (*terminale*).

Nicoll, M. 139, 211, 236 (*madagascariense*).

Nning, J. 200, 384 (*terminale*).

Norman, E.M. 222 (*terminale*).

Noyes, R.D. 975 (*madagascariense*).

Nshorere 121 (*terminale*).

Nsimundeie 2490 (*terminale*).

Nusbaumer, L. 831, 860 (*sambiranense*); 1637 (*madagascariense*).

Nuvunga, A. 318 (*terminale*).

Oldenburg, F.P. 1542 (*africanum*).

Olorunfemi, J. 30589 (*terminale*).

Onana, J.-.M. 1982, 2897 (*terminale*).

Opiko, E. 8758 (*terminale*).

Osmaston, H.A. 1549 (*runsoriense*).

Overlaet, F.G. 1086, 1145 (*terminale*).

Padwa, J.H. 65 (*terminale*).

Parker, R.N. 3652 (*africanum*); 4174 (*guineense*); 4403 (*africanum*).

Parnell, D. 2284 (*terminale*).

Patel, I.H. 479 (*terminale*).

Pauwels, L. 778, 1164, 1919 (*terminale*).

Pavlov, ? [Ethiopia] 97/113 (*terminale*).

Pawek, J. 1906, 6437, 8927, 11601, 12349 (*terminale*).

Pedersen, C. 225 (*terminale*).

Peltier, J. 2676 (*truncicola*); 3069 (*betroka*); 4648 (*madagascariense*).

Peltier, M. 980 (*humblotii*).

Perdue, R.E. 8252, 9288 (*terminale*).

Pereira, A. 1358, 1585 (*terminale*).

Pereira, J.A. 2640 (*terminale*).

Pérez Viso, R. 623 (*terminale*).

Perrier de la Bâthie, J. [M.H.A.] 3 (*madagascariense*); 18 (*myrsinoides*); 1332 (*imamense*); 2324, 2580 (*sambiranense*); 8686, 8699, 8702, 8711 (*madagascariense*); 8718 (*myrsinoides*); 9574, 9577, 9579, 9615 (*imamense*); 13022 (*sambiranense*); 14490 (*truncicola*); 15364 (*madagascariense*); 16830 (*imamense*); 17065 (*truncicola*); 17291 (*madagascariense*); 17783 (*sambiranense*); 18074 (*madagascariense*).

Phillips, E. 2164, 3808 A, 3899, 3993 B, 4612 (*terminale*).

Phillipson, P.B. 4933 (*terminale*); 5785 (*truncicola*).

Pierlot, R. 1461, 1492, 2006, 2008, 2771, 2947, 3090 (*terminale*).

Pignal, M. 1143 (*terminale*).

Pillans, N.S. 5436 (*guineense*).

Pittery, H. 773, 774 (*terminale*).

Plaizier, A.C. 1321 (*terminale*).

Poc’s, T. 88/179 /W, 89/002/C, 89/011/C (*terminale*).

Polhill, R.M. 250 (*runsoriense*); 297, 1479, 2341 (*terminale*).

Pollard, B.J. 953 (*terminale*).

Preuss, P.R. 397 (*terminale*).

Prins, W. 349 (*terminale*).

Procter, J.E.A. 1055 (*terminale*).

Purseglove, J.W. P 315 (*runsoriense*); P 358, P 759, P 1654, P 3025 (*terminale*).

Pynaert, L. 274, 495, 847, 1094, 1513 (*terminale*).

Quarré, P. 39, 698, 7051 (*terminale*).

Quintas, F.J.D. 920 (*terminale*).

Rabevohitra, R. 4150 (*madagascariense*).

Rakoto, R. 6481 (*truncicola*).

Rakotomalala, L. 220 (*madagascariense*).

Rakotomalaza, P.J. 1398 (*madagascariense*).

Rakotonasolo, F. RNF-1535 (*madagascariense*); RNF-1539 (*truncicola*); RNF -674 (*ivohibe*).

Rakotondrafara, A. 111 (*madagascariense*).

Rakotovao 9981, 9990, 12290 (*madagascariense*).

Rakotovao, C. 2404, 2458 (*madagascariense*); 5128 (*imamense*).

Rakotozafy, A. 2202 bis (*madagascariense*).

Ralimanana, H. 96 (*humblotii*); 449 (*madagascariense*).

Ranaivojaona, R. 969, 1185 (*madagascariense*).

Randrianaivo, R. 47, 59 (*madagascariense*).

Randrianasolo, A. 107 (*trichopetiolatum*); 142 (*myrsinoides*); 1163 (*truncicola*).

Randrianasolo, S. 580 (*sambiranense*).

Randriantafika, F. 15, 176 (*madagascariense*).

Ranirison, P. 330 (*sambiranense*); 838 (*madagascariense*); 1032 (*sambiranense*).

Rasoavimbahoaka, F. 8 (*trichopetiolatum*); 314 (*myrsinoides*); 476 (*madagascariense*).

Rasoazanany, H. 93 (*truncicola*).

Ravelonarivo, D. 418 (*madagascariense*); 475 (*myrsinoides*); 556 (*madagascariense*); 636 (*trichopetiolatum*); 792 (*trichopetiolatum*); 1889 (*myrsinoides*); 3042 (*madagascariense*).

Raynal, J. 9592, 9699, 9853, 20648 (*terminale*).

Rayner, W.R. 48 (*terminale*).

Razafitsalama, J. 1145 (*madagascariense*).

Razakamalaka, R. 104 (*madagascariense*).

Razanatsima, A. 28, 598 (*truncicola*).

Reekmans, M. 698, 1247, 2639, 2939, 6747, 6848, 7184 (*terminale*).

Renya, S. 77 (*terminale*).

Réserves Naturelles Madagascar [collections often followed by suffix RN and/or another collector name] 1598, 2475, 2738, 3257, 3388 (*madagascariense*); 3516, 3523 (*ivohibe*); 4480, 4487, 4487, 4499 (*madagascariense*); 5575 (*truncicola*); 5766 (*sambiranense*); 6481 (*truncicola*); 8309, 8388, 8665, 9063, 9177, 9467 (*madagascariense*); 9918 (*truncicola*); 9981, 9990 (*madagascariense*).

Reygaert, F. 153, 226, 380, 1151, 1154, 1314, 1360 (*terminale*).

Richards, M [Mrs H.M.] 830, 4231, 7639, 7692, 10596, 14194, 14308, 14331, 15628, 18480, 18618, 19926, 20353, 21732, 21871, 22005, 24031, 25346, 26922, 27244 (*terminale*).

Richards, P.W. 3373 (*terminale*).

Robertson, N.F. K-9 (*terminale*).

Robertson, S.A. 377, 445, 763, 2148 (*terminale*).

Roberty, G. 6680, 15478, 15837 (*terminale*).

Robin, R. 67 (*terminale*).

Robinette, W.L. 3125 (*terminale*).

Robson, N.K.B. 1662 (*terminale*).

Robyns, W. [F.H.E.A.W.] 684, 734, 3125 (*terminale*).

Rogers, C.G. 358 (*terminale*).

Rogers, F.A. 10182 (*terminale*); 15511 (*africanum*); 18111, 21326, 26252 (*terminale*); 28014 (*africanum*).

Roitzsch, J.R. 7825 (*runsoriense*).

Ross, J.H. 1991 (*africanum*).

Ross, R. 227 (*terminale*); 578, 800, 957 (*runsoriense*).

Rounce, N.V. 534 (*terminale*).

Runyinya, B. 958 (*terminale*).

Rwaburindore, P.K. 549, 3430 (*terminale*).

Ryan, P. 259 (*terminale*).

Sabaya, W.D. DSM-498 (*terminale*).

Sacleux, R.P. 802 (*terminale*).

Sajy 4499 (*madagascariense*); 5766 (*sambiranense*).

Salter, T.M. 303/8 (*guineense*); 303/6, 377/52 (*africanum*); 993 (*guineense*).

Salubeni, A.J. 1195 (*terminale*).

Satabie, B. 1119 (*terminale*).

Saunders, K. 3 (*africanum*); 9 (*terminale*).

Sauquet, H. 86 (*madagascariense*).

Savory, H.J. FHI 25255 (*terminale*).

Schaijes, M. 2088 (*terminale*).

Schatz, G.E. 1713 (*madagascariense*); 1820, 1987 (*humblotii*); 2598 (*truncicola*); 3391 (*myrsinoides*); 4018 (*madagascariense*).

Scheepers, J.C. 396 (*terminale*).

Schimper, G.H.W. 201, 310, 480, 977 [b] (*terminale*); 1227 (*runsoriense*); 1727 (*terminale*).

Schlechter, F.R.R. 640 (*africanum*); 7896 (*guineense*); 12974 (*terminale*).

Schlieben, H.J. 1653, 3254, 4398, 7555 (*terminale*); 8197 (*madagascariense*).

Schnell, R. 4079, 5341 (*terminale*).

Schweinfurth, G.A. 3498 (*terminale*).

Scott-Elliot, G.F. 1825 (*madagascariense*); 6800 (*terminale*); 7733 (*runsoriense*).

Seki, T. JKCAT-635 (*terminale*).

Senni, L. 1905, 2034 (*runsoriense*).

Seret, F. 67, 576 (*terminale*).

Seyrig, A. 0-371 (*betroka*); 156 (*imamense*); 371 (*betroka*).

Shabani, S. 318 (*terminale*).

Sheil, D. 1351 (*terminale*); 1813 (*runsoriense*).

Sidey, J.L. 3100 (*africanum*).

Simon, B.K. 769 (*terminale*).

Simon, G. 671 (*terminale*).

Skene, E. 121 (*terminale*).

Smeds, H. 394, 1350 (*runsoriense*).

Smeyers, F. 72 (*terminale*).

Snowden, J.D. 170, 584, 889, 889 a, 889 b, 890, 1919 (*terminale*).

Solazzo, ? [Ethiopia] 280, 281, 284 (*terminale*).

Soleman, R. Herb. Amani-7244 (*terminale*).

Sosef, M.S.M. 2176 (*terminale*).

Soundy, W.W. 14 (*runsoriense*).

Spitaels, R. 119 (*terminale*).

St. Clair Thompson, G.W. 977 (*terminale*).

Staner, P. 881, 1528 (*terminale*).

Stauffer, H.U. 419, 1043 (*terminale*).

Stevart, T. 8 (*terminale*).

Stewart, [J.?] 266 (*africanum*).

Stewart, R.B. B-19 (*terminale*).

Steyaert, R. 454, 543, 601, 770 (*terminale*).

Stolz, A. 355, 1035, 1159, 1514, 2315 (*terminale*).

Strey, R.G. 8863 (*terminale*); 11187 (*africanum*).

Strid, A. 3113 (*terminale*).

Stuhlmann, F. 1329 (*terminale*).

Sunderland, T.C.H. 1055, 1348 (*terminale*).

Swynnerton, C.F.M. 86 (*terminale*).

Symes, Y.E. 84 (*terminale*).

Synge, P.M. 892, 1444, 1877 (*runsoriense*).

Synnott, T.J. 617 (*terminale*).

Szafranski, F. 1277 (*terminale*).

Tadjouteu, F. 575 (*terminale*).

Tah, K. 291 (*terminale*).

Talbot, P.A. 3211 (*madagascariense*).

Tanner, R.E.S. 3221, 4703, 4930 (*terminale*).

Taton, A. 151 (*terminale*).

Taylor, G. 1408 (*runsoriense*); 1550, 1875, 2022, 2421 (*terminale*); 2913 (*runsoriense*).

Tchiengue, B. 1924, 2232, 3136 (*terminale*).

Tchouto, P. 478 (*terminale*).

Tekle, H.H. 220 (*terminale*).

Tekwe, C. 128 (*terminale*).

Tepe, E.J. 2783 (*terminale*).

Tesfaye, A. 408 (*terminale*).

Thanes, A.S. 650 (*runsoriense*).

Thode, J. A- 2494 (*africanum*).

Thomas, A.S. 650, 767 (*runsoriense*); 2520, 4280 (*terminale*).

Thomas, D. 2176 (*terminale*).

Thomerson, A.D.M. 835 (*runsoriense*).

Thompson, S.A. 1555 (*terminale*).

Thonet, J. 154 (*terminale*).

Thonner, F. 96 (*terminale*).

Thulin, M. 1502 (*runsoriense*); 2916 (*terminale*); 4064 (*runsoriense*).

Tisserant, C. 250, 286, 1040, 1251-1040, 1861, 2078, 3408, 3490, 3491 (*terminale*).

Torre, A.R. 6283, 14805 (*terminale*).

Tosh, J. 100 (*humblotii*).

Tothill, B.H. 2260 (*runsoriense*).

Toussaint, L. 493 (*terminale*).

Townsend, C.C. 2378 (*terminale*).

Troupin, G. 3083, 3196, 3795, 4947, 5577, 5620, 5747, 5975, 6405, 6631, 7115, 7517, 7547, 7787, 8044, 8061, 8773, 9401, 9614, 9826, 10162, 10597, 11501, 11702, 12493, 14630 (*terminale*).

Trut, ? 576 (*terminale*).

Tschinaye, V. 136 (*terminale*).

Tuley, P. 1058 (*terminale*).

Turk, D. 341 (*truncicola*).

Tweedie, M. [Mrs] 714, 4135, 4280 (*terminale*).

Uehara, S. 180 (*terminale*).

Uhlig, C. 548 (*terminale*).

Ujor, E.U. FHI-30026 (*terminale*).

Van Breda, P.A.B. 4164 (*guineense*).

Van der Werff, H. 12591, 12689 (*madagascariense*).

Van Someren, G.R.C. 9835 (*terminale*).

Vanderyst, H. 2881 bis, 8980, 9814, 11349, 12861, 12945, 14229, 14444, 17358, 19168 bis, 30180 bis (*terminale*).

Vatova, A. 15, 639 (*runsoriense*); 1896 (*terminale*); 2362 (*runsoriense*).

Vaughan, J.H. 2634 (*terminale*).

Veken, P. van der 9577 (*terminale*).

Verdcourt, B. 653 (*terminale*).

Vermoesen, F.C.M. 964, 1470, 1520, 1564, 1719 (*terminale*).

Vesey-Fitzgerald, L.D.E.F. 6393 (*terminale*).

Vigreux, M. 15491 (*madagascariense*).

Viguier, R. 758 (*madagascariense*).

Villiers, J.F. 636, 654 (*terminale*).

Vin, P. 45 (*terminale*).

Volkens, G. 2265 (*terminale*).

Vorontsova, M.S. 56, 93, 163 (*terminale*); 498 (*madagascariense*).

Vuyk, D. 51 (*terminale*).

Wagemans, J. 562, 2172 (*terminale*).

Wallace, G.B. 897, 1164 (*terminale*).

Webb, H.R. 62 (*terminale*).

Webster, M.V.B. 8918, AH-09960 (*terminale*).

Wellens, R.P. 286 (*terminale*).

Wells, N.J. 2780 (*africanum*).

Welwitsch, F.M.J. 6032, 6081, 6098, 6106, 6107 (*terminale*).

West-Skinn, R. 19 (*terminale*).

Westphal, E. 2690, 3157 (*runsoriense*).

White, F. 3442 (*terminale*).

Whittall, E. 28 (*terminale*).

Wild, H. 6343 (*terminale*).

de Wilde, J. [M.H.J.R.] 367 (*terminale*).

de Wilde, J.J.F.E. 55 (*runsoriense*); 2327, 5310, 7173, 7893, 9942 (*terminale*).

de Wilde, W.J.J.O. 2023, 2327, 6315 (*terminale*); 7369, 8360, 8480, 9213, 10059 (*runsoriense*).

Williams, G.R. 152 (*terminale*).

Williams, S.L. 192 (*guineense*).

Willy, P. 3771 (*terminale*).

Wilms, F. 3435 (*africanum*).

de Witte, G.F. 3315, 7789, 8006, 8843, 9015, 9866, 9919, 10220, 10827, 10976, 11084, 11842, 11977, 12095, 12111, 12132, 12153, 12531, 12689, 12701, 13040, 13224 (*terminale*).

Wohlhauser, S. 399, 648, 786 (*madagascariense*).

Wolley-Dod, A.H. 1018 (*africanum*).

Wood, D. 816 (*runsoriense*).

Wood, J.M. [see Medley Wood, J.]

Wood, J.R.I. 1864 (*terminale*).

Wright, J.M. 6 (*terminale*).

Yamada, T. 257 (*terminale*).

Yeoman, G.H. 131 (*runsoriense*).

Zapfack, L. 682 (*terminale*).

Zenker, G. 3473, 3681, 3884, 4675 (*terminale*).

Zerny, H. 350 (*terminale*).

Zeyher, C.L.P. 75 (*africanum*); 508, 3471 (*guineense*); 3472 (*africanum*).

Zimmer, F. 183 (*terminale*).

Zimmermann, A. 8065, 8076 (*terminale*).

van Zyl, L. 3136 (*guineense*).
